# Development of efficient genetic toolkits and heterologous chassis for bioprospecting of myxobacteria

**DOI:** 10.1038/s41467-026-74908-x

**Published:** 2026-06-29

**Authors:** Wen-Juan Zhang, Jia-Qi Hu, Rui-Juan Li, Duo-Hong Sheng, Xin-Jing Yue, Qunxin She, Youming Zhang, Yue-Zhong Li, Li Zhuo, Changsheng Wu

**Affiliations:** 1 State Key Laboratory of Microbial Technology, Institute of Microbial Technology, Shandong University, Qingdao, P.R. China; 2 Shenzhen Research Institute of Shandong University, Shenzhen, P.R. China

**Keywords:** Bacterial genetics, Metabolic engineering, Bacterial synthetic biology

## Abstract

Myxobacteria are renowned producers of structurally diverse bioactive natural products. Motivated by a comprehensive inventory of myxobacterial biosynthetic gene clusters (BGCs), we develop efficient genetic toolkits and construct a robust heterologous expression chassis. We identify a pair of *Myxococcus*-derived recombinases, designated MxRecET, that enables efficient genome editing in the model strain *Myxococcus xanthus* DK1622. The synergistic combination of MxRecET recombineering with the transposon-associated nuclease TnpB (ISDra2) enables versatile genetic manipulations, including seamless deletion of large DNA fragments (up to 200 kb), tandem editing of dual loci, and flexible single-nucleotide substitutions within a streamlined two-week workflow. This integrated technology, termed MxDIRECT, facilitates the rational engineering of DK1622 into a plug-and-play chassis designated MxPKS, featuring enhanced growth properties, elimination of competing pathways, an improved precursor supply, and increased robustness. The effectiveness of MxPKS is demonstrated through heterologous expression of four polyketide synthase BGCs, leading to the discovery of multiple unknown polyketides. This work establishes a foundation for accelerated bioprospecting of myxobacteria.

## Abstract (web summary)

Myxobacteria are renowned producers of structurally diverse bioactive natural products but are difficult to engineer. Here the authors create a robust heterologous expression chassis and develop MxDIRECT, a recombineering strategy for scarless marker-free expression of biosynthetic gene clusters.

## Introduction

Myxobacteria (the phylum *Myxococcota*^1^) are well known for their high capacity to produce a large array of specialized secondary metabolites with complex skeletons, pronounced bioactivity, and specific mode of action^2^. Genome mining has revealed numerous untapped biosynthetic gene clusters (BGCs) that do not match known compounds^3–5^, placing this microbial community at the forefront of research on microbial natural products (NPs). However, studies of most non-model myxobacteria are full of challenges because of intrinsic characteristics such as slow growth, limited cultivability, and genetic recalcitrance^6,7^. Transplanting BGCs from undomesticated and genetically intractable bacteria into genetically tractable hosts for heterologous expression is a favored strategy to improve yield and/or activate silent BGCs. However, the strategic heterologous expression of myxobacterial BGCs has been largely hindered by the lack of efficient chassis. Although several strains, such as *Pseudomonas putida*^8^, *Streptomyces coelicolor*^9^, and *Schlegelella brevitalea*^10^, have been sporadically employed as surrogates for myxobacterial BGCs, their application has remained limited, probably owing to poor pathway compatibility. The model myxobacterium *Myxococcus xanthus* DK1622 is a promising platform for the development of an effective chassis dedicated to myxobacterial NPs. It is the best-studied strain in the paradigm of myxobacteria, with preferable physiological traits such as rapid growth (with a generation time of 3–4 h)^11^. In addition, it is a confirmed producer of structurally diverse NPs^2^, demonstrating its robust secondary metabolic capabilities and the availability of common biosynthetic precursors. More importantly, it has a track record of success in terms of heterologous expression of different myxobacterial NPs from both closely and distantly related taxa^2,12^. In addition, we have characterized a group of constitutive promoters employable in DK1622, which also set the stage for yield improvements and/or the activation of cryptic BGCs^13^. Nevertheless, the wild-type DK1622 strain requires improvement, particularly owing to its severe clumping growth and complicated metabolic background.

The establishment of a chassis cell is heavily contingent upon the availability of potent genetic tools. Genetic manipulation in myxobacteria is difficult owing to their slow and aggregative growth pattern, multicellular social behaviors, and resistance to the uptake of exogenous DNA^6^. The dearth of viable selection markers hinders the screening and purification of mutant strains. To date, the predominant genome editing technique for myxobacteria has been the inefficient and laborious process of RecA-dependent single or double homologous recombination (HR)^2,6^, which typically takes months to achieve traceless knockout of a gene via allelic exchange. Compared with other medicinal microorganisms such as *Streptomyces*, *Bacillus*, *Escherichia*, and *Aspergillus*, the progression of genetic tools for myxobacteria lags far behind. Thus, the development of efficient high-fidelity genetic toolkits is imperative to advance chemical and/or biological studies of myxobacteria.

Red/ET recombineering has gained considerable traction for genome editing because of its flexibility and high efficiency^14–16^. This technology was initially developed for editing DNA within *Escherichia coli* using either the bacteriophage lambda Red operon^17–19^ or the Rac prophage RecET system^14^. Each system is composed of an exonuclease, such as RecE or exo, which digests double-stranded DNA from the 5’ to 3’ direction, and an annealase, such as RecT or beta, which binds to the resulting 3’ single-stranded DNA overhang and facilitates its annealing to a complementary single-stranded DNA sequence^20^. This well-orchestrated partnership markedly increases the efficiency of homologous recombination and accordingly enables precise genome editing. Furthermore, the power of Red/ET recombineering is frequently amplified through synergistic integration with other advanced technologies. For instance, its pairing with CRISPR/Cas9-mediated double-strand breaks (DSBs) enhances the efficiency of targeted gene replacement^21–26^. However, our previous trial on the CRISPR/Cas9 system in DK1622 proved highly toxic and exhibited a high off-target rate^27^. In addition, the relatively large size of the Cas9 protein poses a significant delivery barrier in myxobacteria with underdeveloped genetic manipulation systems.

Transposon-associated transposase B (TnpB) represents a compact, RNA-guided nuclease system belonging to the IS200/IS605 transposon superfamily^28^. The system comprises the TnpB protein ( ~ 400 aa) and a transposon-encoded reRNA ( ~ 150 nt), which together form a functional ribonucleoprotein complex^29^. The reRNA comprises a 20-nt guide sequence, which base-pairs with the target DNA for programmable recognition, and a stem-loop structure, which binds and activates TnpB^30^. The TnpB–reRNA complex recognizes a transposon-associated motif (TAM, typically 5’-TTGAT-3’) situated directly beside the protospacer and then introduces a DSB precisely 3 nucleotides downstream of this sequence element^29,30^. Karvelis et al. innovatively applied the archetypal ISDra2 from *Deinococcus radiodurans* for programmable genome editing^29^. Feng et al. reported a TnpB that was sourced from a thermophilic archaeon and is employable in taxonomically distant bacteria such as *E. coli* and *Vibrio alginolyticus*^31^. Compared with that of Cas9 (~1600 aa), the compact architecture of TnpB ( ~ 400 aa) potentially mitigates the severe delivery barriers imposed by the cellular aggregation of myxobacteria^32^. Moreover, the high specificity of TnpB^29^ makes it more suitable for accurate editing within the large, GC-rich, repetitive genomes of myxobacteria^4^, where off-target cleavage poses significant risks. To our knowledge, no precedent exists for TnpB-mediated genome editing in myxobacteria. In particular, the combination of TnpB-mediated DNA cleavage with the robust Red/ET recombineering approach has not yet been reported.

In this study, systematic bioinformatic analyses reveal the high abundance and diversity of BGCs, highlighting the need for an efficient heterologous expression platform. To address this bottleneck, we identify an endogenous recombinase pair, MxRecET, that operates efficiently in *M. xanthus* DK1622. Furthermore, we pioneer a transformative approach by synergizing recombineering with TnpB-mediated DNA cleavage, enabling scarless genomic manipulations. This high-efficiency editing platform streamlines the construction of a chassis cell designated MxPKS, which is subsequently validated by the successful expression of different classes of PKS BGCs. This work establishes a solid foundation for accelerating myxobacterial natural product bioprospecting and advancing fundamental research in these genetically recalcitrant organisms.

## Results

### Compendium of BGCs in myxobacterial genomes

**Fig. 1:**
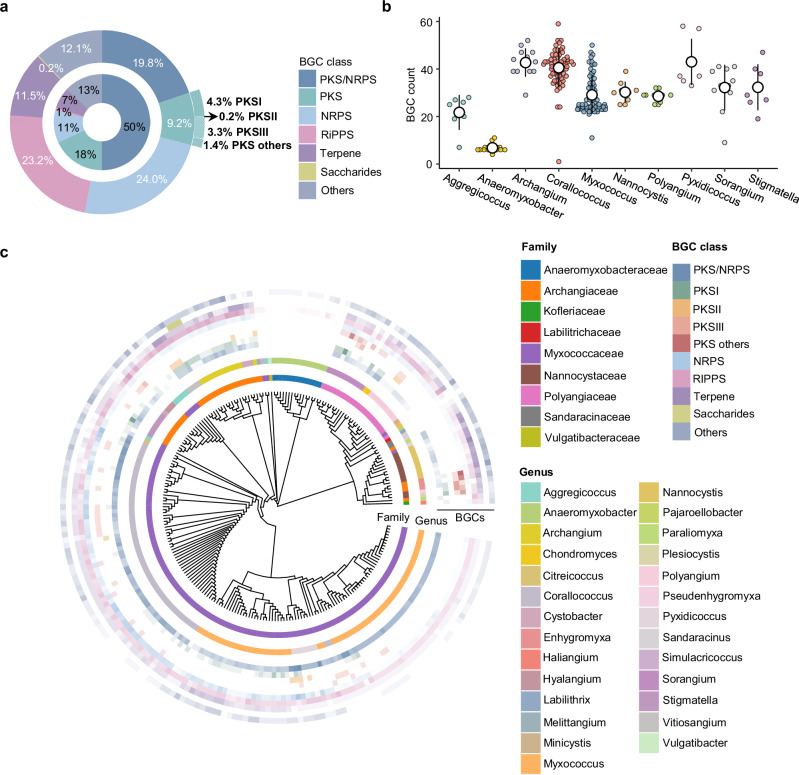
Global analysis of biosynthetic gene clusters distributed in the myxobacterial genomes. **a** A double-layered doughnut chart was constructed to provide an overview of BGC composition in myxobacteria. The outer layer represents the relative abundance of each BGC class, while the inner layer distinguishes the number of known myxobacterial NPs contributed by each class of BGC. The source data for the classification of BGCs and compounds refer to Supplementary Fig. 1 and the known compounds related to the MyxoDB database^2,33^, respectively. **b** Intergeneric variation in BGCs count plotted at the genus level. Each point represents an individual genome. The open circles represent the mean values of BGCs count, with error bars indicating standard deviation (SD) of the mean. Data are presented as the mean ± standard deviation of more than three independent samples. To ensure the accuracy of the mean test, only the top sequenced genera with sufficient statistical significance were included in this analysis. **c** Hierarchical clustering of 240 complete myxobacterial genomes. The phylogenetic tree was constructed using the UBCG pipeline with the maximum likelihood method. The classification of different families and genera is depicted in the first and second inner layer, respectively. The depth of the color in the 3^rd^ to the 11^th^ layers denotes the numbers of each class of BGCs. Source data are provided as a Source Data file.

A total of 240 complete myxobacterial genomes were downloaded from the NCBI RefSeq database and subjected to offline antiSMASH analysis, which revealed 7751 BGCs categorized into 10 different classes (Supplementary Data 1). Remarkably, 85.3% of all the BGCs lacked assignments to known compounds, indicating the vast unexplored biosynthetic potential of myxobacteria (Fig. 1a). Quantifying each BGC class per strain allowed us to determine the relative abundance of the BGC classes (Supplementary Fig. 1). Non-ribosomal peptide synthetases (NRPSs) are the most abundant BGC classes, accounting for 24% of total BGCs and contributing 11% of known compounds found in the MyxoDB database^2,33^. Ribosomally synthesized and post-translationally modified peptides (RiPPs) constitute the second highest proportion, up to 23.2% of total BGCs. Paradoxically, only five specific chemical scaffolds have been characterized to date^2,33^. Polyketide synthetases (PKSs) can be categorized into four main subtypes: type I, type II, type III, and other PKS variants. These BGCs represent a small fraction (9.2%) of the total BGCs but contribute a disproportionately large, although still moderate, share (18%) of known myxobacterial NPs. Although the type II PKSs are prevalent in Gram-positive bacteria such as actinomycetes, they are exceptionally rare in myxobacteria. The aromatic polyketides known as pyxidicyclines are the sole category of type II PKS–derived products from myxobacteria^9^. The modular hybrid NRPS/PKS biosynthetic machinery represents ~20% of the total BGCs in myxobacteria. However, these versatile systems are responsible for a considerably greater fraction—approximately 50% of all known myxobacterial NPs, including blockbuster scaffolds such as epothilone. Taken together, the statistical prevalence of unknown myxobacterial BGCs necessitates the development of a robust chassis.

We compared the average number of BGCs per genome at the genus level (Fig. 1b). The strains from *Archangium, Corallococcus*, and *Pyxidicoccus* harbor an average number of >42 BGCs per genome, supporting the importance of these genera and highlighting that they are worthy priorities for investigation. A phylogenetic tree based on 240 complete myxobacterial genomes, annotated at the family and genus levels, revealed the evolutionary relationships among these strains and their potential for secondary metabolite biosynthesis (Fig. 1c). The BGCs found within the families *Polyangiaceae*, *Archangiaceae*, and *Myxococcaceae* are substantially diverse, encompassing almost all known categories, whereas the family *Anaeromyxobacteraceae* has few BGC types. These results are aligned with the results of a pangenome study of myxobacteria by the Stevens group^3^. The prevalence of RiPPs is notable, with at least one such BGC present in each of the strains examined. NRPSs, hybrid NRPS/PKSs, and terpene BGCs are widely distributed across different families and genera. Saccharide BGCs are sparsely distributed in several genera, potentially conferring specific ecological advantages (Supplementary Fig. 2). The taxonomy-dependent distribution of BGCs in the realm of myxobacteria is consistent with the work by the Müller group, who demonstrated that the uniqueness of myxobacterial NPs is highly genus specific^34^. Collectively, the atlas delineated for the distribution of BGCs streamlines the prioritization of promising strains for genome mining of myxobacteria.

### Discovery and optimization of a recombineering system for *M. xanthus* DK1622

**Fig. 2:**
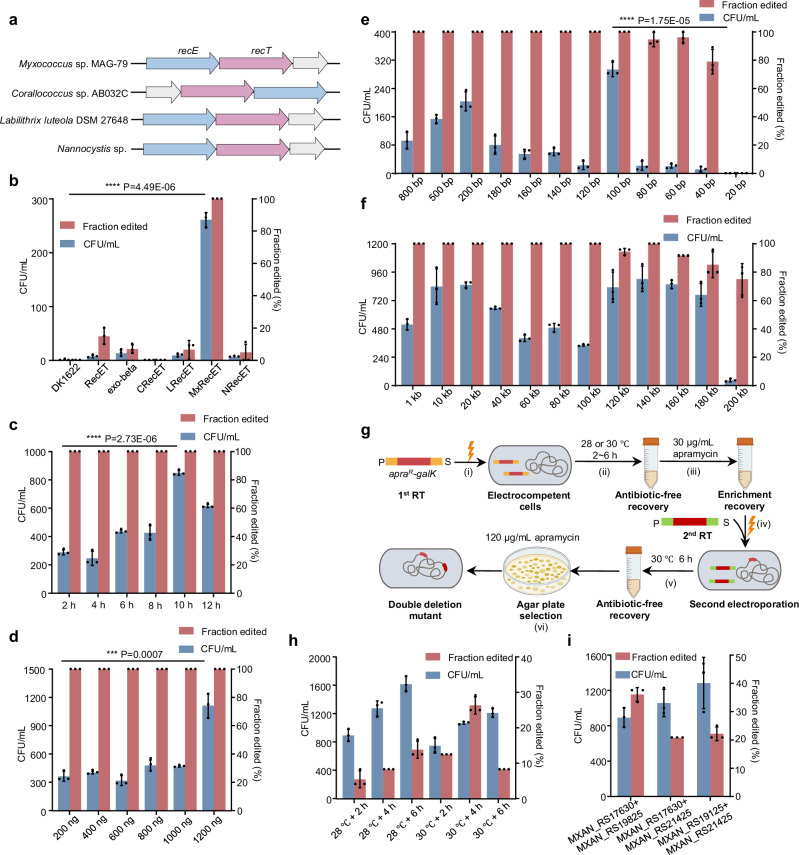
Development of MxRecET recombineering technology for *M. xanthus* DK1622. **a** Identification of *recET* orthologs in *Myxococcales*. Light blue arrows denote *recE* exonuclease homologs, magenta arrows indicate *recT* annealase alleles, and white arrows stand for hypothetical protein genes. The taxonomic origin of each *recET* operon is indicated. **b** Functional characterization of different RecET recombinases in *M. xanthus* DK1622. Recombination efficiency was reflected by total CFU counts and edited fraction per transformation.*E. coli* Rac prophage-encoded RecET and phage-derived λ Red (exo-beta) systems served as controls. **c**–**e** Effects of recovery time, exogenous donor DNA quantity, and homology arm length on MxRecET recombination efficiency. **f** Exploration on the upper limit of DNA fragment excision by the MxRecET system in DK1622. Data represent the *mch* locus; corresponding results for *ta* and *bgc18* loci are provided in Supplementary Fig. 4. **g** Schematic of the two-step electroporation strategy for dual-locus deletion: (i) first electroporation with an apramycin-resistance RT cassette. “P” and “S” stand for phosphorylated protection and phosphorothioated protection, respectively. (ii) recovery in antibiotic-free medium at 28 °C or 30 °C for 2–6 h. (iii) enrichment with 30 μg/mL apramycin. (iv) second electroporation using another apramycin-resistance cassette RT. (v) cells were recovered in antibiotic-free CTT medium at 30 °C for 6 h. (vi) final selection on solid medium containing 120 µg/mL apramycin. Illustrations created in BioRender. 1, 1. (2026) https://BioRender.com/l6mb85s. **h** Effects of recovery temperature and time on tandem editing efficiency. **i** MxRecET-mediated tandem editing efficiency across different dual-locus combinations. Data are mean ± SD, *n* = 3. All positive clones were verified by PCR and sequencing from 24 randomly selected colonies per group. Two-tailed Student’s t-test was used for comparisons between two groups; two-way ANOVA with Šidák’s test was used for multi-factor analysis. ***P < 0.001, ****P < 0.0001. Source data are provided as a Source Data file.

The vast untapped biosynthetic potential revealed by the systematic bioinformatics analysis underscored the pressing need to develop efficient genetic toolkits to construct an efficient plug-and-play DK1622-derived heterologous expression platform. While widely used recombineering systems such as λ Red are phage-derived, native recombinase pairs from *E. coli*^23^, *Burkholderia*^35^, and *Bacillus*^36^ have also demonstrated high efficiency in their respective hosts. We proposed that myxobacteria may also possess corresponding endogenous systems that could be harnessed for accurate genome editing. Therefore, we used the amino acid sequence of *E. coli* RecT protein^37^ as a probe to perform homologous alignment in the myxobacterial genomes. This practice led to eight endogenous recombinase operons in myxobacteria, four of which encompass alleles for both *recE* and *recT* (Fig. 2a). The theoretical sequence was substantialized via commercial DNA synthesis and placed under the control of the medium-strength constitutive promoter rrnD5^13^. The resulting constructs were separately integrated at the *attB1* locus of the *M. xanthus* DK1622 genome using Mx8 integrase^38^. The constitutive expression of RecET recombinases had little effect on cell fitness, as evaluated by the growth rate, plasmid transformation efficiency and mutation frequency (Supplementary Fig. 3). The recombineering was carried out by introducing a linear selection marker (apramycin-resistance gene, *apra*^*R*^) with flanking homology arms of 500 bp, whereby 5 kb of the *mch* BGC (encoding myxochromide) in RecET-bearing DK1622 was targeted. In parallel, 24 colonies from each case were subjected to PCR and DNA sequencing to verify the accuracy of the deletion. The *E. coli* Rac prophage-encoded RecET and the phage-derived λ Red (exo-beta) systems were employed as controls. Consequently, the RecET operon from a strain coded as *Myxococcus* sp. MAG-79 (hereafter denoted MxRecET) achieved the highest colony-forming units (CFU) per transformation and afforded nearly 100% editing accuracy under positive selection with 60 μg/mL apramycin (Fig. 2b).

To systematically optimize the MxRecET recombinase system, we selected three distinct genomic loci in DK1622 for 5-kb fragment deletion, including the *mch* BGC encoding myxochromide, the *ta* BGC encoding myxovirescin, and *bgc18* encoding an uncharacterized compound. These loci were chosen to evaluate key operational parameters, including post-electroporation recovery duration (Fig. 2c, Supplementary Fig. 4a, and Supplementary Fig. 4e), donor DNA quantity (Fig. 2d, Supplementary Fig. 4b, and Supplementary Fig. 4f), and homology arm length (Fig. 2e, Supplementary Fig. 4c, and Supplementary Fig. 4g). For each condition, we quantified CFU per transformation, and randomly selected 24 colonies for PCR validation and Sanger sequencing to determine editing efficiency. Notably, while CFU/mL varied substantially across different parameters, editing efficiency remained consistently near 100% under most tested conditions. Based on maximal CFU, we established optimal conditions of 10 h recovery time and 1200 ng exogenous donor DNA applicable across all three loci. Interestingly, the optimal homology arm length exhibited a consistent bimodal distribution, with peaks at short (100–140 bp) and intermediate (200–500 bp) lengths. We posit that shorter double-stranded homology arms (100–140 bp) are processed by MxRecE exonuclease to generate ~80–100 nt single-stranded overhangs, sufficient for MxRecT binding and efficient homologous sequence annealing^37^, whereas 200–500 bp arms support assembly of MxRecT into a fully functional nucleoprotein helical filament for catalyzing strand exchange reactions^39^. In contrast, homology arms shorter than 60 bp compromised editing efficiency, with complete loss of recombination observed below 20 bp. To further characterize the operational limits of the optimized system, we assessed the maximum size for deletion (Fig. 2f, Supplementary Fig. 4d, and Supplementary Fig. 4h). The system efficiently mediated deletions up to 180 kb, and even at 200 kb, maintained 60%–80% genetic fidelity at the *mch* and *ta* loci, despite a marked reduction in colony formation. Collectively, these findings demonstrate that editing efficiency of MxRecET recombinases is influenced by both operational parameters and genomic context of the target locus^23^.

We next sought to evaluate the feasibility of MxRecET for tandem editing of two arbitrary loci with a size of ~550 bp within the genes *MXAN_RS17630* and *MXAN_RS19125*, which are separated by a significant genomic distance of 3.8 Mb. Our initial approach involved a one-step co-electroporation of two distinct antibiotic-resistance cassettes flanked by ~100 bp homology arms. However, PCR screening of 96 colonies failed to identify a single clone with the desired double knockouts. We attributed this outcome to inefficient codelivery and the degradation of unprotected linear DNA. Instead, we developed a stepwise electroporation protocol employing phosphorothioated repair templates (RTs) to increase DNA stability. This strategy involved initial transformation followed by low-dose antibiotic selection (30 µg/mL apramycin) of single-recombinant intermediates prior to a second transformation targeting the second locus (Fig. 2g). We evaluated the effects of post-electroporation recovery temperature (28 or 30 °C) and duration (2–6 h) on dual-locus editing efficiency. Recovery at 30 °C for 4 h yielded a decent editing efficiency of ~27% (Fig. 2h). Under these optimized conditions, dual deletions targeting *MXAN_RS17630*, *MXAN_RS19125*, *MXAN_RS19825*, or *MXAN_RS21425* were achieved, with efficiencies consistently ranging from 20% to 40% (Fig. 2i).

The recycling of the *apra*^*R*^ marker is pivotal for the continuous genetic manipulation, which could be achieved by the site-specific Cre/loxP recombination (Supplementary Fig. 5)^40^. The positive selection marker *apra*^*R*^ and counterselectable marker *galK*^41^ were constructed into a linear dual-selectable marker flanked by *loxP* sites and homology arms to replace the myxovirescin cluster^42^ (Supplementary Fig. 6). Subsequently, Cre recombinase expressed from self-replicated vector pZJY4111^43^ mediated excision of the *apra*^*R*^-*galK* cassette, with a 100% marker removal accuracy (Supplementary Fig. 7). The Cre vector could be cured by a single passage on a nonselective agar plate.

### TnpB-assisted MxRecET recombineering for seamless editing

**Fig. 3:**
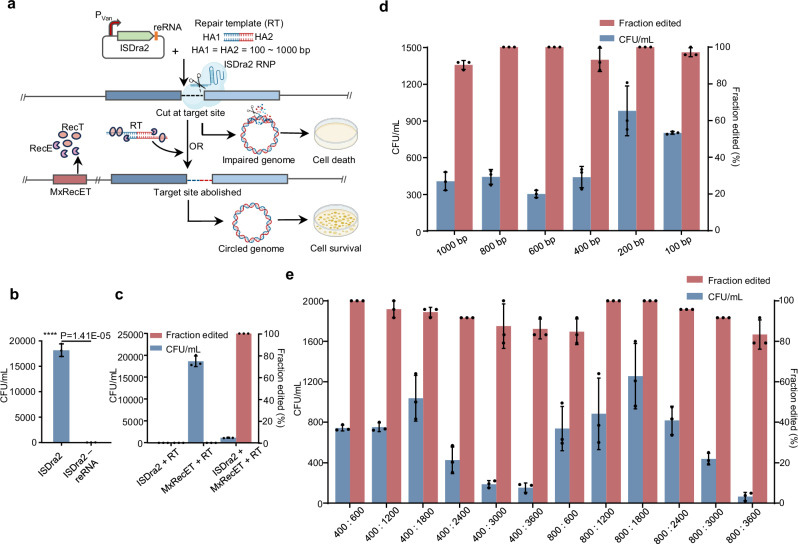
Development of the combined ISDra2-MxRecET technology. **a** Diagrammatic sketch for the combination of TnpB cleavage and MxRecET recombineering to enable facile scarless gene editing in DK1622. Illustrations created in BioRender. 1, 1. (2026) https://BioRender.com/elllytf. **b** Plasmid interference assay. The schematic diagram is present in Supplementary Fig. 8. The cleavage of *apra*^*R*^ by ISDra2-reRNA was manifested by the abolition of transformants. **c** Synergistic ISDra2–MxRecET enabled seamless editing without selectable marker. The transformation and editing efficiency were compared following (co)-electroporation of DK1622 with ISDra2 expression plasmid, MxRecET system, and/or a 200-bp homology repair template (RT). **d** The effect of homology arm length on the recombination efficiency of ISDra2-MxRecET technology. A continuous fragment ( ~ 0.5 kb) of the *mch* BGC was targeted for scarless deletion. **e** Optimization of the mass ratio between the ISDra2-expressing donor plasmid and repair template. Data are presented as the mean ± standard deviation of three independent biological replicates. Statistical significance was determined using a two-tailed Student’s t-test. All analyses were performed using GraphPad Prism 8.0, with *P* values indicated as follows: ****P < 0.0001. The corresponding results for *ta* and *bgc18* loci are provided in Supplementary Fig. 11. Source data are provided as a Source Data file.

The residual *loxP* scar introduced by Cre-mediated recombination can potentially compromise genomic stability following the iterative gene deletions required for chassis construction. Additionally, the sequential electroporation steps extend the timeframe of genetic manipulation. To overcome these challenges, we explored the integration of MxRecET recombineering with TnpB cleavage for continuous seamless gene knockout in DK1622. Briefly, TnpB induces targeted DSBs at a specific genomic locus, after which the MxRecET system uses a donor DNA fragment as a template for homology-directed repair and eliminates the original target cut site recognized by the TnpB/reRNA complex. By contrast, in the absence of MxRecET, TnpB-induced DSBs are either inefficiently repaired or lethal (Fig. 3a).

ISDra2 from *Deinococcus radiodurans* has been applied for programmable genome editing in *E. coli*^29^, human cells^44^, *Streptomyces*^45^, and plants^46^, which led us to surmise that it could also be workable in *M. xanthus* DK1622. This hypothesis was subsequently supported by the results of a plasmid interference assay (Supplementary Fig. 8). Briefly, ISDra2 alone or ISDra2 with an reRNA targeting *apra*^*R*^ was separately cloned into the kanamycin-resistant vector pSWU19^38^. The resulting transformants were challenged with a second plasmid pZJY4111, carrying an *apra*^*R*^ cassette, after which they were selected with apramycin (60 µg/mL). Colony formation was abrogated in the ISDra2–reRNA group but not in the ISDra2-only group, confirming the functionality of this exogenous ISDra2–reRNA system (Fig. 3b). This phenotype resulted from ISDra2–reRNA-mediated cleavage of the *apra*^*R*^ cassette rather than from the toxicity of ISDra2 itself, as evidenced by comparable growth curves between DK1622 expressing ISDra2–reRNA and wild-type DK1622 (Supplementary Fig. 9).

Subsequently, we explored ISDra2 as a counterselection method to promote precise MxRecET-mediated homologous recombination repair and to remove unedited cells from the population. A fragment ( ~ 0.5 kb) within the myxovirescin BGC was selected as a representative editing target, since it is nonessential for growth and contains a suitable transposon-associated motif (TAM) for scarless deletion (Supplementary Fig. 10). DK1622 cells with or without MxRecET were transformed with 400 ng of ISDra2-expressing plasmid and/or 1800 ng of 200-bp recombinogenic RT that was phosphorothioated at both termini. In the absence of MxRecET, the introduction of ISDra2 for DNA cleavage severely inhibited colony formation in DK1622 (Fig. 3c). The lethality of DSBs persisted even if RT was co-introduced. Conversely, co-transformation of the ISDra2 functional plasmid and a suitable RT into DK1622 cells that constitutively expressed the MxRecET recombinases resulted in robust colony growth. The combined ISDra2–MxRecET system achieved 100% accurate editing (24/24), whereas MxRecET alone produced no correct mutants among the same number of screened colonies (0/24). Therefore, the concurrent presence of ISDra2, MxRecET, and RT was essential for editing in absence of selectable marker. We next optimized key parameters of the combined ISDra2–MxRecET system, including homology arm length and the stoichiometry of the ISDra2-expressing plasmid to the RT, testing on the same three genomic loci (*mch*, *ta*, and *bgc18*) as employed for MxRecET alone. For the *mch* locus, scarless deletion efficiency remained consistently above 90% across all homology arm lengths tested, with higher CFU/mL value observed at 100–200 bp (Fig. 3d). This optimal range mirrored that of MxRecET alone, although the ISDra2–MxRecET system exhibited certain advantages in CFU/mL, potentially due to additional selective pressure from ISDra2-mediated DSBs. We further determined that an optimal mass ratio of 800 ng ISDra2 plasmid to 600–2400 ng RT maximized colony recovery (Fig. 3e). However, the efficiency of the ISDra2–MxRecET system also appeared to be influenced by genomic context. For instance, the optimal homology arm length for the *bgc18* locus was 800 bp, whereas the optimal plasmid-to-RT ratio was 800:600 ng for the *ta* locus (Supplementary Fig. 11). Nevertheless, all tested conditions for the three loci yielded decent CFU counts ( > 200) and consistently high editing efficiencies ( > 80%), confirming the robustness and generalizability of the ISDra2–MxRecET system. Hereafter, we designate this technology MxDIRECT (Myxobacterial Dual Integration of RecET recombineering and Cleavable TnpB). Notably, the workflow avoids the introduction and subsequent excision of selectable markers and thus expedites a clean mutation within a timeframe of approximately two weeks.

### Applying MxDIRECT for seamless deletion of large DNA fragments and single-nucleotide editing

**Fig. 4:**
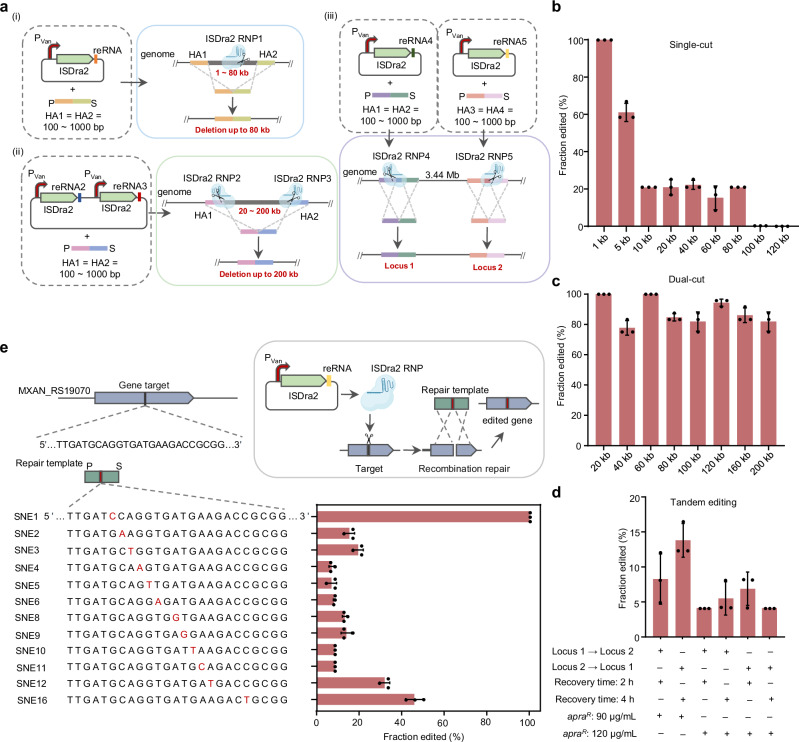
The MxDIRECT technology enabled flexible genome editing. **a** Diagrammatic sketch for facile scarless gene editing in DK1622 using the MxDIRECT technology: (i) a single-cut at the center of the target region by ISDra2, followed by MxRecET-mediated homology repair to delete up to 80 kb of DNA fragment; (ii) dual-cut at the two boundaries of the target fragment by ISDra2 increase the size limit to 200 kb; (iii) dual-cut at two distinct loci enables the tandem editing. **b** Size-dependent efficiency of seamless deletions using the single-cut strategy. The ISDra2-induced DSB was positioned near the center of gene *MXAN_RS17630* of myxochelin BGC. The bar chart displays the percentage for correct seamless deletions of 1–120 kb. **c** The dual-cut strategy enhanced the efficiency and size limit of large DNA deletions. The bar chart displays the percentage for correct seamless deletions of 20–200 kb. **d** Optimization of dual-locus seamless deletions based on two-step electroporation strategy. The schematic sketch for tandem seamless deletion can refer to Supplementary Fig. 13. The bar chart displays the efficiency of achieving correct dual knockouts. Locus 1 targets a 524 bp of *MXAN_RS19075* in the myxovirescin BGC; Locus 2 targets a 588 bp of *MXAN_RS32145* in the alkylpyrone BGC. The impact of recovery time following the first electroporation and the final concentration of the selection antibiotic on the editing efficiency was investigated. **e** Flexible single-nucleotide genome editing using MxDIRECT. Only the 20-nt sequences of the target region flanked by the TAM motif (TTGAT) and the variable RTs are shown. The mismatches between the target and recombinogenic RTs are indicated by bases in red. The editing efficiency of each RT is presented as bar charts. A total of 24 single colonies were selected randomly per condition to determine editing efficiency for (**b**–**e**). Data are presented as mean ± SD of three independent biological replicates. Illustrations created in BioRender. 1, 1. (https://BioRender.com/horxqeu). Source data are provided as a Source Data file.

Having established the optimized MxDIRECT protocol, we next investigated its upper limit for seamless deletions. Using a single-cut strategy (Fig. 4a, i), where an ISDra2-induced DSB was positioned near the center of the target region, we observed that the deletion efficiency decreased as the fragment size increased. While an efficiency of ~20% was maintained for deletions up to 10 kb, the practical upper limit for this approach was found to be ~80 kb (Fig. 4b). To overcome this size limitation, we hypothesized that inducing simultaneous DSBs at both flanks of the target region would increase efficiency, probably through two mechanisms: (i) increasing counterselection pressure, as the cell would need to repair two breaks to survive, and (ii) facilitating MxRecET-mediated recombination by exposing both homologous ends simultaneously, promoting efficient exonuclease processing and strand annealing. We engineered a single plasmid (on a pZJY4111 vector backbone) to co-express two distinct functional ISDra2 nucleases targeting both ends of the desired deletion fragment (Fig. 4a, ii). Compared with the single-cut strategy, the dual-cut strategy not only substantially increased the overall deletion efficiency but also dramatically extended the upper size limit (Fig. 4c). Most notably, the dual-cut strategy achieved more than 80% efficiency for seamless deletion of a 200 kb fragment, a feat unattainable with the single-cut approach.

We further adapted MxDIRECT to achieve seamless knockouts at two distinct genomic loci (Fig. 4a, iii). Initial attempts to employ a single plasmid co-expressing two distinct ISDra2 nucleases with two separate homology repair templates failed to yield the intended dual mutations. Screening of 96 colonies revealed that editing occurred exclusively at a single locus in all cases (Supplementary Fig. 12). We hypothesized that this outcome is likely attributable to the low frequency of simultaneously delivering three distinct DNA components into a single cell via electroporation. Therefore, we employed a two-step electroporation protocol by sequentially transforming cells with two component pairs, each comprising an ISDra2 cleavage plasmid and its corresponding repair template (Supplementary Fig. 13). Notably, 1 of 24 randomly selected colonies harbored the desired dual-knockout genotype. Optimization of this sequential protocol revealed the influence of deletion order, recovery time after the first electroporation, and final antibiotic concentration on the overall efficiency, yielding a maximum dual-knockout efficiency of 14% (Fig. 4d). Nevertheless, further optimization would be necessary to generalize this two-step electroporation protocol to broader experimental settings.

We also investigated whether the MxDIRECT technology is employable for homology-directed single-nucleotide editing (SNE). We constructed a plasmid-borne ISDra2 targeting the gene *MXAN_RS19070* within the myxovirescin BGC, wherein the target site was flanked by the TAM motif TTGAT. Twelve 200-bp mutagenic oligonucleotides were commercially synthesized as homologous repair templates, and each template contained a single nucleotide mutation across the 20-bp guide-target matching region. As a result, the efficiency of point mutations varied significantly depending on position and/or class. The mutation (G → C) at the nucleotide immediately adjacent to the TAM motif resulted in a gene efficiency of 100%, whereas the mutation efficiency at other positions fluctuated between 10% and 40% (Fig. 4e). Nonetheless, MxDIRECT still conferred all 12 possible classes of point mutations. To assess the influence of chromosomal context, we carried out SNE of *MXAN_RS17630* in the myxochelin BGC. Notably, the respective editing efficiencies at guide positions 1 and 8 from the TAM motif were 42% and 75% (Supplementary Fig. 14), which markedly contrasted with the 100% and 16% observed for *MXAN_RS19070*. We thus tentatively concluded that the SNE efficiency of MxDIRECT is governed by the interplay of mutation position, mutation class, and chromosomal microenvironment.

### Construction of the chassis cell MxPKS

Recently, we engineered a foundational chassis strain designated *M. xanthus* Hu04^47^ derived from wild-type DK1622, in which four key genes associated with multicellular social behaviors and two BGCs have been deleted (KO1–KO6 in Table 1). Compared with parental DK1622, Hu04 exhibited improved growth characteristics, particularly a more dispersed morphology in liquid culture and a compact single colony size on solid agar media (Supplementary Fig. 15), which are conducive to the fermentation and genetic manipulation of chassis cells for heterologous expression. Herein, MxDIRECT-mediated knockout of *imuB*^48^ associated with SOS mutagenesis in *M. xanthus* Hu04 accelerated the transition into the logarithmic phase, delayed the onset of the decline phase, and extended the stationary phase in the resultant strain KO7 (Supplementary Fig. 16). The superior phenotypic traits of KO7, such as abated cell–cell adhesion, convergent colonization, and a faster growth rate (Supplementary Fig. 17), are advantageous for the uptake of exogenous large complex BGCs by electroporation and the purification of mutant strains during genetic manipulation.

**Table 1:** The genotype of the chassis MxPKS compared with the parental strain DK1622

Mutants^a^	Region	Functions of the regions	Phenotypic improvements	Ref.	Size (bp)	Positions
Wild type	–	–	–	–	–	–
KO1	*aglZ*	Regulation of EPS biosynthesis and A-motility	Improve growth and genetic manipulation properties	^87^	4188	3,504,676-3,508,863
KO2	*pilA*	Regulation of EPS biosynthesis and S-motility	Improve growth and genetic manipulation properties	^88^	663	7,158,798-7,159,460
KO3	*difA*	Regulation of EPS biosynthesis	Improve growth and genetic manipulation properties	^89^	1386	8,235,892-8,237,277
KO4	*mazF*	type II toxin-antitoxin system PemK/MazF family toxin	Improve growth and genetic manipulation properties	^90^	369	1,962,661-1,963,029
KO7	*imuB*	Error-prone DNA polymerase associated with SOS mutagenesis	Improve growth and genetic manipulation properties	^48^	1512	4,858,117-4,859,628
KO5	BGC1(*car*)	Carotenoid biosynthesis (terpene synthase)	Eliminate competitive pathways and simplify metabolic background	^91^	7847	8,115,317-8,123,163
KO6	BGC17 (*dkx*)	Dkxanthene biosynthesis (hybrid PKS/NRPS)	Eliminate competitive pathways and simplify metabolic background	^92^	47,465	5,254,048-5,301,512
KO8	BGC15	Myxochromide biosynthesis (hybrid PKS/NRPS)	Eliminate competitive pathways and simplify metabolic background	^50^	4967	5,002,027-5,006,993
KO9	BGC13	Myxovirescin biosynthesis (hybrid PKS/NRPS)	Eliminate competitive pathways and simplify metabolic background	^42^	40,061	4,738,865-4,778,925
KO10	BGC19	Myxalamid biosynthesis (hybrid PKS/NRPS)	Eliminate competitive pathways and simplify metabolic background	^51^	28,796	5,610,658-5,639,453
KO11	BGC10	Myxochelin biosynthesis (siderophore NRPS)	Eliminate competitive pathways and simplify metabolic background	^52^	20,176	4,280,160-4,300,335
KO12	BGC24	Alkylpyrone biosynthesis (type III PKS)	Eliminate competitive pathways and simplify metabolic background	^53^	4434	8,167,029-8,171,462
IS13	EmrB- RND	EmrB multidrug efflux system	Enhance tolerance to toxic products	^54,55^	8966	4,177,428
IS14	NCM pathway	Non-canonical pathway for malonyl-CoA production	Augment the intracellular supply of vital precursor	^56^	3840	9,078,280

^a^ KO and IS mean knockout and insertion, respectively. Numbers indicate the sequential order of genetic manipulations from wild-type *M. xanthus* DK1622. KO1–KO6 represent knockouts completed in *M. xanthus* Hu04; KO7–KO12 were generated using MxDIRECT in this study; IS13 and IS14 indicate functional element insertions into the chassis strain.

Inspired by prior work by Sourice and coworkers^49^, we proceeded to delete endogenous BGCs in *M. xanthus* KO7 to reduce metabolic burden and eliminate competing pathways for precursors and energy. MxDIRECT was employed to sequentially delete the central biosynthetic genes of five (hybrid) PKS/NRPS BGCs (myxochromide^50^, myxovirescin^42^, myxalamid^51^, myxochelin^52^, and alkylpyrone^53^) while retaining precursor and post-modification genes (Table 1 and Supplementary Fig. 18). Compared with wild-type DK1622, the derivative strain KO12 exhibited a simplified HPLC profile, with notable improvement in the medium- to low-polarity regions (Supplementary Fig. 19). These regions are particularly important because they are where most heterologous products typically localize, facilitating target compound isolation and purification. Nevertheless, NMR-based metabolomic profiling^33^ of KO12 revealed the persistent accumulation of other compound classes, notably quinolines and cyclodipeptides (Supplementary Fig. 20). Identification of the underlying BGCs is currently under way to further simplify the metabolic background.

To increase the robustness of the chassis, we introduced exogenous resistance–nodulation–division (RND) and multidrug resistance B (EmrB) family efflux pumps^54,55^ into strain KO12 (Supplementary Fig. 21), enabling the export of diverse toxic metabolites that could otherwise lead to cellular stress and reduced cell viability. Specifically, the relevant resistance genes originating from our in-house strain *Vitiosangium* sp. SDU295, which contains a unique type II PKS gene cluster *vtc* (see next section), were PCR cloned under the control of the promoter J23104^13^. Compared with the KO12 strain, the resulting IS13 strain exhibited elevated minimum inhibitory concentrations (MICs) for diverse antibiotics with uncompromised cellular fitness, indicating an enhanced capacity to export toxic compounds. Given that the majority of myxobacterial NPs with prominent bioactivity are derived from PKSs and/or hybrid PKS/NRPSs^2^, we further introduced the artificial non-carboxylative malonyl-CoA (NCM) pathway^56^ to augment the intracellular pool of the critical precursor malonyl-CoA (Table 1). The malonyl-CoA level of IS14 was 52% higher than that of wild-type DK1622 (Supplementary Fig. 22). The strain IS14, designated MxPKS, represents a rationally engineered chassis cell in the field of myxobacterial NPs research.

### Heterologous expression of myxobacterial polyketides in MxPKS

**Fig. 5:**
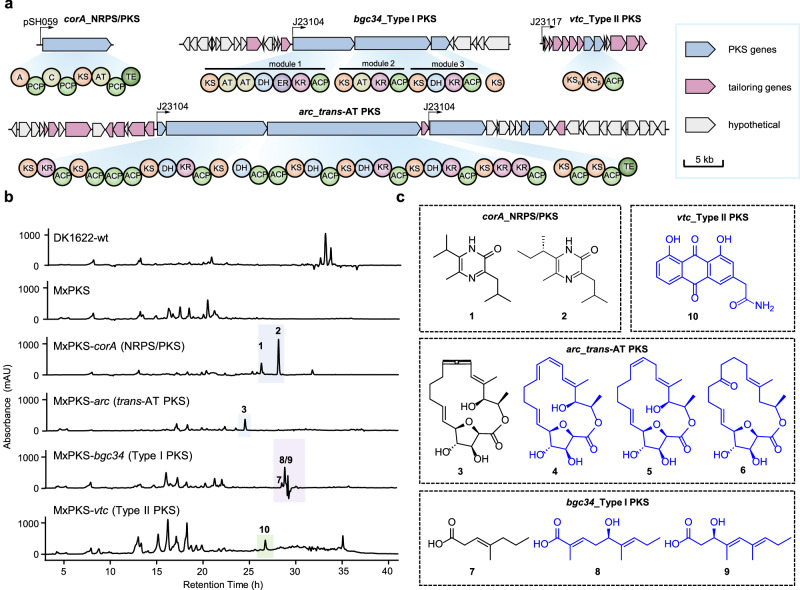
Heterologous expression of different classes of PKS BGCs in the chassis MxPKS. **a** Schematic diagram for gene arrangement of the BGCs coding for 5-methylated pyrazinones (*corA*), archangiumides (*arc*), branched fatty acids (*bgc34*), and anthraquinone (*vtc*), respectively. The module and domain arrangements of modular PKS and/or hybrid PKS/NRPS megasynthetases are specified. The positions for the insertion of the constitutive promoter(s) in each BGC are indicated by black arrows. The relative strength of each promoter has been previously characterized by our research group^13^. Domain abbreviations: KS ketoacyl synthase; AT acyltransferase; KR ketoreductase; DH dehydratase; ER enoylreductase; ACP acyl carrier protein; C condensation; A adenylation; PCP peptidyl carrier protein; TE Thioesterase. **b** HPLC-DAD comparison (detected at 254 nm) of MxPKS and DK1622 expressing *corA*, *arc*, *bgc34*, and *vtc*. The representative peaks for 5-methylated pyrazinones, archangiumides, branched fatty acids, and anthraquinone in MxPKS are color highlighted. **c** Structures of compounds **1**–**10**. Compounds **4**–**6** and **8**–**10** depicted in blue are previously undescribed structures. The structural determination of new compounds by 2D NMR analysis are provided in Supplementary Fig. 26, and chemical calculations of ECD spectra are in Supplementary Fig. 27. The detailed de novo structure elucidation and NMR data assignments are present in Supplementary Notes 1–6. Source data are provided as a Source Data file.

The efficacy of MxPKS was evaluated for the heterologous expression of myxobacterial PKS BGCs. We first selected the hybrid NRPS/PKS gene *corA* (~9 kb, Fig. 5a), which we recently demonstrated to autonomously encode 5-methylated pyrazinones (**1** and **2**) in *Corallococcus exiguus* SDU70^32^. The gene *corA* was cloned under the control of the potent promoter pSH059 in the plasmid pMiniHimar, and the resulting construct was expressed in the engineered MxPKS. Comparative HPLC–DAD analysis demonstrated that the production yield of coralinones in MxPKS reached ~2 mg/L (Fig. 5b).

Our group previously identified archangiumide (**3**) from *Archangium violaceum* SDU8, a first-in-class macrolide featuring a rare allene moiety^57,58^. Herein, we constructed a genomic library of SDU8, and subsequent PCR screening with primers specific to the *arc* BGC coding for archangiumide (Fig. 5a) yielded three overlapping fragments averaging ~40 kb each. These fragments were assembled in *E. coli* into the complete *arc* BGC ( ~ 60 kb) using ExoCET technology^59^. To ensure robust expression, the native promoters upstream of the core PKS genes, *arcA* and *arcC*, were replaced with the strong constitutive promoter J23104 via RedEx technology^60^. The cassette *oriT*–*attP*–*φC31* was added to the BAC expression vector to integrate into the genome of the MxPKS chromosome. Encouragingly, we observed the production of archangiumide (**3**) in MxPKS, as confirmed by HPLC‒DAD analysis (Fig. 5b). Scale-up fermentation (100 L) with the strain MxPKS-*arc* followed by systematic isolation led to **3** (10.2 mg) and three additional new analogues: **4** (0.5 mg), **5** (0.9 mg), and **6** (0.7 mg) (Fig. 5c). De novo structural elucidation (Supplementary Notes 1–3) revealed that these compounds arise from double-bond rearrangements or oxidation/reduction of the parental conjugated allene, thereby significantly expanding the chemical diversity of this structural family. In addition, the successful heterologous expression of archangiumide in MxPKS offers prospects for the systematic dissection of the enigmatic enzymology underlying allene formation—a long-standing puzzle.

Encouraged by our success in heterologous expression of known BGCs, we further harnessed MxPKS for cryptic myxobacterial PKS BGCs. With the help of the systematic biosynthetic summarization of myxobacteria shown in Fig. 1, the PKS BGCs that were not hybridized with NRPS were subjected to BigSCAPE/CORASON^61^ analysis (Supplementary Fig. 23). This directed our attention to a type I modular PKS gene cluster (*bgc34*, ~40 kb; Supplementary Table 1) from *Archangium violaceum* SDU8^57^. The three PKS megasynthetases encoded by the central biosynthetic genes feature unusual domain organization: i) module 1 contains two AT domains; ii) module 3 lacks an AT domain; and iii) the assembly line ends with a KS domain instead of a typical TE domain (Fig. 5a). Therefore, a cosmid containing intact *bgc34* was recovered from the SDU8 genomic library, and the promoter J23104 was then inserted in front of the first central PKS gene using RedEx technology^60^. The cassette *oriT*–*attP*–*φC31* was added into the BAC expression vector to integrate *bgc34* into the MxPKS chromosome. Notably, comparative HPLC‒DAD profiling demonstrated a peak present in the strain MxPKS*-bgc34* but completely absent in the control chassis (Fig. 5b). Chromatographic isolation of the *bgc34*-specified compounds from a 5 L culture led to the identification of three branched unsaturated fatty acids, including the known compound **7** (1.3 mg) and the previously undescribed compounds **8** (1.3 mg) and **9** (0.9 mg) (Fig. 5c). A detailed elucidation of the molecular structures of these compounds can be found in the supplementary data.

A type II PKS BGC termed *vtc* spanning 12.2 kb (Supplementary Table 2) in *Vitiosangium cumulatum* SDU295 attracted our particular interest because this class of biosynthetic machinery is notably scarce in Gram-negative bacteria. Prior to this work, a very similar case within myxobacteria was pyxidicyclines, a group of nitrogen-containing, tetracycline-like aromatic polyketides encoded by the type II PKS BGC *pcy* from *Pyxidicoccus fallax* An d48^9^. A comparison of *vtc* and *pcy* clearly corroborated a bona fide difference between these two BGCs (Supplementary Fig. 24). Further phylogenetic analysis of the two core KS genes in *vtc* using the web bioinformatic tool NaPDos2^62^ revealed that their closest matches are *Streptomyces*-derived KS genes coding for the antibiotic resistomycin, characterized by a unique discoid pentacyclic scaffold (Supplementary Fig. 25)^63^. The whole *vtc* was PCR cloned from the native host SDU295, and then integrated into the genome of MxPKS for heterologous expression. Subsequent HPLC–DAD profiling revealed that MxPKS carrying the promoter-engineered *vtc* BGC produced a new peak, exhibiting a characteristic UV absorption maximum at 430 nm. Scaled-up fermentation (30 L) of MxPKS-*vtc* followed by repeated chromatographic separation yielded **10** (1.5 mg). Intensive interpretation of 2D NMR (Supplementary Fig. 26) and high-resolution mass spectrometry data identified it as a new anthraquinone derivative (Fig. 5c). It structurally resembles the well-known compound emodin, which is widely found in various plants, fungi, and lichens^64^. Notably, during the isolation of **10**, additional minor peaks were observed. Although these were present in insufficient quantities for full structural elucidation, their UV absorption maxima at ~480 nm and 530 nm suggested that *vtc* may also orchestrate more extensively polycyclic frameworks in addition to tricyclic anthraquinones. Similarly, the *pcy* gene cluster from *Pyxidicoccus fallax* An d48 has been reported to encode both tricyclic anthraquinones and nitrogen-containing tetracene quinones^9^.

Taken together, these heterologous expression studies conclusively demonstrate that the engineered MxPKS chassis is a versatile platform for harnessing diverse PKS subtypes, including modular type I PKSs, hybrid PKS/NRPSs, and type II PKSs. The BGCs for the process can be sourced from phylogenetically diverse genera, including but not limited to *Myxococcus*, *Corallococcus, Archangium*, and *Pyxidicoccus*. Furthermore, the utility of the chassis extends to the successful production of a broad spectrum of structurally distinct chemical scaffolds, including macrolides, fatty acids, and aromatic polyketides. This robust capability underscores the potential of the engineered MxPKS system for broad applications in myxobacterial NP discovery and metabolic engineering.

## Discussion

Myxobacteria are a rich source of structurally diverse bioactive natural products, yet as revealed by our bioinformatic analysis, their vast reservoir of uncharacterized biosynthetic gene clusters underscores the need for efficient genetic tools to accelerate natural product discovery. Previously established genetic manipulation approaches, such as RecA-dependent homologous recombination, transposon mutagenesis, and heterologous expression in surrogate hosts, are constrained by low efficiency, poor adaptability, or long timelines^6,7^, while CRISPR/Cas9 systems exhibit pronounced toxicity and off-target effects in *M. xanthus* DK1622^27^. We developed the endogenous MxRecET system, which enables deletions of up to 200 kb with high fidelity, fundamentally overcoming the efficiency bottleneck. Beyond enabling heterologous expression chassis for natural product discovery, this robust platform holds great interest for the broader myxobacteria community devoted to fundamental research into myxobacterial biology or diverse biotechnological applications. For instance, this technology paves the way for engineering myxobacteria as living biocontrol agents by optimizing their predatory capacity against plant pathogens^65^ or as alternative chassis for the bioproduction of useful enzymes^66^. Nevertheless, limitations remain: while the moderate rrnD5 promoter^13^ achieves sufficient editing efficiency, constitutive recombinase expression may pose genomic instability risks in these repeat-rich genomes, suggesting a need for inducible regulation. Additionally, the efficacy of MxRecET may vary across phylogenetically distant lineages because of host-specific factors (codon usage, protein interaction networks, and DNA repair machinery). This limitation could be addressed by deploying alternative recombinases, such as the three additional RecET-like systems uncovered in this study, or by rational engineering of MxRecET to enhance cross-species functionality.

Building on MxRecET, we next sought to establish a programmable, scarless, marker-free editing paradigm avoiding Cas9-associated toxicity and off-target effects in *M. xanthus* DK1622. To this end, we developed MxDIRECT, which integrates Red/ET-based recombineering with TnpB nuclease cleavage. MxDIRECT offers three key advantages over MxRecET alone: (1) the elimination of antibiotic resistance markers avoids subsequent Cre/loxP-mediated removal; (2) the prevention of chromosomal scar formation mitigates the risks of undesired rearrangements; and (3) accelerated sequential multiplex editing via alternative TnpB plasmids substantially reduces the time and labor required for experiments. The underlying principle of MxDIRECT, which pairs an endogenous RecET-like system with a compact TnpB nuclease, may generalize beyond myxobacteria to serve as a framework for the vast majority of bacterial species. However, synchronous multisite editing remains challenging with MxDIRECT. This limitation likely stems from the inefficiency of co-delivering multiple DNA components into a single cell. Another possibility is that competition among multiple target sites for MxRecET dilutes enzymatic activity below the thresholds required for efficient recombination. This bottleneck could be circumvented through sequential electroporation strategies, which we have validated, while future efforts to enhance co-delivery efficiency and/or optimize component stoichiometry may enable true single-step multiplex editing.

Although MxDIRECT achieved all 12 possible classes of point mutations, the SNE efficiency varied significantly at different sites, primarily governed by two interrelated factors: (1) the position and type of the targeted mutation within the guide sequence modulate ISDra2 cleavage efficiency; (2) the local chromosomal microenvironment influences DNA accessibility and thus the exposure of the target site to editing machinery. While genome editing technologies, including those utilizing Cas9 or TnpB, offer transformative potential, their efficiency can be hampered by a spectrum of escape mutations^67,68^. Common escape mechanisms primarily involve small insertions ( ≤ 50 bp), IS element insertions, small deletions ( ≤ 50 bp), and point mutations^69^. Genomic analysis of *M. xanthus* DK1622 revealed a substantial burden of mobile genetic elements^6^. This high density of endogenous MGEs, particularly IS elements, may exacerbate escape mutations by providing readily available templates or targets for transposition, thereby competing with or disrupting intended homologous recombination events. Therefore, further efforts should be made to either suppress endogenous transposition or shield target sites from IS element integration. Charpentier and colleagues enhanced HDR efficiency by fusing Cas9 to the N-terminal domain of CtIP, a key homologous recombination initiation factor, thereby recruiting CtIP to DNA cleavage sites and significantly improving transgene integration^70^. More recently, Li et al. developed RED-CRISPR, featuring exo-beta fused with Cas9, which improved the editing efficiency and reduced off-target effects^71,72^. Building upon these principles, engineering a MxRecET–ISDra2 fusion protein that leverages the targeting ability of ISDra2 to directly deliver MxRecET to DNA cleavage sites could enhance the performance of MxDIRECT, for example, by expanding deletion size limits, improving single-base editing efficiency, enabling multiplex editing, and reducing escape mutations.

The MxDIRECT technology enabled the rational engineering of MxPKS, a chassis designed to express type I modular PKSs and PKS/NRPS hybrids, since these biosynthetic machineries produce most of the well-known myxobacterial natural products, including but not limited to high-value compounds such as epothilone and myxothiazol^2^. With this advanced tool, we have successfully addressed critical limitations of wild-type DK1622 by improving its growth characteristics, streamlining the metabolic background, and augmenting the precursor supply. However, the construction of a truly superior “plug-and-play” chassis is an ongoing endeavor being carried out with MxDIRECT: (1) The ability to perform scarless knockouts makes it feasible to eliminate autolysis-related genes implicated in programmed cell death^73,74^, thereby sustaining high-density cultivation for prolonged production phases^10^. (2) The precise deletion of restriction-modification systems^75^, key determinants of the natural barrier to exogenous DNA uptake, could be pursued without the accumulation of unwanted selection markers. (3) Morphological engineering^76^, such as disruption of cell‒cell adhesion pathways to generate hyperdispersed phenotypes amenable to high-throughput handling, also becomes readily achievable. (4) NRPS and RiPP BGCs represent comparably abundant yet underexplored reservoirs (24% and 23.2%, respectively), with their peptide products often exhibiting medium to high polarities that complicate metabolomic profiling. Our NMR-based analysis of MxPKS revealed persistent accumulation of quinolines and cyclodipeptides in this polarity range (Supplementary Fig. 20). Targeted identification of underlying BGCs followed by subsequent deletion represents a key strategy to further simplify the metabolic background. (5) Terpene BGCs constitute 11.5% of the pangenome, presenting a promising opportunity to engineer a dedicated terpene chassis by augmenting the isoprenoid precursor (FPP/GGPP) supply for functional characterization of the numerous unexplored terpene synthases revealed by our systematic bioinformatics analysis (Fig. 1).

Our bioinformatic analysis reveals compelling opportunities to develop additional, taxonomically specialized chassis systems. Notably, the genera *Archangium*, *Corallococcus*, *Pyxidicoccus*, and *Sorangium* exhibit exceptional biosynthetic capacity and/or proven track records for high-value bioactive compounds^2^. These phylogenetically distinct lineages likely possess unique precursor pools, post-modification enzymology, and regulatory architectures that could prove essential for activating silent or poorly expressed BGCs refractory to heterologous production in *M. xanthus*. However, extending MxDIRECT technology to these non-model myxobacteria presents substantial challenges, principally the efficient delivery of genetic cargo into strains with underdeveloped transformation protocols^6,7^. The compact size of TnpB ( ~ 400 aa) relative to that of Cas9 (~1600 aa) offers a critical advantage in this context, facilitating delivery via conjugation or engineered secretion systems where large payloads prove prohibitive. Successful implementation will require systematic optimization of electroporation parameters^77^ and/or development of conjugative delivery platforms^78–80^, wherein broad-host-range conjugation or repurposed Type IV/VI secretion systems could function as “molecular syringes” for direct cell-to-cell transfer of miniaturized genetic elements encoding MxRecET and/or TnpB machinery. Such expanded chassis diversity, coupled with streamlined delivery solutions, holds promise for unlocking the biosynthetic potential of non-model strains.

## Methods

### Systematic bioinformatics analysis of BGCs in myxobacterial genomes

A total of 240 completely sequenced myxobacterial genomes were retrieved from NCBI RefSeq database using the “datasets download” command-line tool. The antiSMASH 7.0^81^ was employed to mine all the genome sequences for the presence of putative natural-product BGCs. The annotations were performed using the default settings, incorporating the extended parameters of ClusterBlast, Cluster Pfam analysis, and Pfam-based Gene Ontology (GO) term annotation. The annotated BGCs were summarized for each strain (Supplementary Data 1). The phylogenetic tree of genomes was constructed using the GTDB-Tk 1.6.0^82^ and visualized via the “ggtree” R package^83^, with the characteristic values represented by the counts of BGC categories per genome. BGCs similarity network was constructed using BiGSCAPE 1.1.0^61^ tool, with the BGCs deposited the MIBiG repository 2.0^84^ as references. The analysis was performed under default settings, combining all classes and preserving singletons. Sequence similarities were calculated for raw distance cutoffs of 0.30–0.70, incremented by 0.05. Results for PKS types were visualized as a sequence similarity network using the “MetaNet” R package 0.2.5^85^ (https://github.com/Asa12138/MetaNet) at a cutoff of 0.65.

### Bacterial strains, plasmids, and growth conditions

The strains, plasmids and primers used in this study are provided in Supplementary Tables 3–5, respectively. *E. coli* strains were cultivated in Luria-Bertani (LB) medium (10 g/L tryptone, 5 g/L yeast extract, 5 g/L NaCl, pH 7.0). Myxobacterial strains were grown in CTT medium (10 g/L casein peptone, 1.97 g/L MgSO₄·7H₂O, 10 mL/L 1 M Tris-HCl, pH 7.6) or VY/2 medium (5 g/L baker’s yeast, 1 g/L CaCl₂, 0.5 g/L MgSO₄·7H₂O) with shaking at 30 °C, 200 rpm.

### Development of MxRecET recombineering for *M. xanthus* DK1622

*Bioinformatic analysis of endogenous RecET recombinase from myxobacteria:* The amino acid sequences of RecT from *E. coli* were used as query sequences for a PSI-BLAST search against the NCBI non-redundant (nr) protein database. The search was taxonomically restricted to the phylum *Myxococcota*, with cutoff criteria set at >60% query coverage and >30% sequence identity.

*Characterization of MxRecET recombineering*: The DNA sequences coding for putative RecET recombinases were then commercially synthesized and cloned into the pSWU19 vector^38^. The resulting vector was subsequently integrated into *attB1*_Mx8 site within the tandem tRNA^Asp^ gene *trnD1* of *M. xanthus* DK1622 genome via Mx8 site-specific recombination. A certain amount (200–1200 ng) of linear DNA cassette containing the selection marker flanked by different lengths (20–800 bp) of homology arms targeting a specific genomic locus (1–200 kb) was introduced via electroporation (1250 V, 400 Ω, 25 µF). After a period (2–12 h) of recovery in 1 mL antibiotic-free CTT liquid medium, resuscitated bacterial suspension was spread onto several CTT plates (15 cm × 15 cm) for accurate colony enumeration supplemented with 60 µg/mL apramycin. The total number of colonies from 1 mL bacterial suspension was reported as colony-forming units per milliliter (CFU/mL). To determine the precise recombination efficiency, resistant colonies were randomly selected from each transformation for genotypic verification. The successful replacement of the target genomic fragment by the *apra*^R^ cassette was confirmed by PCR screening and subsequent Sanger sequencing. The recombination efficiency was then expressed as the percentage of sequence-confirmed positive clones.

*Removal of antibiotic marker through Cre/loxP recombination*: The *loxP71-apraR-galK-loxP66* marker was excised from a verified clone by introducing the Cre-expressing plasmid pZJY4111^43^. Markerless mutants were isolated through dual selection with chloramphenicol (30 µg/mL) and 2-deoxy-galactose (DOG, 10 mg/mL). The final genomic modification, leaving a single 34-bp *loxP72* scar, was verified by PCR and sequencing. Finally, the Cre-expressing plasmid was cured by passaging the strain on antibiotic-free CTT agar for 5–7 days to yield the final markerless mutant.

### The MxDIRECT technology for genome editing

*Construction of ISDra2 cleavage plasmids:* The ISDra2 cleavage system was constructed using the self-replicating plasmid pZJY4111 with apramycin selection marker. The functional cassette included the *ISDra2* nuclease gene and 231 bp of reRNA, which was placed under the control of a vanillate-inducible promoter. For a given target site, a 20-bp guide sequence, located immediately downstream of a TnpB-associated motif (TAM) TTGAT was synthesized and ligated to the 3’ end of the reRNA scaffold. For dual-cut strategies, two distinct ISDra2–reRNA expression cassettes targeting different loci were incorporated into a single pZJY4111 plasmid.

*Preparation of repair templates:* The left and right homology arms (100–1000 bp length), flanking the target genomic locus, were individually amplified from DK1622 genomic DNA. The left arm and right arm were then seamlessly fused in a second round of overlapping PCR to generate the full-length RT. To protect the templates from intracellular nuclease degradation, this final product was re-amplified using primers terminally modified with phosphorothioate bonds.

One step co-transformation of *ISDra2 cleavage plasmid and repair templates: M. xanthus* DK1622 expressing MxRecET recombinases were co-transformed by electroporation with a mixture containing the ISDra2 cleavage plasmid and the corresponding linear repair template. Following electroporation, the cells were immediately recovered in antibiotic-free CTT liquid medium for an optimized period, typically ranging from 4 to 8 h. The recovered culture was then plated directly onto CTT agar containing 0.5 mM vanillate and 60 µg/mL apramycin. After growth of 5–7 days, all generated mutants were initially screened by colony PCR and subsequently confirmed by Sanger sequencing. This general protocol was adapted for single-locus seamless deletions or point mutations.

*Two-step transformation for dual-locus deletions:* Electrocompetent cells were transformed with the ISDra2 cleavage plasmid and homologous repair donor targeting Locus 1, recovered in antibiotic-free CTT medium at 30 °C for 2–4 h, and then enriched by incubating with apramycin (30 μg/mL) and vanillate (0.25 mM) for an additional 6–8 h. The resulting enriched culture was then subjected to a second transformation with the ISDra2 cleavage plasmid and homologous repair donor targeting Locus 2. Following a final recovery in antibiotic-free CTT medium for 6–8 h, the culture was plated onto solid CTT medium containing apramycin (90–120 μg/mL) and vanillate (0.5 mM) to select for dual-knockout mutants, which were subsequently verified by PCR.

### Construction of the chassis cell MxPKS

*Determination of malonyl-CoA level*: The malonyl-CoA content of DK1622 engineered strains was basically performed using a previously described method^56^. Cells were collected at the logarithmic growth phase by centrifugation at 10733 × *g* for 10 min at 4 °C. The cell pellet was resuspended in 2 mL ice‑cold PBS buffer, and cells were lysed by ultrasonication. The lysate was then centrifuged at 8000 g for 10 min at 4 °C. The standard curve for quantitative determination of intracellular malonyl-CoA was constructed by serially diluting malonyl-CoA standards, performing TMB-HRP colorimetric reaction via double-antibody sandwich ELISA kit (Fankew, Shanghai Kexing Trading Co., Ltd.). Absorbance at 450 nm was measured, and concentration (ng/L) was determined by linear regression. The OD values measured from experimental samples can be converted to the corresponding malonyl-CoA concentrations via the regression equation of standard curve.

*Determination of minimum inhibitory concentrations (MICs)*: The strains were cultured to the logarithmic growth phase, and the initial inoculum was adjusted to an OD_600_ of ~0.05. The suspension was then added to 96-well plates containing serially diluted antibiotics. After incubation at 30 °C for 36–48 h, the MIC was defined as the lowest antibiotic concentration that completely inhibited visible bacterial growth. All experiments were performed in biological and technical triplicates to ensure reproducibility.

### Heterologous expression of BGCs in MxPKS

*Construction of fosmid genomic library:* High-molecular-weight genomic DNA was randomly sheared to an average size of ~40 kb. The sheared DNA fragments were then subjected to end-repair and blunt-end ligation into the pCC1FOS vector. The resulting ligation mixture was packaged into lambda phage particles, which were subsequently used to transfect *E. coli* EPI300 competent cells. The transfected cells were plated on LB agar containing chloramphenicol to select for positive clones. The final library was titered, and individual clones were arrayed into 384-well plates for long-term storage and subsequent screening.

*Cloning of BGCs:* The NRPS/PKS gene *corA* and the Type II PKS *vtc* gene cluster were directly amplified from the genomic DNA of *Corallococcus exiguus* SDU70 and *Pyxidicoccus fallax* An d48, respectively. The forward primers were designed to append the strong constitutive promoters pSH059 and moderate constitutive promoter J23117 upstream of the *corA* and the *vtc* core biosynthetic genes, respectively. The large BGCs *bgc34* and *arc* were retrieved from the fosmid genomic library of *Archangium violaceum* SDU8. Fosmid clones harboring the target BGCs were identified through PCR screening with specific primers. Overlapping genomic fragments were released by restriction digestion and seamlessly assembled into the pBeloBAC11 vector using RecE/RecT-mediated Linear plus Linear Homologous Recombination (LLHR) in *E. coli* GBdir^59^. The pBeloBAC11 backbone was further engineered to include the *oriT-attP-φC31* cassette, which directs the random integration of the cloned BGCs into the MxPKS chromosome.

*RedEx-mediated promoter replacement*: To enhance gene expression, the native promoter of the target biosynthetic gene cluster (BGC) was replaced using RedEx recombineering^60^. Briefly, the BAC plasmid harboring the BGC was maintained in *E. coli* GBred-gyrA462, where exo-beta recombinase expression was induced with 10% L-arabinose. A linear cassette containing the strong constitutive promoter BBa_J23104^13^ and an *amp-ccdB* dual selection marker, flanked by 40-bp homology arms, was introduced by electroporation. Successful recombinants were selected on LB agar containing ampicillin (100 μg/mL). Subsequently, the *amp-ccdB* marker was precisely excised from the BAC by PacI digestion. The linearized plasmid was then transformed into the *ccdB* counter-selection strain *E. coli* GB2005 for re-circularization via Gibson assembly, yielding the final promoter-exchanged construct, which was verified by PCR and Sanger sequencing.

*Fermentation and metabolic analysis:* Engineered MxPKS strains harboring target BGCs were cultivated to generate seed cultures by inoculating mycelia into 50 mL of CTT liquid medium and shaking (200 rpm, 30 °C) for 3 days. For production, 10 mL of the seed culture was transferred into 100 mL of VY/2 production medium. The production cultures were incubated for 7 days (200 rpm, 30 °C). After fermentation, the culture supernatant was harvested and the metabolites were adsorbed onto 2 g of HP-20 resin. The resin was collected, dried, packed into a column, washed with H₂O, and subsequently eluted with methanol. The methanolic eluate was evaporated under vacuum to yield a crude extract. The crude extracts were analyzed by HPLC and LC-MS. HPLC analysis was performed on an Agilent 1260 system with a Phenomenex C_18_ column (4.6 × 250 mm, 5 μm). A linear gradient of methanol (solvent B, with 0.1% TFA) in water (solvent A, with 0.1% TFA) from 20% to 100% B over 50 min was used at a flow rate of 0.8 mL/min. Supporting spectroscopic data for all isolated compounds are provided in Supplementary Figs 28–72.

### Statistics & reproducibility

Sample sizes were determined by referring to published studies related to genome editing. No statistical method was used to predetermine sample size. All experiments were performed in biological triplicate, with replicate numbers stated in the figure legends. Data are presented as mean ± SD. Two‑tailed Student’s t‑test was used for two‑group comparisons, and two‑way ANOVA combined with Šidák’s test was used for multi‑factor analysis. No data were excluded from the analyses. The experiments were not randomized. The investigators were not blinded to allocation during experiments and outcome assessment. All myxobacterial strains were cultured under uniform conditions with parallel experimental setup.

### Reporting summary

Further information on research design is available in the Nature Portfolio Reporting Summary linked to this article.

## Supplementary information

Supplementary Information

Description of Additional Supplementary Files

Supplementary Data 1

Reporting Summary

Transparent Peer Review file

## Source data

Source Data

## Data Availability

The genome data used in this work are downloaded from NCBI RefSeq database (https://www.ncbi.nlm.nih.gov/refseq/), with details given in Supplementary Data 1. The processed spectroscopy data of the new compounds and Sanger sequencing data of all genome-edited strains in this study have been deposited in the figshare repository^86^ (10.6084/m9.figshare.31264693). All other experimental data, including annotated DNA sequences and numerical raw data, are compiled in the Source Data zip file provided with this article. Source data are provided with this paper.
