# Supplementary material for: Development of efficient genetic toolkits and heterologous chassis for bioprospecting of myxobacteria
Source: Nat Commun. 2026 Jun 29;17:8074. doi: 10.1038/s41467-026-74908-x (PMC13454183; doi:10.1038/s41467-026-74908-x)
Supplement: Supplementary file 1 [file 41467_2026_74908_MOESM1_ESM.pdf]

Supplementary information for the manuscript

Development of efficient genetic toolkits and heterologous chassis for bioprospecting of myxobacteria

Wen-Juan Zhang,[1, ⊥] Jia-Qi Hu,[1, ⊥] Rui-Juan Li,[1] Duo-Hong Sheng,[1] Xin-Jing Yue,[1] Qunxin She,[1] Youming Zhang,[1] Yue-Zhong Li,[1, *] Li Zhuo,[1, 2, *] and Changsheng Wu [1, 2, *]

[1] *State Key Laboratory of Microbial Technology, Institute of Microbial Technology, Shandong University, 266237 Qingdao, P.R. China*

[2] *Shenzhen Research Institute of Shandong University, 518057 Shenzhen, P.R. China*

* Corresponding authors emails: wuchangsheng@sdu.edu.cn (C.W.); zhuoli1992@sdu.edu.cn (L.Z.); lilab@sdu.edu.cn (Y.Z.L.)

⊥ These authors contributed equally

Table of Contents

Supplementary Figure 1. The number of each class of BGCs in the 240 myxobacterial genomes 4
Supplementary Figure 2. Heatmap showing the average abundance of different BGC classes within various myxobacterial genera 5
Supplementary Figure 3. The constitutive expression of RecET recombinases imposed little effects on cell fitness of DK1622........ 6
Supplementary Figure 4. Optimal parameters validation of MxRecET at the genomic loci *ta* and *bgc18*........ 7
Supplementary Figure 5. Diagrammatic sketch for the genome editing by MxRecET and Cre/loxP systems 8
Supplementary Figure 6. Verification of the recombination efficiency using the MxRecET system 9
Supplementary Figure 7. Verification of the efficiency for marker removal using Cre/loxP system.... 10
Supplementary Figure 8. Diagrammatic sketch for plasmid interference assay 11
Supplementary Figure 9. Comparison of the growth curve of DK1622-ISDra2-reRNA mutant and wild-type DK1622 11
Supplementary Figure 10. Schematic diagram of ISDra2-MxRecET mediated marker-free targeted knockout 12
Supplementary Figure 11. Optimal parameters validation of ISDra2-MxRecET system for scarless deletion at the genomic loci *ta* and *bgc18*. 12
Supplementary Figure 12. Diagrammatic sketch (a) and editing efficiency (b) of dual-cutting using a single plasmid co-expressing two distinct ISDra2 nucleases 13
Supplementary Figure 13. Diagrammatic sketch of the two-step electroporation protocol for MxDIRECT-mediated tandem editing........ 14
Supplementary Figure 14. Validation of MxDIRECT-mediated single-nucleotide editing (SNE) at an additional chromosomal locus 14
Supplementary Figure 15. The growth characteristics of the engineered strain *M. xanthus* Hu04 and the wild-type DK1622 in liquid culture (a) and solid agar media (b) 15
Supplementary Figure 16. Comparison of the growth curve of strain KO7 and with the wide-type DK1622 16
Supplementary Figure 17. Scanning electron microscope (SEM) observation of the strains KO7 and DK1622 16
Supplementary Figure 18. Diagrammatic summarization of the genome-reduced mutants of DK1622........17
Supplementary Figure 19. HPLC-DAD comparison of wild type DK1622 and the engineered strain KO12........ 18
Supplementary Figure 20. ^{1}H NMR-based metabolomic profiling of the engineered strain KO12...... 18
Supplementary Figure 21. Introduce of efflux pump system into the engineered strain IS13 19
Supplementary Figure 22. Introduction of the NCM pathway into engineered strain IS13 elevated malonyl-CoA levels 20
Supplementary Figure 23. Sequence similarity network (SSN) of myxobacterial PKS BGCs constructed by BiG-SCAPE tool........ 21
Supplementary Figure 24. Gene organization comparison of the type II BGCs *vtc* and *pcy*........ 22
Supplementary Figure 25. Phylogenetic analysis of the two core KS genes in *vtc* BGC 23
Supplementary Figure 26. 2D NMR correlation analysis of new compounds 24
Supplementary Figure 27. Experimental and calculated ECD spectra of compounds **8** and **9** 24
Supplementary Table 1. Gene annotations for *bgc34* in *Archangium gephyra* SDU8........ 25
Supplementary Table 2. Gene annotations for *vtc* in *Vitiosangium* sp. SDU295........ 26
Supplementary Table 3. List of strains used in this study 27
Supplementary Table 4. List of plasmids used in this study 28
Supplementary Table 5. List of primers used in this study........ 29
Supplementary Note 1. NMR data assignment of **4** in methanol-d_4........ 30
Supplementary Note 2. NMR data assignment of **5** in methanol-d_4........ 31

Supplementary Note 3. NMR data assignment of **6** in methanol-d_4 ... 32
Supplementary Note 4. NMR data assignment of **8** in methanol-d_4 ... 33
Supplementary Note 5. NMR data assignment of **9** in methanol-d_4 ... 34
Supplementary Note 6. NMR data assignment of **10** in DMSO-d_6 ... 35
SUPPORTING SPECTRA OF THE ISOLATED COMPOUNDS ... 36
Supplementary References ... 59

Supplementary Figure 1. The number of each class of BGCs in the 240 myxobacterial genomes. Source data are provided in the Source Data file.

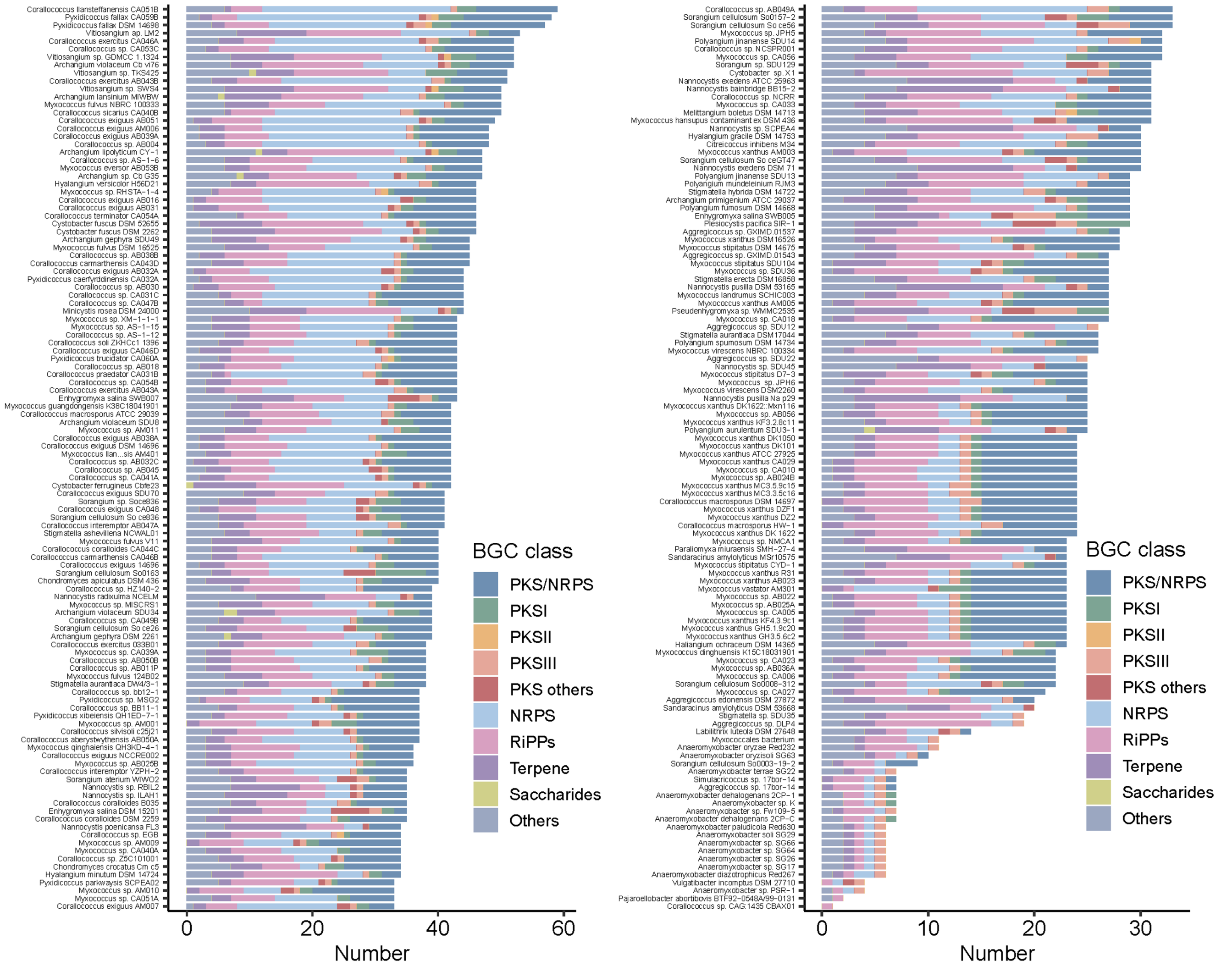

Supplementary Figure 2. Heatmap showing the average abundance of different BGC classes within various myxobacterial genera. The x-axis represents the genera, and the y-axis represents the BGC classes. Color intensity corresponds to the average number of each BGC class present in each genus, as indicated by the color scale. Source data are provided in the Source Data file.

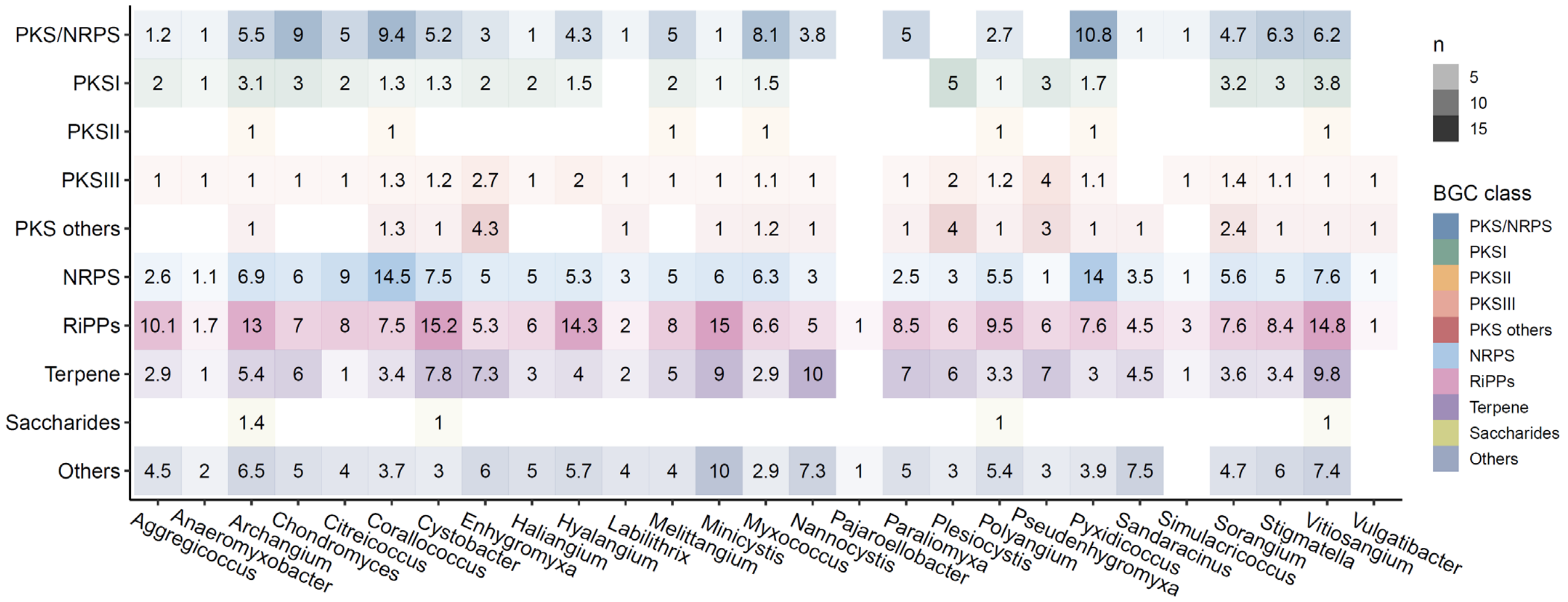

Supplementary Figure 3. The constitutive expression of RecET recombinases imposed little effects on cell fitness of DK1622. (**a**) Profiles of four RecET plasmids harboring moderate constitutive promoter rrnD5. (**b**) Schematic sketch for construction of RecET-integrated mutants derived from DK1622 via Mx8 site-specific recombination. *attP*_Mx8: attachment site on the donor plasmid; *attB*_Mx8: chromosomal attachment site; *attL* and *attR*: left and right attachment sites generated after site-specific recombination. (**c**) Growth curve analysis of DK1622 mutants containing different RecET recombinases. (**d** & **e**) Comparison of plasmids transformation efficiency of DK1622 wild type and DK1622-MxRecET mutant. Self-replicated plasmid pZJY4111 (**d**) and suicide plasmid pMiniHimar (**e**) were tested, respectively. There were no significant differences in the transformation efficiency of DK1622 and DK1622-MxRecET mutant. (**f**) Comparison of mutation frequency analysis of DK1622 wild type and DK1622-MxRecET mutant. The strains were plated on medium containing nalidixic acid (40 μg/mL), and spontaneous mutation frequencies were calculated by counting spontaneously resistant colonies, according to the method reported by Tzeng et al.[1] Values are means of the biological replicates, and the error bars indicate the standard deviations of three biological replicates. Source data are provided in the Source Data file.

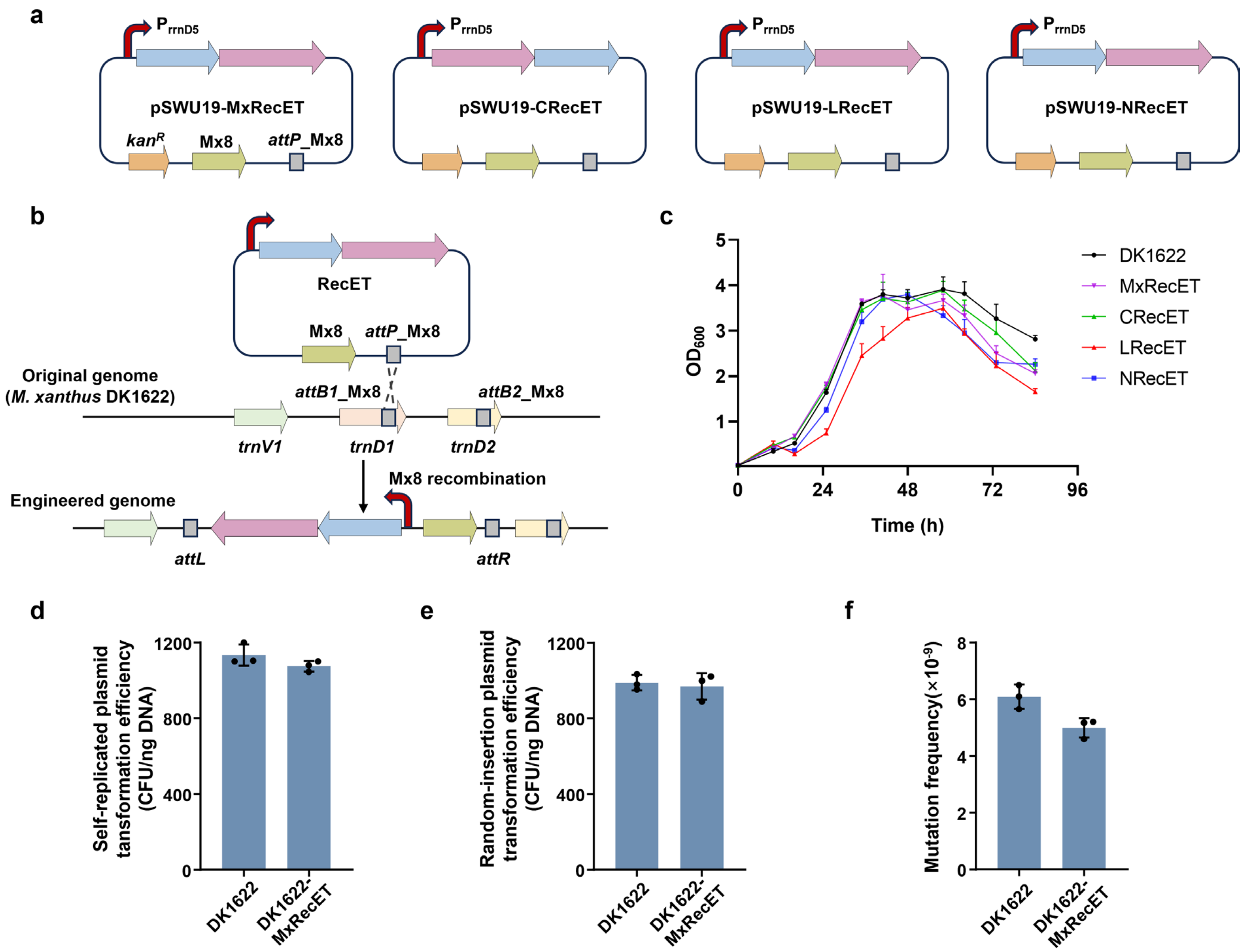

Supplementary Figure 4. Optimal parameters validation of MxRecET at the genomic loci *ta* and *bgc18*. The assessed parameters included post-electroporation recovery time (**a**, **e**), exogenous donor DNA dosage (**b**, **f**), homology arm length (**c**, **g**), and knockout fragment size (**d**, **h**). A total of 24 single colonies were selected randomly by PCR and Sanger sequencing to test the editing efficiency. Data are presented as mean ± SD, with n = 3 biological replicates. Statistical significance was analyzed by two-tailed Student's t-test using GraphPad Prism 8.0. P values for panel a, b, e and f are shown as follows: P(a)=9.85E-06 , P(b)=0.00065 , P(e)=0.0032 , P(f)=0.0001 .****P < 0.0001,***P < 0.001, **P < 0.01. Source data are provided in the Source Data file.

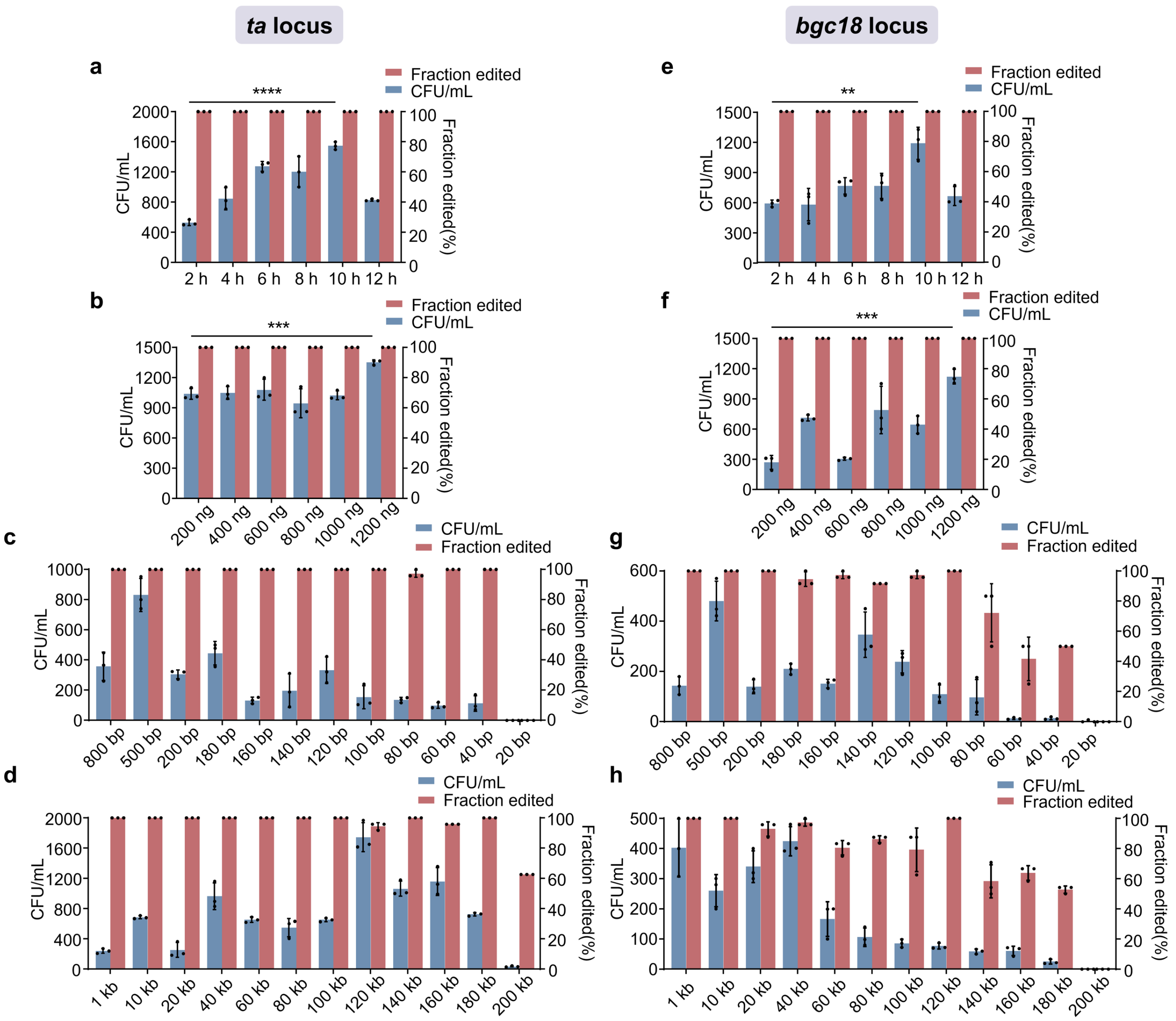

Supplementary Figure 5. Diagrammatic sketch for the genome editing by MxRecET and Cre/loxP systems. The architectural arrangement of the default selectable cassette *apra*^{R}-*galK* was bracketed by 100-bp variable homology arms HA1 and HA2. MxRecET system was integrated into the genome at the *attB1*_Mx8 site via Mx8 site-specific recombination; Then, the *apra*^{R}-*galK* cassette is electroporated into the MxRecET-containing cells to instigate the homologous recombination replacement of the targeted chromosomal DNA fragment; The Cre-expressing plasmid is introduced to excise dual selectable cassette to attain precise construction of markerless modification. A 34-bp scar *loxP* is left on the chromosome; Finally, a single passage on non-selective medium cures pZJY4111 plasmid, rendering the resulting clones ready for the next round of markerless genome editing. Illustrations created in BioRender. 1, 1. (2026) https://BioRender.com/viyamco.

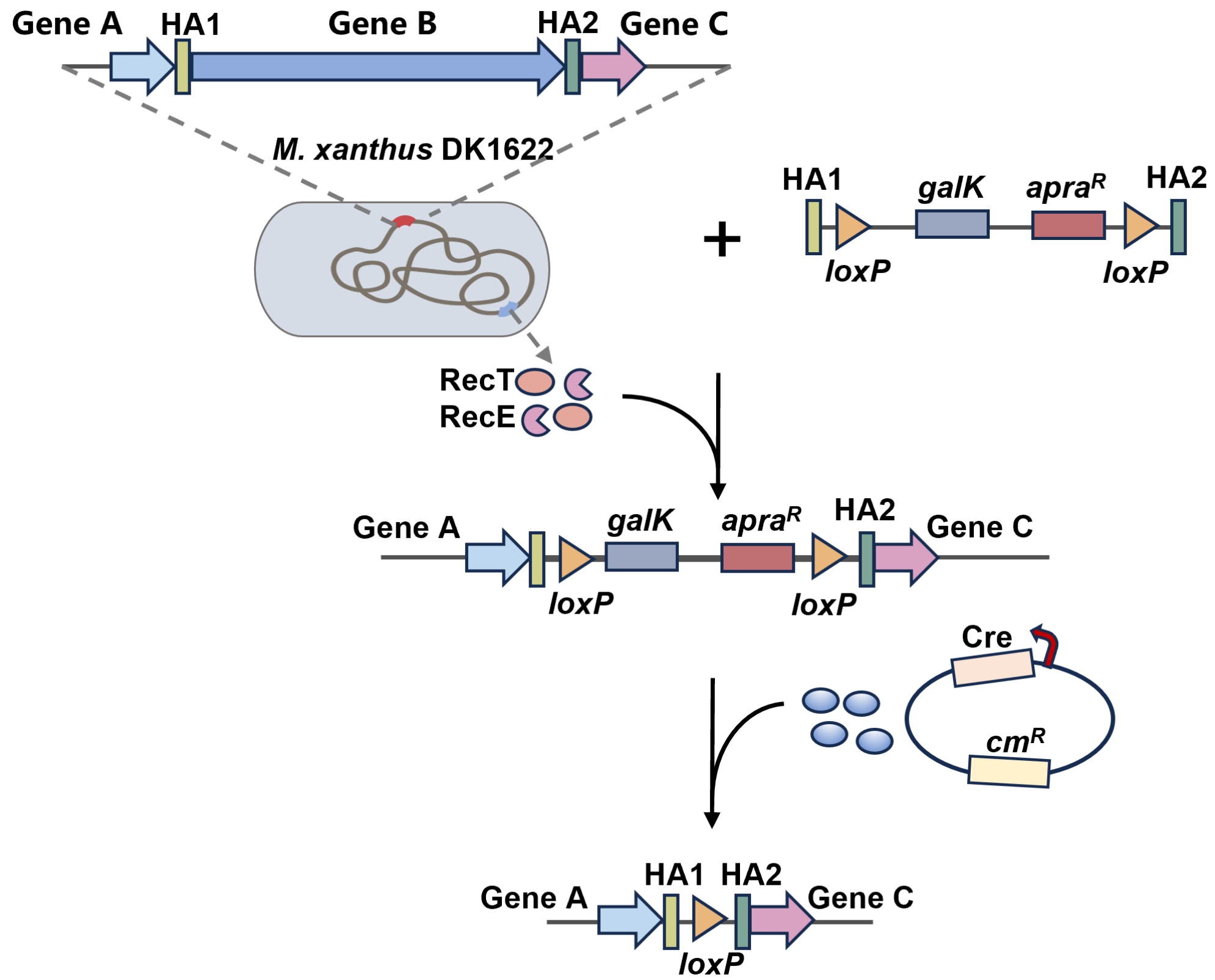

Supplementary Figure 6. Verification of the recombination efficiency using the MxRecET system. (**a**) Schematic of myxovirescin BGC replacement by a dual *apra*^{R}-*galK* marker. (**b**) PCR verification showed that all 16 single colonies were positive, as the amplified bands were 3425 bp, rather than the myxovirescin cluster (~40 kb). M: Trans 2K plus II Marker. (**c**) Further sequencing results indicated that the donor fragment (*loxP71-apra*^{R}*-galK-loxP66*) successfully replaced the target fragment harboring the complete *loxP71* and *loxP66* sites. Source data are provided in the Source Data file.

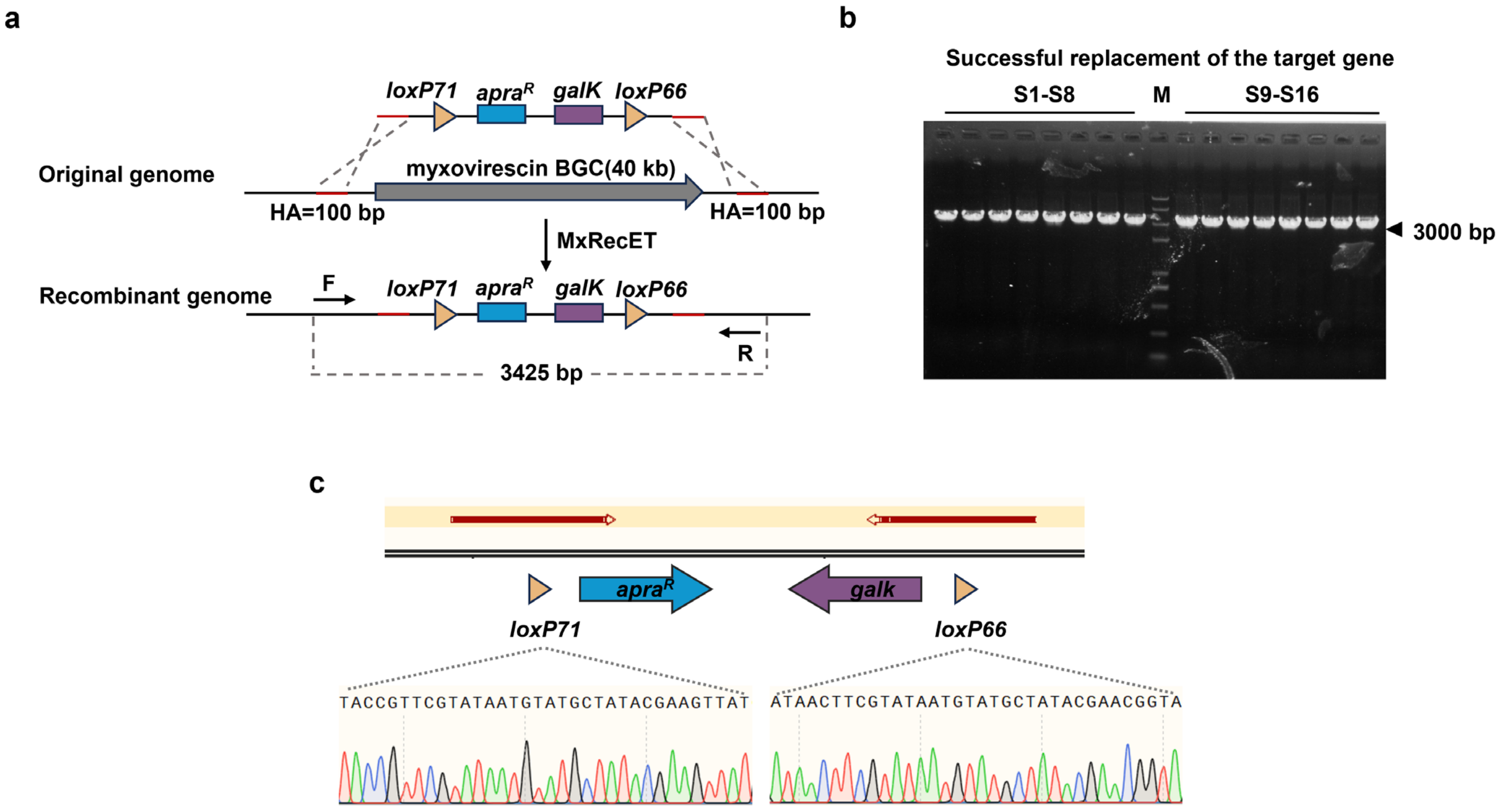

Supplementary Figure 7. Verification of the efficiency for marker removal using Cre/loxP system. (**a**) Schematic of the scar sequence left after the excision of dual-selection marker. (**b**) PCR verification of marker removal by Cre excision. Bands for the randomly selected 8 colonies gave the correct size (~760 bp). -: negative control, the region *loxP71-apra*^{R}*-galK-loxP66* was not removed (3425 bp). M: Trans 2K plus II Marker. (**c**) Sequencing result indicated that the region *loxP71-apra*^{R}*-galK-loxP66* was excised by the Cre recombinase, leaving a 34-bp of *loxP72* scar. Source data are provided in the Source Data file.

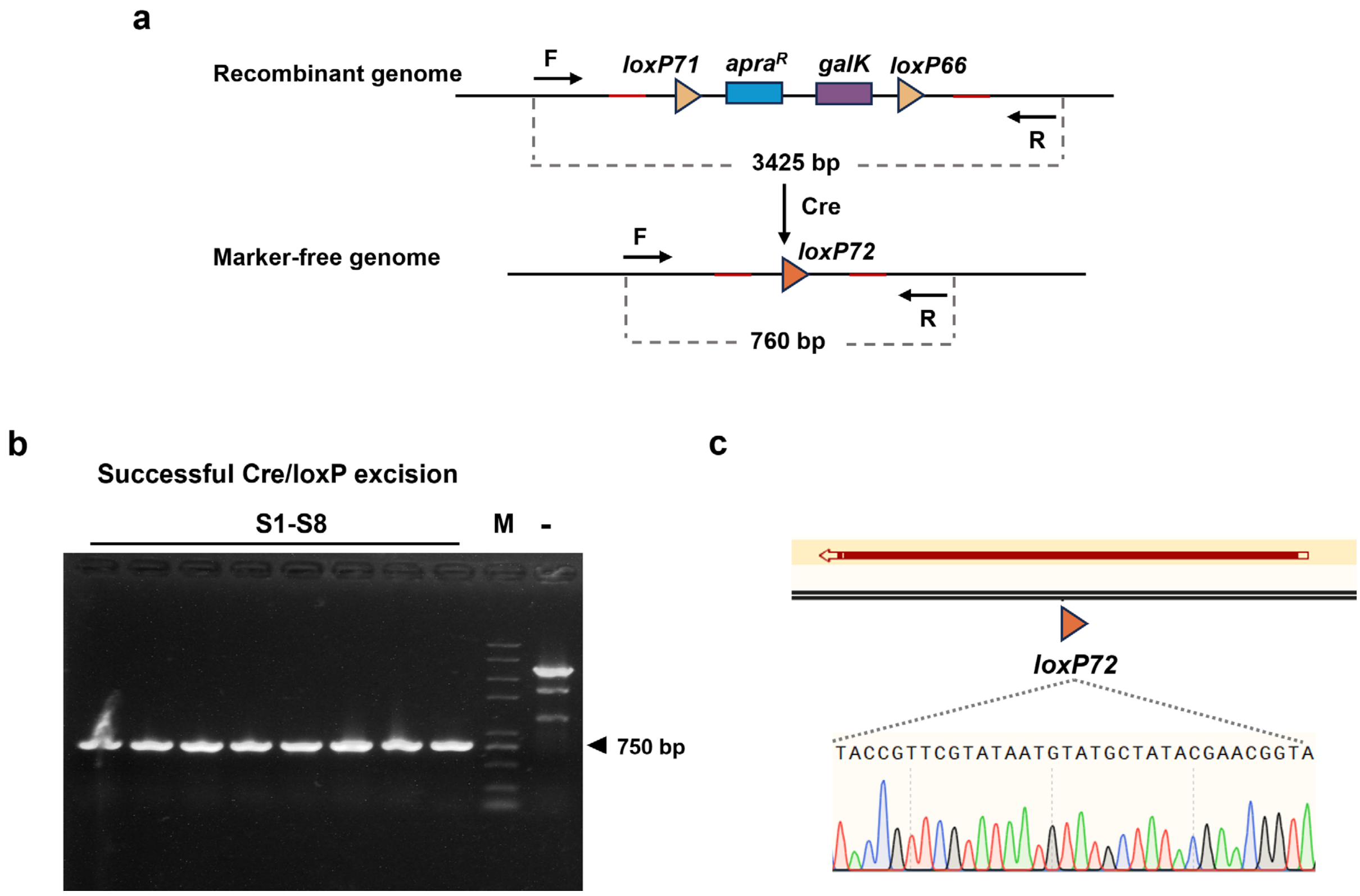

Supplementary Figure 8. Diagrammatic sketch for plasmid interference assay. The target of ISDra2-reRNA of the first plasmid pSWU19 is located in the apramycin-resistant cassette (*apra*^{R}) of the second plasmid pZJY4111. The functionality of ISDra2-reRNA was judged by the colony formation under the selection of apramyin (60 µg/mL). Illustrations created in BioRender. 1, 1. (2026) https://BioRender.com/viyamco.

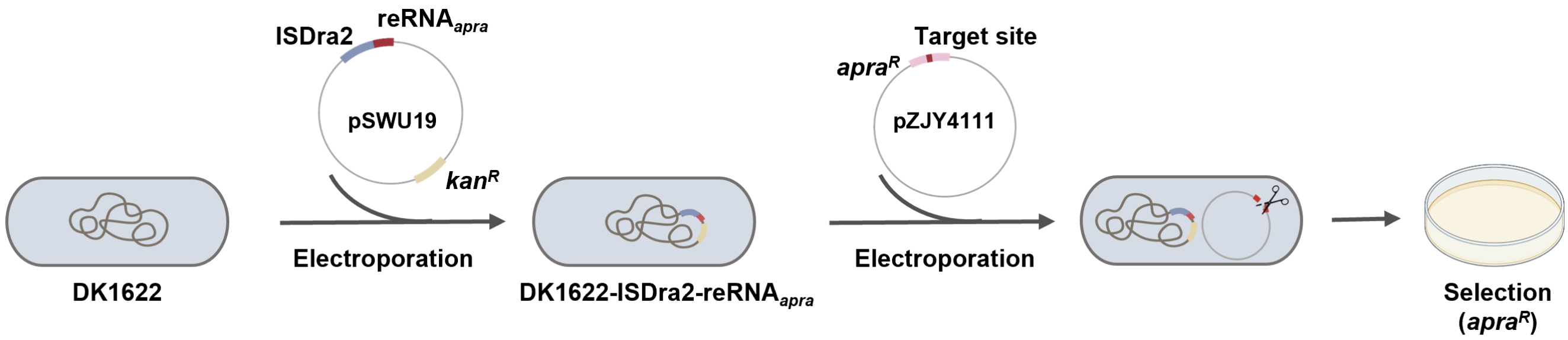

Supplementary Figure 9. Comparison of the growth curve of DK1622-ISDra2-reRNA mutant and wild-type DK1622. The expression of ISDra2 did not affect the growth of *M. xanthus* DK1622. Source data are provided in the Source Data file.

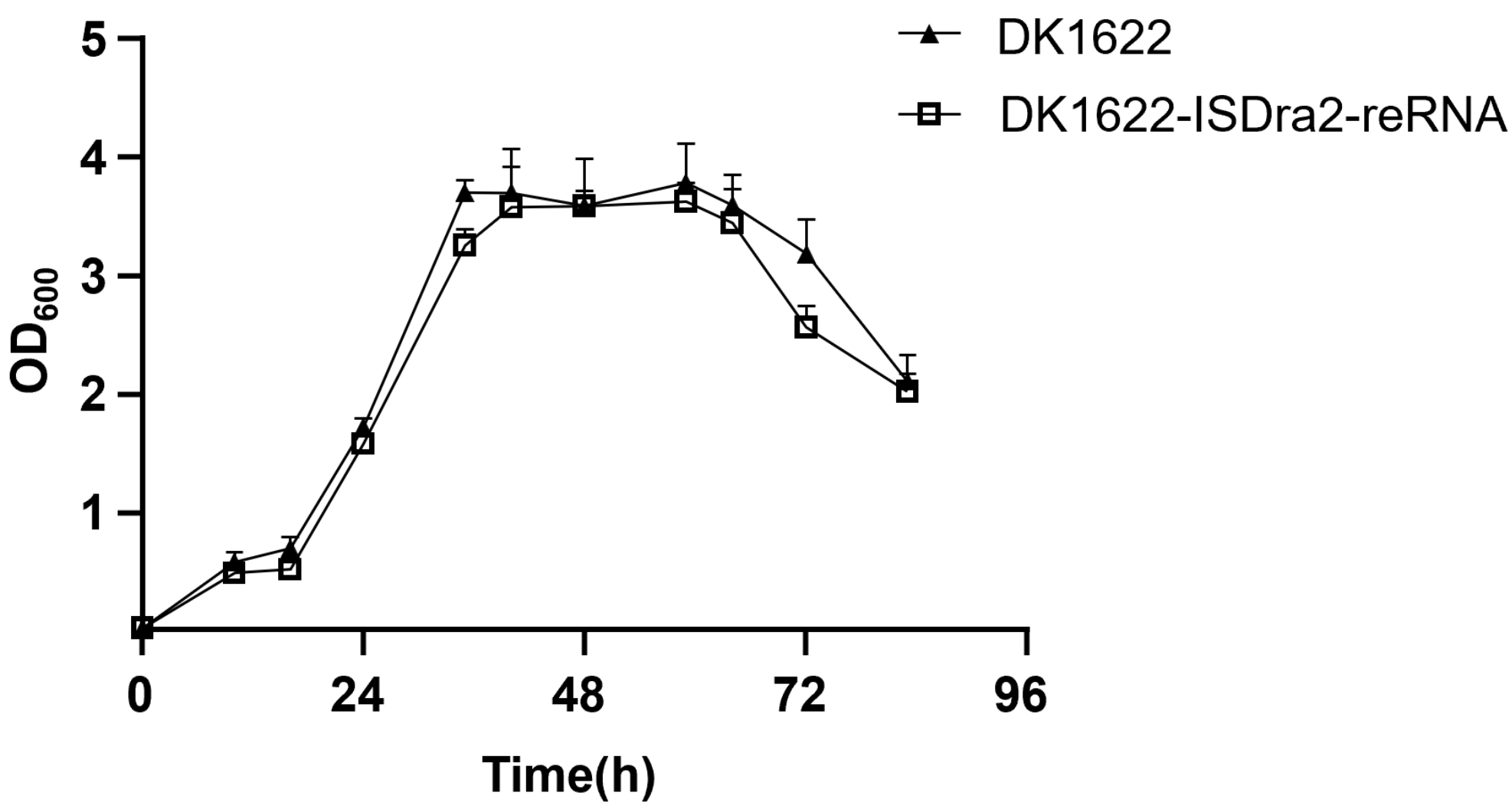

Supplementary Figure 10. Schematic diagram of ISDra2–MxRecET mediated marker-free targeted knockout. This strategy achieves the deletion of a 0.5 kb fragment in the myxovirescin gene cluster. Illustrations created in BioRender. 1, 1. (2026) https://BioRender.com/viyamco.

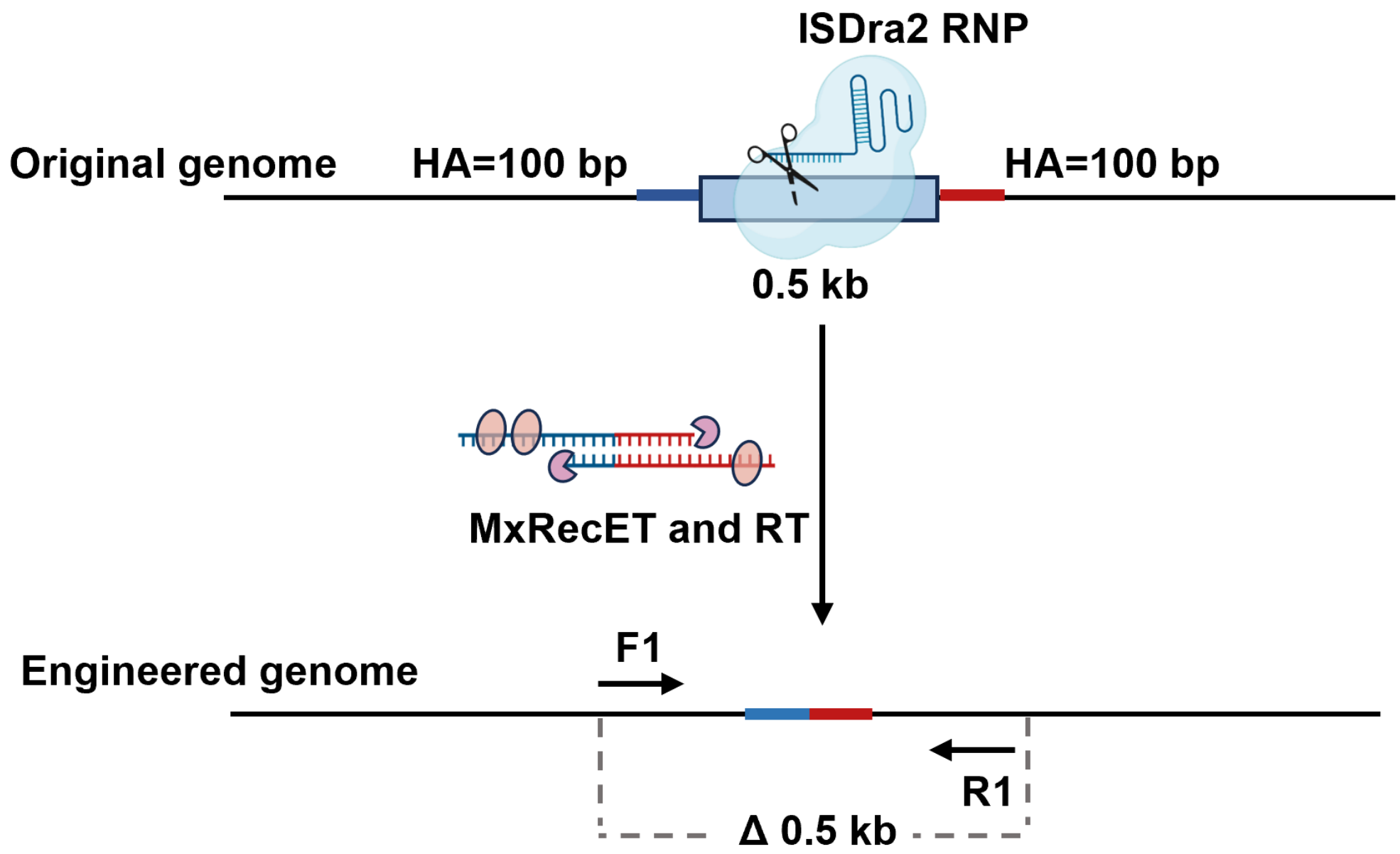

Supplementary Figure 11. Optimal parameters validation of ISDra2–MxRecET system for scarless deletion at the genomic loci *ta* and *bgc18*. The assessed parameters included homology arm length (**a**, **c**), and the plasmid and RT ratios (**b**, **d**). A total of 24 single colonies were selected randomly by PCR and Sanger sequencing to test the editing efficiency. Data are presented as mean ± SD, with n = 3 biological replicates. Source data are provided in the Source Data file.

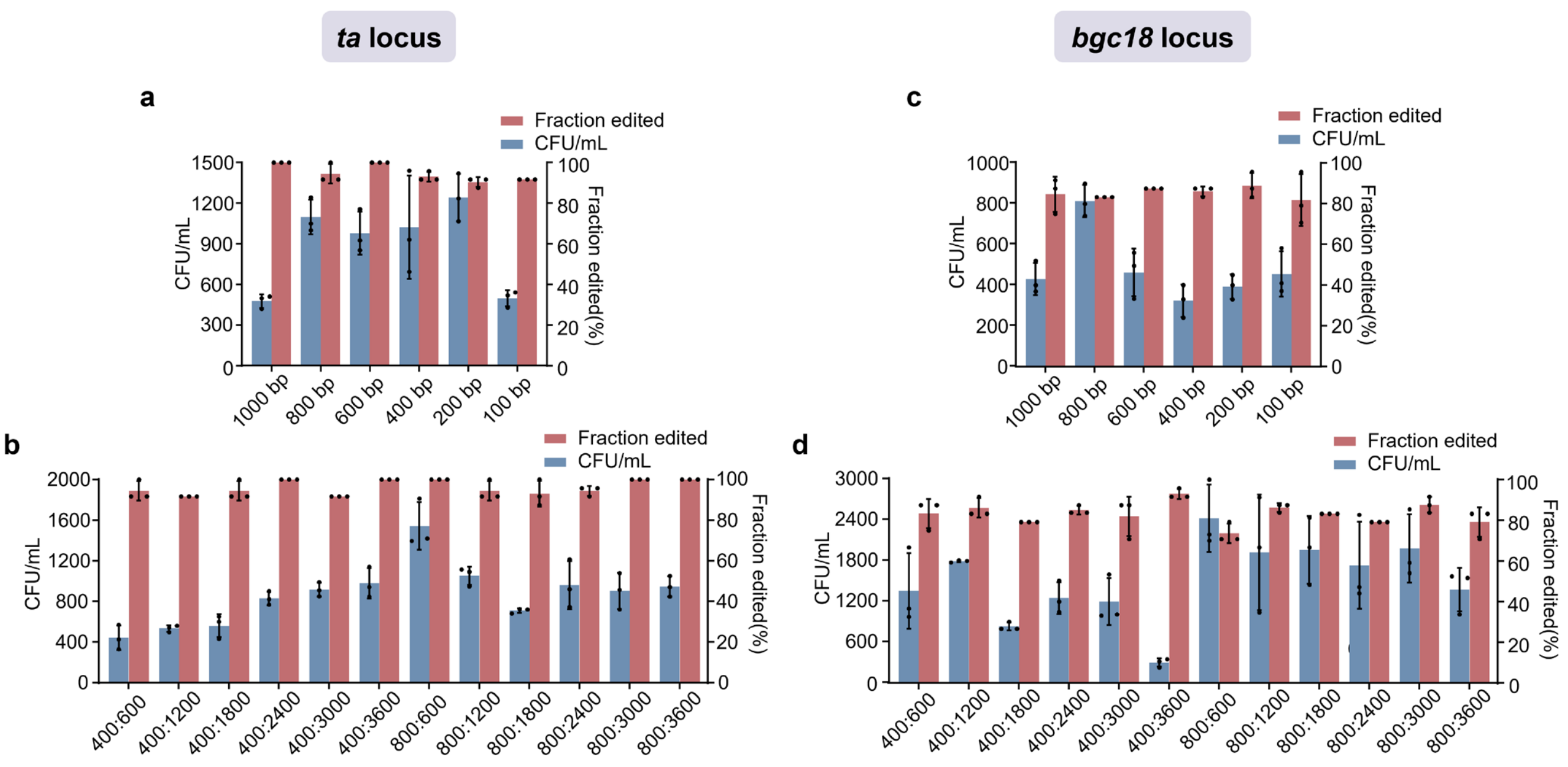

Supplementary Figure 12. Diagrammatic sketch (a) and editing efficiency (b) of dual-cutting using a single plasmid co-expressing two distinct ISDra2 nucleases. Locus 1 targets a 524 bp of *MXAN_RS19075* in the myxovirescin BGC; Locus 2 targets a 588 bp of *MXAN_RS32145* in the alkylpyrone BGC. Screening of 96 colonies showed exclusively 100% efficiency at Locus 2 while no editing at Locus 1. Illustrations created in BioRender. 1, 1. (2026) https://BioRender.com/viyamco. Source data are provided in the Source Data file.

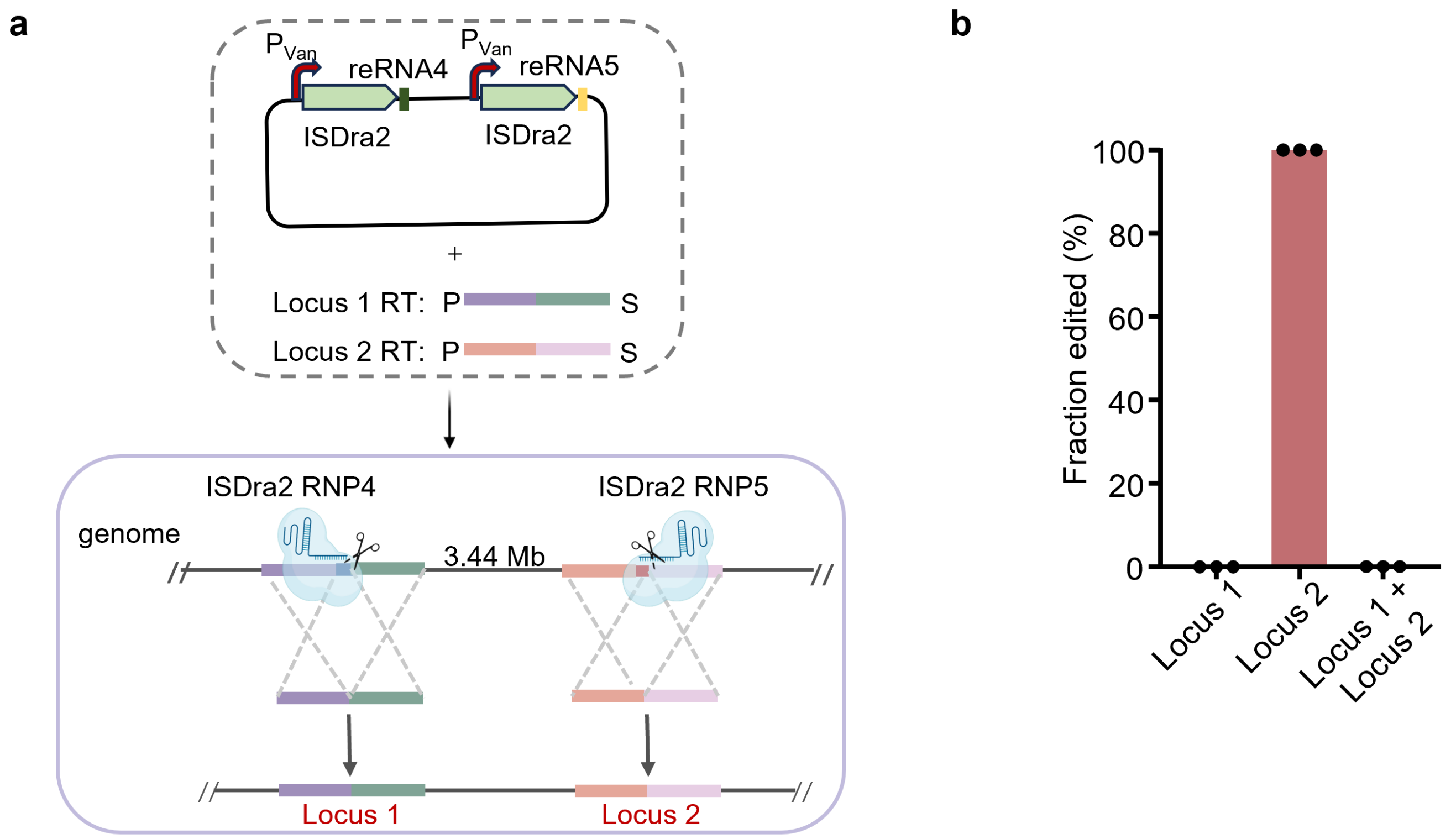

Supplementary Figure 13. Diagrammatic sketch of the two-step electroporation protocol for MxDIRECT-mediated tandem editing: (i) co-electroporation of the IsDra2 plasmid and the RT designed for locus 1; (ii) recover the cells in antibiotic-free CTT medium at 30 °C for 2~4 hours; (iii) add apramycin and vanillate to the recovered culture from the previous step to a final concentration of 30 µg/mL and 0.25 mM, respectively, and continue the incubation for another 6~8 hours; (iv) co-electroporation of the IsDra2 plasmid and the RT designed for Locus 2; (v) recover the cells in antibiotic-free CTT medium at 30 °C for 6~8 hours. (vi) screen the cells using plates containing apramycin (90–120 µg/mL) and vanillate (0.5 mM). Illustrations created in BioRender. 1, 1. (2026) https://BioRender.com/viyamco.

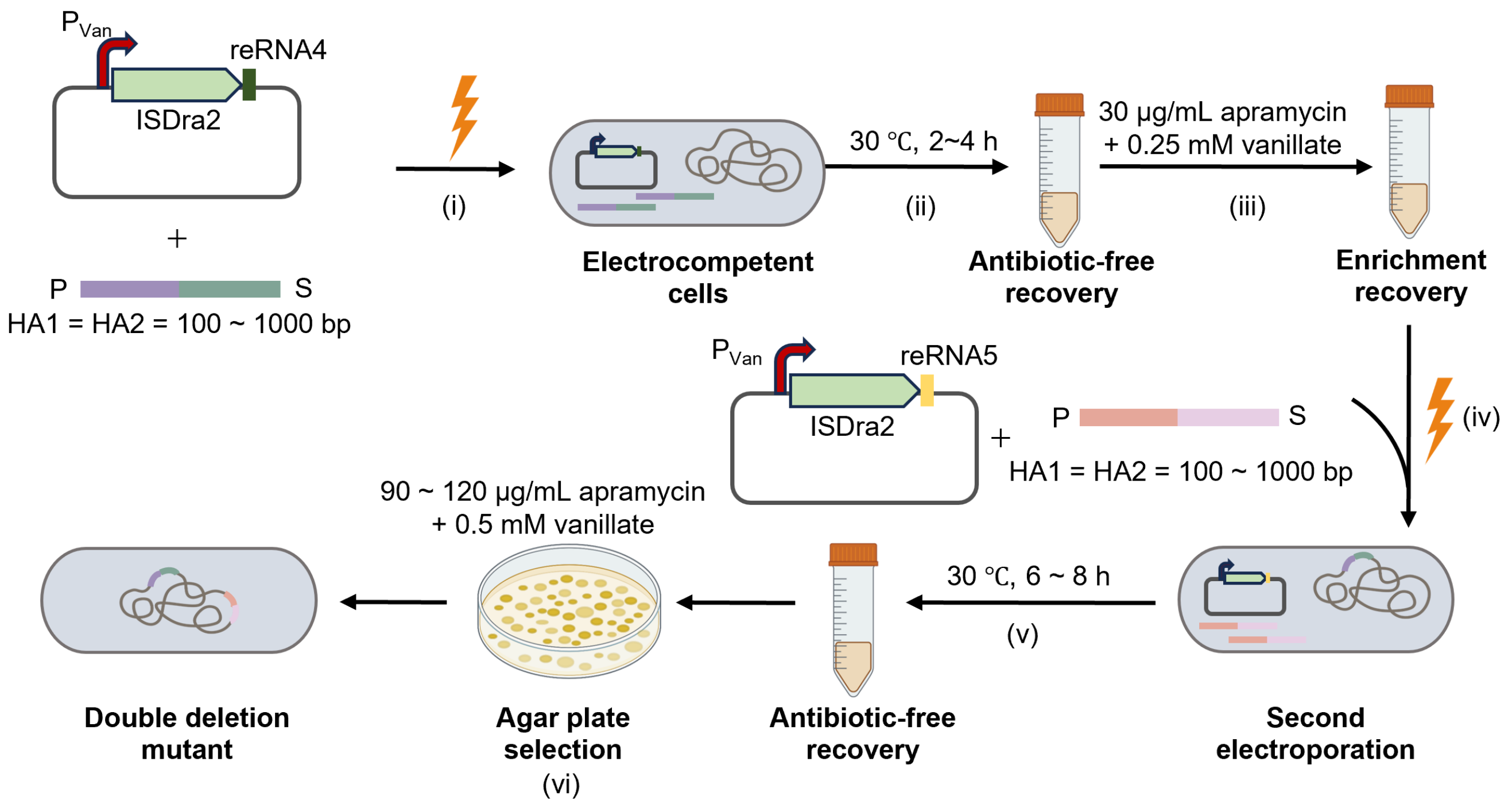

Supplementary Figure 14. Validation of MxDIRECT-mediated single-nucleotide editing (SNE) at an additional chromosomal locus. (**a**) Mutation positions and loci information. The targeting gene is *MXAN_RS17630* within myxochelin BGC, with SNEs introduced at position 1 and 8 of the guide sequence, as highlighted by red color. (**b**) Editing efficiency analysis. A total of 24 single colonies were selected randomly by PCR and Sanger sequencing. Source data are provided in the Source Data file.

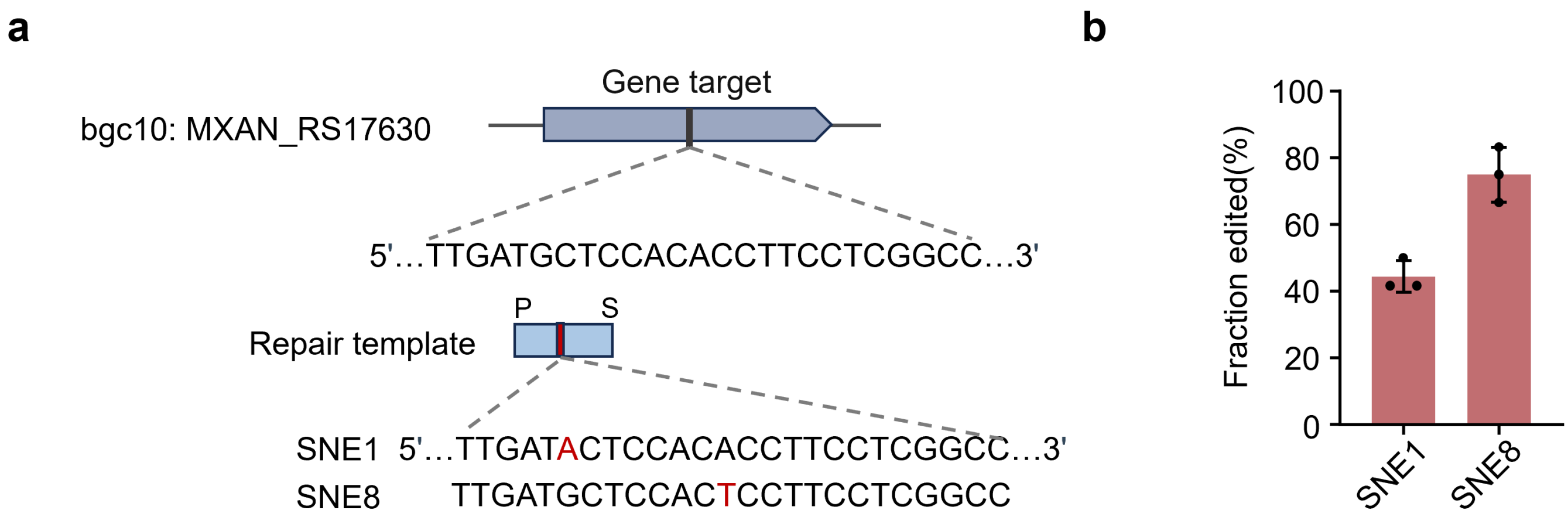

Supplementary Figure 15. The growth characteristics of the engineered strain *M. xanthus* Hu04 and the wild-type DK1622 in liquid culture (a) and solid agar media (b). The red bars represent 1000 px. Illustrations created in BioRender. 1, 1. (2026) https://BioRender.com/viyamco.

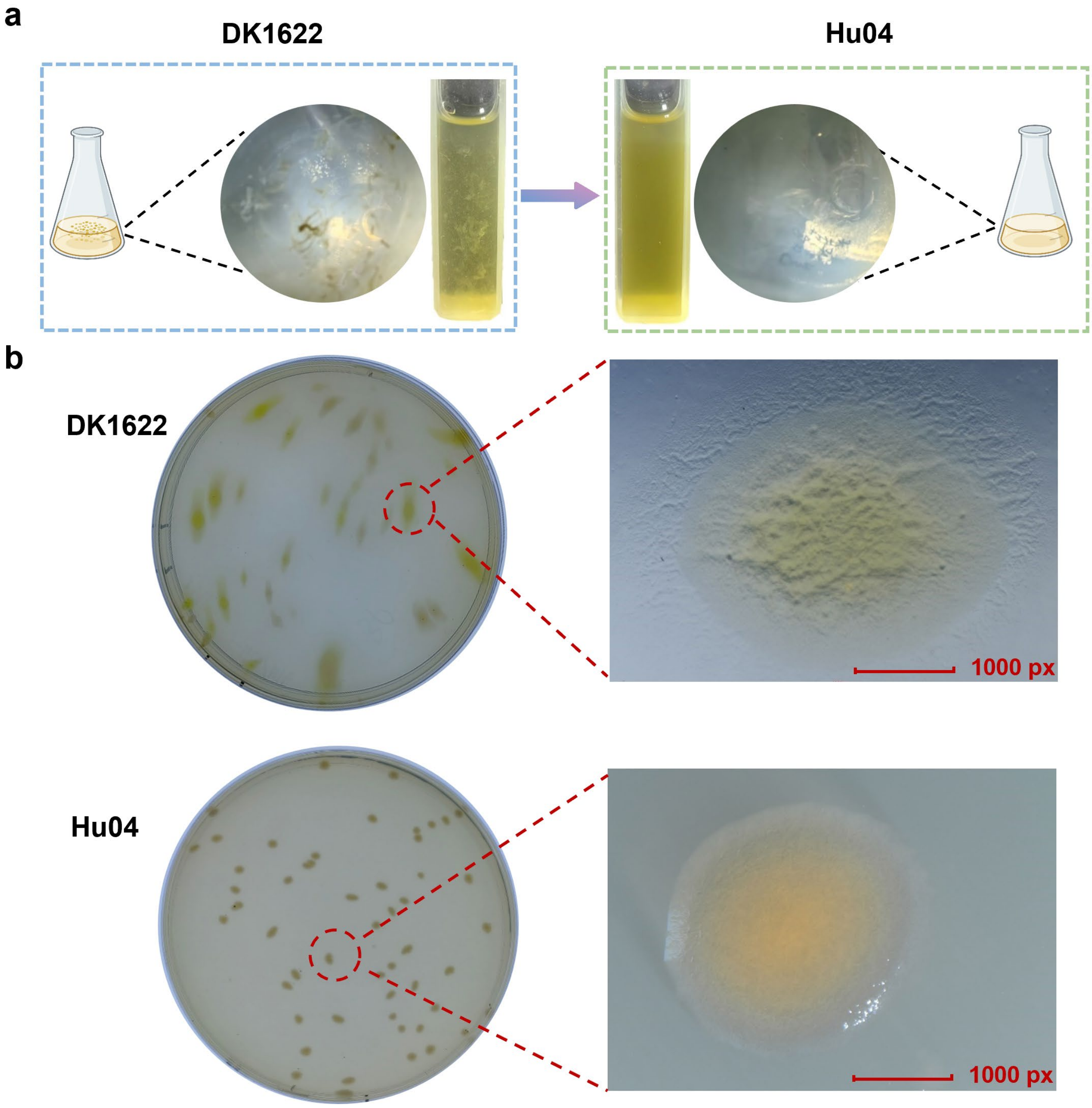

Supplementary Figure 16. Comparison of the growth curve of strain KO7 and with the wide-type DK1622. Source data are provided in the Source Data file.

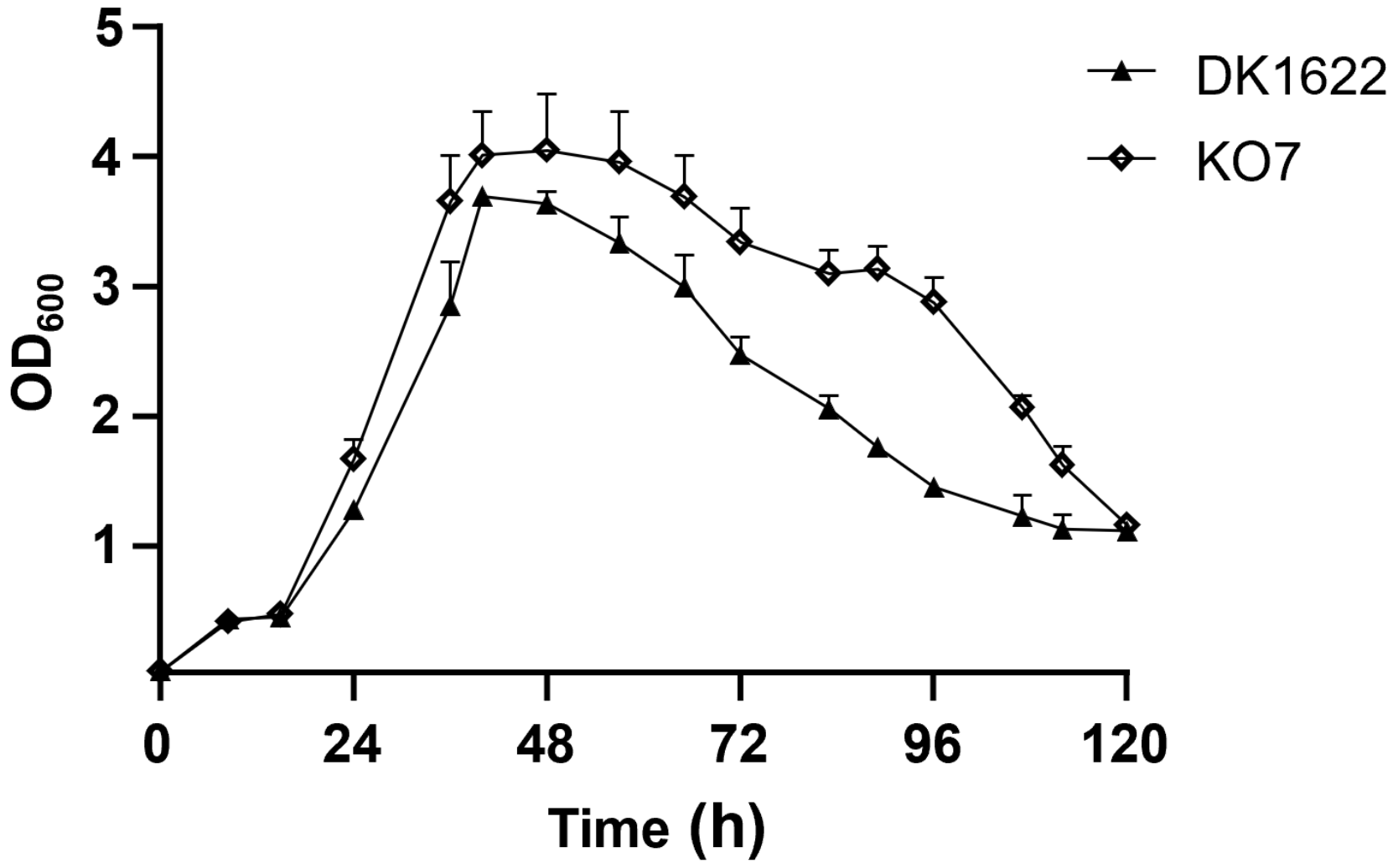

Supplementary Figure 17. Scanning electron microscope (SEM) observation of the strains KO7 and DK1622. Compared with DK1622, KO7 strain exhibited less extracellular matrix and abated cell-cell adhesion.

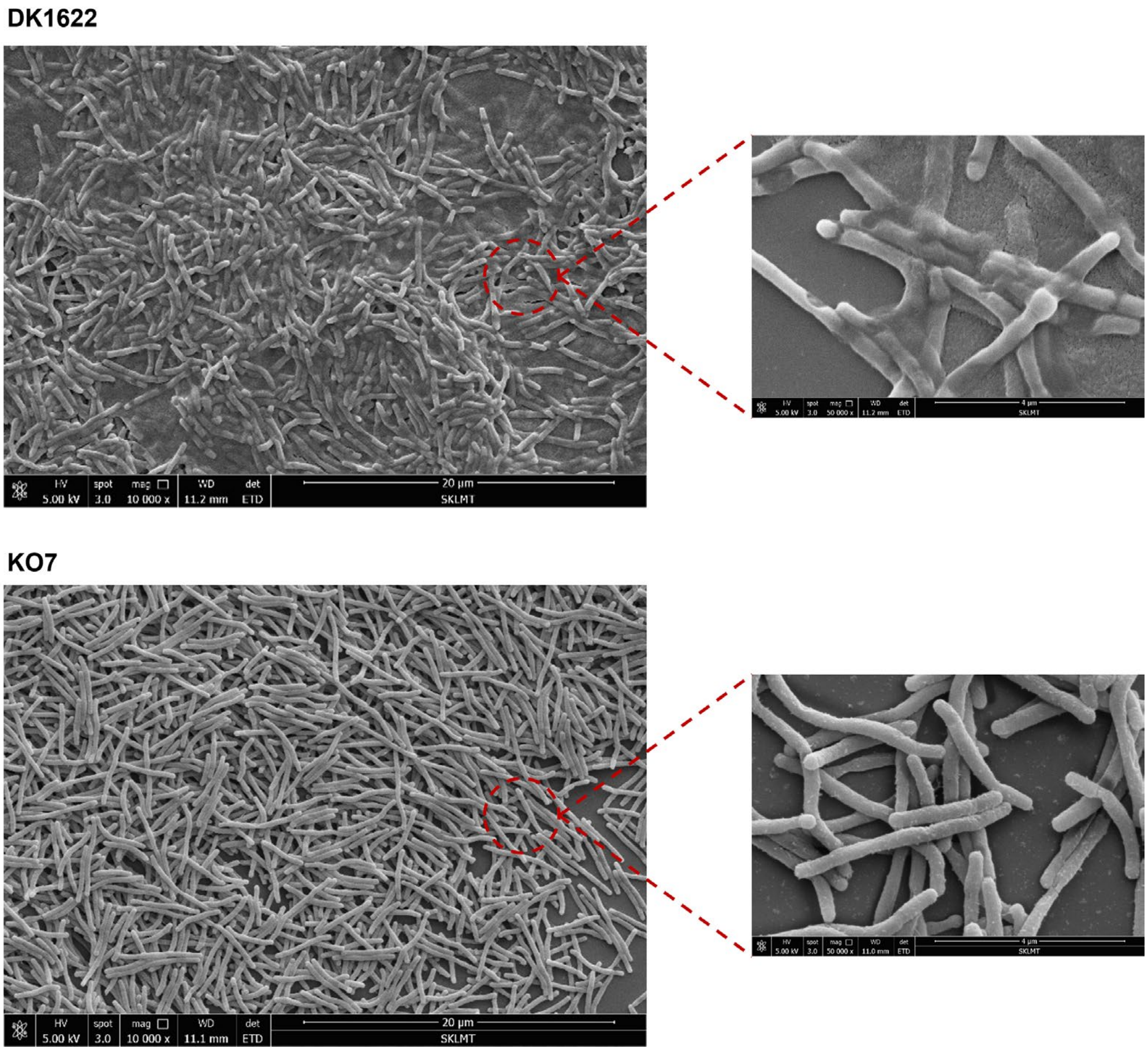

Supplementary Figure 18. Diagrammatic summarization of the genome-reduced mutants of DK1622. (**a**) The distribution of 24 BGCs in the circular genome map of the DK1622 wild type was predicted by the antiSMASH 7.0 analysis. The sequential deletion of different BGCs and genes in DK1622 led to the construction of the mutant *M. xanthus* KO12. (**b**) PCR verification of the deletions of different BGCs. The primers were all designed to be located upstream and downstream of the homology arms of knockout region. If successful deletion occurred, the PCR band would be much smaller than that of the knockout gene cluster, and the size would be approximately 500-1000 bp. M: Trans 2K plus II Marker. Source data are provided in the Source Data file.

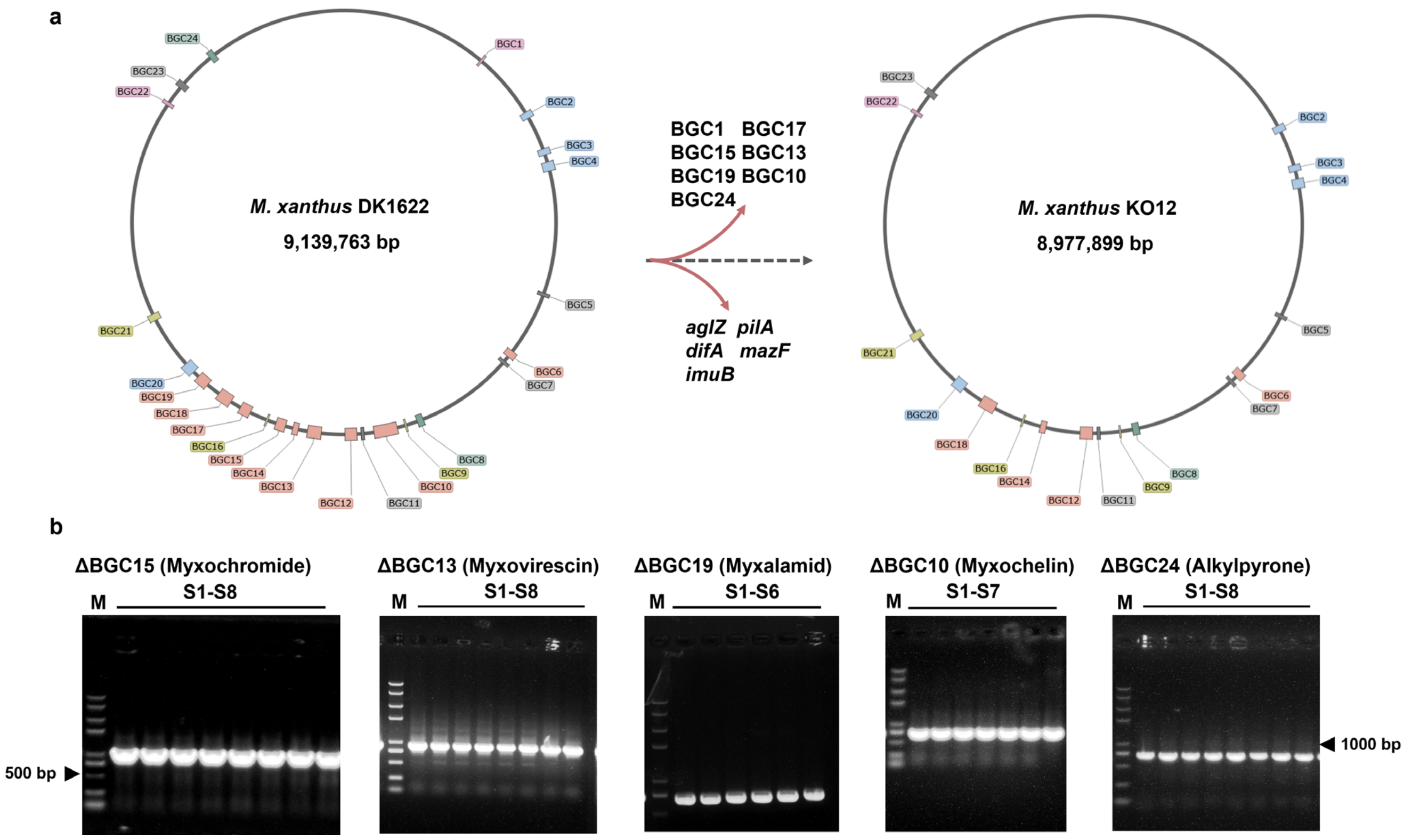

Supplementary Figure 19. HPLC-DAD comparison of wild type DK1622 and the engineered strain KO12. The chromatograms were detected at 254 nm. Source data are provided in the Source Data file.

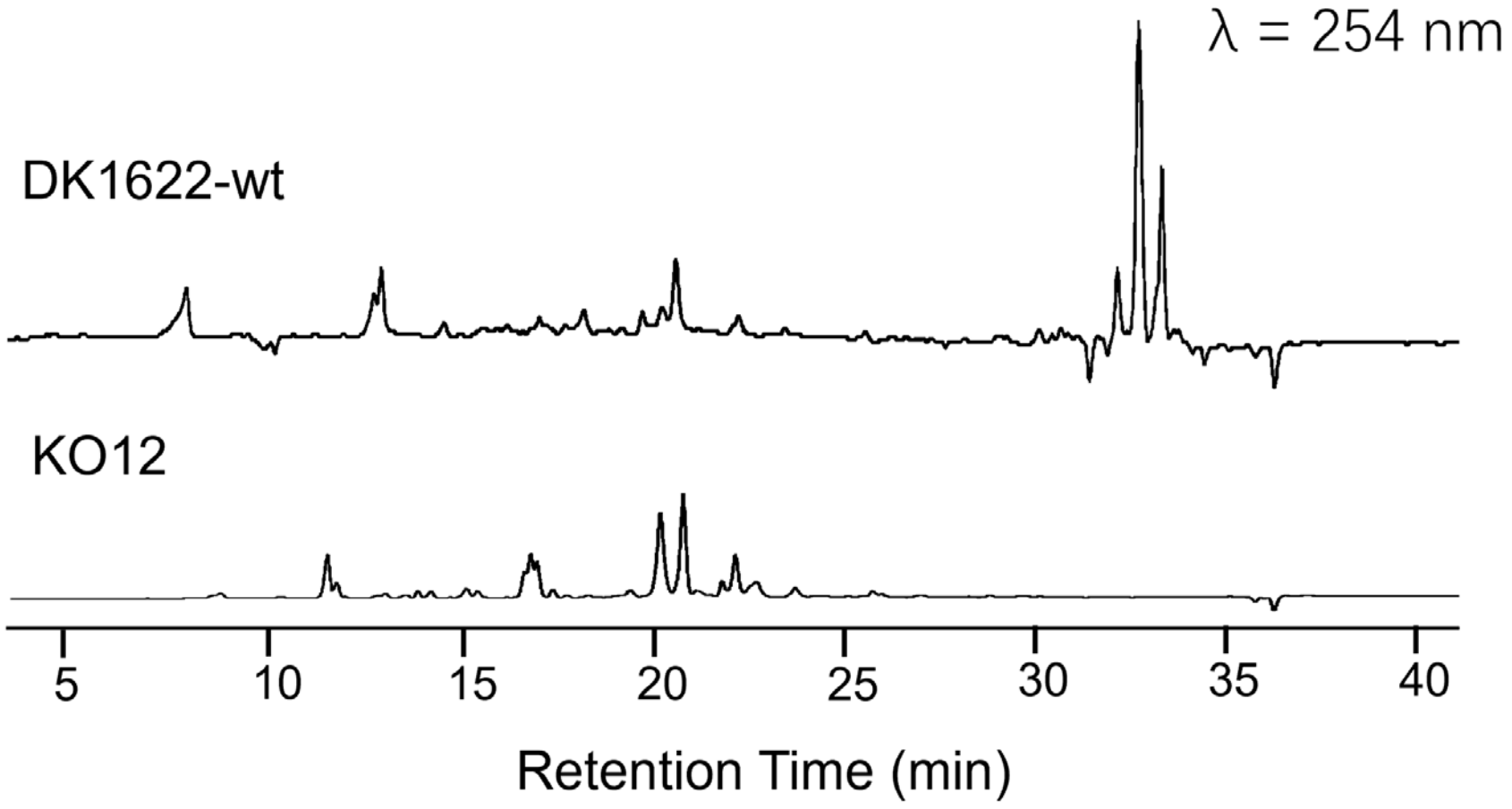

Supplementary Figure 20. ^{1}H NMR-based metabolomic profiling of the engineered strain KO12. Analysis of the ^{1}H NMR of KO12 crude extract revealed the persistent accumulation of various specialized metabolites, key signals attributed to the compound classes of quinolines (highlighted in blue) and cyclodipeptides (highlighted in pink) were indicated. The typical chemical skeletons for quinolinones and cyclodipeptides are attached below.

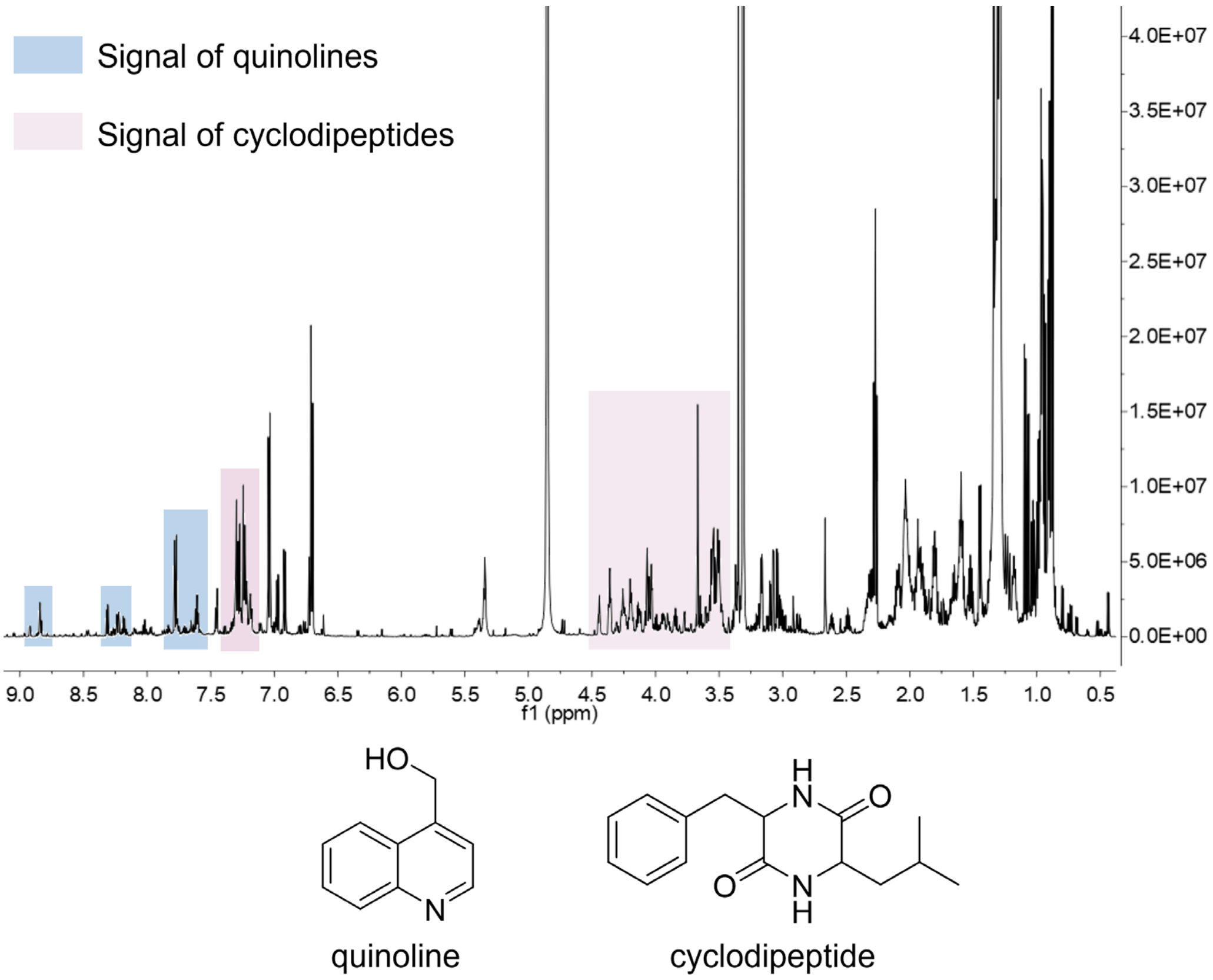

Supplementary Figure 21. Introduce of efflux pump system into the engineered strain IS13. (**a**) Genetic circuit of the multidrug efflux system. gene *A*: drug resistance transporter, EmrB/QacA; gene *B*: RND efflux system, outer membrane lipoprotein; gene *C*: RND family efflux transporter MFP subunit; gene *D*: AcrB/AcrD/AcrF family protein. (**b**) Comparion of minimum inhibitory concentrations (MICs) of doxycycline, tetracycline, penicillin, norfloxacin, azithromycin, and erythromycin of strains IS13 (red) and KO12 (black). The expression of antibiotic efflux pump in IS13 increased its tolerance to the tested antibiotics. (**c**) Comparison of growth curves of the strains IS13 and KO12. Source data are provided in the Source Data file.

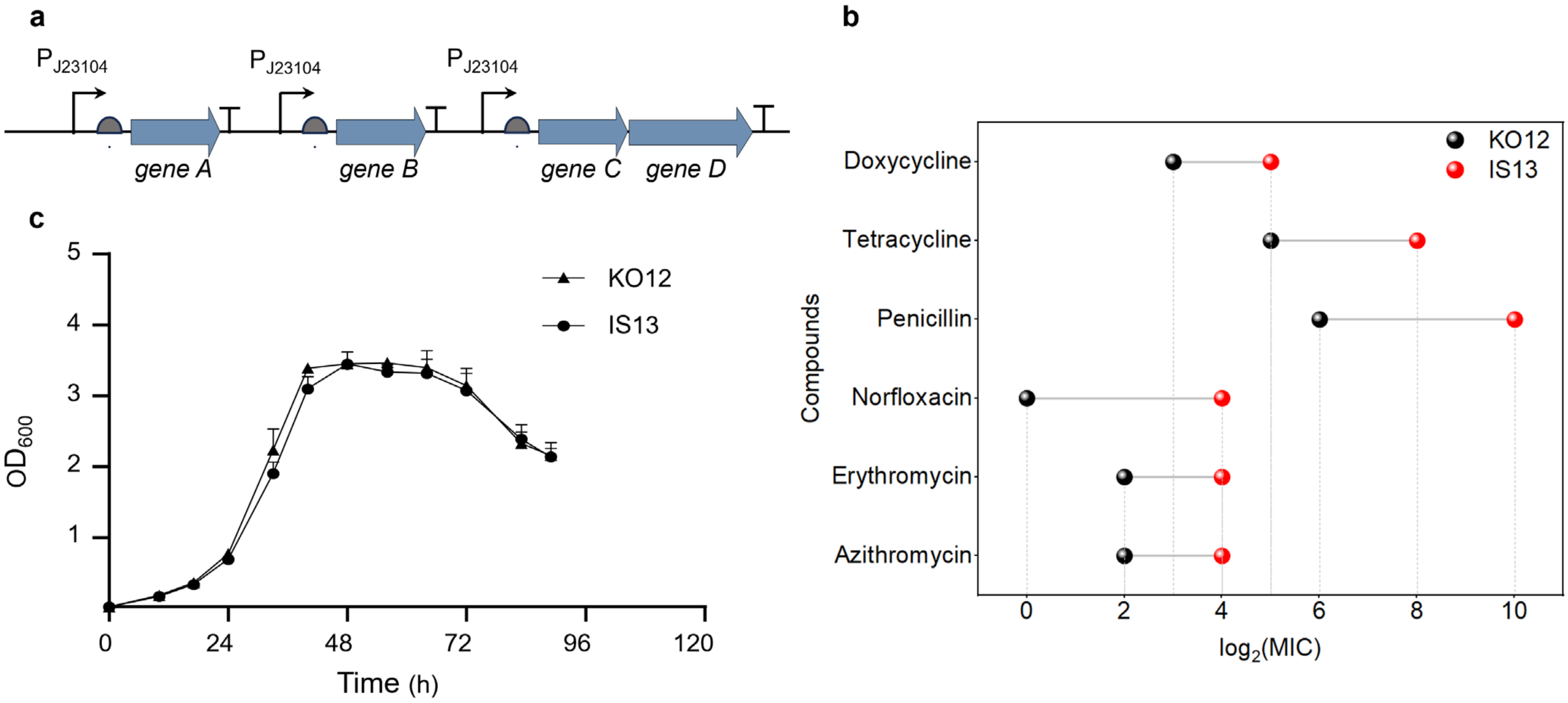

Supplementary Figure 22. Introduction of the NCM pathway into engineered strain IS13 elevated malonyl-CoA levels. (**a**) Gene circuit diagram of the NCM pathway. *cgpanD*: aspartate 1-decarboxylase; *bauA*: aminotransferase; *mcr-C*: malonyl-CoA reductase. (**b**) Schematic of the NCM pathway. (**c**) Standard curve for the quantitative determination of intracellular malonyl-CoA content in microbial samples. The standard curve was constructed by serially diluting malonyl-CoA standards, performing TMB-HRP colorimetric reaction via double-antibody sandwich ELISA, measuring the absorbance (OD value) at a wavelength of 450 nm, and conducting linear fitting of concentration (ng/L) value. The OD values measured from experimental samples can be converted to the corresponding malonyl-CoA concentrations via the regression equation of this curve. The equation and coefficient of determination (R^2) are shown. (**d**) Comparison of intracellular malonyl-CoA level of strains DK1622, IS13, and IS14. Data are presented as mean ± SD, with n = 3 biological replicates. Statistical significance was analyzed by two-tailed Student's t-test using GraphPad Prism 8.0. P value = 0.0021, **P < 0.01. Source data are provided in the Source Data file.

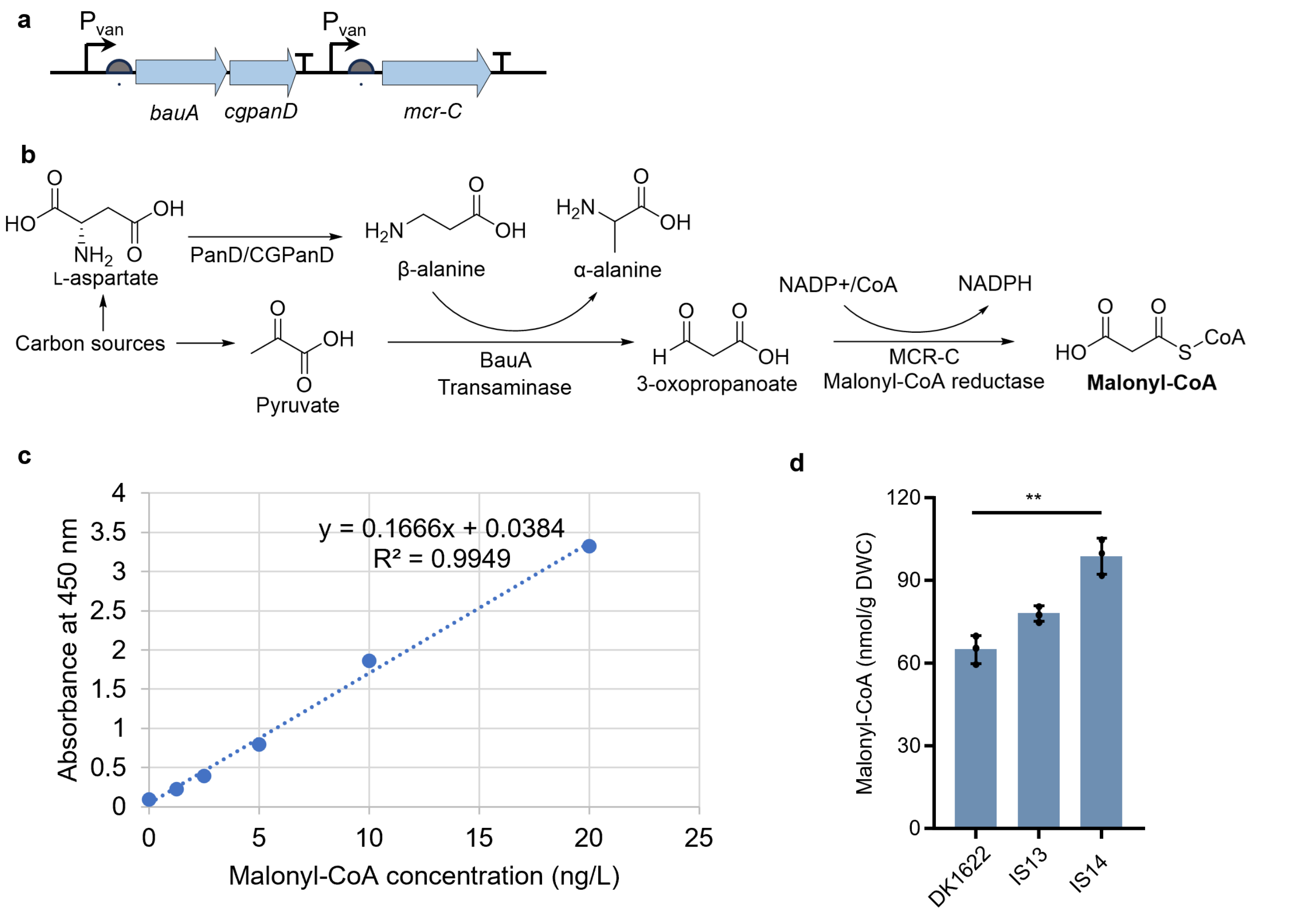

Supplementary Figure 23. Sequence similarity network (SSN) of myxobacterial PKS BGCs constructed by BiG-SCAPE tool. All the myxobacterial PKS BGCs (represented by circles) and the functionally characterized PKS BGCs (represented by squares) from the MIBiG database were analyzed. The different categories of BGCs are shown by different colors. The BGCs *bgc34* from *Archangium violaceum* SDU8 and *vtc* from *Vitiosangium cumulatum* SDU295 are specified. The sequence similarities were calculated for raw distance cutoffs of 0.30–0.70, incremented by 0.05. Results for PKS types were visualized as a sequence similarity network at a cutoff of 0.65. Source data are provided in the Source Data file.

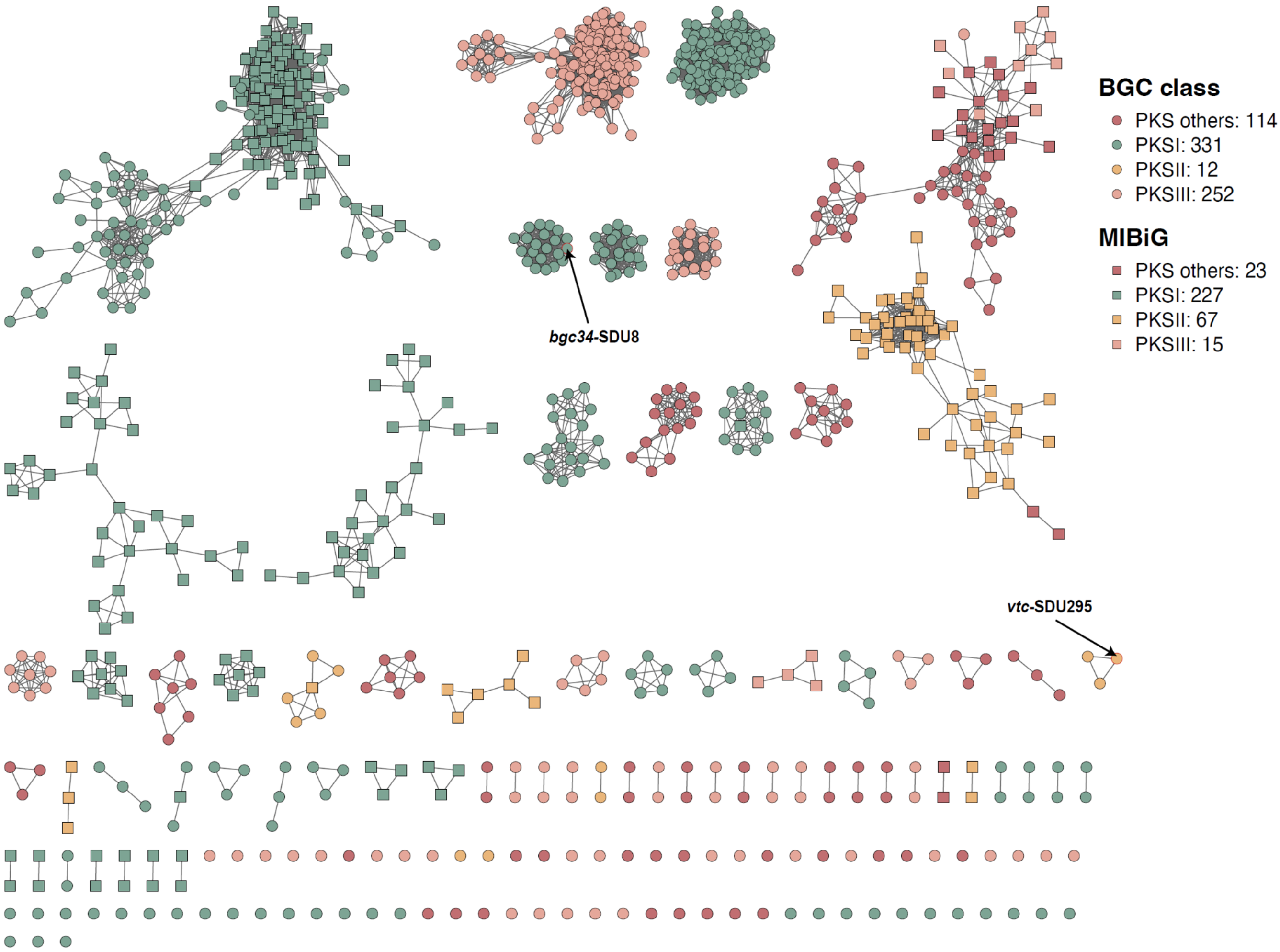

Supplementary Figure 24. Gene organization comparison of the type II BGCs *vtc* and *pcy*. The gene cluster *vtc* is derived from *Vitiosangium cumulatum* SDU295, and *pcy* is derived from *Pyxidicoccus fallax* An d48. The shading of the connecting gray lines indicated the percentage identity of gene sequences, with darker shades representing higher similarity. The core biosynthetic genes shared less than 50% sequence identity, and the tailoring genes were also distinct, collectively indicating that the *vtc* BGC encoded a distinct metabolite from pyxidicyclines. The Structures of pyxidicyclines encoded by *pcy* and anthraquinone encoded by *vtc* are attached below.

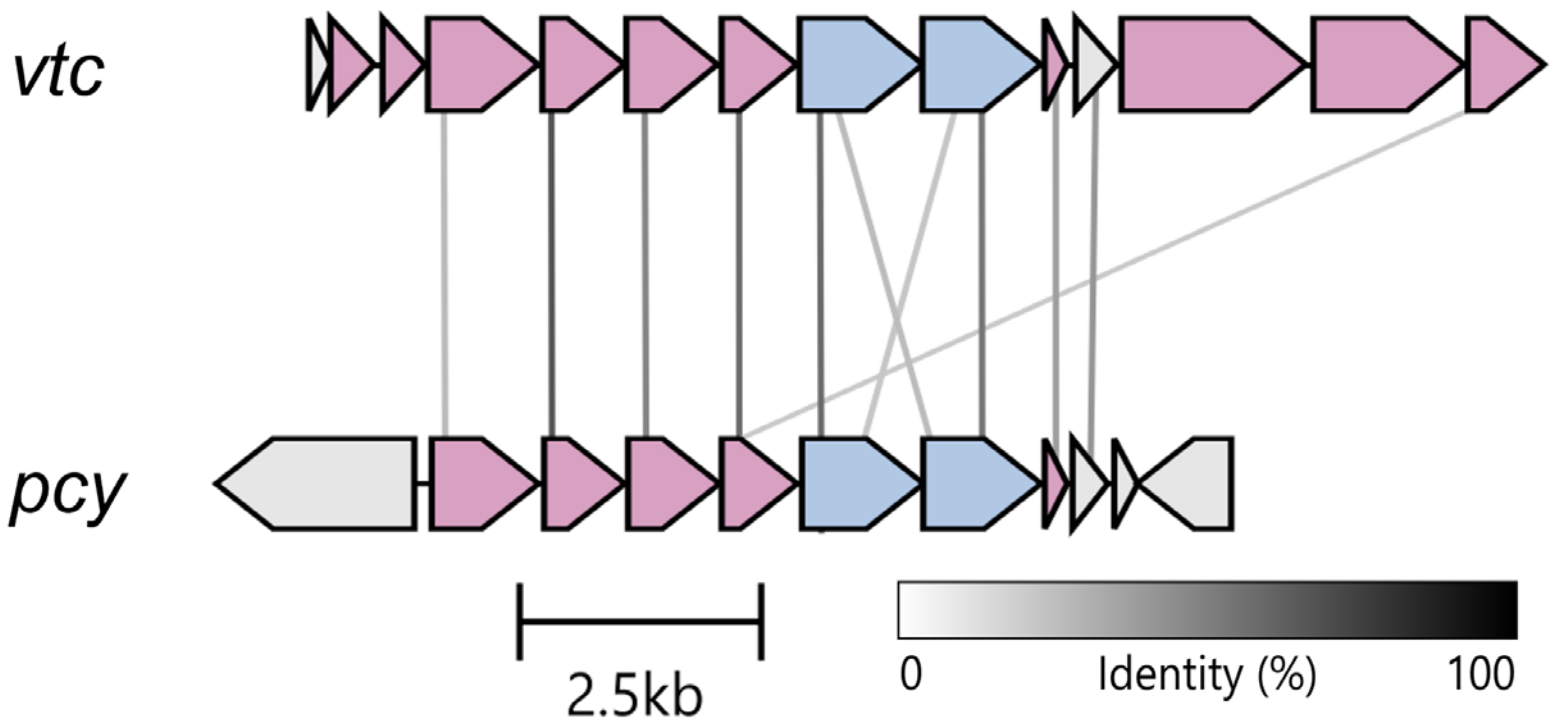

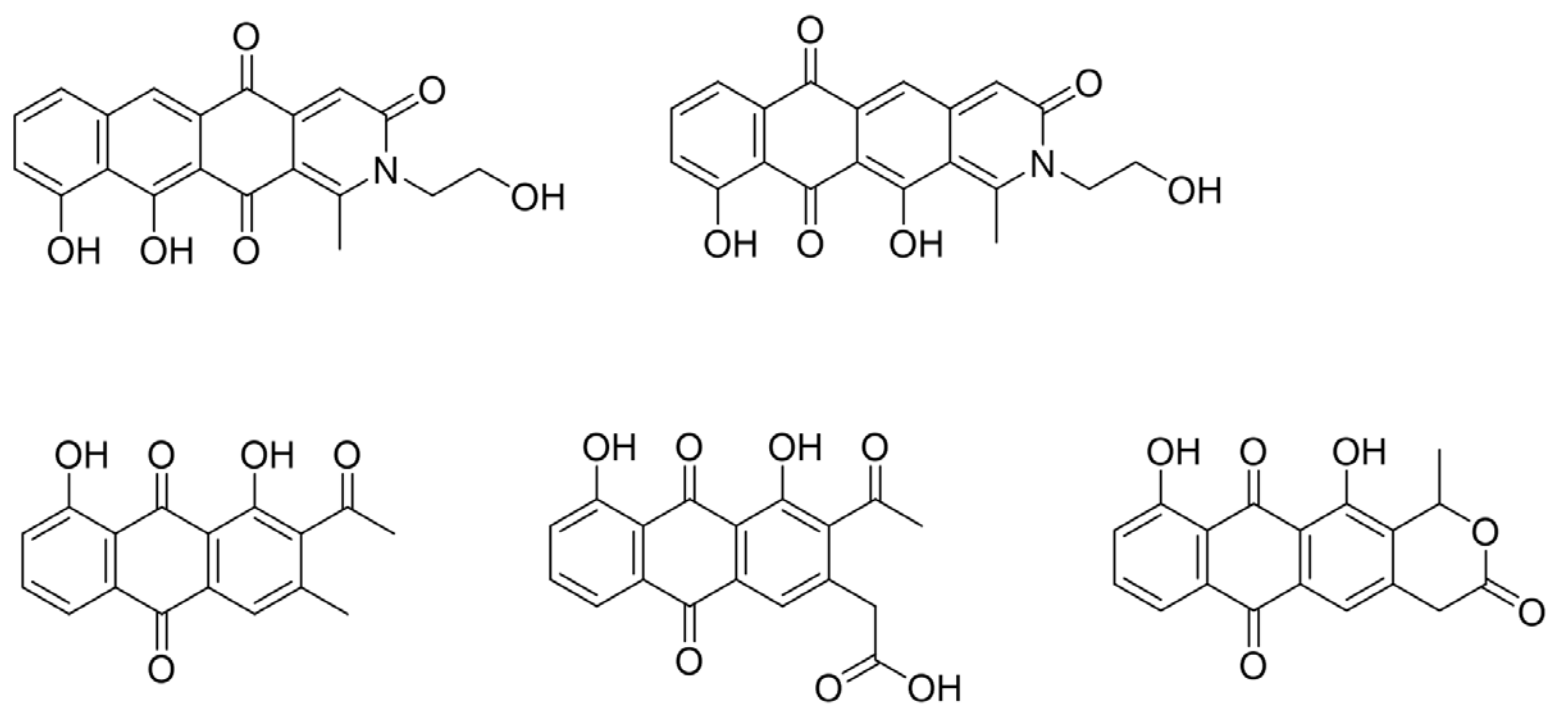

pyxidicyclines encoded by *pcy*

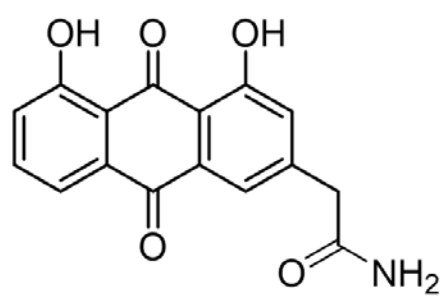

10

anthraquinone encoded by *vtc*

Supplementary Figure 25. Phylogenetic analysis of the two core KS genes in *vtc* BGC. The KS domains within the *vtc* gene cluster were predicted using antiSMASH 7.0. The analysis revealed two adjacent KS domains, designated *vtc*-KS$_{\alpha}$-ORF8 and *vtc*-KS$_{\beta}$-ORF9. The amino acid sequences of *vtc*-KS$_{\alpha}$-ORF8 and *vtc*-KS$_{\beta}$-ORF9 were analyzed using the online tool NaPDoS2, which retrieved 414 relevant KS sequences from the database. The phylogenetic tree was constructed with these 414 sequences along with the two query sequences, and the result was visualized using iTOL. Source data are provided in the Source Data file.

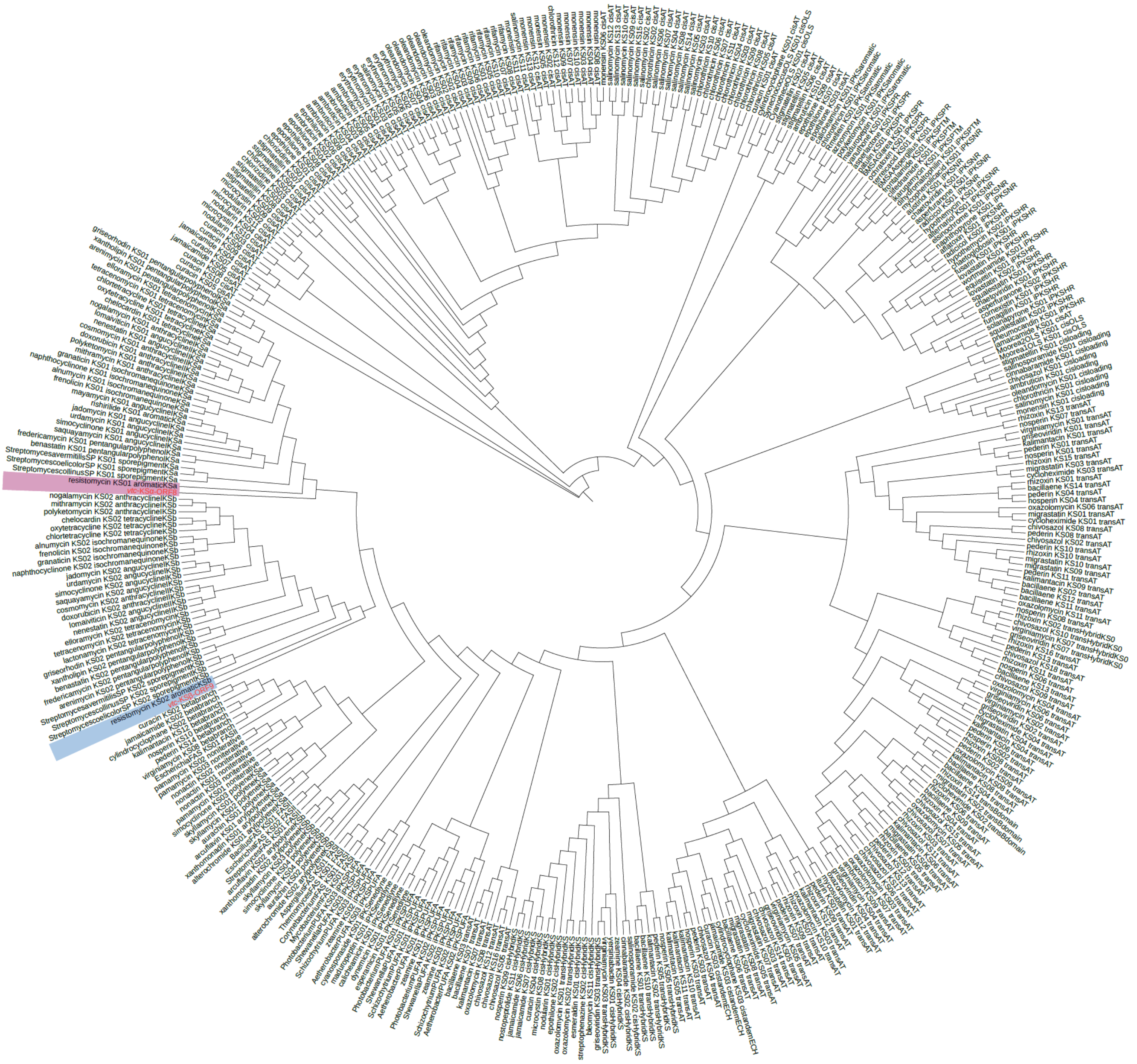

Supplementary Figure 26. 2D NMR correlation analysis of new compounds. HMBC ( ), ^{1}H–^{1}H COSY (━) of new compounds **4**–**6**, and **8**–**10,** and key ^{1}H–^{1}H NOESY ( ) of new compounds **5**, **6**.

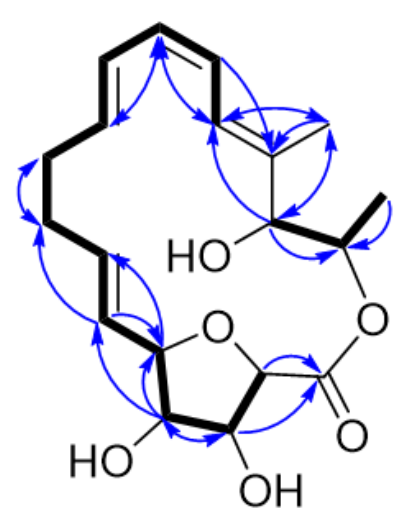

4

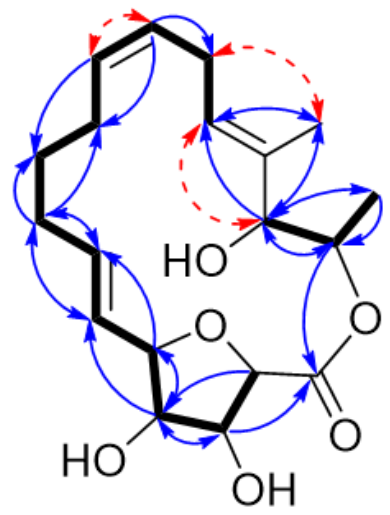

5

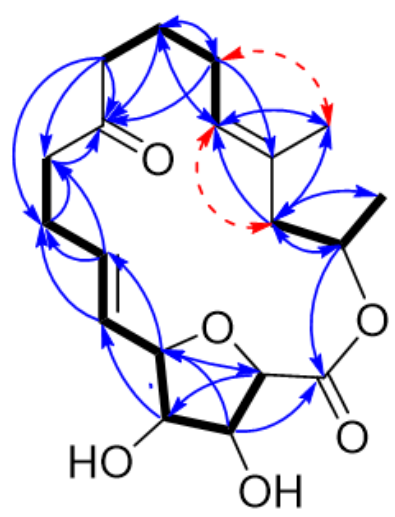

6

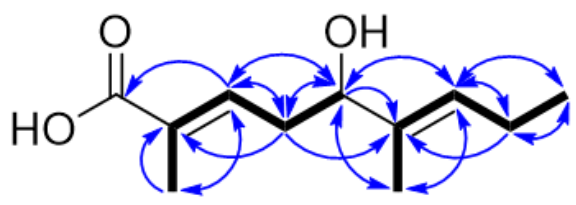

8

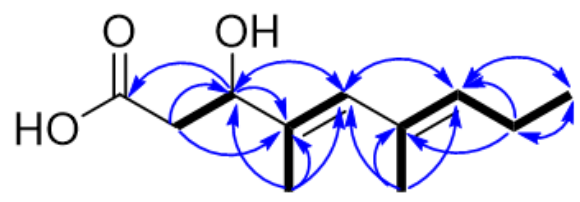

9

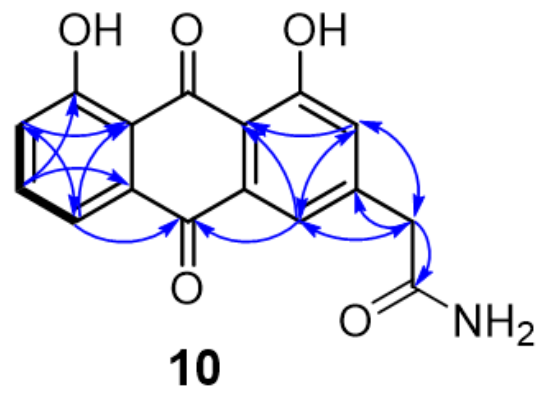

10

Supplementary Figure 27. **Experimental and calculated ECD spectra of compounds 8 and 9.** Source data are provided in the Source Data file.

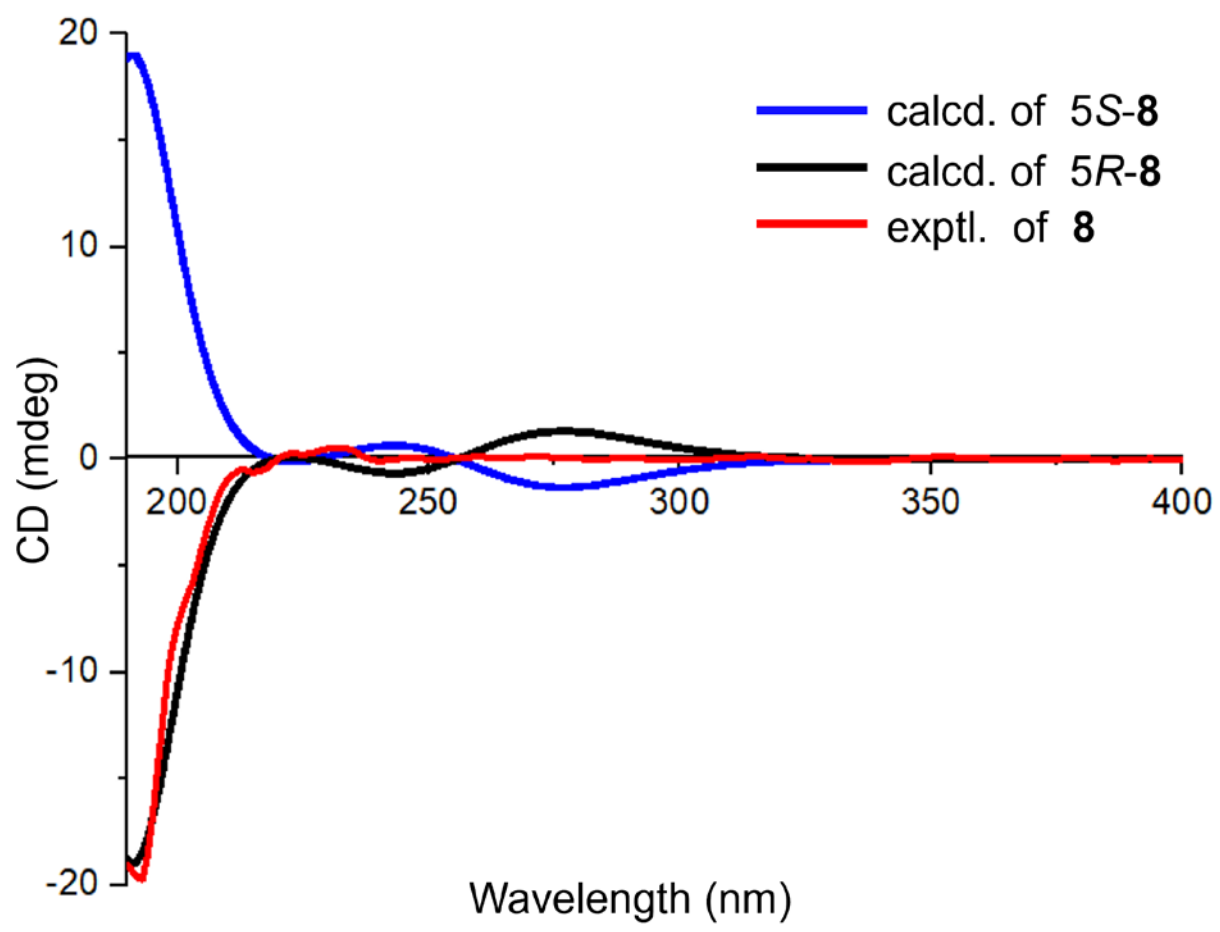

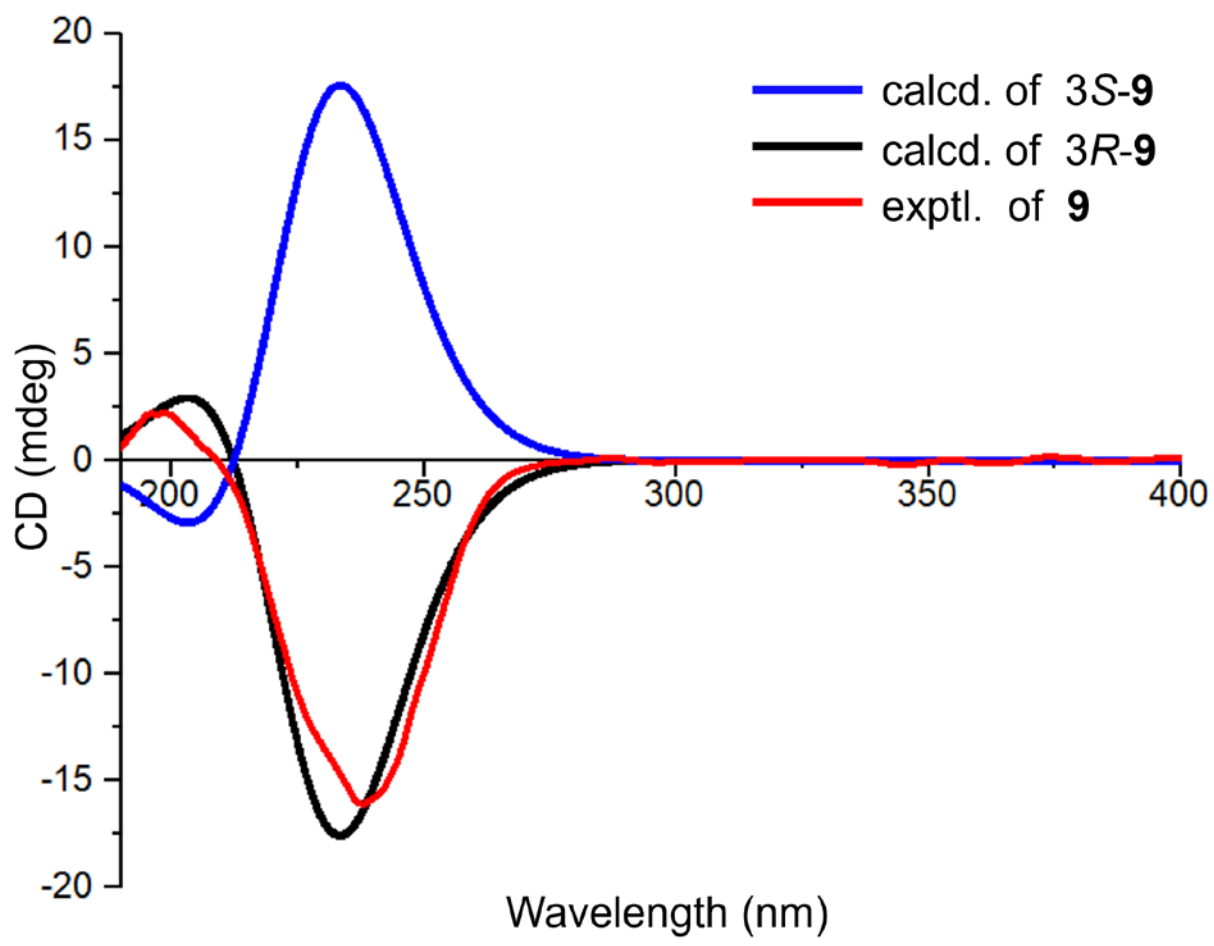

Supplementary Table 1. Gene annotations for *bgc34* in *Archangium gephyra* SDU8.

ORF	Size (bp)	Proposed function	NCBI similarity	ID%
1	237	unknown	twin-arginine translocase TatA/TatE family subunit [*Archangium violaceum*]; WP_204219910.1	100%
2	1002	input-output signaling	HAMP domain-containing histidine kinase [*Archangium violaceum*]; QRN93957.1	99.70%
3	372	regulation	response regulator [*Archangium violaceum*]; WP_204219908.1	100%
4	330	lipid binding and transfer activities	SCP2 sterol-binding domain-containing protein [*Archangium violaceum*]; WP_204219907.1	100%
5	1173	unknown	CaiB/BaiF CoA-transferase family protein [*Archangium violaceum*]; WP_204219906.1	100%
6	7743	type I polyketide biosynthesis	type I polyketide synthase [*Archangium violaceum*]; WP_204219905.1	100%
7	9687	type I polyketide biosynthesis	type I polyketide synthase [*Archangium violaceum*]; WP_204219904.1	100%
8	2355	type I polyketide biosynthesis	type I polyketide synthase [*Archangium violaceum*]; WP_204219903.1	100%
9	495	regulation	response regulator [*Archangium violaceum*]; WP_204219902.1	100%
10	2193	ATP-binding	ATP-binding protein [*Archangium violaceum*]; WP_239469846.1	100%
11	507	regulation	response regulator [*Archangium violaceum*]; WP_204219901.1	100%
12	825	unknown	hypothetical protein [*Archangium violaceum*]; WP_204219900.1	100%

Supplementary Table 2. Gene annotations for *vtc* in *Vitiosangium* sp. SDU295.

ORF	Size (bp)	Proposed function	NCBI similarity	ID%
1	231	unknown	hypothetical protein [*Vitiosangium* sp.]; WP_108073787.1	93.42%
2	459	unknown	hypothetical protein [*Vitiosangium* sp.]; WP_158502052.1	97.37%
3	447	cyclase/dehydrase	SRPBCC family protein [*Vitiosangium* sp.]; WP_158502051.1	97.97%
4	1161	Peptidase_S9	alpha/beta fold hydrolase [*Vitiosangium* sp.]; WP_108073786.1	98.19%
5	864	biosynthetic-additional	cyclase family protein [*Vitiosangium* sp.]; WP_108073935.1	98.61%
6	960	cyclase/dehydrase	SRPBCC family protein [*Vitiosangium* sp.]; WP_158502050.1	98.73%
7	792	short-chain dehydrogenase/reductase SDR	SDR family oxidoreductase [*Vitiosangium* sp.]; WP_108073784.1	99.24%
8	1296	Alpha-ketoacyl synthase	beta-ketoacyl-[acyl-carrier-protein] synthase family protein [*Vitiosangium* sp.]; WP_199243092.1	98.84%
9	1248	Beta-ketoacyl synthase	beta-ketoacyl-[acyl-carrier-protein] synthase family protein [*Vitiosangium* sp.]; WP_108073782.1	99.04%
10	237	biosynthetic-additional (rule-based-clusters) PP-binding	[acyl carrier protein [*Archangium violaceum*]; WP_204225857.1	84.62%
11	435	unknown	holo-ACP synthase [*Vitiosangium* sp.]; WP_108073780.1	97.9%
12	1941	asparagine synthase (glutamine-hydrolyzing)	asparagine synthase (glutamine-hydrolyzing) [*Vitiosangium* sp.]; WP_108073779.1	99.07%
13	1587	dehydrogenase	NAD(P)/FAD-dependent oxidoreductase [*Vitiosangium* sp.]; WP_108073777.1	98.86%
14	804	short-chain dehydrogenase/reductase SDR	SDR family NAD(P)-dependent oxidoreductase [*Vitiosangium* sp.]; WP_108073776.1	98.50%

Supplementary Table 3. List of strains used in this study.

Strain	Genotype or description	Source or reference
Escherichia coli DH5α	Δ(lacZYA-argF) U169; *recA1*; *endA1*; *hsdR17*(r_k^- m_k^+);λ^-	Obtained from Company
Escherichia coli RedGB2005	Linear-to-circular recombination	Laboratory collection
Escherichia coli Dir	Linear-by-linear recombination	Laboratory collection
Myxococcus xanthus DK1622	Wild-type strain	D.Kaiser University of Standford
Myxococcus xanthus Hu04	DK1622 Δ*aglZ* Δ*pilA* Δ*difA* Δ*mazF* ΔBGC1 ΔBGC17	Laboratory collection
KO7	Hu04 Δ*imuB*	This study
KO8	Hu04 Δ*imuB* ΔBGC15	This study
KO9	Hu04 Δ*imuB* ΔBGC15 ΔBGC13	This study
KO10	Hu04 Δ*imuB* ΔBGC15 ΔBGC13 ΔBGC19	This study
KO11	Hu04 Δ*imuB* ΔBGC15 ΔBGC13 ΔBGC19 ΔBGC10	This study
KO12	Hu04 Δ*imuB* ΔBGC15 ΔBGC13 ΔBGC19 ΔBGC10 ΔBGC24	This study
IS13	KO12+EmrB- RND	This study
IS14	KO12+EmrB- RND+ NCM pathway	This study

Supplementary Table 4. List of plasmids used in this study.

Plasmid name	Description	Source or reference	Use
pSWU19	Site-specific integration vector with Mx8 *attB* integration site; Kmr	Laboratory collection	Targeted integration of the RecET system into the DK1622 or Hu04 genome
pZJY4111	Self-replicating plasmid;Apra or Cmr	Laboratory collection	Express the TnpB system or Cre protein
pBAC	F factor; low copy number; typically up to 300 kb or more;Cmr or Apra	From a company	Construct a BAC library; Capture the gene cluster
pMiniHimar	Transposase; random integration into the genome;Kmr	Laboratory collection	Gene cluster was inserted into MxPKS
pSWU19-MxRecET	Assemble the MxRecET system into the pSWU19 plasmid via Gibson assembly	This study	Targeted integration of the MxRecET system into the DK1622 or Hu04 genome
pSWU19-ARecET	Assemble the ARecET system into the pSWU19 plasmid via Gibson assembly	This study	Targeted integration of the ARecET system into the DK1622
pSWU19-LRecET	Assemble the LRecET system into the pSWU19 plasmid via Gibson assembly	This study	Targeted integration of the LRecET system into the DK1622
pSWU19-NRecET	Assemble the NRecET system into the pSWU19 plasmid via Gibson assembly	This study	Targeted integration of the NRecET system into the DK1622
pZJY4111-Cre	Assemble the Cre system into the pZJY4111 plasmid via Gibson assembly	This study	Express the Cre protein in DK1622 or Hu04
pZJY4111-ISDra2-Myxochromide	Construct a TnpB knockout system targeting the *mch* gene cluster via Gibson assembly	This study	Scarless knockout of the *mch* gene cluster
pZJY4111-ISDra2-Myxovirescin	Construct a TnpB knockout system targeting the Myxovirescin gene cluster via Gibson assembly	This study	Scarless knockout of the Myxovirescin gene cluster
pZJY4111-ISDra2-Myxalamid	Construct a TnpB knockout system targeting the Myxalamid gene cluster via Gibson assembly	This study	Scarless knockout of the Myxalamid gene cluster
pZJY4111-ISDra2-Myxochelin	Construct a TnpB knockout system targeting the Myxochelin gene cluster via Gibson assembly	This study	Scarless knockout of the Myxochelin gene cluster
pZJY4111-ISDra2-Alkylpyrone	Construct a TnpB knockout system targeting the Alkylpyrone gene cluster via Gibson assembly	This study	Scarless knockout of the Alkylpyrone gene cluster
pZJY4111-ISDra2-*imuB*	Construct a TnpB knockout system targeting the gene *imuB* via Gibson assembly	This study	Scarless knockout of the gene *imuB*
pBAC-*φC31*-*bgc34*	J23104; *φC31* integrase; *bgc34*	This study	Site-specific integration of the *bgc34* BGC into the genome
pMiniHimar-*corA*	pSH059; transposase; *corA*	This study	Random integration of the *corA* gene into the genome
pBAC-*φC31*-*arc*	J23104; *φC31* integrase; archangumide	This study	Site-specific integration of the *arc* BGC into the genome
pBAC-*φC31*-*vtc*	J23117; *φC31* integrase; Type II PKS	This study	Site-specific integration of the *vtc* BGC into the genome

Supplementary Table 5. List of primers used in this study.

Primer	Sequence (5-3)*	Use
MyqaJRecT-F	CGAAAGAGGAGAAATACTAGATGCTCGCTGAACGCGTC	Amplification of MyqaJRecT(MxRecET system) fragments with pSWU19-derived homology arms
MyqaJRecT-R	GTTATTGCTCAGCGGTGGCACTACGTGGCCGCCGTCTC	
pSWU19-F	TGCCACCGCTGAGCAATAACTAG	Amplification of pSWU19 vector backbone
pSWU19-R	CTAGTATTTCTCCTCTTTCGCTAGAGGAATTG	
MyqaJRecT-check-F	CGCGGCCTTTTTACGGTTCC	Check for the integration of the RecET system into the genome
MyqaJRecT-check-R	GGTCCACCGCATCCCAAATC	
Cre-F	AAAGAGGAGAAATACTAGATGTCCAACCTGCTGACGGT	Amplification of cre fragments with pZJY4111-derived homology arms
Cre-R	ACCGCCTTTGAGTGAGCTTTTCAGTCGCCGTCCTCCAG	
pZJY4111-F	AGCTCACTCAAAGGCGGTAATACG	Amplification of pZJY4111 vector backbone
pZJY4111-R	CTAGTATTTCTCCTCTTTCTCTAGAGGAATTGTTATCC	
Cre-check-F	GGAACCTCTTACGTGCCGATCA	Check for the integration of the cre into the DK1622 or Hu04
Cre-check-R	GAGAAAGCGCCACGCTTCC	
ISDra2-Myxovirescin-guide-F	ATGATAAGGAATAAGGCTTTCGTGGTCAGG	Amplification ISDra2 with a guide targeting the Myxovirescin gene cluster
ISDra2-Myxovirescin-guide-R	CCGCGGTCTTCATCACCTGCTTGAACCTCACACGACTAAAGTCGC	
ISDra2-Myxochelin-guide-F	GCTCCACACCTTCCTCGGCCGAATTCTTTTTTCCAGGCATCAAATAAAACGAAAGG	Amplification pZJY4111-ISDra2 with a guide targeting the Myxochelin gene cluster
ISDra2-Myxochelin-guide-R	GGCCGAGGAAGGTGTGGAGCTTGAACCTCACACGACTAAAGTCGC	
ISDra2-Alkylpyrone-guide-F	GAAGACTCACACCGAAGAAAGAATTCTTTTTTCCAGGCATCAAATAAAACGAAAGG	Amplification pZJY4111-ISDra2 with a guide targeting the Alkylpyrone gene cluster
ISDra2-Alkylpyrone-guide-R	TTTCTTCGGTGTGAGTCTTCTTGAACCTCACACGACTAAAGTCGC	
ISDra2-check-F	TGAGCTGATACCGCTCGCC	Check for the presence of ISDra2 during the knockout procedure
ISDra2-check-R	CCGTGGGAATTTCCGCAATCC	
Myxovirescin-check-F	CTTCCGGAGGGAACTCACGG	Verify the successful knockout of the Myxovirescin biosynthetic gene cluster
Myxovirescin-check-R	GTTCGTCATCGCCGACCGTT	
Myxochelin-check-F	GGAGACGGACTTCATCCGCG	Verify the successful knockout of the Myxochelin biosynthetic gene cluster
Myxochelin-check-R	GTATTCGCCGGGTCGAGCT	
Alkylpyrone-check-F	GGAAGAAGACGACCTCAAGCGC	Verify the successful knockout of the Alkylpyrone biosynthetic gene cluster
Alkylpyrone-check-R	GCCGCGTTCACCGTCAGA	
bgc34-check-F	TGAAGTCCCGGATCTCCCGT	Detect the electroporation of *bgc34* into IS14
bgc34-check-R	CATGGCGCGTCACCATCT	
arc-check-F	ACCAGATCGACGAGCACGAATC	Detect the electroporation of archangumide bgc into IS14
arc-check-R	ATGCCAGGCCGTCTGCAT	
vtc-check-F	CGCGAGCAAGGGCATCAC	Detect the electroporation of Type II PKS bgc into IS14
vtc-check-R	CCACGCATACGCCCATCC	
corA-check-F	CGACCGCTTCGTTCCAGTCC	Detect the electroporation of corA bgc into IS14
corA-check-R	GGAACACGGCGGCATCAGAG	

Supplementary Note 1. NMR data assignment of **4** in methanol-*d₄*.

The HRESIMS analysis of compound **4** exhibited an $[M + H - H_2O]^+$ peak at *m/z* 333.1697 (calcd for $C_{19}H_{25}O_5$, 333.1697), establishing its molecular formula as $C_{19}H_{26}O_6$. The degree of unsaturation remained consistent with that of **3** (archangiumide). Detailed comparison of the ^1H and DEPTQ NMR data (Supplementary Table 6) revealed that the major differences between compound **4** and **3** were the disappearance of the characteristic allenyl group in **3** and the emergence of new olefinic signals. Further analysis of COSY, HSQC, and HMBC correlations (Supplementary Figure 26) established three contiguous spin systems exhibiting similar coupling constants, consistent with the presence of a conjugated diene. This evidence indicated that the $\Delta^{11,\,12}$ double bond in the allene group of **3** had migrated to the $\Delta^{10,\,11}$ position in compound **4.** The large coupling of 15.6 Hz established an *E* for the double bond $\Delta^{10,\,11}$, while the small coupling of 10.8 Hz indicated a *Z* for the double bond $\Delta^{12,\,13}$. The ^1H NMR signals of H-6 and H-7 overlapped, precluding determination of the double-bond geometry by coupling constants. However, given that the absolute configurations of **3** have been unambiguously determined by X-ray diffraction, the shared biosynthetic origin of compounds **3** and **4** from modular PKS assembly line of *arc* gene cluster enabled us to assign *E* configurations of double bonds $\Delta^{6,\,7}$ and $\Delta^{14,\,15}$, as well as *16S, 17R*, respectively. Furthermore, the comparative analysis of ^1H NMR chemical shifts and coupling constants at the postions C16 and C17 with those of compound **3** also supported this assignment.

Supplementary Table 6

Pos.	δ_C, type	δ_H, mult. (*J* in Hz)
1	171.4, C	–
2	81.8, CH	4.43, d (7.2)
3	79.4, CH	4.26, t (7.2)
4	81.3, CH	3.93, t (7.2)
5	86.1, CH	3.99, t (7.2)
6	132.2, CH	5.69, m
7	137.0, CH	5.67, m
8	30.5, CH_2	2.32, m; 2.42, m
9	30.9, CH_2	2.24, m; 2.38, m
10	133.2, CH	5.63, dt (15.6, 5.4)
11	129.3, CH	6.65, dd (15.6, 11.4)
12	130.8, CH	5.92, t (10.8)
13	124.3, CH	6.05, t (10.8)
14	125.6, CH	6.55, d (11.4)
15	136.7, C	–
16	81.0, CH	4.00, d (9.6)
17	72.4, CH	4.95, dq (9.6, 6.0)
18	18.2, CH_3	1.37, d (6.0)
19	12.1, CH_3	1.72, s

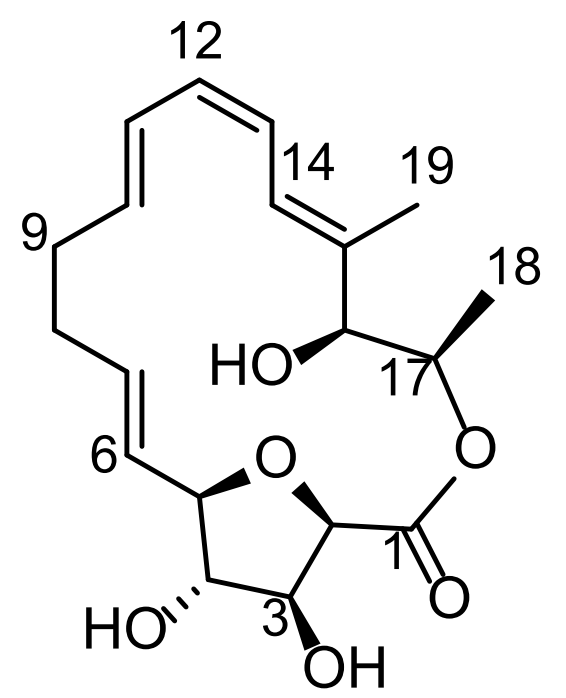

Supplementary Note 2. NMR data assignment of **5** in methanol-d_4.

The HRESIMS analysis of **5** exhibited an $[M + Na]^+$ ion at *m/z* 375.1782 (calcd for $C_{19}H_{28}O_6Na$, 375.1784), establishing a molecular formula of $C_{19}H_{28}O_6$. This molecular formula corresponds to one degree of unsaturation less than that of **4**. The absence of one set of olefinic signals in the DEPTQ and HSQC spectra confirmed the reduction of a double bond. The site of unsaturation loss of $\Delta^{10,11}$ double bond was pinpointed through detailed 2D NMR analysis (Supplementary Figure 26). Key HMBC correlations from H-12 to C-10, H-8 to C-10 and from H-11 to C-9, in conjunction with the COSY spin system of H_2-9/H_2-10/H_2-11, defined a -CH_2-CH_2-CH_2-CH=CH- fragment. The configurations of the remaining double bonds were determined from coupling constants and NOE data. For $\Delta^{11,12}$, the small coupling constant (10.8 Hz) between H-11 and H-12 supported a *Z* configuration. For $\Delta^{14,15}$, an *E* geometry was assigned based on NOE correlations between H_2-13 and H_3-19, as well as between H-14 and H-16. The double bond $\Delta^{6,7}$ was difficult to assign due to severe signal overlap in the deuterated methanol. However, upon switching the NMR solvent to pyridine-d_5, the alkene proton signals for H-6 and H-7 became fully resolved, revealing a large coupling constant (J = 15.0 Hz) consistent with an *E* configuration. Likewise, the stereochemistry of **5** (*2R, 3S, 4S, 5R, 16S, 17R*) was assigned by comparative analysis of its ^{1}H NMR chemical shifts and coupling constants (Supplementary Table 7) at the relevant positions with those of compounds **3** and **4**.

Supplementary Table 7

Pos.	δ_C, type	δ_H, mult. (*J* in Hz)
1	171.8, C	–
2	81.4, CH	4.44, d (7.2)
3	78.2 CH	4.30, t (7.8)
4	80.4, CH	3.90, t (7.8)
5	85.7, CH	4.00, t (7.8)
6	131.9, CH	5.56, m
7	136.5, CH	5.60, m
8	30.6, CH_2	2.10, m
9	28.4, CH_2	1.57, m
10	25.2, CH_2	1.85, m; 2.37, m
11	131.2, CH	5.28, td (10.8, 3.6)
12	130.2, CH	5.46, td(10.8, 3.6)
13	26.9, CH_2	2.61, m; 2.97, m
14	130.8 CH	5.28, t (10.8)
15	135.6, C	–
16	82.3, CH	3.92, d (9.6)
17	72.5, CH	4.84, m
18	18.5, CH_3	1.33, d (6.0)
19	10.8, CH_3	1.55, s

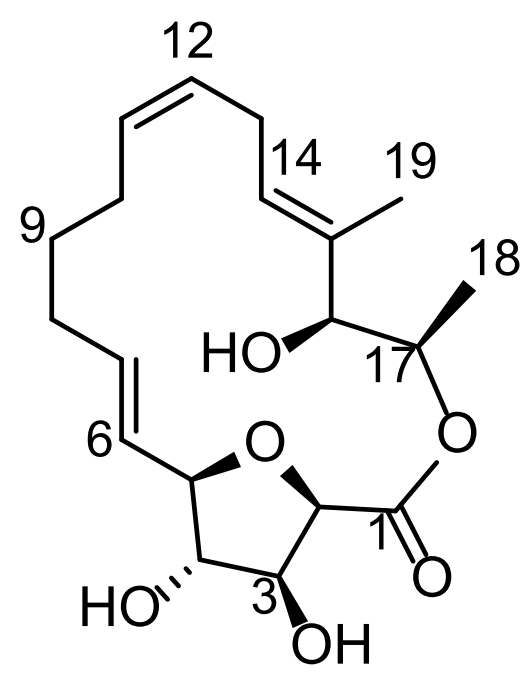

Supplementary Note 3. NMR data assignment of **6** in methanol-*d₄*.

Compound **6** displayed an $[M + Na]^+$ peak at *m/z* 375.1775 (calcd for $C_{19}H_{28}O_6Na$, 375.1784), consistent with the molecular formula $C_{19}H_{28}O_6$. Comparison of the DEPTQ spectrum (Supplementary Table 8) with those of **5** revealed key differences: the absence of one set of olefinic carbon signals and one oxygenated methine signal, accompanied by the emergence of a new carbonyl carbon resonance. The saturation of a double bond was confirmed by the COSY correlations, which established a continuous spin system of three methylene units from H_2-11 to H_2-14. The location of the newly formed carbonyl group was definitively assigned to C-10 based on key HMBC correlations from H_2-12, H_2-13, H_2-11, and H_2-9 to C-10, the carbonyl carbon at δ_C 213.9. An additional structural variation at C-16, which was present as a methylene group rather than a hydroxylated methine, was corroborated by HMBC correlations (Supplementary Figure 26) from H_3-18 and H_3-19 to C-16, and from H_2-16 to C-14. On the basis of this spectroscopic evidence, compound **6** was characterized as a keto derivative of **5**, featuring a carbonyl group at C-10 and a methylene group at C-16. The configurations of the remaining double bonds were determined from coupling constants and NOE data. The large coupling constant of 15.6 Hz between H-6 and H-7 supported an *E* configuration for $\Delta^{6,7}$. For $\Delta^{14,15}$, an *E* geometry was assigned based on NOE correlations between H_2-13 and H_3-19, as well as between H-14 and H-16. The absolute configuration (2*R*, 3*S*, 4*S*, 5*R*, 16*S*, 17*R*) was assigned by analogy with compounds **3–5**, as supported by the similar coupling constants, together with the shared biosynthetic origin of the *arc* PKS assembly line.

Supplementary Table 8

Pos.	δ_C, type	δ_H, mult. (*J* in Hz)
1	172.3, C	–
2	81.8, CH	4.46, d (7.2)
3	78.2 CH	4.30, t (7.2)
4	80.6, CH	3.98, t (7.2)
5	86.0, CH	4.10, t (7.8)
6	132.8, CH	5.52, dd (15.6, 7.8)
7	135.0, CH	5.62, ddd (15.6, 9.6, 4.2)
8	31.0, CH_2	2.08, m; 2.16, m
9	22.4, CH	1.49, m; 1.96, m
10	213.9, C	–
11	39.5, CH_2	2.31, m; 2.67, m
12	43.9, CH	2.49, m
13	22.9, CH_2	2.23, m; 2.33, m
14	127.8, CH	5.16, m
15	132.2, C	–
16	47.4, CH_2	2.27, m
17	70.6, CH	5.17, m
18	21.3, CH_3	1.24, d (6.0)
19	15.9, CH_3	1.61, s

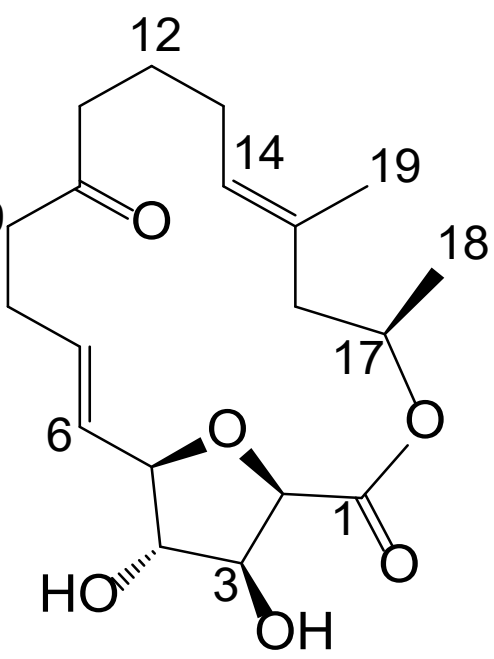

Supplementary Note 4. NMR data assignment of **8** in methanol-*d₄*.

Compound **8** was obtained as a weakly yellow oil with the molecular formula $C_{11}H_{18}O_3$ established by HRESIMS data from $[M + H - H_2O]^+$ peak at *m/z* 181.1227 (calcd. 181.1223 for $C_{11}H_{17}O_2$), representing the existence of three unsaturation levels. ^{1}H NMR (600 MHz, methanol- d_4) and DEPTQ NMR (150 MHz, methanol-d_4) data was shown in Supplementary Table 9. The combination of ^{1}H NMR and HSQC spectra of **8** exhibited the feature of one carbonyl (δ_C 171.5), four oleffnic carbons ranging from δ_C 129.4 to δ_C 140.2, one oxygenated methine (δ_C 77.3), two methylenes (δ_C 35.4, δ_C 21.6) and two vinylic methyls (δ_C 12.5, δ_C 11.1), and one aliphatic methyl (δ_C 14.3). The planar structure of **8** was connected through the extensive elucidation of HMBC and COSY spectra (Supplementary Figure 26). The continuous spin system between H-3−H-5 and H-7−H-9 in the COSY spectrum proved the linear main skeleton of **8**. A set of key HMBC correlations have been observed between the carbonyl C-1 (δ_C 171.5) and H-3, oxygenated methine C-5 (δ_C 77.3) and H-4 (δ_H 2.43), H-7 (δ_H 5.41), successfully complete the final linkage of these two spin systems.The *R* configuration of the hydroxyl-bearing stereocenter at C-5 was assigned via comparison of the experimental ECD spectrum with DFT-calculated spectrum at the B3LYP/TZVP level (Supplementary Figure 27).

Supplementary Table 9

Pos.	δ_C, type	δ_H, mult. (*J* in Hz)
1	171.5, C	–
2	129.8, C	–
3	140.2, CH	6.76, td (7.2, 1.2)
4	35.4, CH_2	2.43, m
5	77.3, CH	4.06, t (7.2)
6	136.9, C	–
7	129.4, CH	5.41, t (7.2)
8	21.6, CH_2	2.05, m
9	14.3, CH_3	0.97, t (7.8)
10	12.5, CH_3	1.81, d (1.2)
11	11.1, CH_3	1.62, s

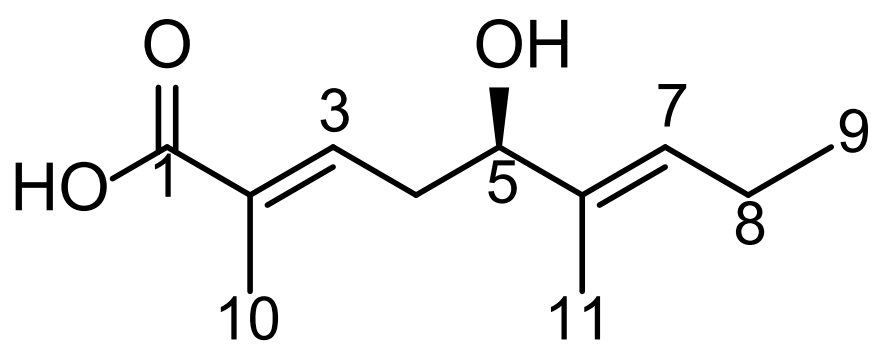

Supplementary Note 5. NMR data assignment of **9** in methanol-*d4*.

Compound **9** was also isolated as a weakly yellow oil with the molecular formula $C_{11}H_{18}O_3$ established by HRESIMS $[M + H - H_2O]^+$ peak at *m/z* 181.1227 (calcd. for $C_{11}H_{17}O_2$ 181.1223), indicating three unsaturation levels. ^{1}H NMR and DEPTQ NMR data (Supplementary Table 10) in combination with HSQC spectrum of **9** indicated the presence of one carbonyl (δ_C 176.3), four oleffnic carbons ranging from δ_C 126.3 to δ_C 139.2, one oxygenated methine (δ_C 74.8), two methylenes (δ_C 42.3, δ_C 23.5) and two vinylic methyls (δ_C 13.4, δ_C 23.8), and one aliphatic methyl (δ_C 14.3). The planar structure of **9** was closely similar to those of **8**, and the distinction lies in the different positions of hydroxyl and double bonds. The locations of substituents were confirmed through the continuous spin system between H-7−H-9 in the COSY spectrum and HMBC correlations (Supplementary Figure 26) between C-5(δ_C 126.3) and H-3 (δ_H 4.46), H-7 (δ_H 5.24), H-10 (δ_H 1.61) and H-11(δ_H 1.72). The absolute configuration at C-3 was assigned as *R*, based on a good match between the experimental ECD spectrum and the DFT-calculated spectrum for the (3*R*)-enantiomer (Supplementary Figure 27).

Supplementary Table 10

Pos.	δ_C, type	δ_H, mult. (*J* in Hz)
1	176.3, C	–
2	42.3, CH_2	2.50, d (6.6)
3	74.8, CH	4.46, t (6.6)
4	139.2, C	–
5	126.3, CH	5.90, s
6	132.7, C	–
7	130.7, CH	5.24, t (7.2)
8	23.5, CH_2	1.91, m
9	14.3, CH_3	0.92, t (7.2)
10	13.4, CH_3	1.61, d (1.2)
11	23.8, CH_3	1.72, s

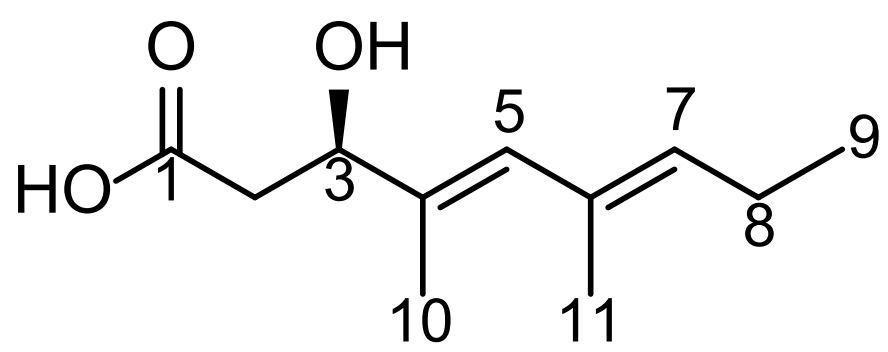

Supplementary Note 6. NMR data assignment of **10** in DMSO-d_6.

Compound **10** was isolated as a dark yellow oil, which formed a fluorescent yellow solution characteristic of anthraquinones. High-resolution mass spectrometry (HRESIMS) established its molecular formula as $C_{16}H_{11}NO_5$ based on a sodium adduct ion $[M + Na]^+$ at *m/z* 320.0537 (calcd. for $C_{16}H_{11}NO_5Na$, *m/z* 320.0535), consistent with a molecule containing one nitrogen atom. Due to its limited solubility in methanol, NMR spectra for **10** were acquired in DMSO-d_6 (Supplementary Table 11). The ^1H and DEPTQ spectra revealed signals consistent with a substituted anthraquinone framework. Specifically, the DEPTQ spectrum showed nine quaternary carbons, including three downfield signals indicative of carbonyl or amide groups (δ_C 191.7, 181.6, and 170.8). The ^1H NMR spectrum displayed signals for two exchangeable protons, corresponding to two phenolic hydroxyl groups on the aromatic rings. A detailed comparison of the 1D NMR data with that of the known anthraquinone, rhein, confirmed that **10** possessed the same core skeleton but was appended with an additional methylene group (δ_H 3.56, δ_C 42.3) and an amide group C-16 (δ_C 170.8). The precise location of this carboxymethyl substituent was unambiguously established by key HMBC correlations. Long-range correlations were observed from the methylene protons (H_2-15, δ_H 3.56) to C-1 (δ_C 147.1), C-2 (δ_C 120.6), C-14 (δ_C 124.6), and C-16. By integrating all 2D NMR data, including COSY and HMBC correlations (Supplementary Figure 26), the structure of **10** was unequivocally determined to be a new rhein analogue.

Supplementary Table 11

Pos.	δ_C, type	δ_H, mult. (J in Hz)
1	147.1, C	–
2	120.6, CH	7.67, d (1.2)
3	133.1, C	–
4	181.6, C	–
5	133.4, C	–
6	119.4, CH	7.74, d (7.8)
7	137.4, CH	7.82, t (7.8)
8	124.5, CH	7.40, d (8.4)
9	161.4, C	–
10	116.0, C	–
11	191.7, C	–
12	114.5, C	–
13	161.4, C	–
14	124.6, CH	7.29, s
15	42.3, CH_2	3.56, s
16	170.8, C	–

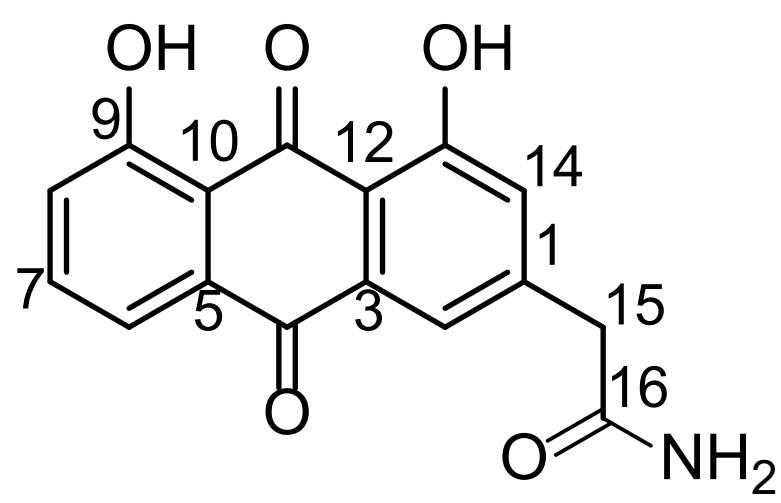

SUPPORTING SPECTRA OF THE ISOLATED COMPOUNDS

Supplementary Figure 28. ^{1}H NMR (600 MHz, methanol-d_4) of **1**.

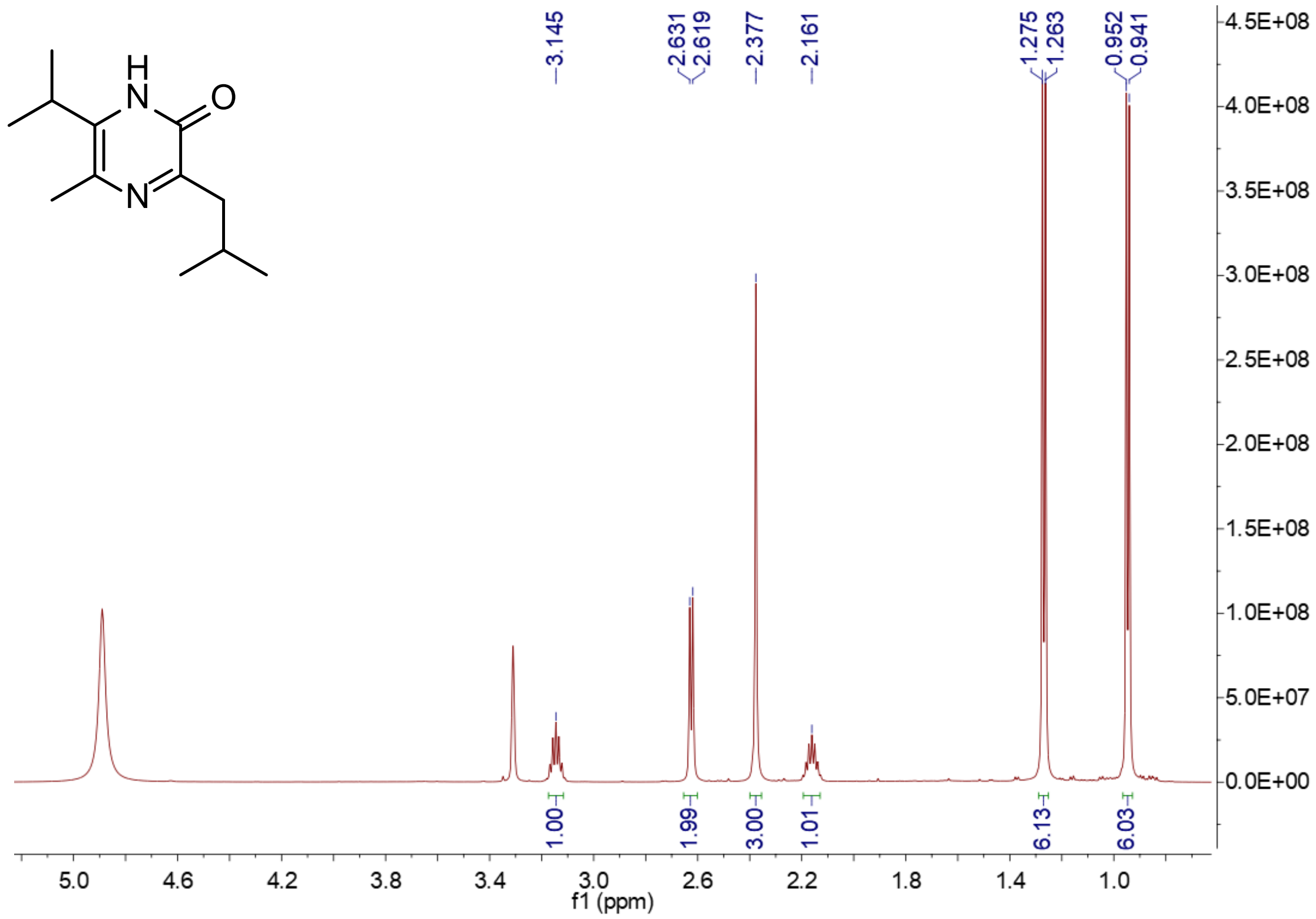

Supplementary Figure 29. ^{1}H NMR (600 MHz, methanol-d_4) of **2**.

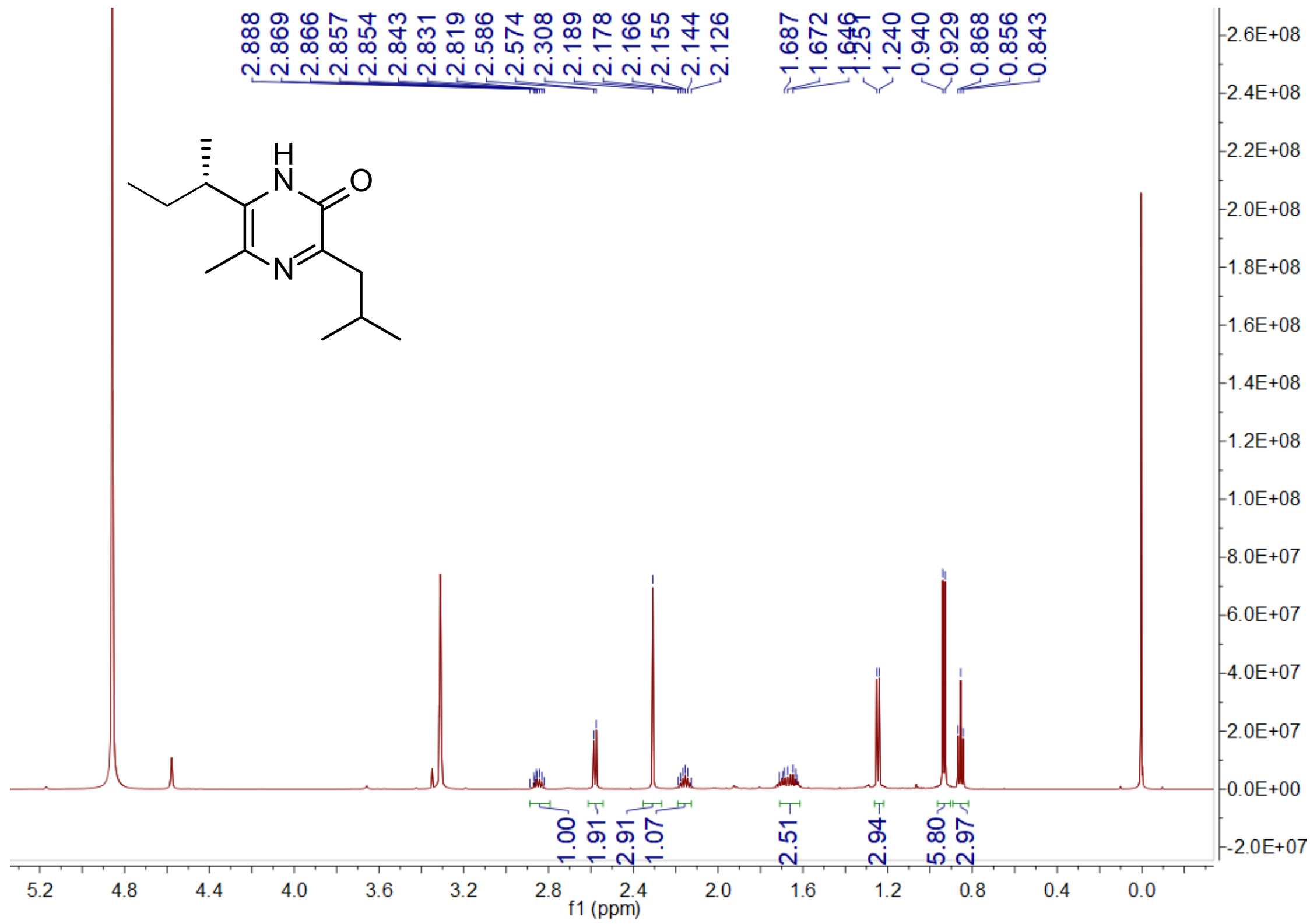

Supplementary Figure 30. ^{1}H NMR (600 MHz, methanol-d_4) of **3**.

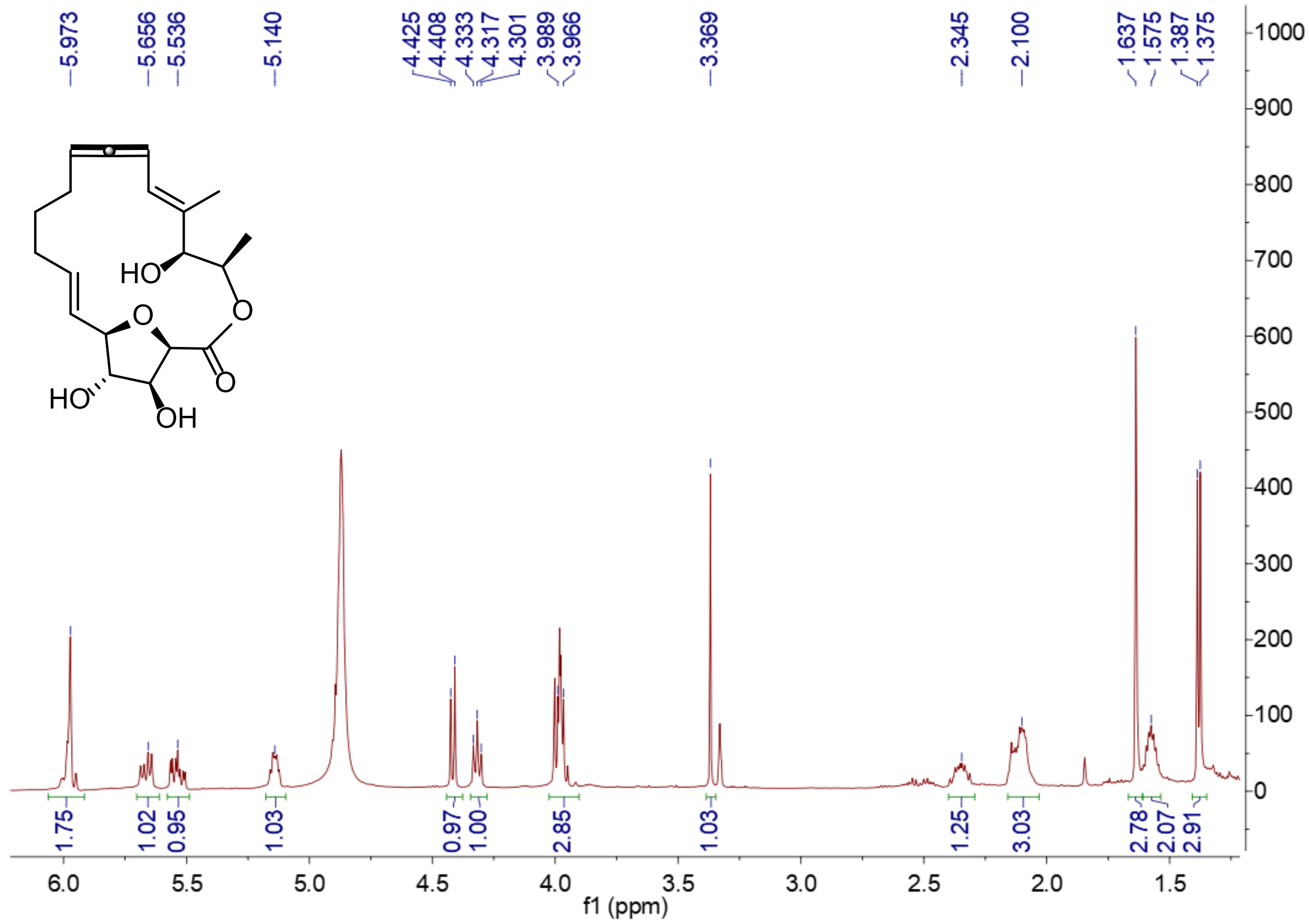

Supplementary Figure 31. ^{13}C NMR (150 MHz, methanol-d_4) of **3**.

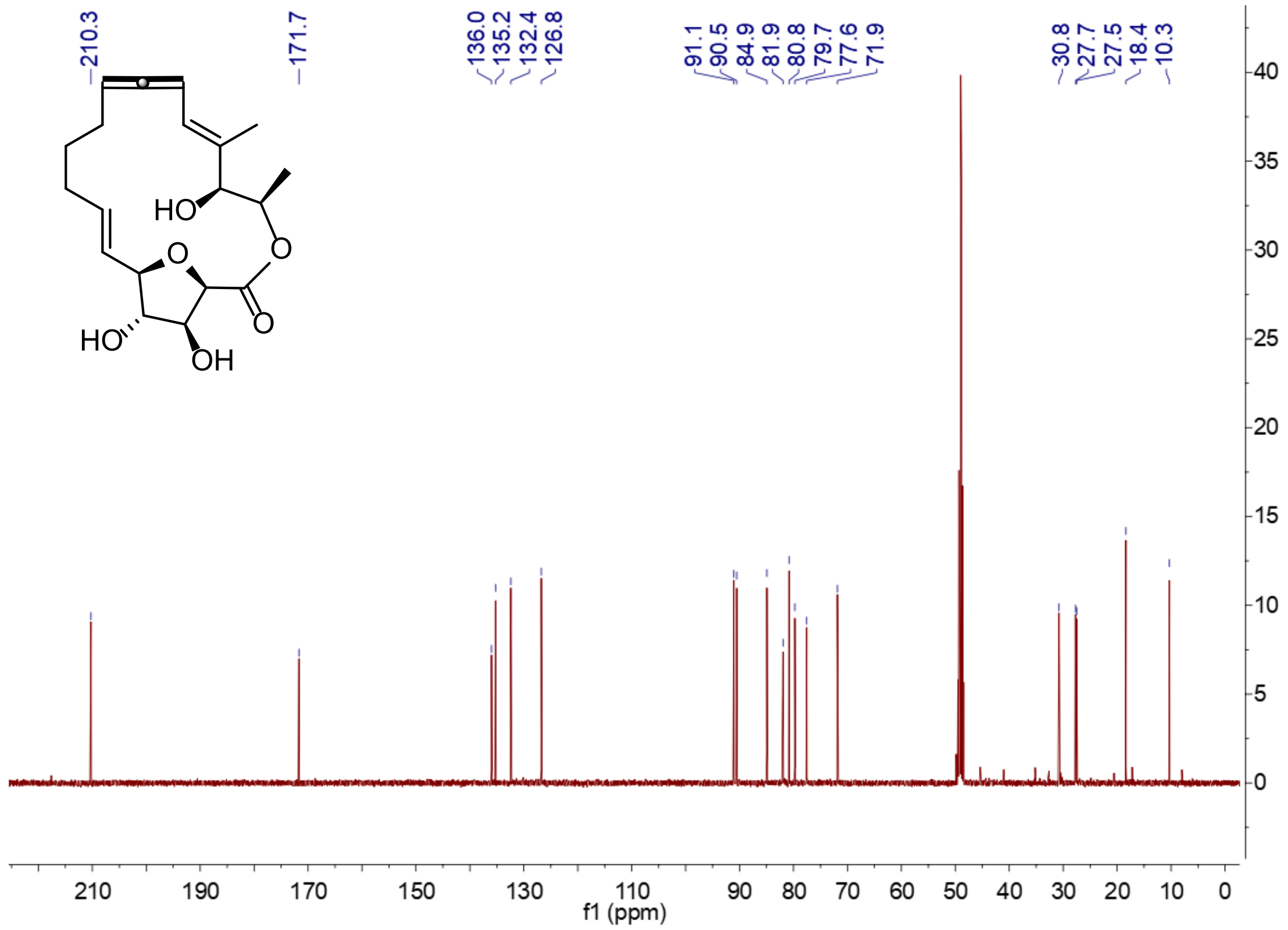

Supplementary Figure 32. ^{1}H-^{13}C HSQC NMR (600 MHz, methanol-d_4) spectrum of **3**.

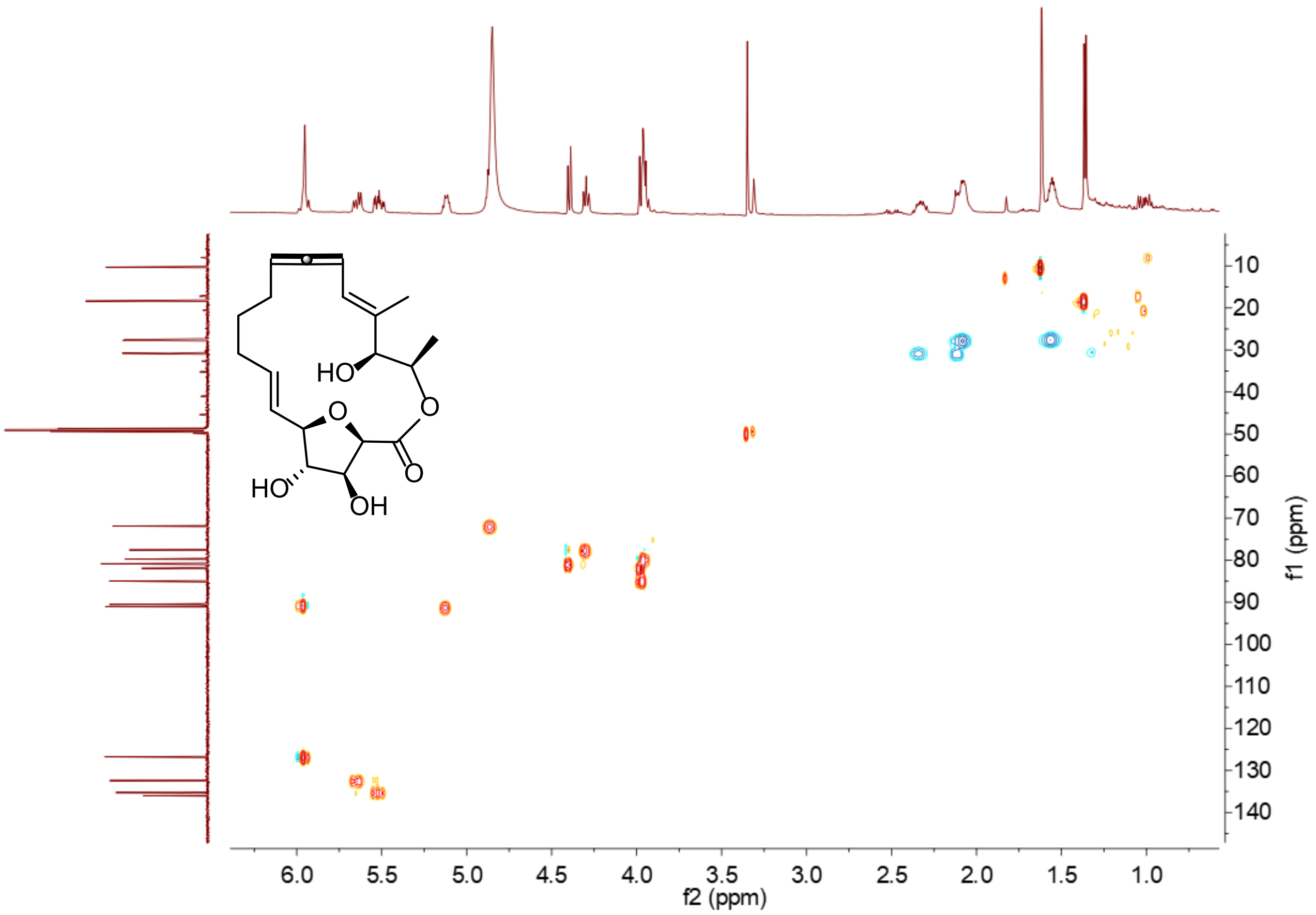

Supplementary Figure 33. ^{1}H-^{13}C HMBC NMR (600 MHz, methanol-d_4) spectrum of **3**.

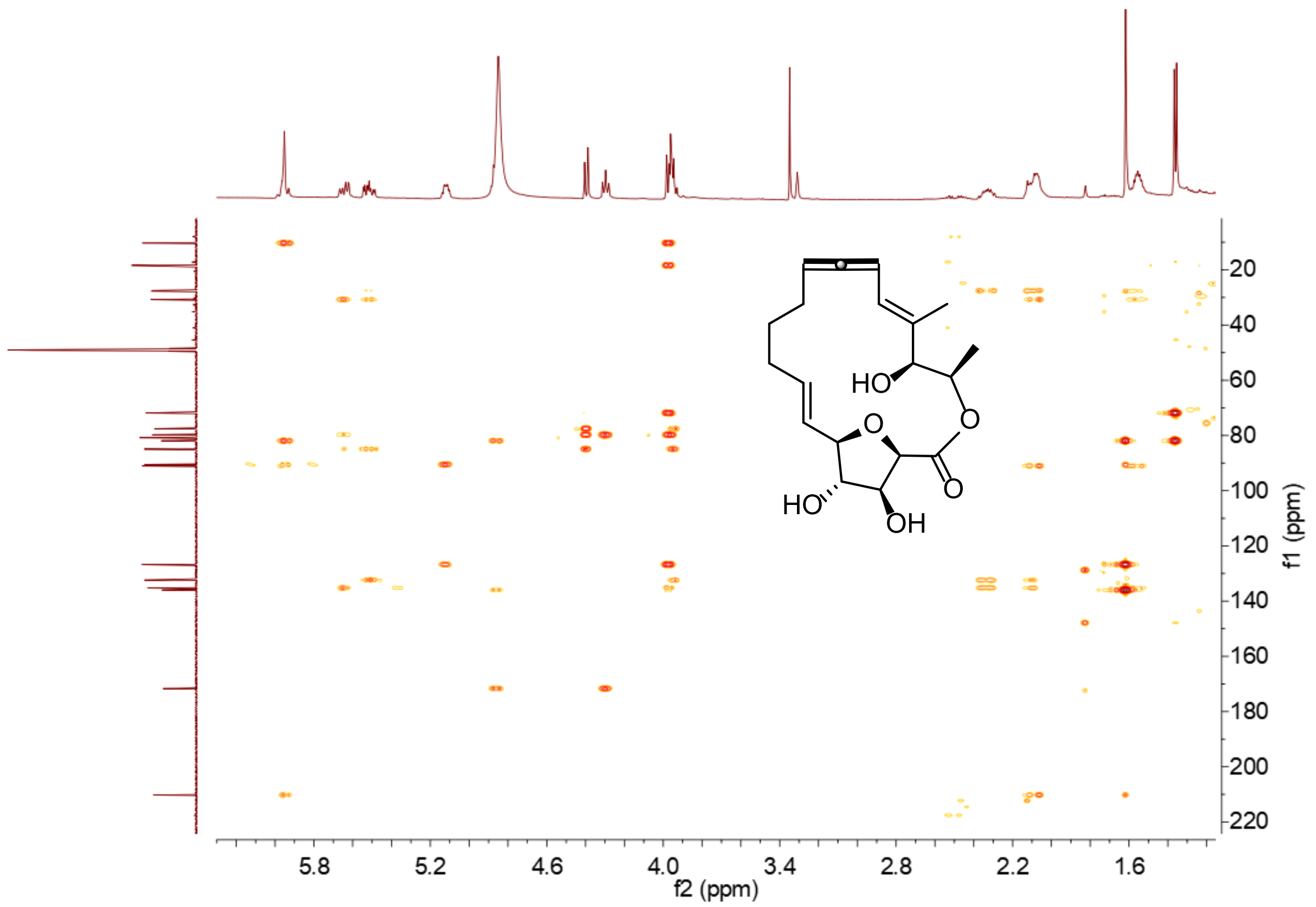

Supplementary Figure 34. ^{1}H-^{1}H COSY NMR (600 MHz, methanol-d_4) spectrum of **3**.

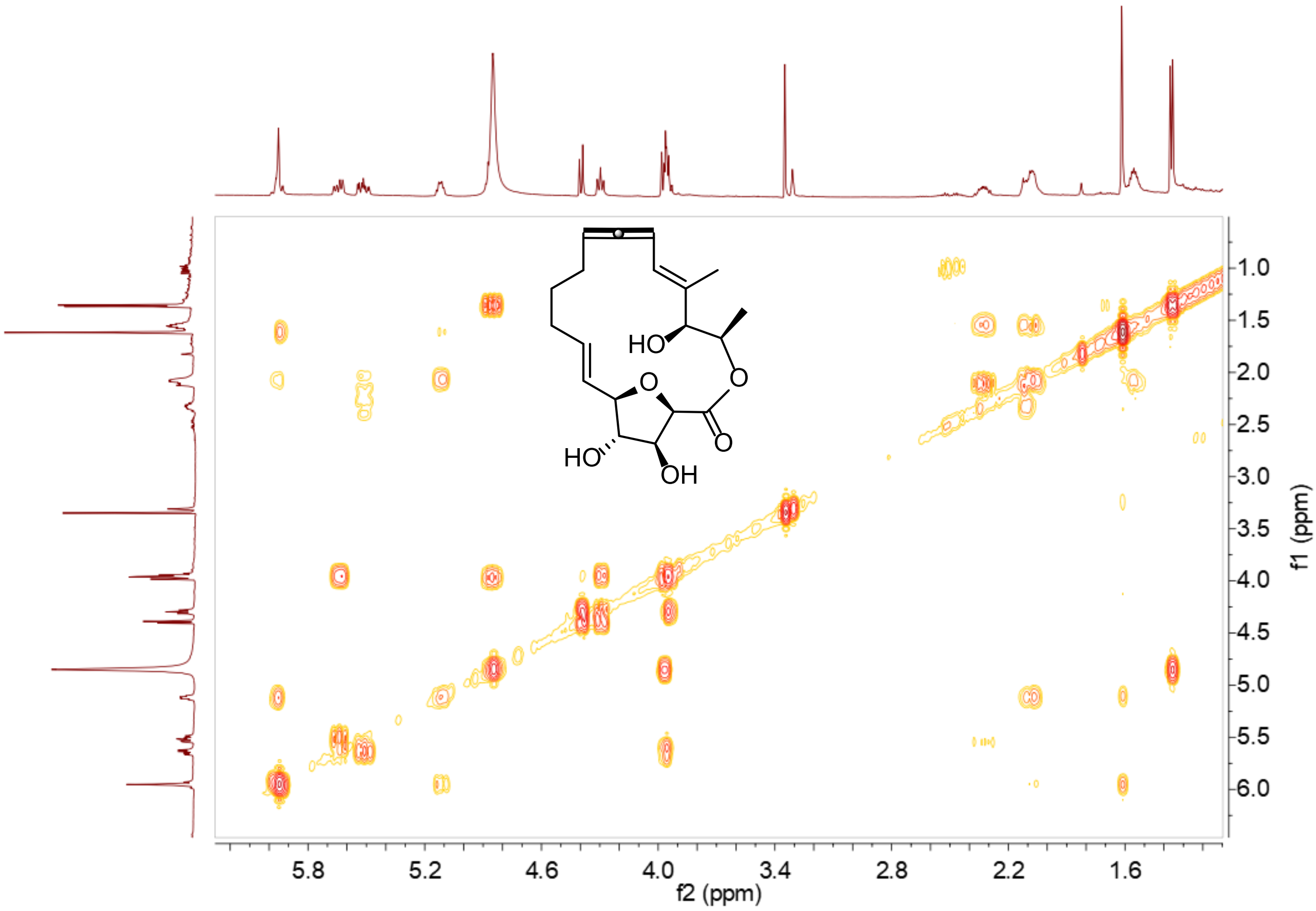

Supplementary Figure 35. ^{1}H NMR (600 MHz, methanol-d_4) of **4**.

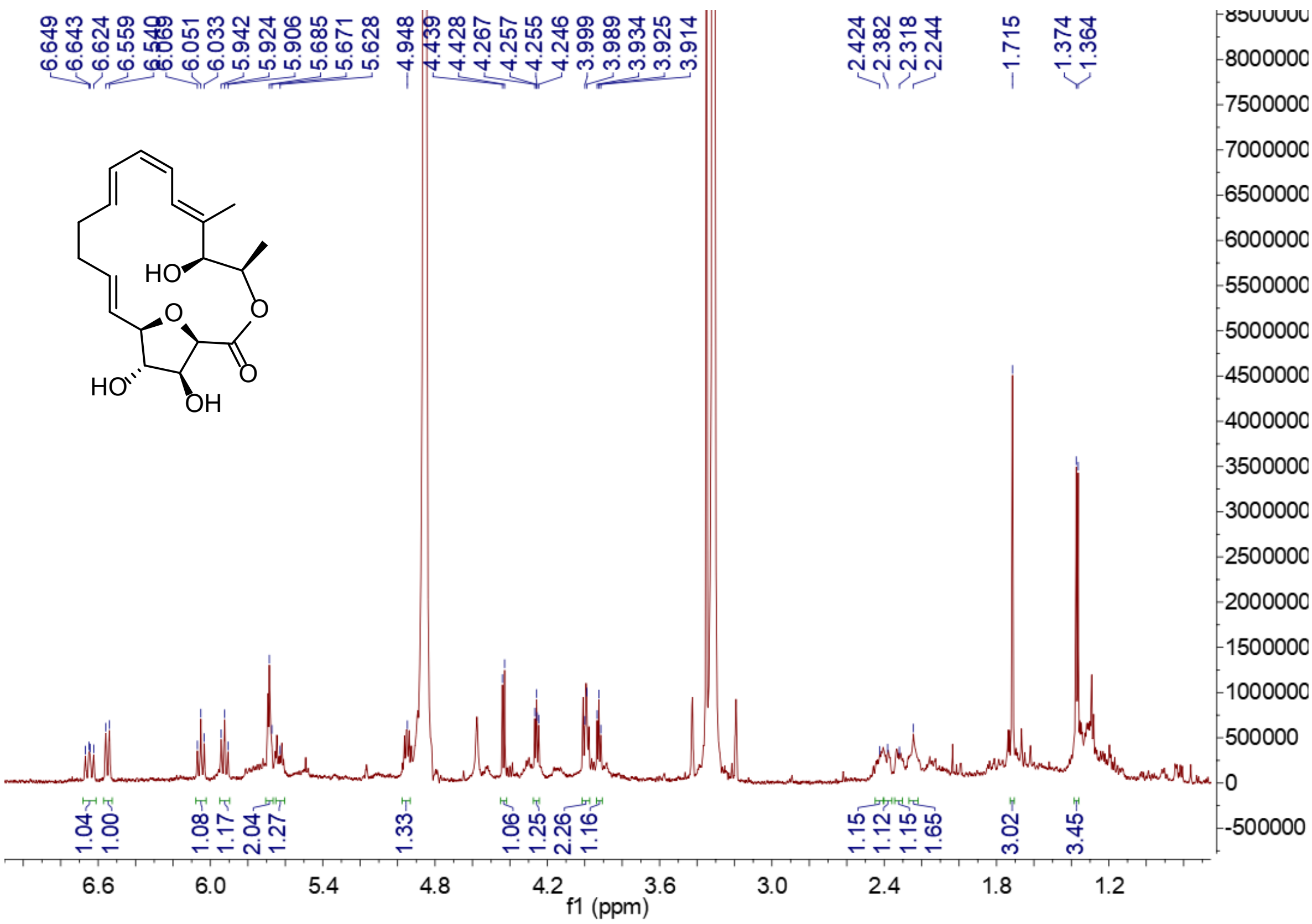

Supplementary Figure 36. DEPTQ NMR (150 MHz, methanol-d_4) of **4**.

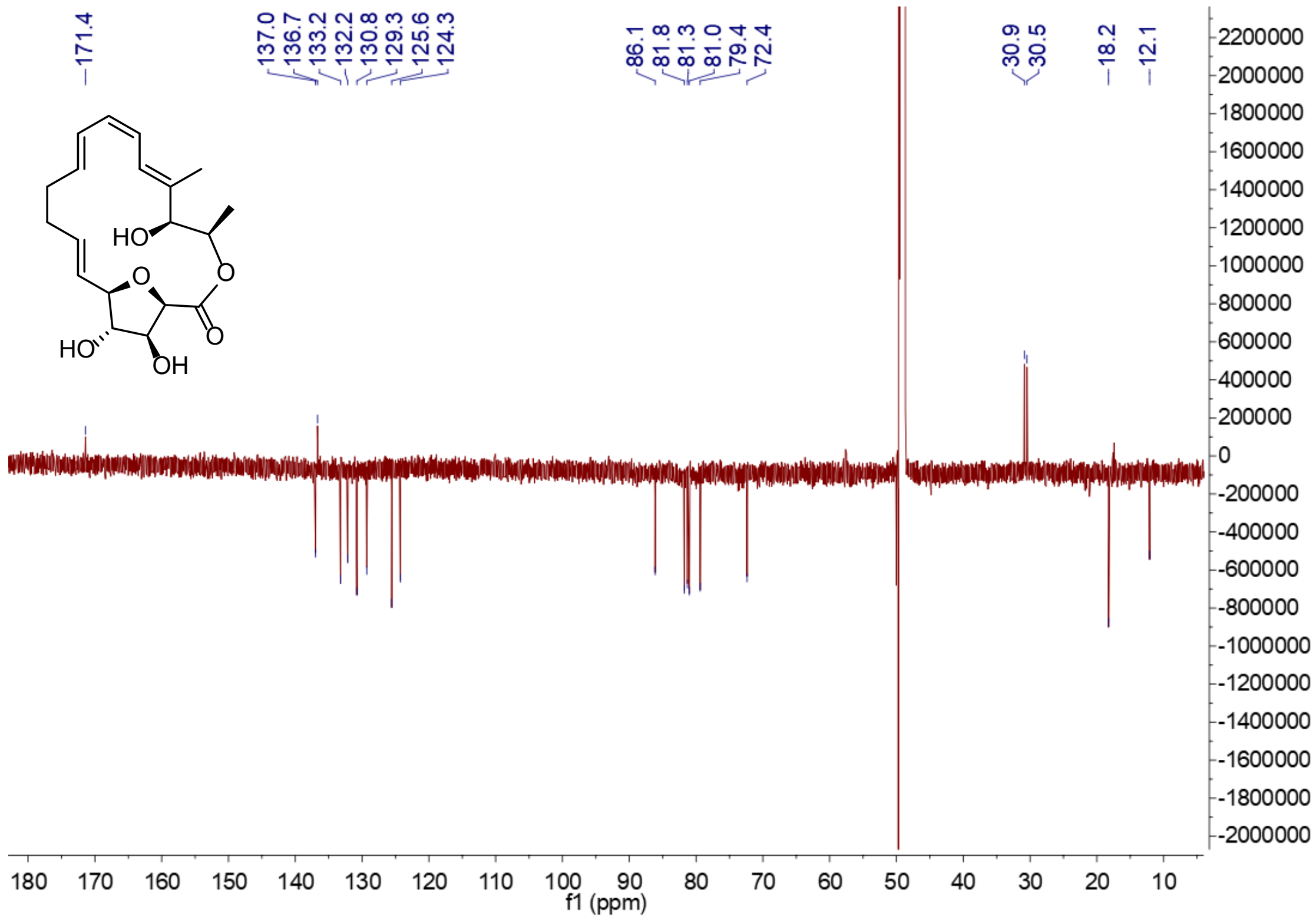

Supplementary Figure 37. ^1H-^{13}C HSQC NMR (600 MHz, methanol-d_4) spectrum of **4**.

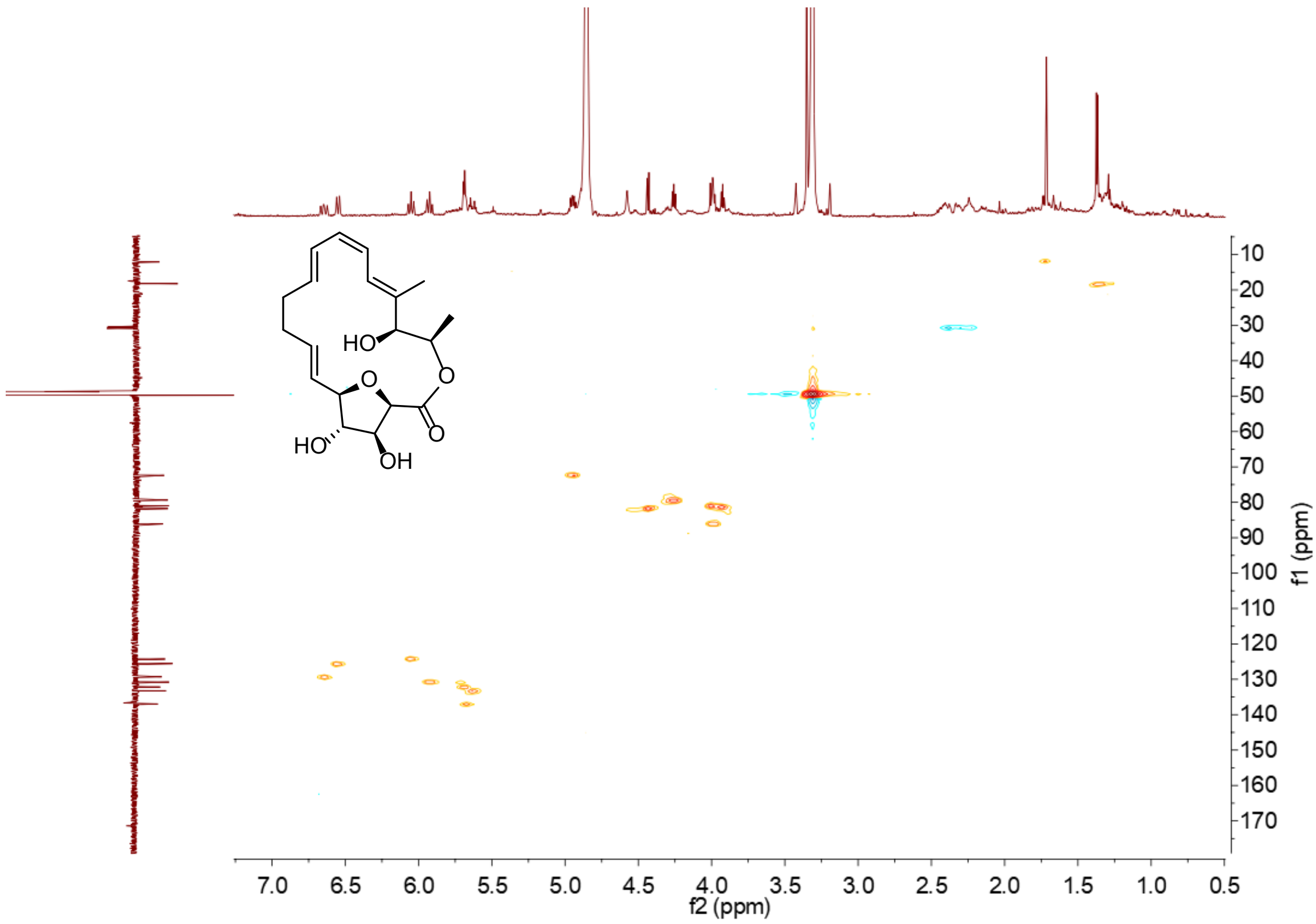

Supplementary Figure 38. ^{1}H-^{13}C HMBC NMR (600 MHz, methanol-d_4) spectrum of **4**.

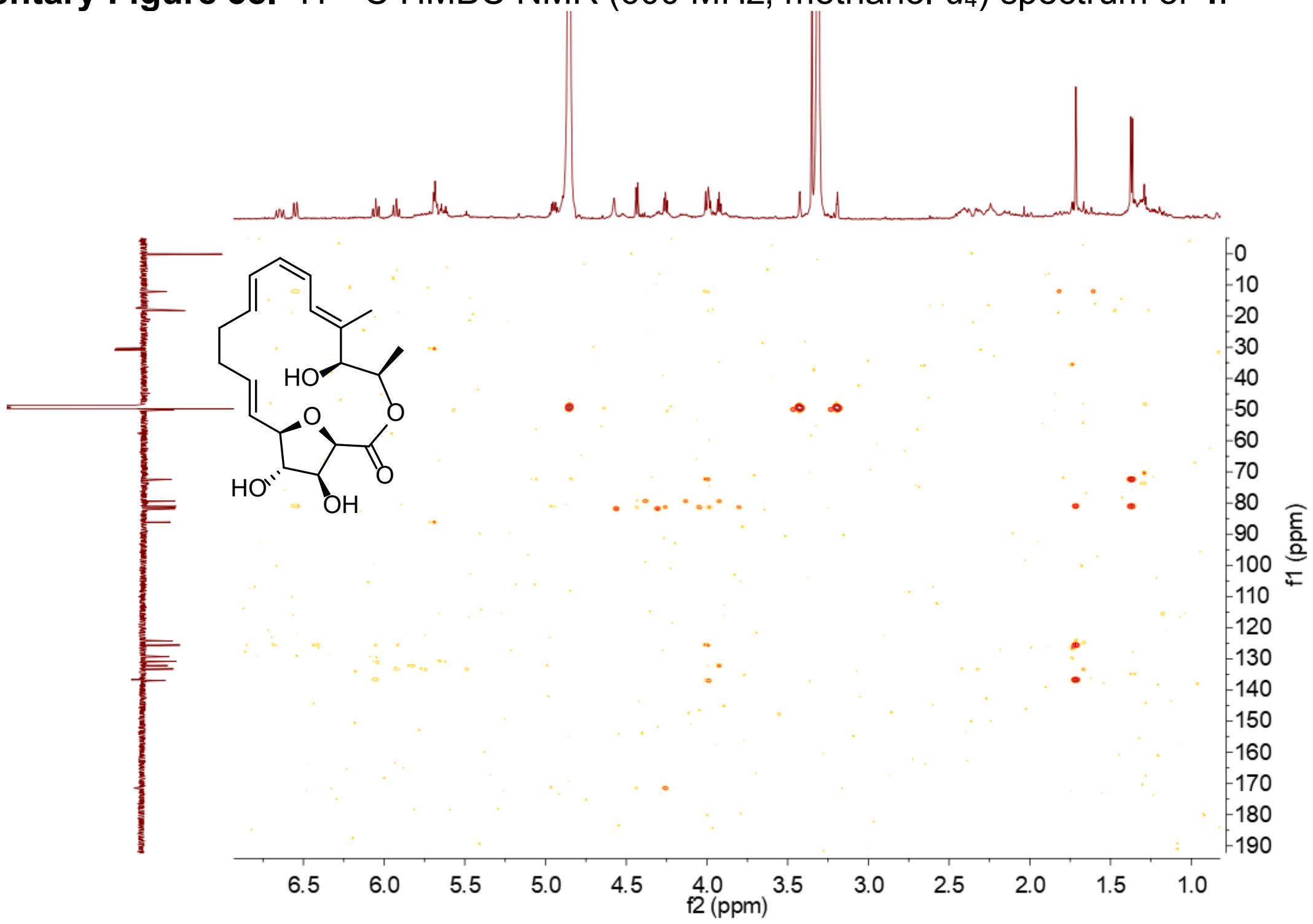

Supplementary Figure 39. ^{1}H-^{1}H COSY NMR (600 MHz, methanol-d_4) spectrum of **4**.

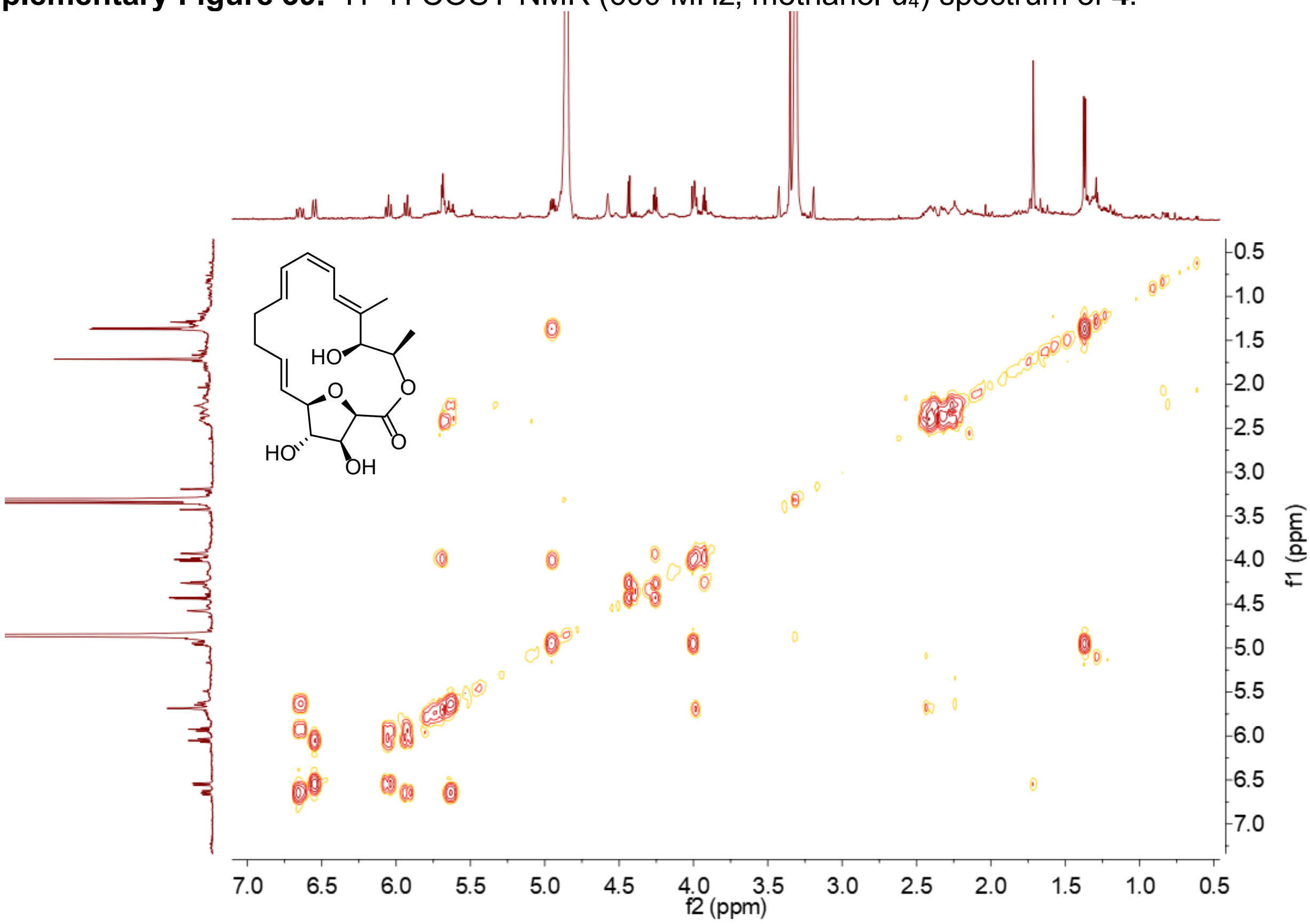

Supplementary Figure 40. HRESIMS spectrum of **4**.

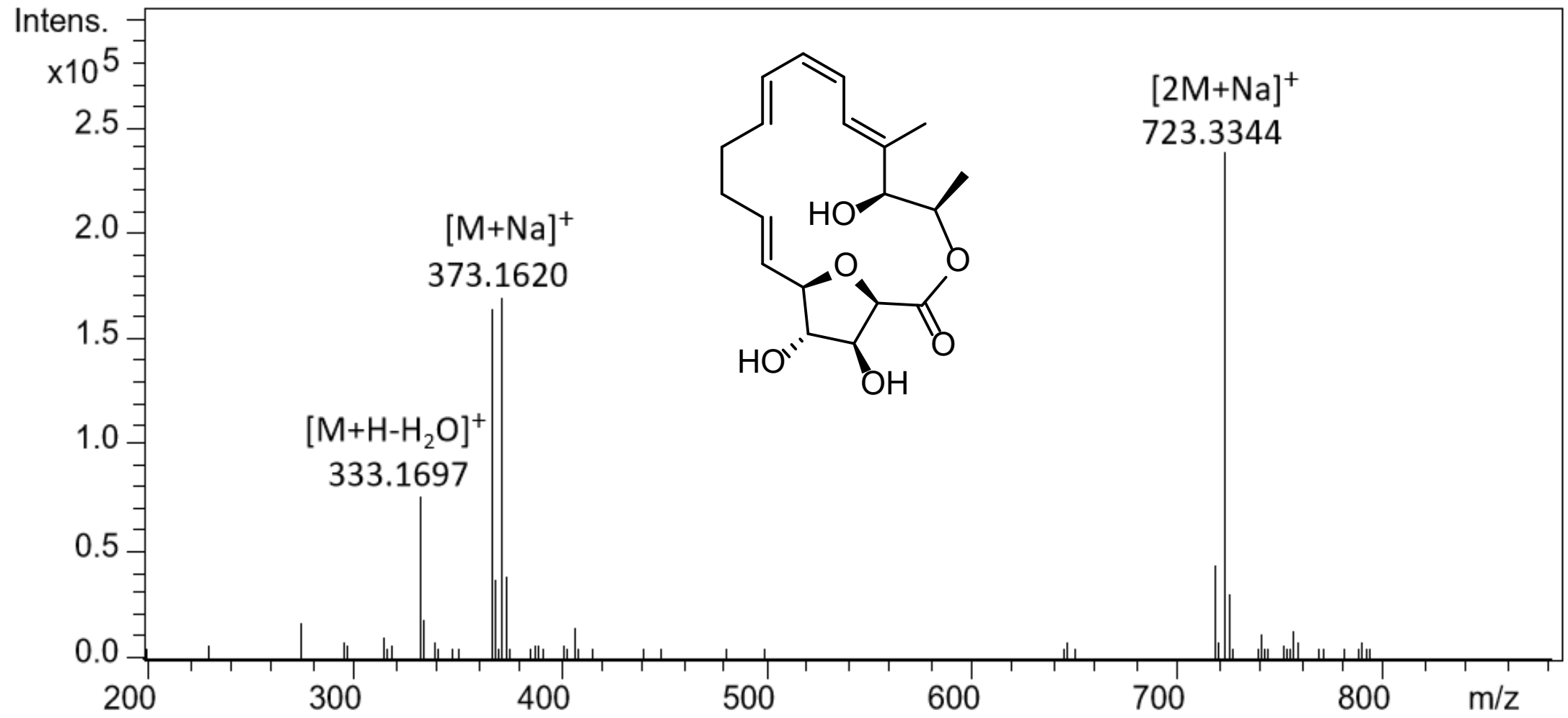

Supplementary Figure 41. ^{1}H NMR (600 MHz, methanol-d_4) of **5**.

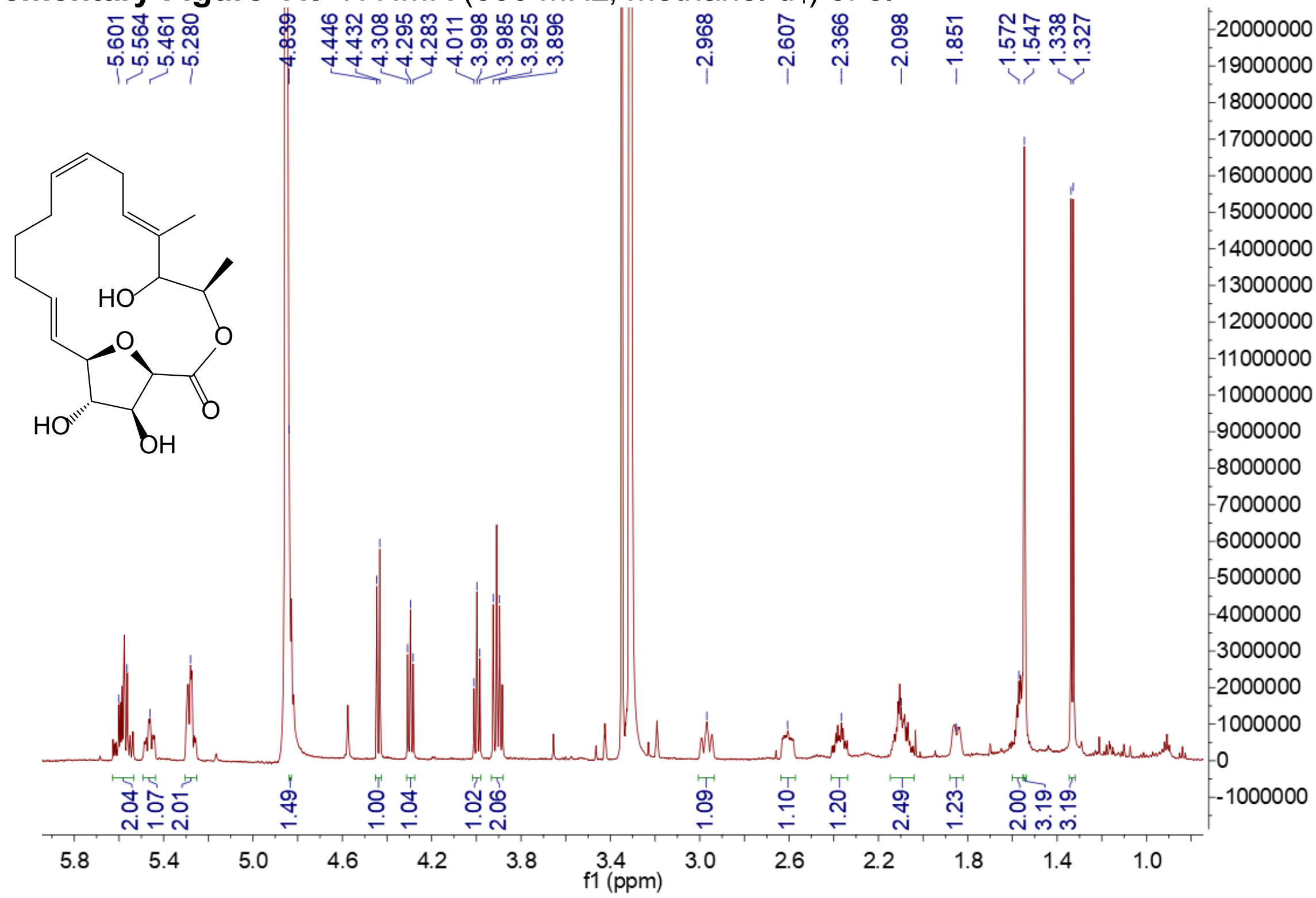

Supplementary Figure 42. ^{1}H NMR (600 MHz, pyridine-d_5+D_2O) of **5**.

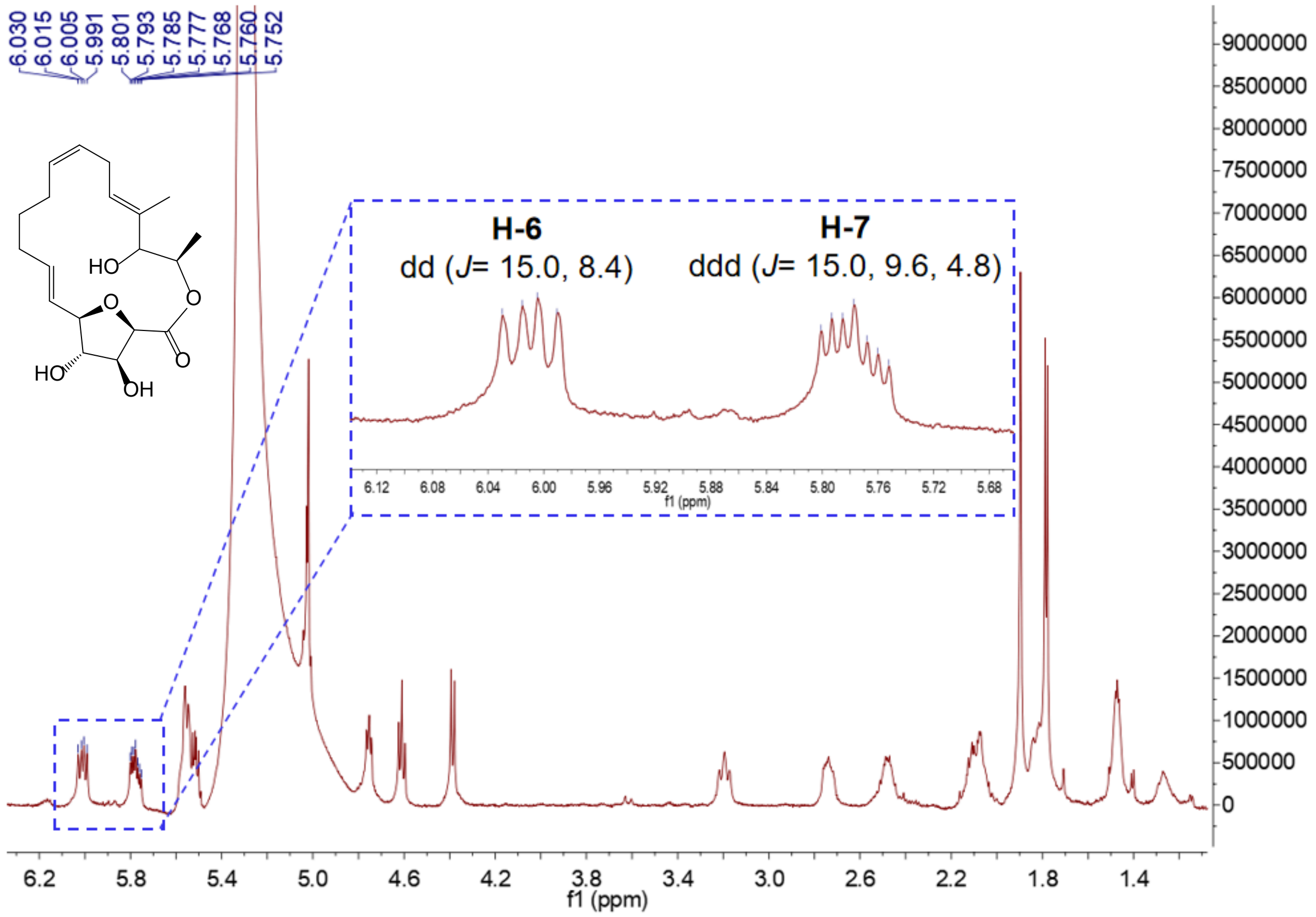

Supplementary Figure 43. DEPTQ NMR (150 MHz, methanol-d_4) of **5**.

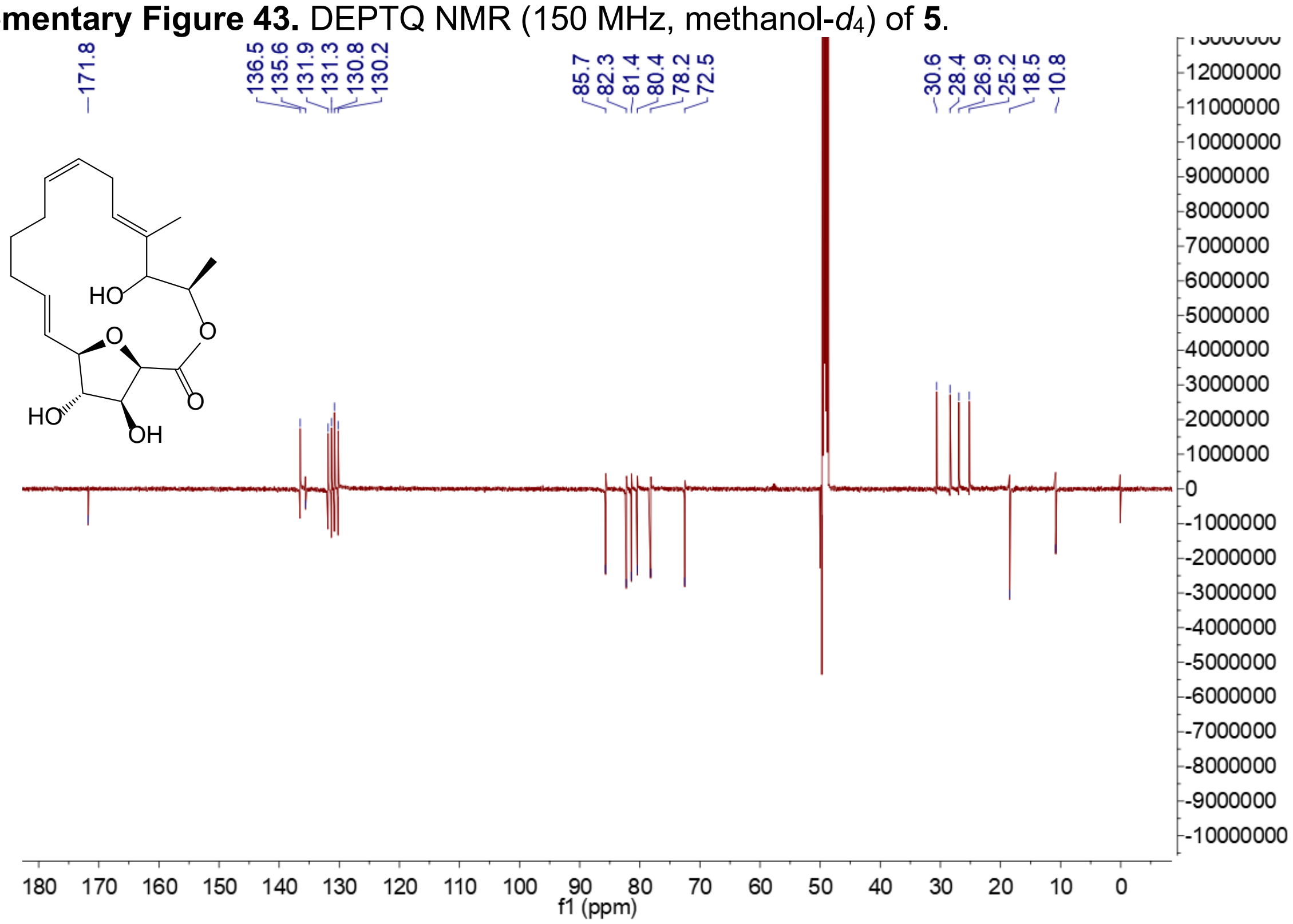

Supplementary Figure 44. ^{1}H-^{13}C HSQC NMR (600 MHz, methanol-d_4) spectrum of **5**.

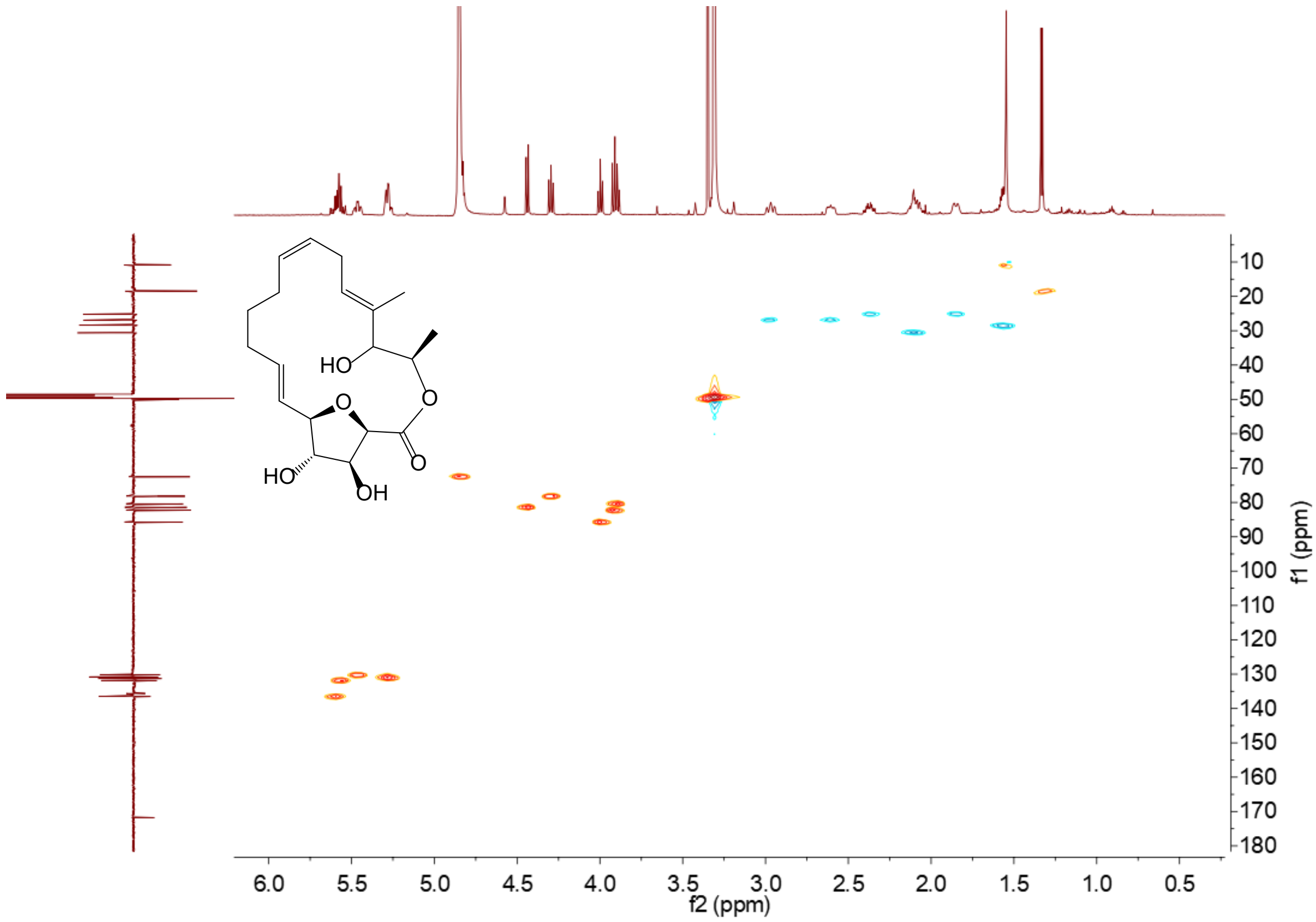

Supplementary Figure 45. ^{1}H-^{13}C HMBC NMR (600 MHz, methanol-d_4) spectrum of **5**.

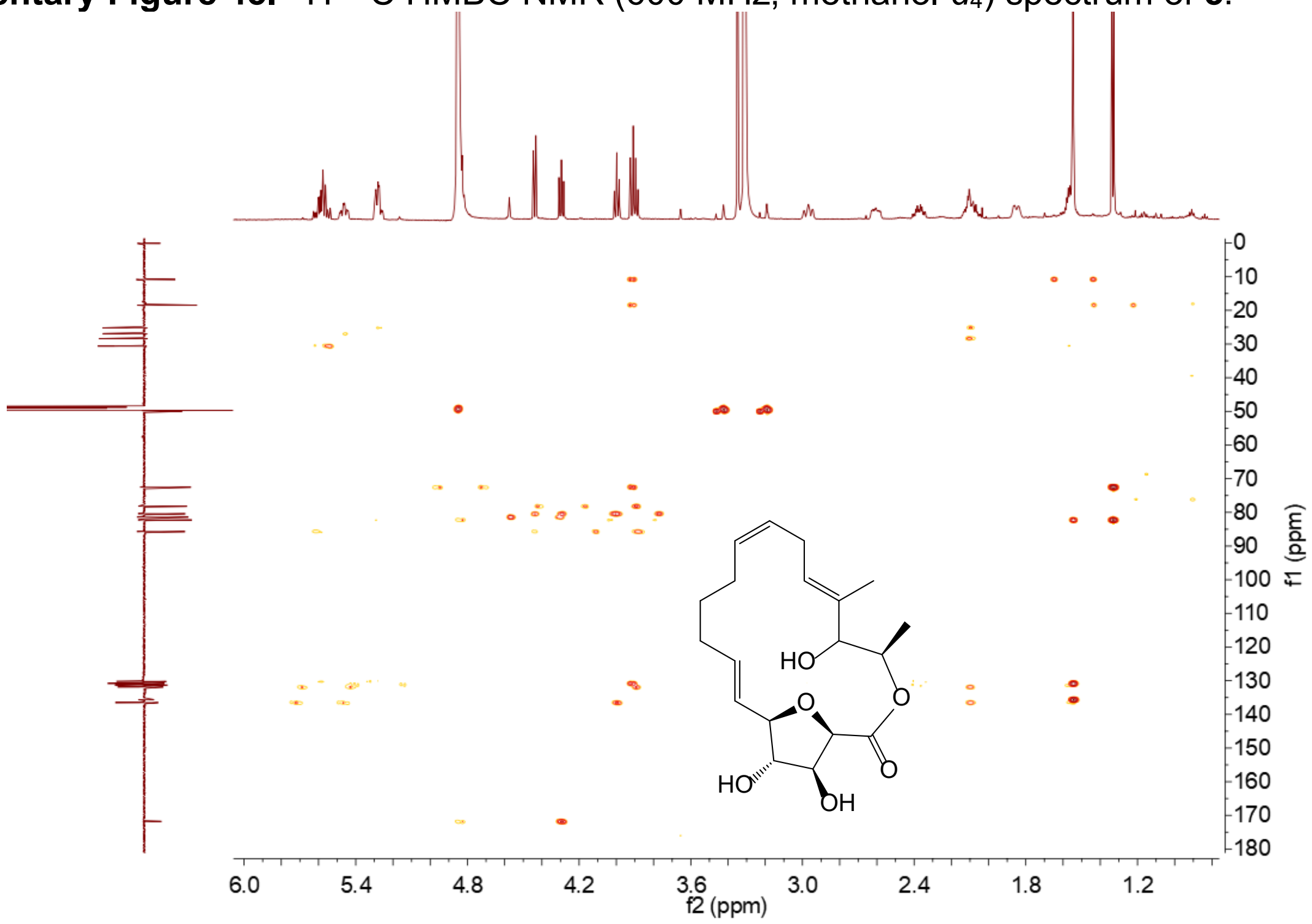

Supplementary Figure 46. ^{1}H-^{1}H COSY NMR (600 MHz, methanol-d_4) spectrum of **5**.

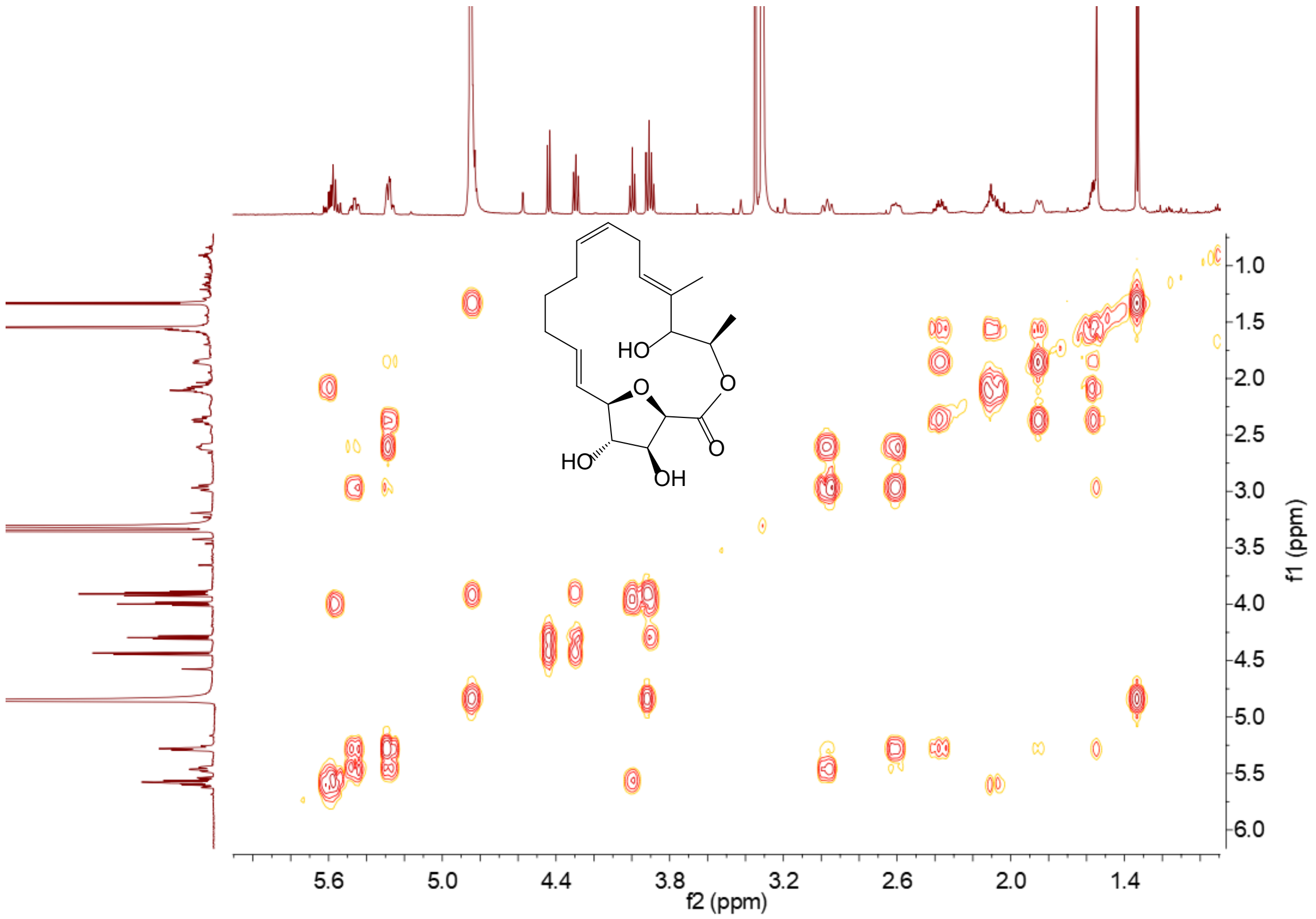

Supplementary Figure 47. ^{1}H-^{1}H NOESY NMR (600 MHz, methanol-d_4) spectrum of **5**.

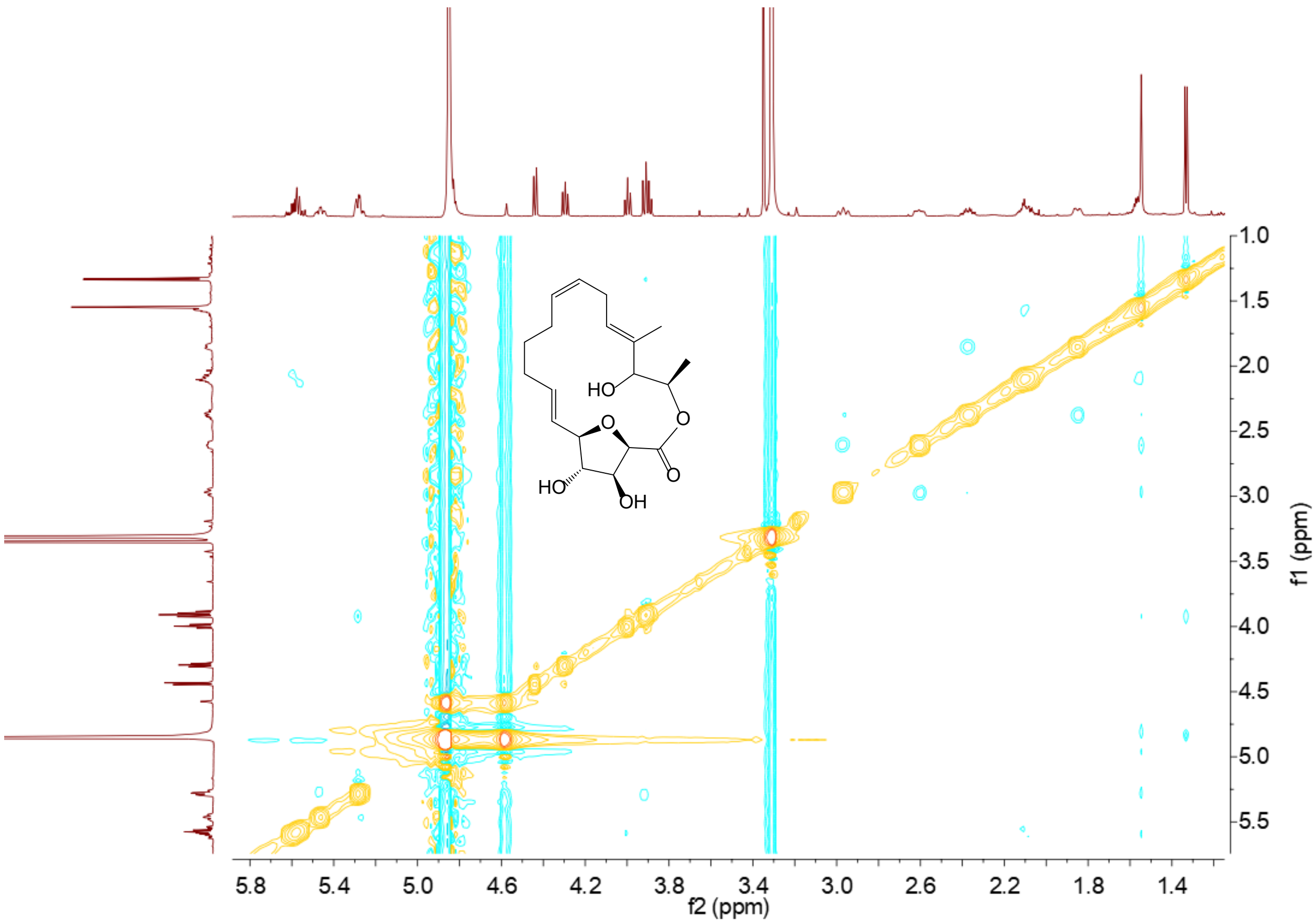

Supplementary Figure 48. HRESIMS spectrum of **5**.

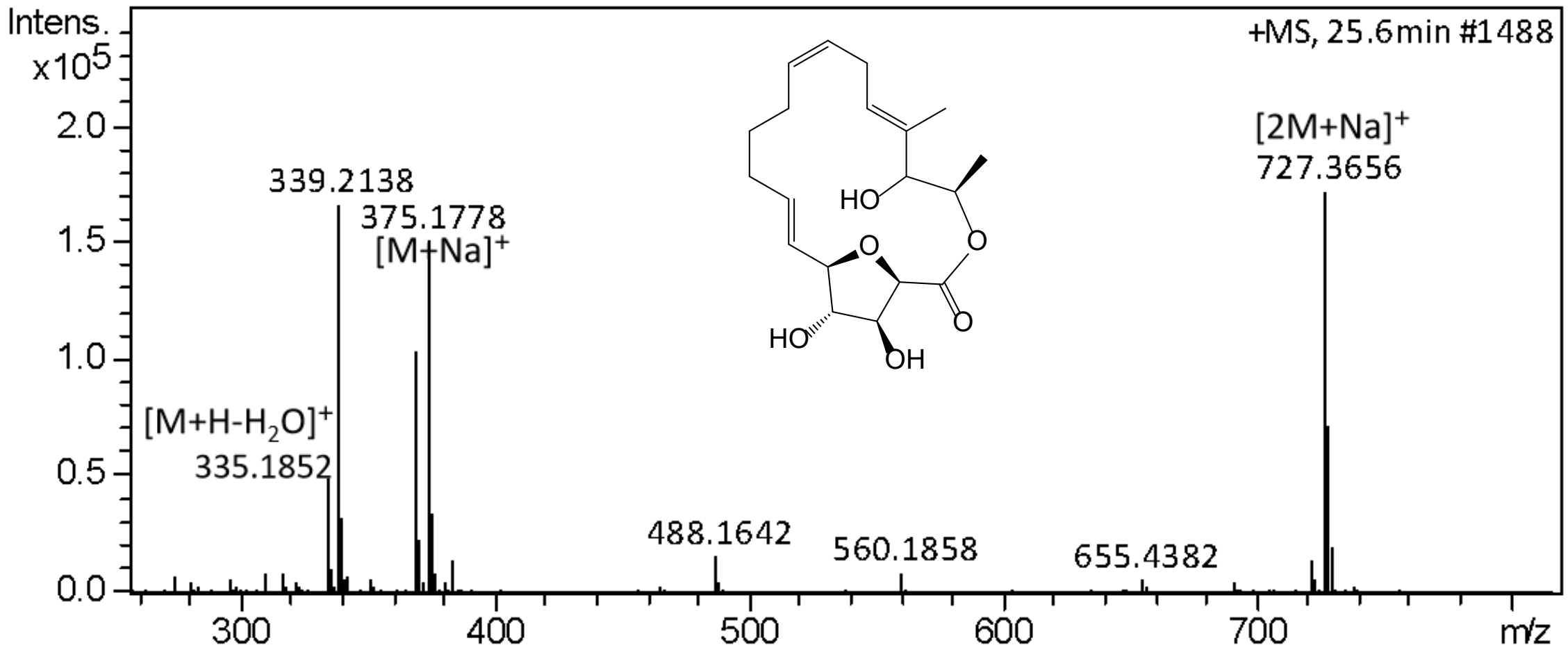

Supplementary Figure 49. ^{1}H NMR (600 MHz, methanol-d_4) of **6**.

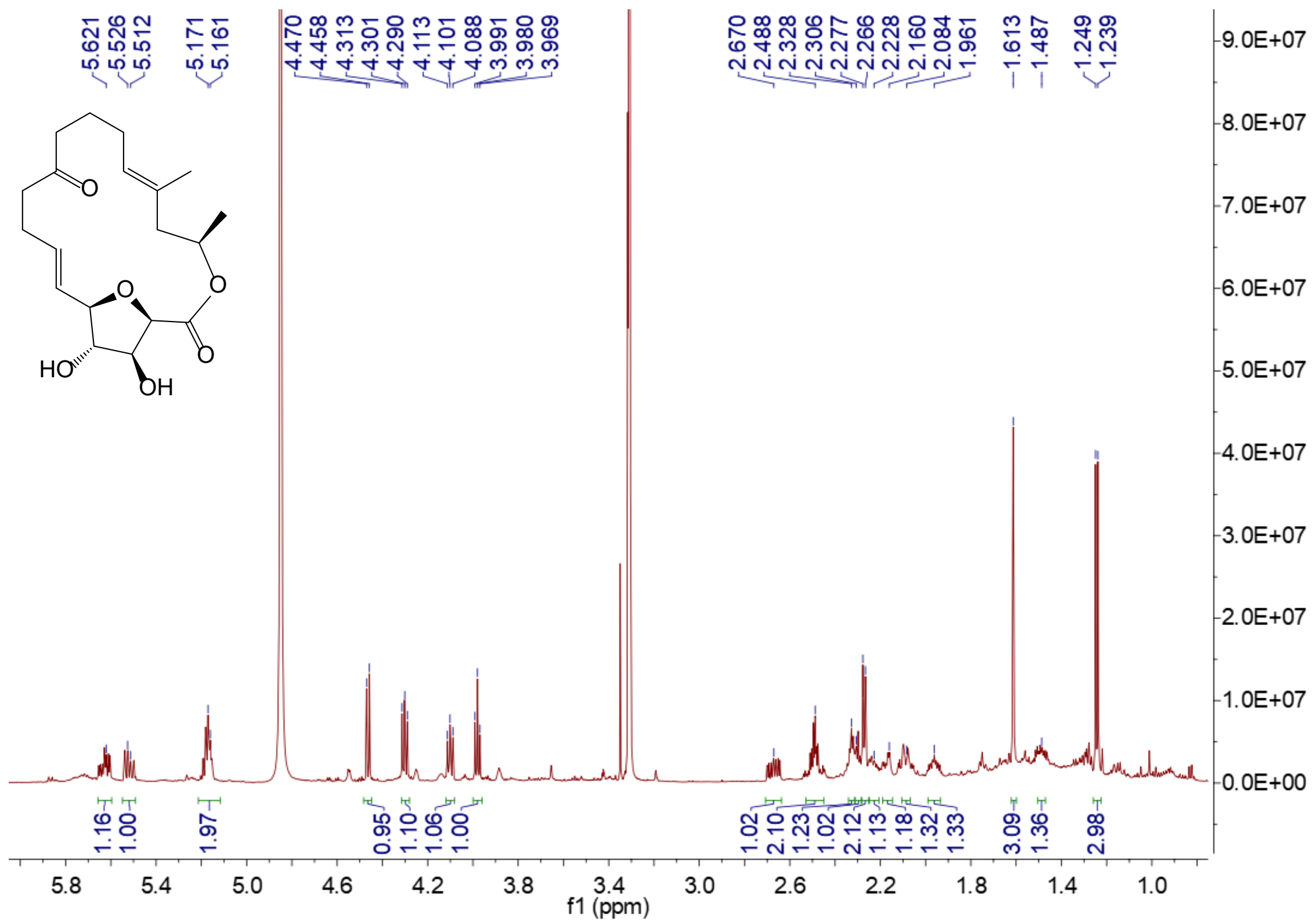

Supplementary Figure 50. DEPTQ NMR (150 MHz, methanol-d_4) of **6**.

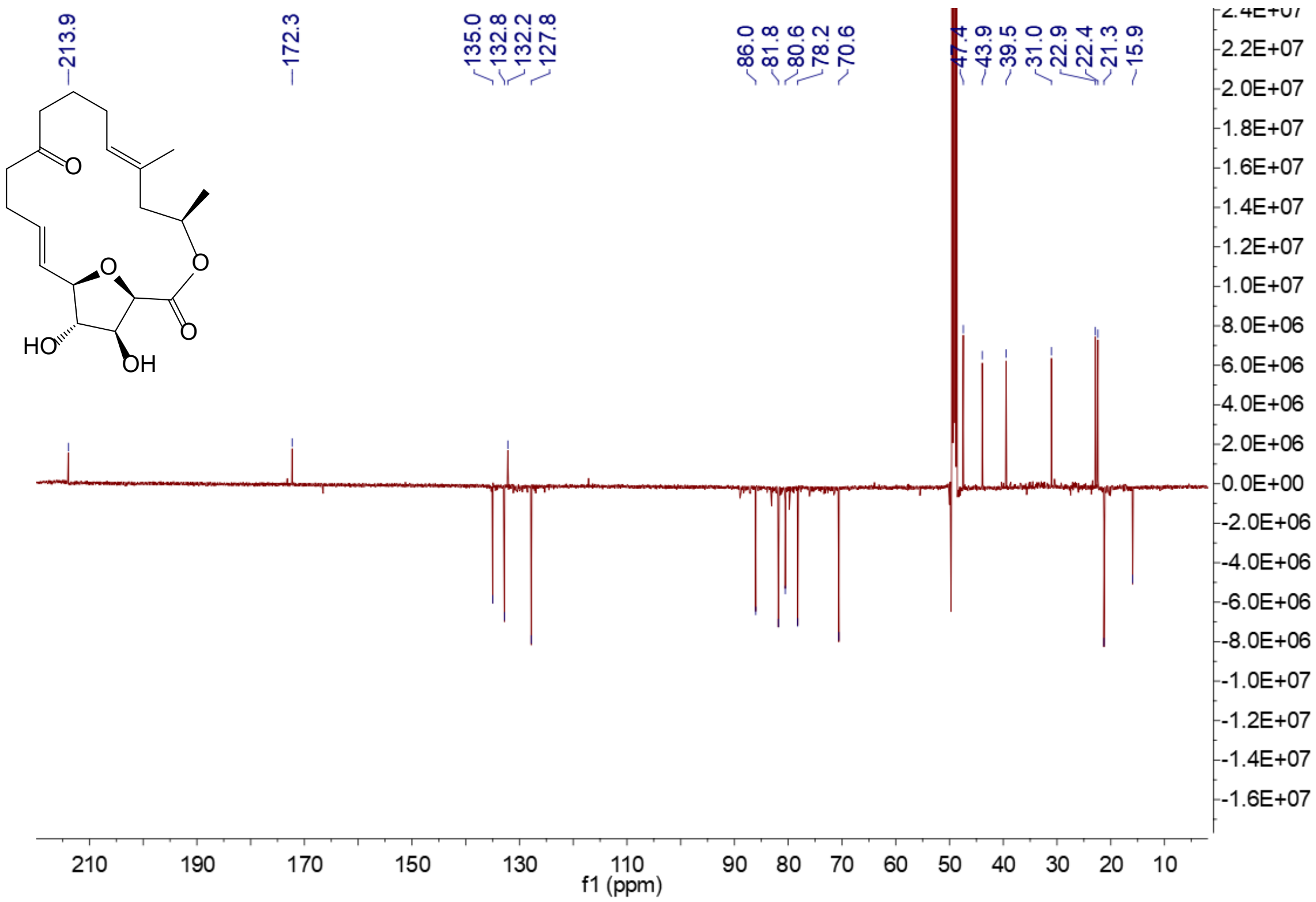

Supplementary Figure 51. ^{1}H-^{13}C HSQC NMR (600 MHz, methanol-d_4) spectrum of **6**.

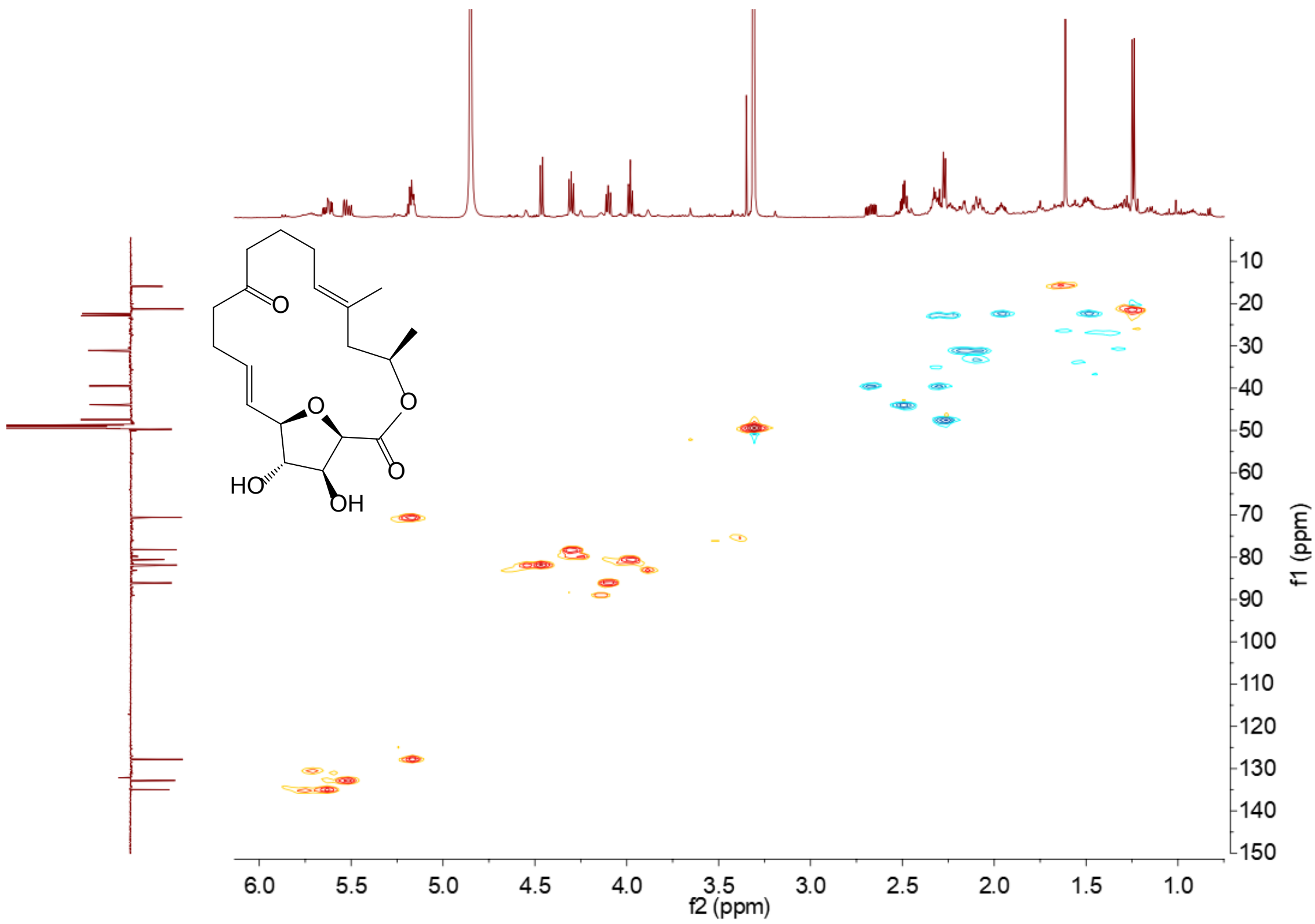

Supplementary Figure 52. ^{1}H-^{13}C HMBC NMR (600 MHz, methanol-d_4) spectrum of **6**.

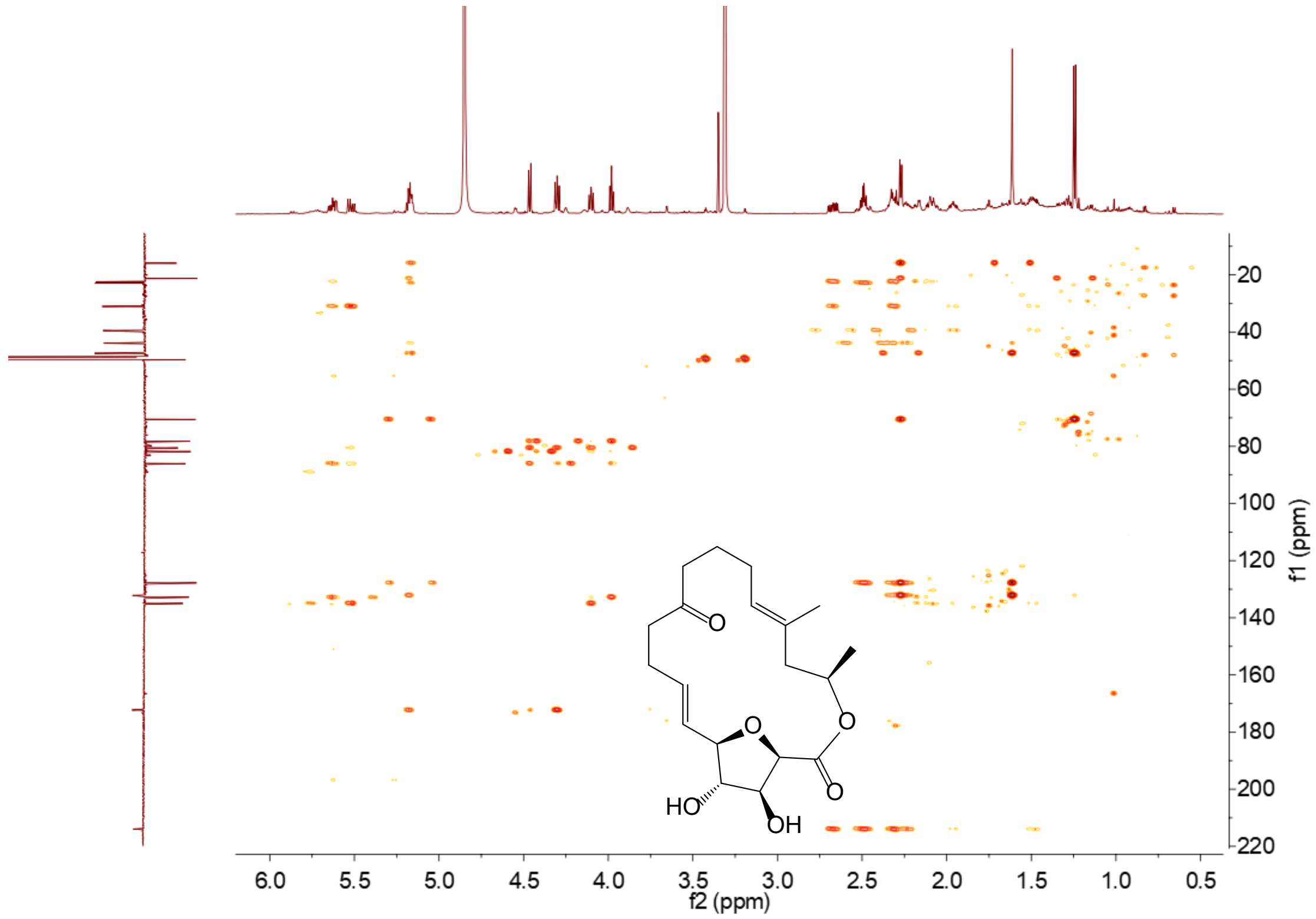

Supplementary Figure 53. ¹H-¹H COSY NMR (600 MHz, methanol-d_4) spectrum of **6**.

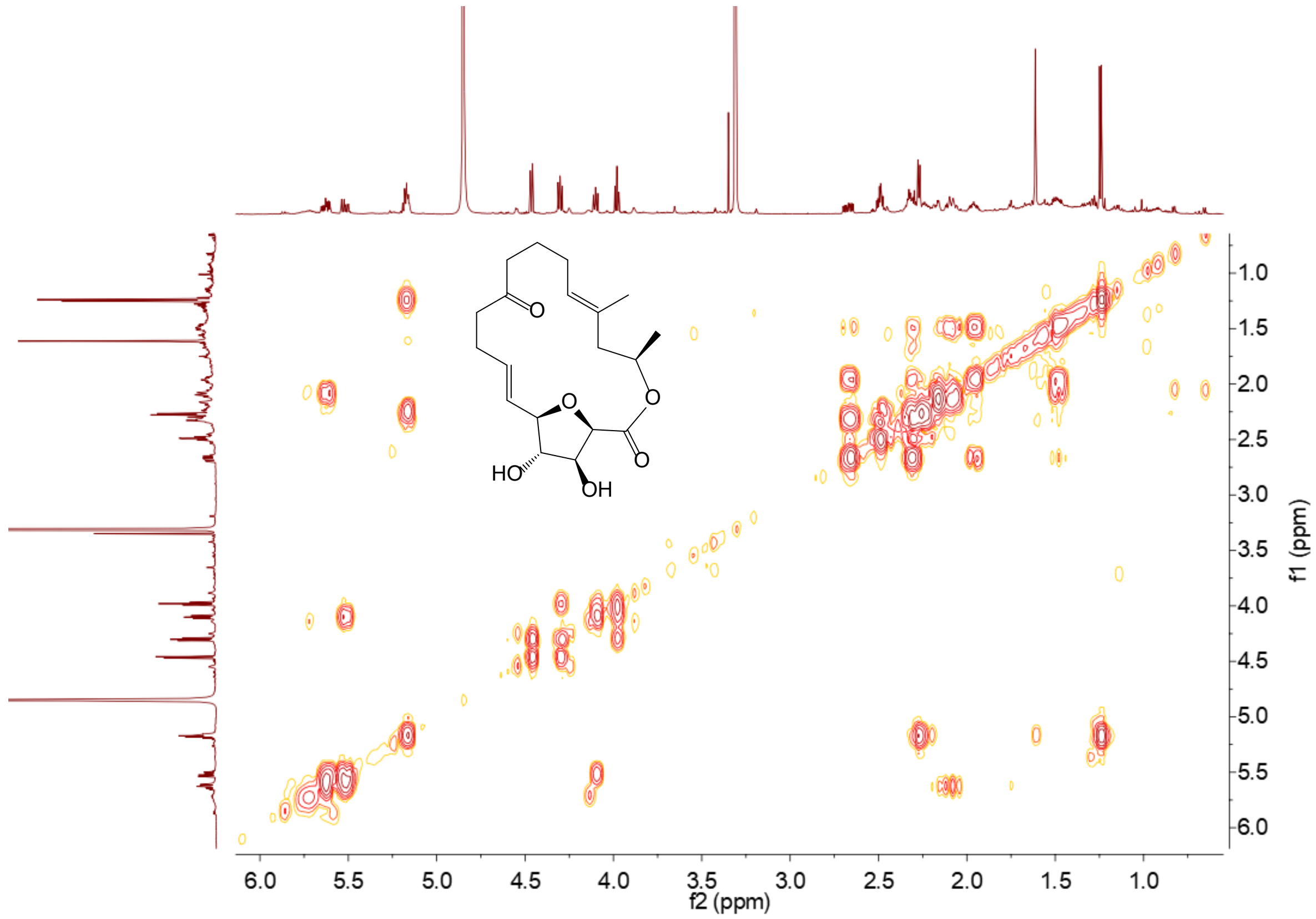

Supplementary Figure 54. ¹H-¹H NOESY NMR (600 MHz, methanol-d_4) spectrum of **6**.

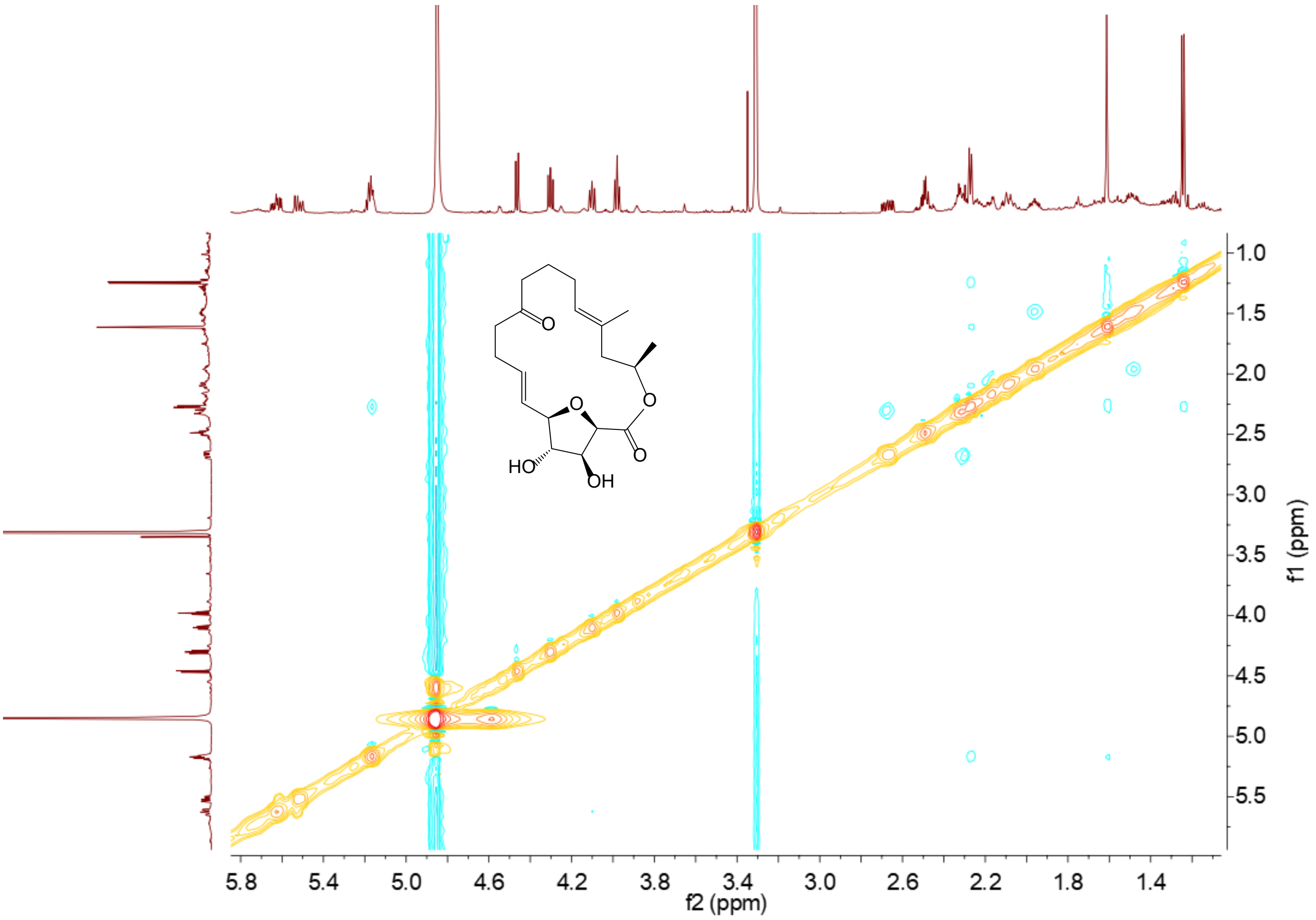

Supplementary Figure 55. HRESIMS spectrum of **6**.

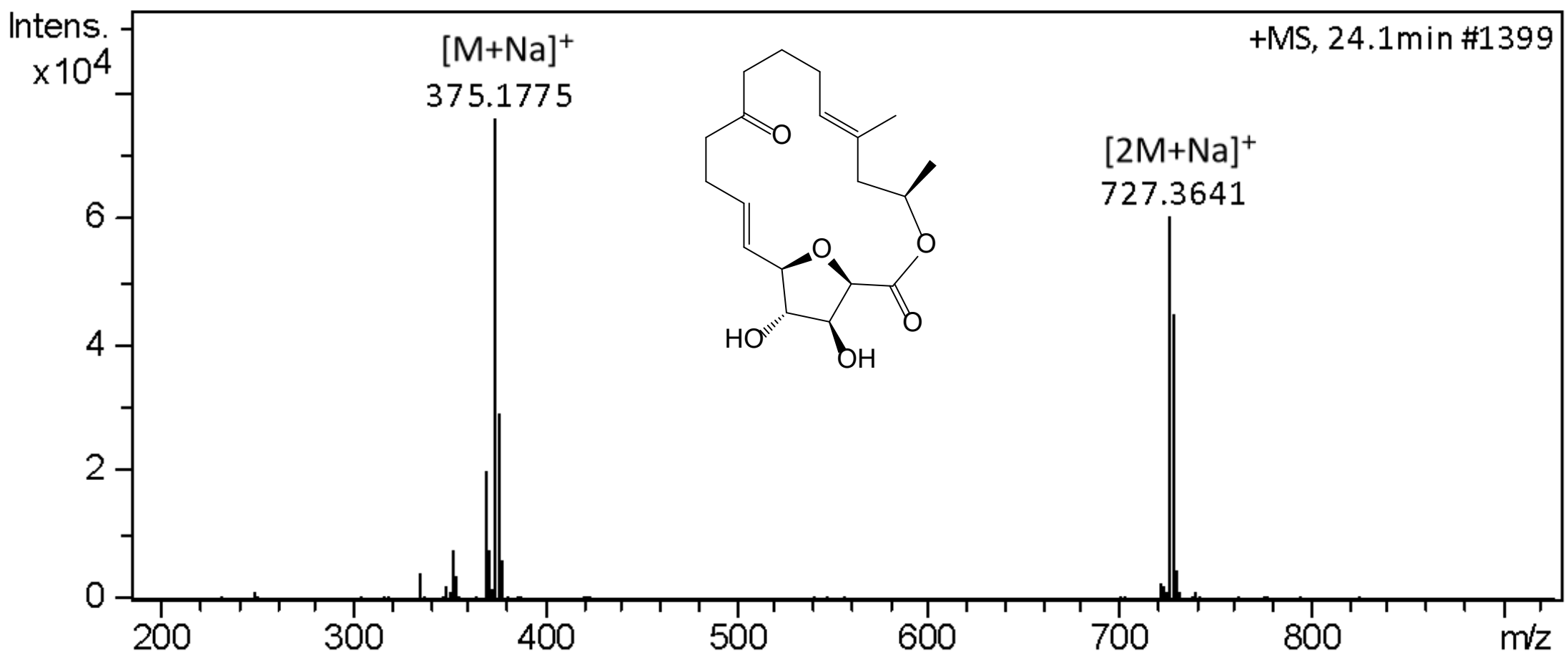

Supplementary Figure 56. ^{1}H NMR (600 MHz, methanol-d_4) of **7**.

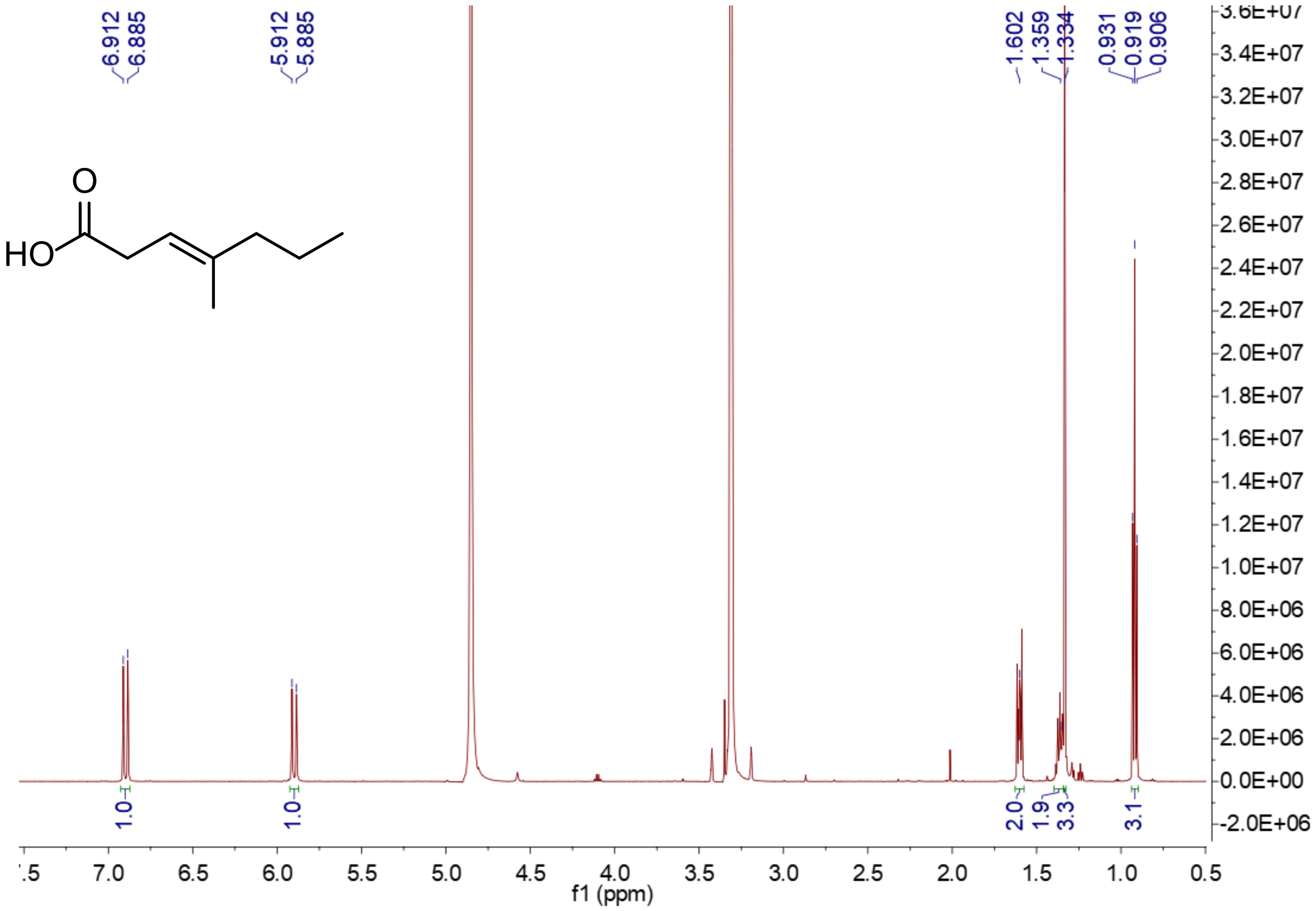

Supplementary Figure 57. ^{1}H NMR (600 MHz, methanol-d_4) of **8**.

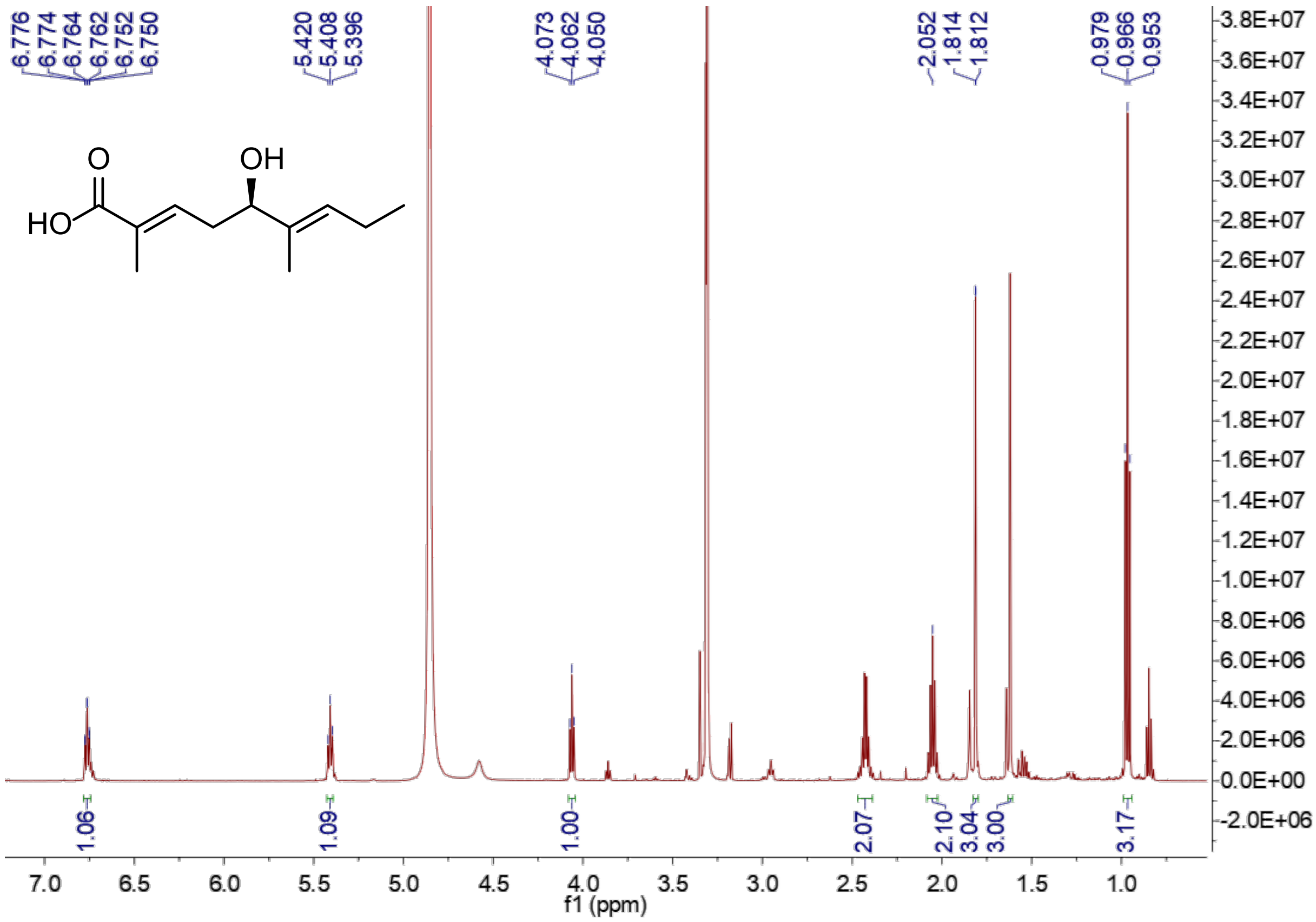

Supplementary Figure 58. ^{1}H-^{13}C HSQC (600 MHz, methanol-d_4) spectrum of **8**.

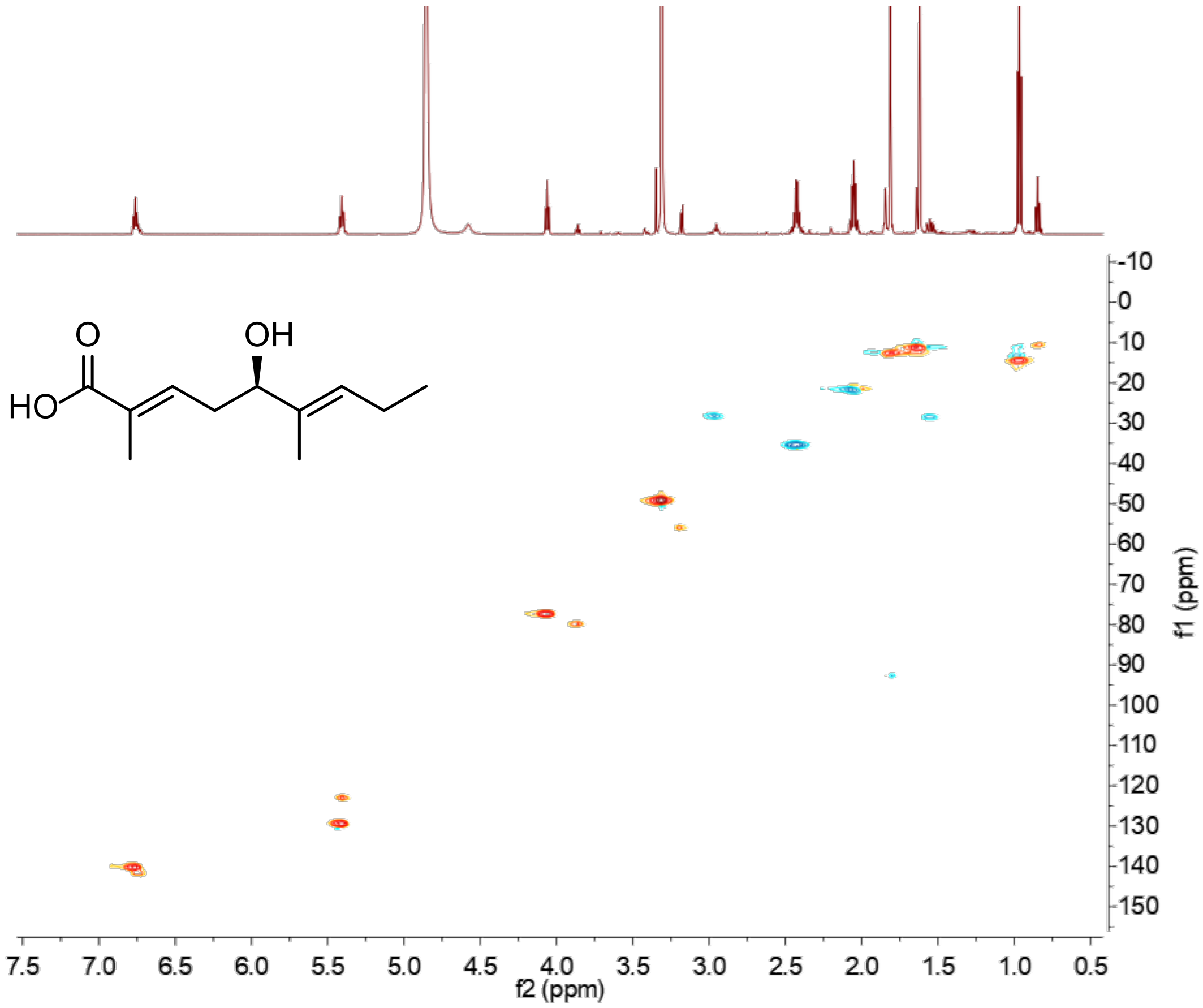

Supplementary Figure 59. ^{1}H-^{13}C HMBC NMR (600 MHz, methanol-d_4) spectrum of **8**.

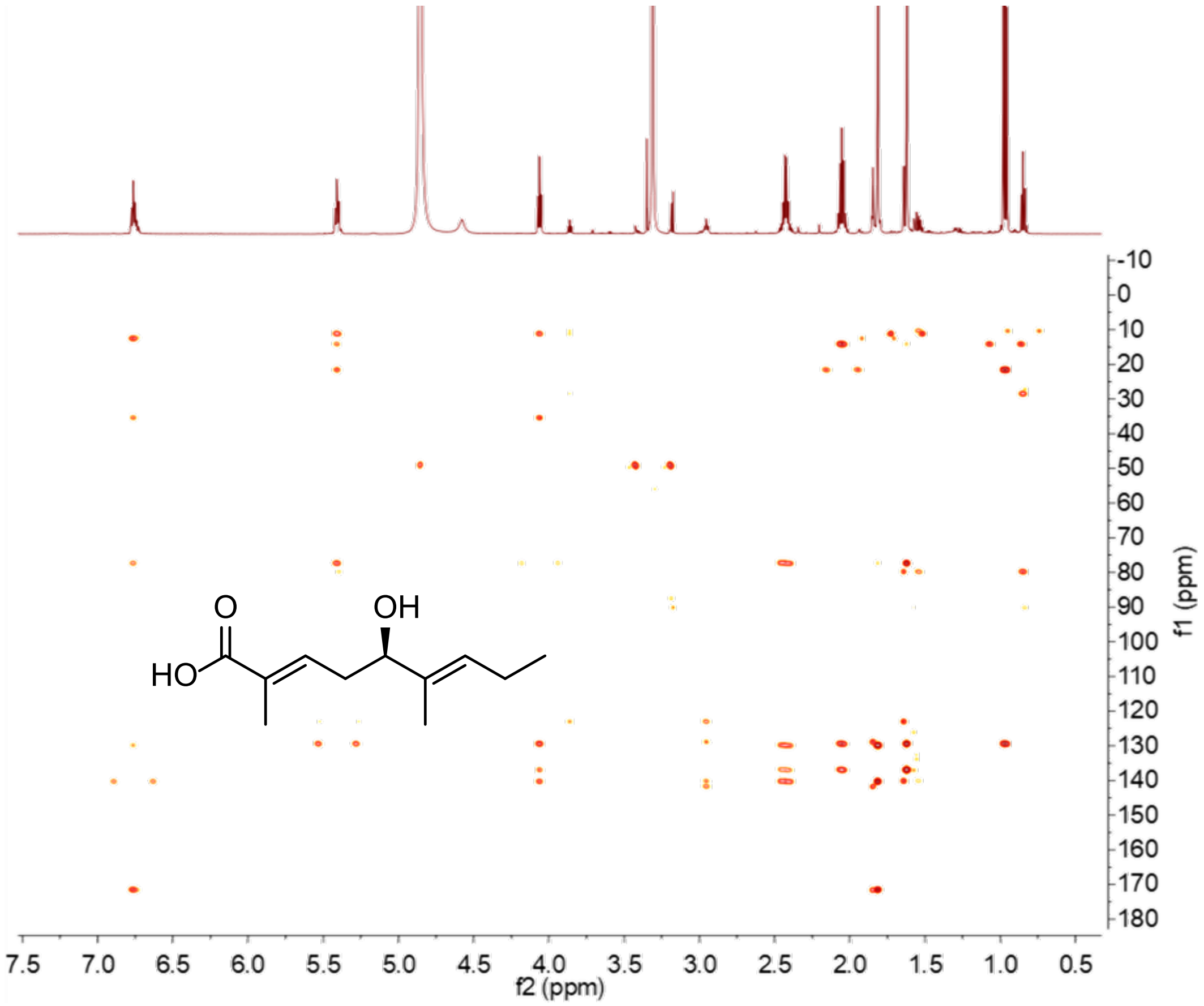

Supplementary Figure 60. ^{1}H-^{1}H COSY NMR (600 MHz, methanol-d_4) spectrum of **8**.

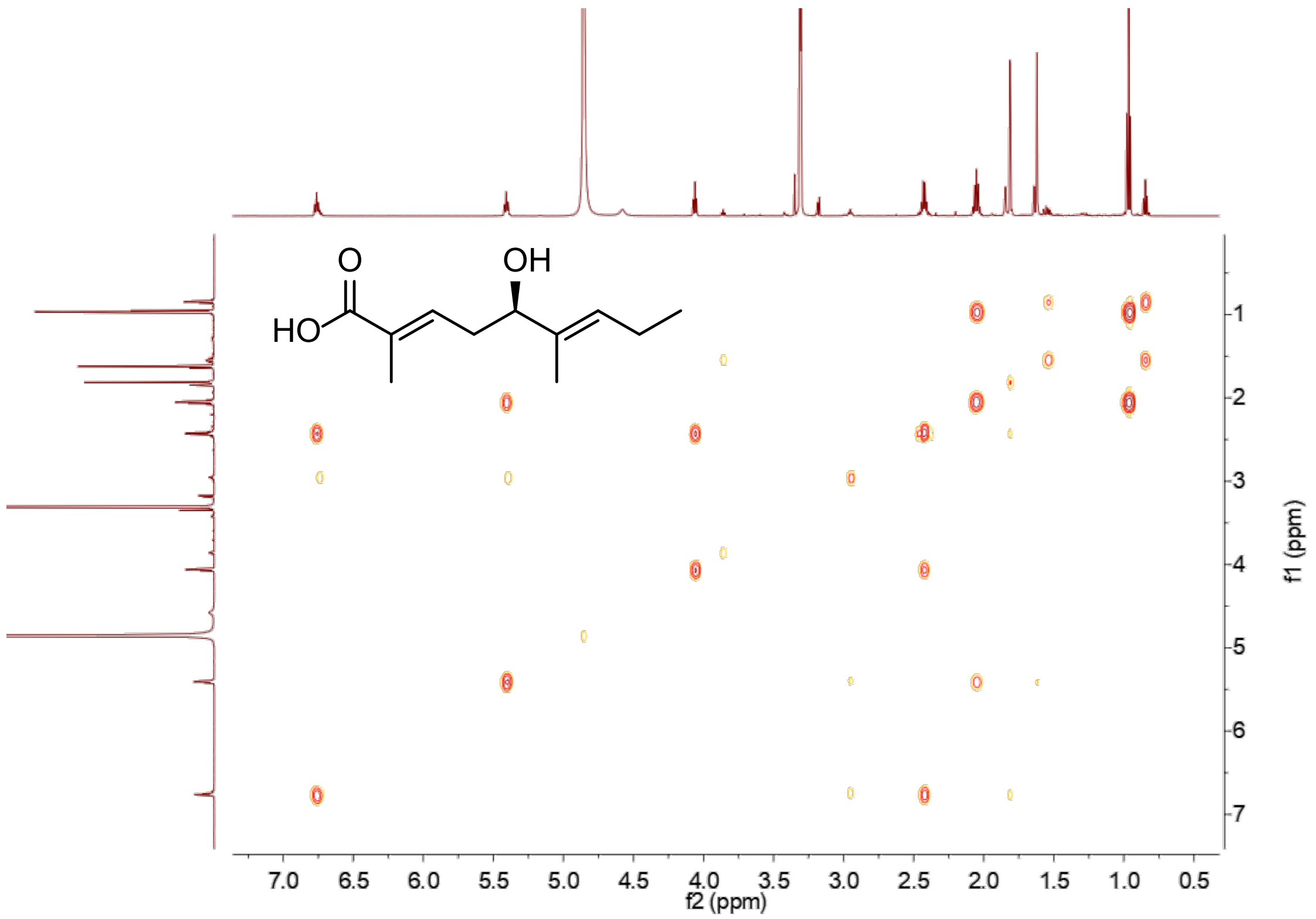

Supplementary Figure 61. HRESIMS spectrum of **8**.

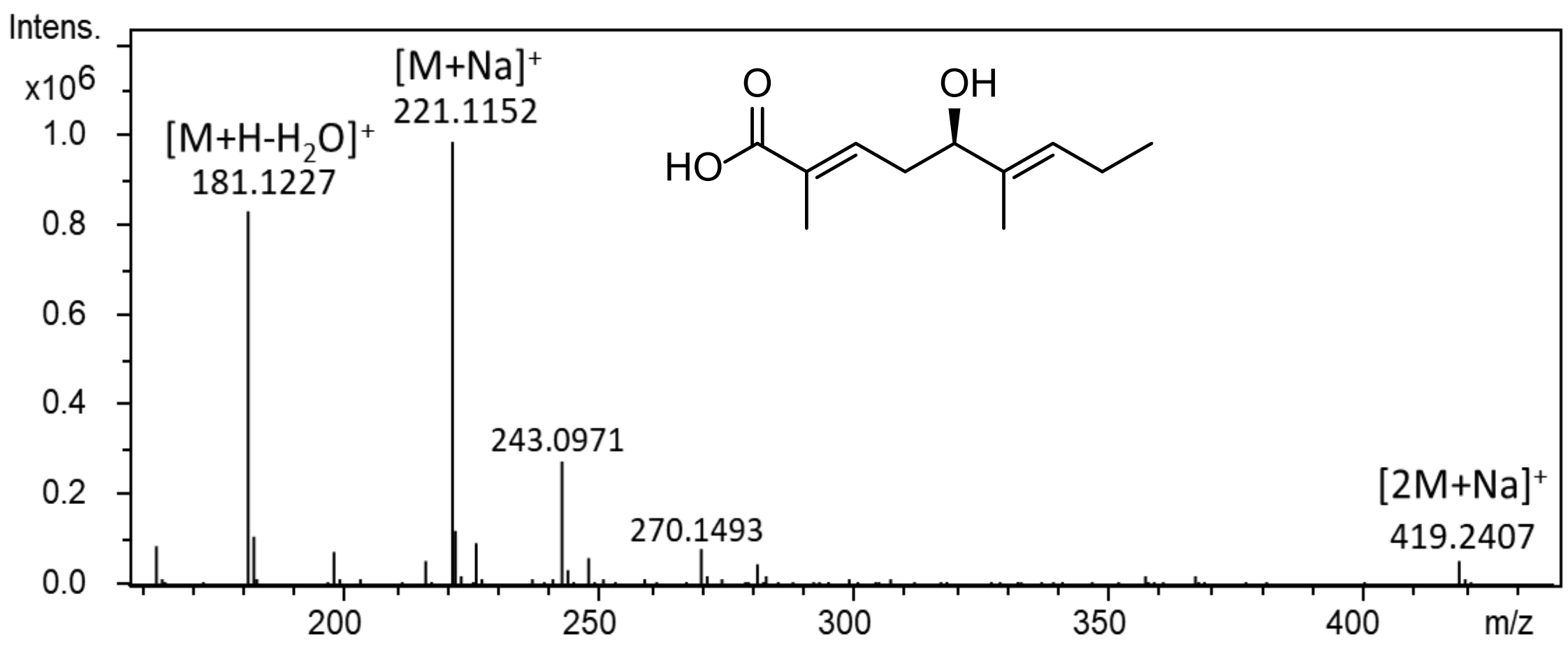

Supplementary Figure 62. ^{1}H NMR (600 MHz, methanol-d_4) of **9**.

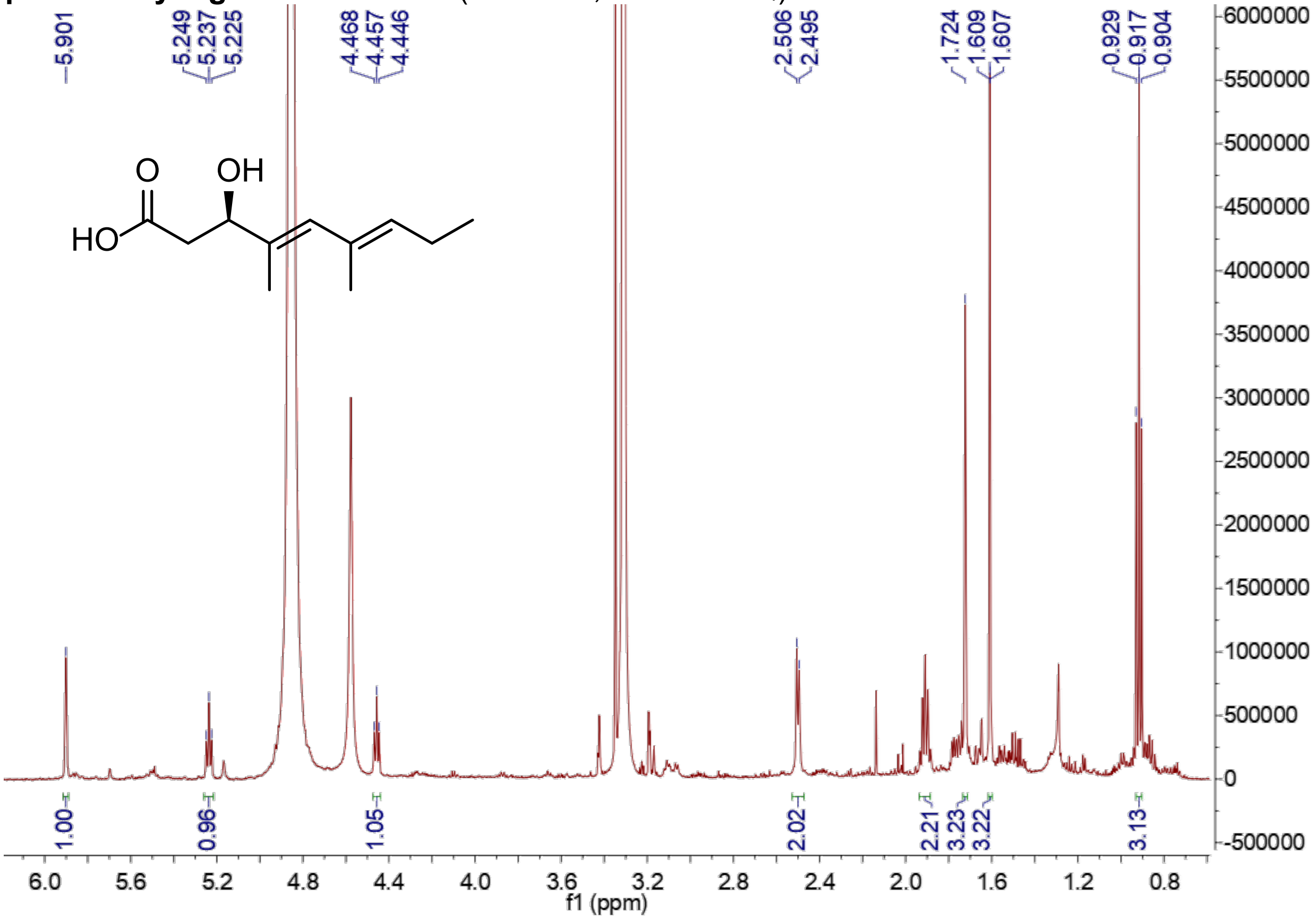

Supplementary Figure 63. ^{1}H-^{13}C HSQC NMR (600 MHz, methanol-d_4) spectrum of **9**.

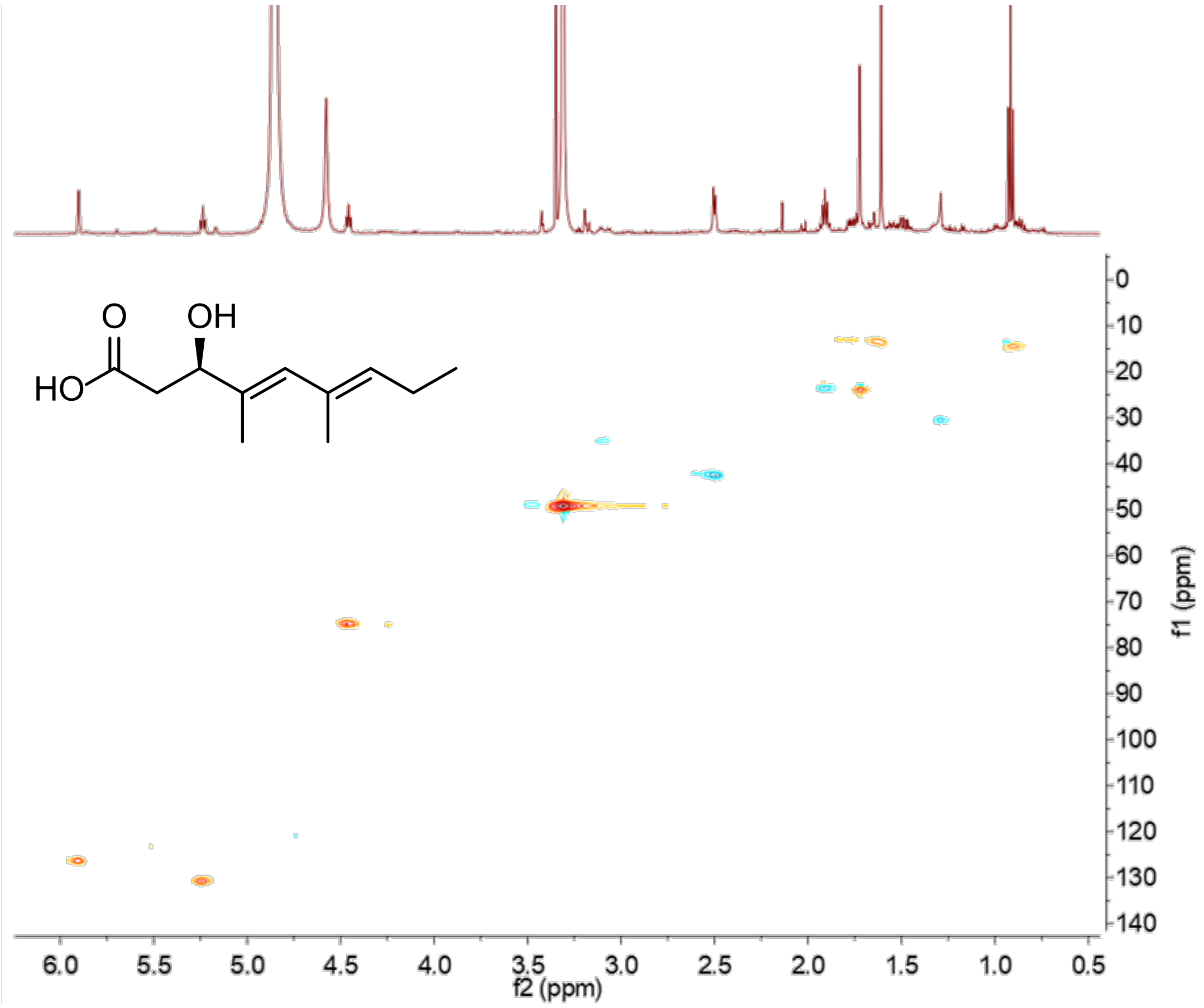

Supplementary Figure 64. ¹H-¹³C HMBC NMR (600 MHz, methanol-d_4) spectrum of **9**.

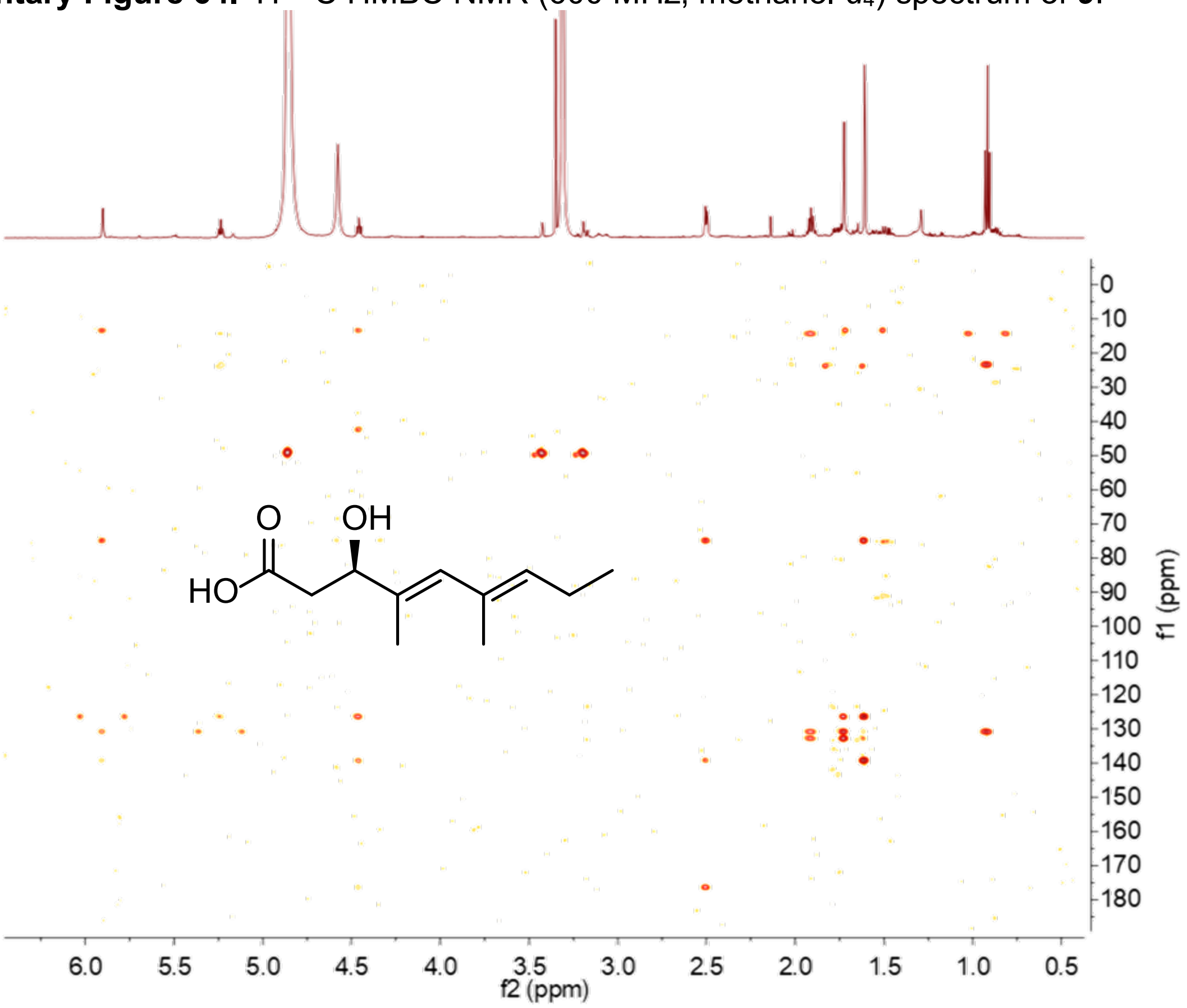

Supplementary Figure 65. ¹H-¹H COSY NMR (600 MHz, methanol-d_4) spectrum of **9**.

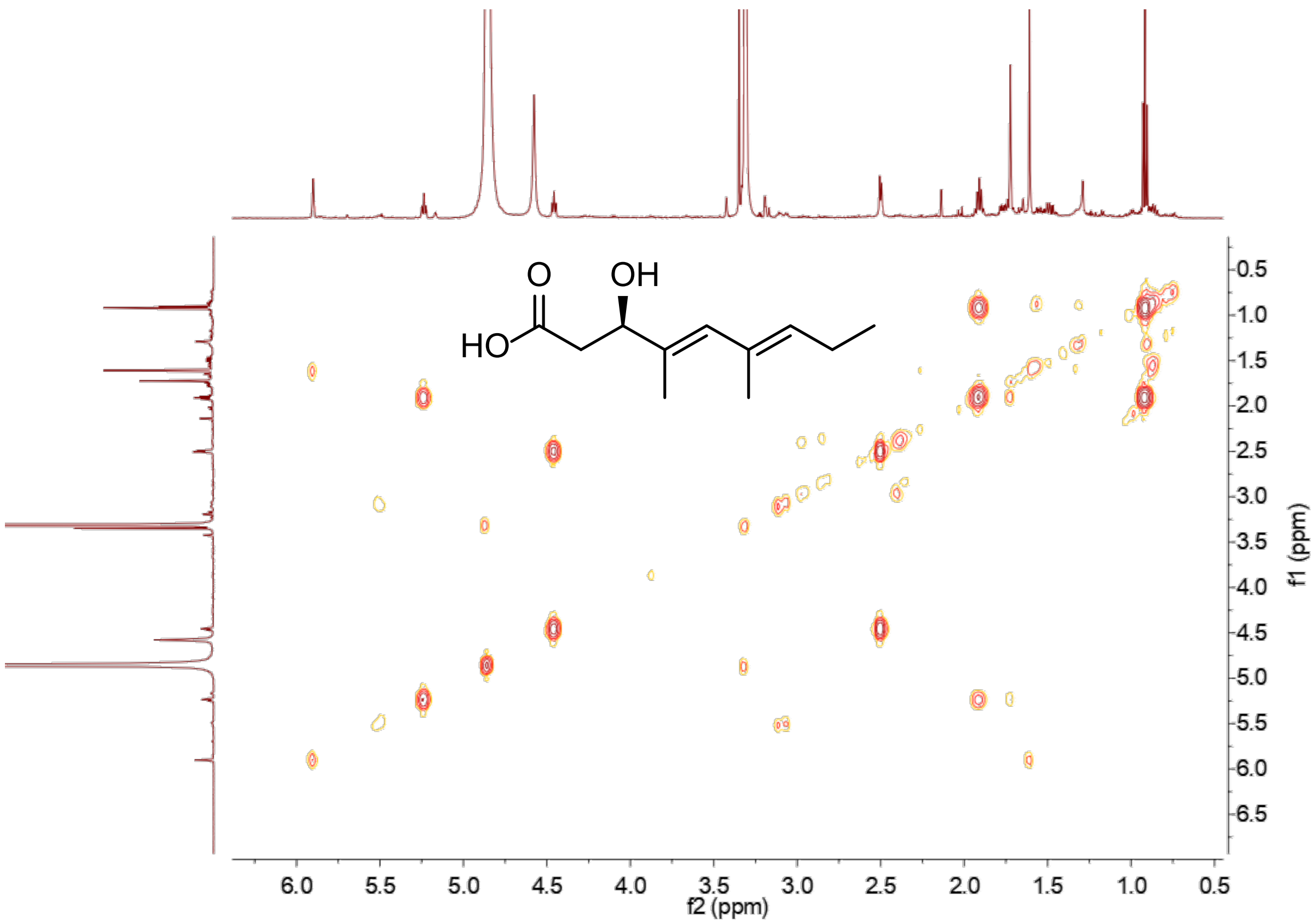

Supplementary Figure 66. HRESIMS spectrum of **9**.

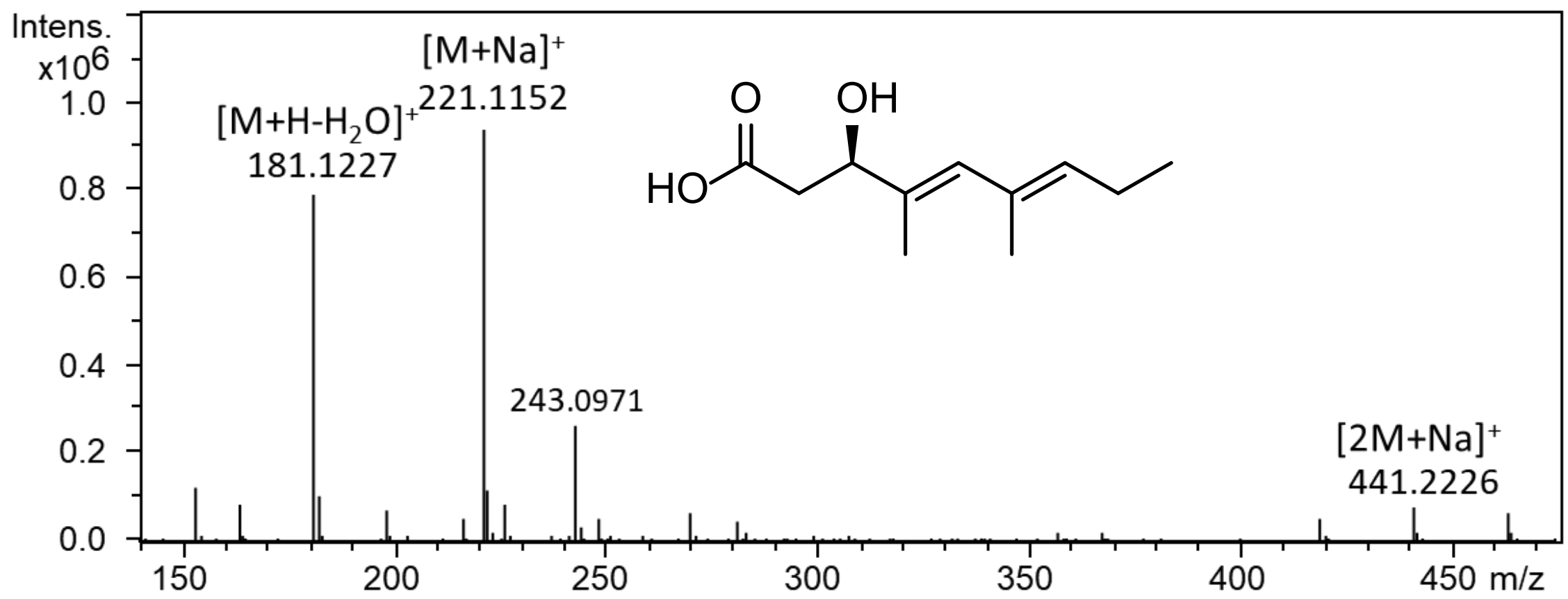

Supplementary Figure 67. ^{1}H NMR (600 MHz, DMSO-d_6) of **10**.

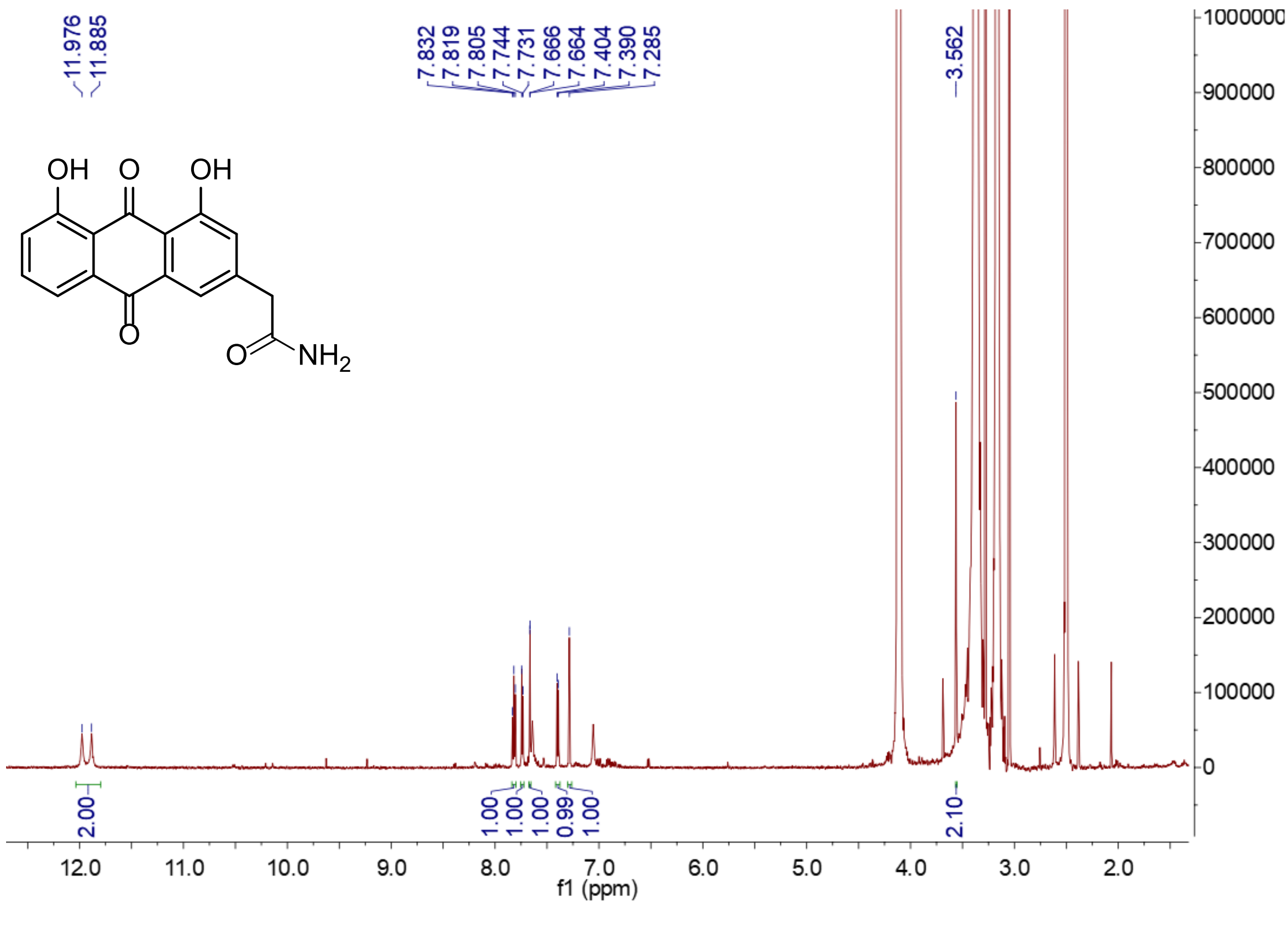

Supplementary Figure 68. DEPTQ NMR (150 MHz, DMSO-d_6) of **10**.

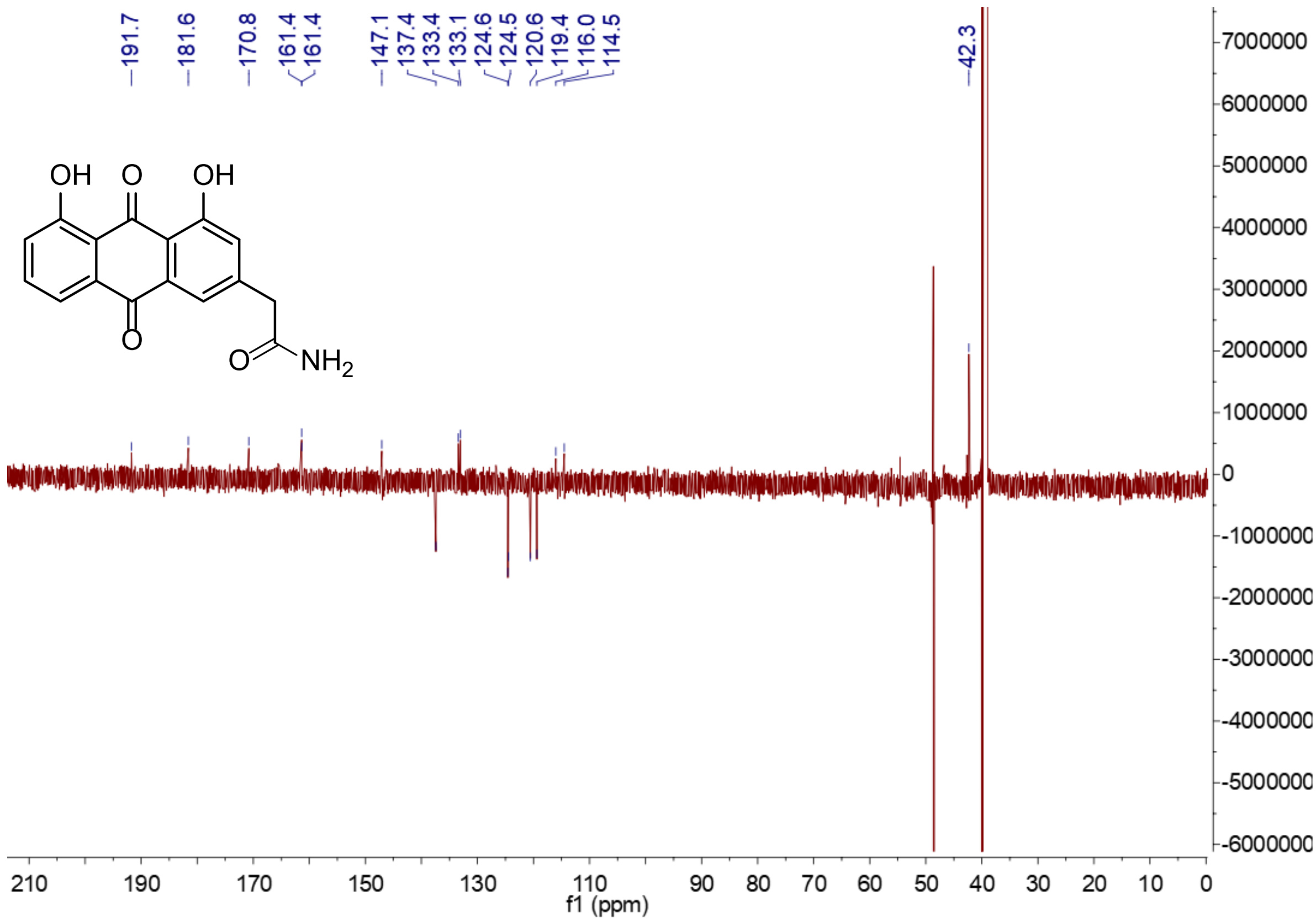

Supplementary Figure 69. ^{1}H-^{13}C HSQC (600 MHz, DMSO-d_6) spectrum of **10**.

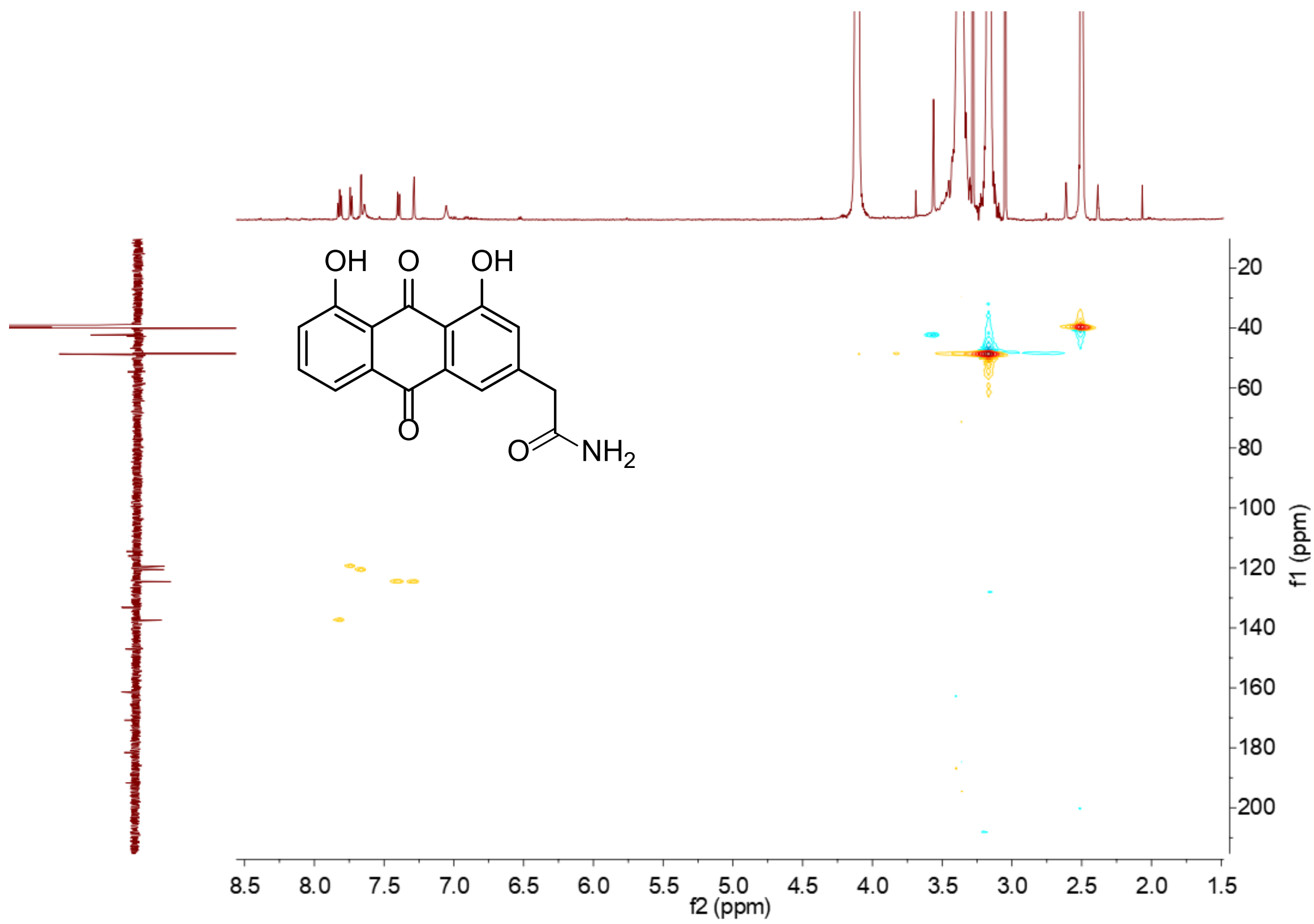

Supplementary Figure 70. ^1H-^{13}C HMBC NMR (600 MHz, DMSO-d_6) spectrum of **10**.

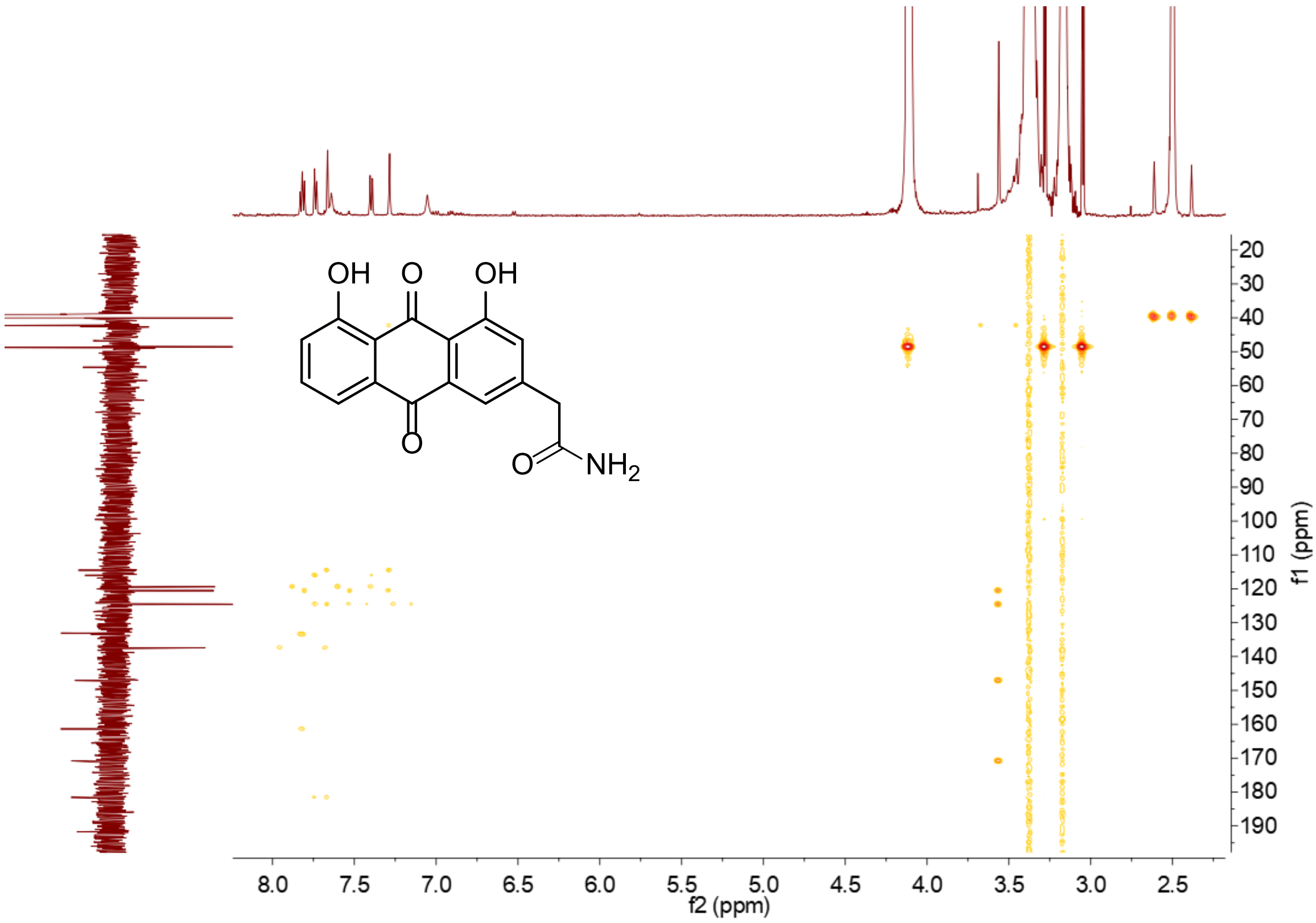

Supplementary Figure 71. ^1H-^1H COSY NMR (600 MHz, DMSO-d_6) spectrum of **10**.

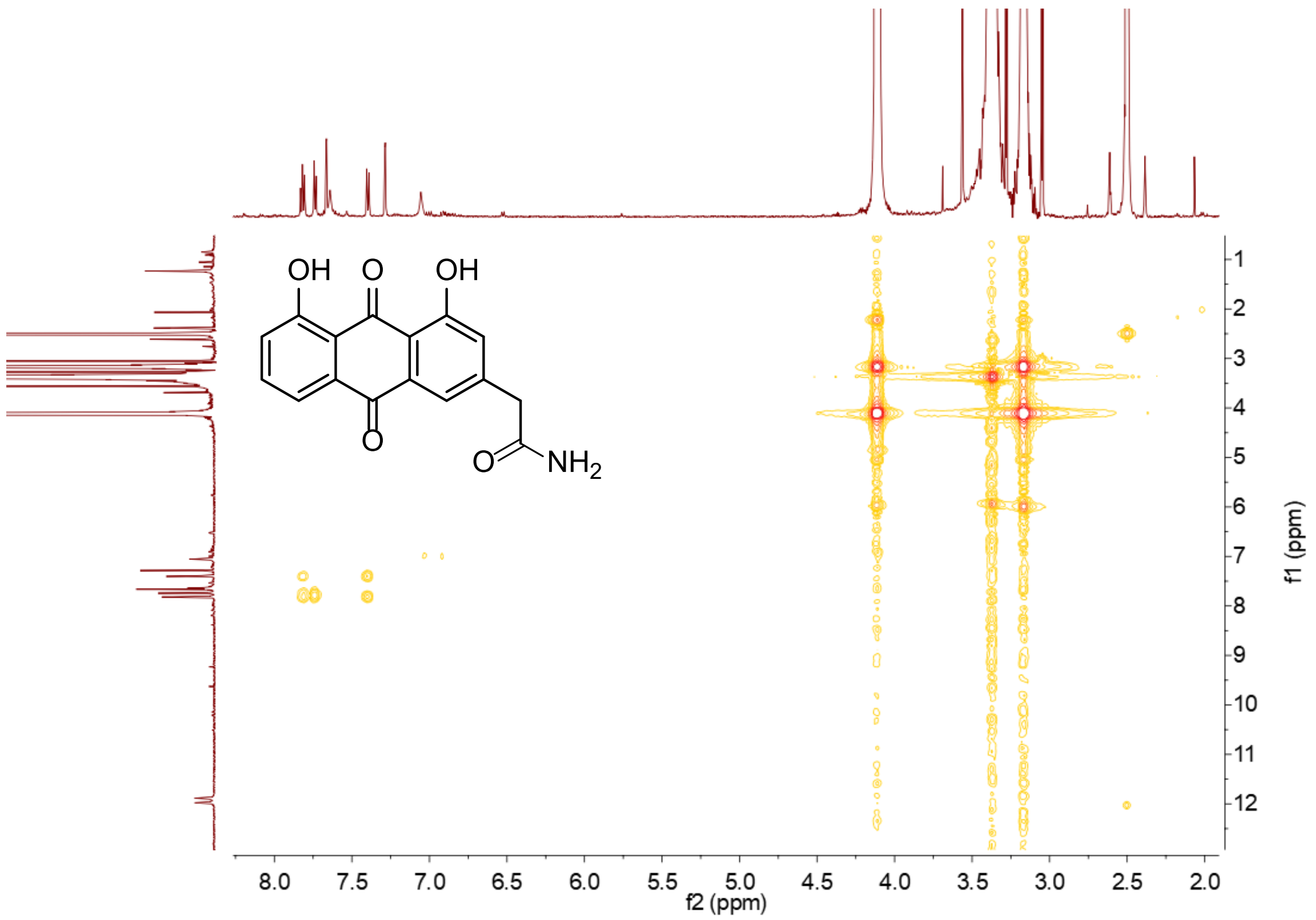

Supplementary Figure 72. HRESIMS spectrum of **10**.

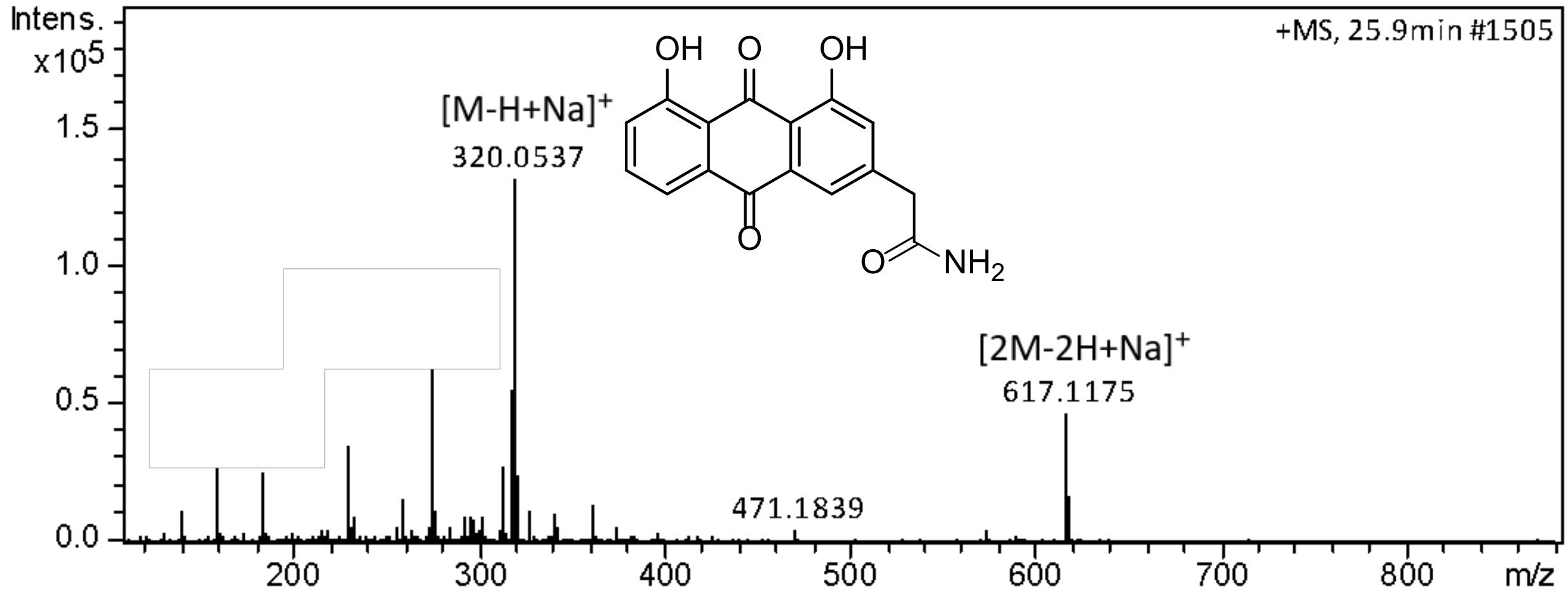

Supplementary References

1. Tzeng, L., *et al.* DNA Replication during Aggregation Phase Is Essential for *Myxococcus xanthus* Development. *J. Bacteriol.* **188**, 8, 2774-2779 (2006).
