# Supplementary material for: Development of efficient genetic toolkits and heterologous chassis for bioprospecting of myxobacteria
Source: Nat Commun. 2026 Jun 29;17:8074. doi: 10.1038/s41467-026-74908-x (PMC13454183; doi:10.1038/s41467-026-74908-x)
Supplement: Supplementary file 2 [file 41467_2026_74908_MOESM2_ESM.pdf]

<u>Description of Additional Supplementary Files</u>

Supplementary Data 1. Comprehensive annotations for 7,751 BGCs derived from 240 myxobacterial genomes. Note: “Product prediction”, BGC annotation predicted by BiG-SCAPE; “BGC class”, BGC type based on the product prediction; “GCF”, Gene Cluster Family number from the BiG-SCAPE clustering; “Ref_id”, reference BGC identity of MIBiG database classified by this GCF; “Type”, matching status of this BGC with the reference BGC identity.
