# Supplementary material for: Development of efficient genetic toolkits and heterologous chassis for bioprospecting of myxobacteria
Source: Nat Commun. 2026 Jun 29;17:8074. doi: 10.1038/s41467-026-74908-x (PMC13454183; doi:10.1038/s41467-026-74908-x)
Supplement: Supplementary file 3 [file 41467_2026_74908_MOESM4_ESM.pdf]

nature portfolio

Corresponding author(s): Changsheng Wu, Li Zhuo, Yue-Zhong Li

Last updated by author(s): Jun 12, 2026

Reporting Summary

Nature Portfolio wishes to improve the reproducibility of the work that we publish. This form provides structure for consistency and transparency in reporting. For further information on Nature Portfolio policies, see our Editorial Policies and the Editorial Policy Checklist.

Statistics

For all statistical analyses, confirm that the following items are present in the figure legend, table legend, main text, or Methods section.

n/a	Confirmed	
☐	☒	The exact sample size (*n*) for each experimental group/condition, given as a discrete number and unit of measurement
☐	☒	A statement on whether measurements were taken from distinct samples or whether the same sample was measured repeatedly
☐	☒	The statistical test(s) used AND whether they are one- or two-sided<br>*Only common tests should be described solely by name; describe more complex techniques in the Methods section.*
☒	☐	A description of all covariates tested
☒	☐	A description of any assumptions or corrections, such as tests of normality and adjustment for multiple comparisons
☐	☒	A full description of the statistical parameters including central tendency (e.g. means) or other basic estimates (e.g. regression coefficient) AND variation (e.g. standard deviation) or associated estimates of uncertainty (e.g. confidence intervals)
☐	☒	For null hypothesis testing, the test statistic (e.g. *F*, *t*, *r*) with confidence intervals, effect sizes, degrees of freedom and *P* value noted<br>*Give P values as exact values whenever suitable.*
☒	☐	For Bayesian analysis, information on the choice of priors and Markov chain Monte Carlo settings
☒	☐	For hierarchical and complex designs, identification of the appropriate level for tests and full reporting of outcomes
☒	☐	Estimates of effect sizes (e.g. Cohen's *d*, Pearson's *r*), indicating how they were calculated

Our web collection on statistics for biologists contains articles on many of the points above.

Software and code

Policy information about availability of computer code

Data collection	Bruker Compass DataAnalysis was used for chromatography and mass spectrometry. Bruker TopSpin was used for NMR data aquisition. SnapGene 4.1.9 was used for sequence data.
Data analysis	Raw genome sequencing data were retrieved from NCBI RefSeq database using the "datasets download" command-line tool. Biosynthetic gene clusters were annotated by antiSMASH 7.0, and classification by BiG-SCAPE 1.1.0 with the MIBiG repository 2.0 and PFAM database 32.0. The phylogenetic tree was constructed using the GTDB-Tk 1.6.0 and visualized via the "ggtree" R package. The NMR spectra were phased and baseline corrected by MestReNova (version 6.1.0). The MS data were analyzed by software DataAnalysis version 4.2. Statistical data were analyzed using GraphPad Prism 8.0.

For manuscripts utilizing custom algorithms or software that are central to the research but not yet described in published literature, software must be made available to editors and reviewers. We strongly encourage code deposition in a community repository (e.g. GitHub). See the Nature Portfolio guidelines for submitting code & software for further information.

Data

Policy information about availability of data

All manuscripts must include a data availability statement. This statement should provide the following information, where applicable:

- Accession codes, unique identifiers, or web links for publicly available datasets
- A description of any restrictions on data availability
- For clinical datasets or third party data, please ensure that the statement adheres to our policy

The genome data used in this work are downloaded from NCBI RefSeq database [https://www.ncbi.nlm.nih.gov/refseq/], with details given in Supplementary Data 1. The processed spectroscopy date of the new compounds and Sanger sequencing data of all genome-edited strains in this study have been deposited in the figshare repository [doi: https//doi.org/10.6084/m9.figshare.31264693]. All other experimental data, including annotated DNA sequences and numerical raw data, are compiled in the Source Data zip file provided with this article.

Research involving human participants, their data, or biological material

Policy information about studies with human participants or human data. See also policy information about sex, gender (identity/presentation), and sexual orientation and race, ethnicity and racism.

Reporting on sex and gender	This research did not include human participants.
Reporting on race, ethnicity, or other socially relevant groupings	This research did not include human participants.
Population characteristics	This research did not include human participants.
Recruitment	This research did not include human participants.
Ethics oversight	This research did not include human participants.

Note that full information on the approval of the study protocol must also be provided in the manuscript.

Field-specific reporting

Please select the one below that is the best fit for your research. If you are not sure, read the appropriate sections before making your selection.

☒ Life sciences ☐ Behavioural & social sciences ☐ Ecological, evolutionary & environmental sciences

For a reference copy of the document with all sections, see nature.com/documents/nr-reporting-summary-flat.pdf

Life sciences study design

All studies must disclose on these points even when the disclosure is negative.

Sample size	For experiments involving quantifications of results, three biological replicates were used to test the variablility between samples. The sample size was determined following standard design in the field.
Data exclusions	No data were excluded from analyses.
Replication	All replicates from independent experiments were consistent with the results presented in the manuscript. Means and individual value distribution demonstrating reproducibility of our experiment were shown in the figures.
Randomization	The experiments were not randomized.All samples were processed in parallel under identical conditions,and no additional covariates were present that would require randomization for this study design.
Blinding	The investigators were not blinded to allocation during experiments and outcome assessment.

Reporting for specific materials, systems and methods

We require information from authors about some types of materials, experimental systems and methods used in many studies. Here, indicate whether each material, system or method listed is relevant to your study. If you are not sure if a list item applies to your research, read the appropriate section before selecting a response.

Materials & experimental systems

n/a	Involved in the study
☒	☐ Antibodies
☒	☐ Eukaryotic cell lines
☒	☐ Palaeontology and archaeology
☒	☐ Animals and other organisms
☒	☐ Clinical data
☒	☐ Dual use research of concern
☒	☐ Plants

Methods

n/a	Involved in the study
☒	☐ ChIP-seq
☒	☐ Flow cytometry
☒	☐ MRI-based neuroimaging

Plants

Seed stocks	No plants were used in this research.
Novel plant genotypes	No plants were used in this research.
Authentication	No plants were used in this reseach.
