# Supplementary material for: Development of efficient genetic toolkits and heterologous chassis for bioprospecting of myxobacteria
Source: Nat Commun. 2026 Jun 29;17:8074. doi: 10.1038/s41467-026-74908-x (PMC13454183; doi:10.1038/s41467-026-74908-x)
Supplement: Supplementary file 4 [file 41467_2026_74908_MOESM5_ESM.pdf]

nature portfolio

Peer Review File

Development of efficient genetic toolkits and heterologous chassis for bioprospecting of myxobacteria

Corresponding Author: Professor Changsheng Wu

This file contains all reviewer reports in order by version, followed by all author rebuttals in order by version.

Version 0:

Reviewer comments:

Reviewer #1

(Remarks to the Author)
This manuscript reports the development of a genetic toolkit in Myxococcus xanthus, to unlock the large potential of myxobacterial secondary metabolites. Myxobacteria are prolific producers of bioactive natural products with complex structures and potent biological activities. However, the discovery and production of new compounds are hindered by difficulties in culturing and genetically manipulating non-model myxobacteria. This work builds on previous genetic tool development (e.g. HR, galK counterselection, φC31-mediated site-specific integration, cre-loxP, pMiniHimar mariner-transposon) by this group, employing a RecET recombineering system identified from other myxobacteria (MxRedET), and the TnpB system (specifically ISDra2) to enable markerless deletions up to 200 kb, duplex editing, and SNP generation. They use these new and existing tools to engineer a chassis strain of Myxococcus xanthus DK1622, MxPKS (derived from a previously engineered Hu04 strain) to evaluate heterologous expression of BGCs resulting in discovery of novel polyketides. While there are some promising novel findings here, it is often hard to determine the impact due to missing data, teasing out relevant new versus previous genetic tools for the ultimate effort of designing the chassis to evaluate known BGCs for function and elucidate novel BGCs or lack of clear rationale for experimental design. To this end, the manuscript reads almost like 2 separate papers—a comprehensive search of BGCs across myxobacteria and tool development—the tool development beyond the recombineering machinery and the TnpB editing not directly relevant to the construction of MxPKD (e.g. the Cre-loxP work) should be greatly reduced in scope or removed. In addition, there are several instances of awkward phrasing and grammatical errors throughout the text, and the manuscript would benefit from careful editorial revision to improve clarity and readability. There are also many opportunities to edit the text to better describe and confer rationale, experimental design, and interpretation of results to a more general audience. Therefore, I would only support publication after a major revision.

Some specific points that need to be addressed are here:
Red/ET should have the "/" as they are distinct recombineering systems (Lambda Red, RecET) if using that terminology; Red/ET is not generally common in the broader field, its usually just referred to as recombineering. Also ensure that Redα is also referred as "exo" and Redβ as "beta" as these are the more commonly used gene names in the broader field. Also, for consistency, ensure that "RedET" consistently refers to the same system; currently, it alternates between the E. coli and myxobacterial systems which makes the context confusing.

When stating "In addition, Red/ET recombineering excels over RecA-dependent HR by requiring only short homology arms (40–100 bp), simply crafted through PCR" it should be stated that this is known for E. coli, as longer arms are often still needed in other bacteria

The citation #26 is not correct for this statement: "For instance, its combination with Cre/loxP facilitates complex, markerless manipulations"

The statement "Its pairing with CRISPR/Cas9-mediated double-strand breaks dramatically enhances the efficiency of targeted gene replacement." Is missing seminal references for E. coli (e.g. https://doi-org.ornl.idm.oclc.org/10.1038/srep15096, https://doi.org/10.1021/acssynbio.5b00187)

The authors should include description of the broader classification of the TnpB as well as introduction of the system they chose to deploy (ISDra2) and why instead of CRISPR-Cas9 in the introduction and not just the discussion as rationale for

the study. In the Introduction, the justification for adopting TnpB instead of the well-established CRISPR/Cas platforms is not so clear. The authors mention its small size and specificity but could clarify why these features are particularly advantageous in M. xanthus.

In the introduction, the authors imply that morphological improvements of DK1622 (such as dispersed growth and single-colony formation) and the removal of specific BGCs inherently translate to better heterologous expression outcomes. This assumption lacks further explanation. The authors should provide a logical explanation connecting these phenotypic traits to enhanced biosynthetic performance.

“It was advisable to exclude these two families as priority for future genome mining endeavors” sounds like a subjective statement. Can the authors provide clarification?

The authors state “The resulting constructs were separately integrated at the Mx8 site of the M. xanthus DK1622 genome.” but it appears from the text and Figure S3 that there is only 1 construct? It is also missing in the text how and where this construct is integrated. It appears from Figure S3 its using the φC31-mediated site-specific integration referenced later based on the attP-attB sites shown? This all needs to be described here.

It is unclear why a medium-strength constitutive promoter was selected. Given that recombineering genes are often toxic when constitutively expressed, many bacterial systems instead employ transient or inducible expression strategies. The authors should therefore assess and report any effects on cell fitness and potential changes in mutation frequency to provide a more comprehensive evaluation.

Figure 2 includes multiple statistical significance annotations (e.g., P values and asterisks), but the caption does not specify which statistical tests were used to generate these values. The authors should clearly state the statistical test applied. This clarification should also be extended to all other figures where statistical comparisons are presented.

Figure 2a, “yqaJ” is the label for what I assume is the recE homolog, the authors should explain this in the text or change to recE. The caption refers to “RecE,” whereas the figure itself labels it as “yqaJ.” Please standardize the nomenclature throughout for clarity. Additionally, it is unclear what the white arrows in panel (a) represent are these hypothetical or uncharacterized neighboring genes?
The authors state that they use “flanking homology arms as short as 60 bp” but do not specify what the length of the HA sequence is for Figure 2a

Figure 2b, the red bar should be labeled fraction edited, and the y-axes needs to be labeled accordingly

Figures 2 c-f, the authors state they are showing recombination efficiency but are only showing CFU/ml, which does not necessarily equate with editing and must be verified by PCR with a statistically relevant number of colonies. More discussion is needed before you discuss the experimental parameters for better understanding of what was gleaned from these data as well. For example, why is there a substantial drop off after 200 bp, but a jump at 100 bp (c)? Why a substantial jump at 10 h from 8 h (d)? 1000ng DNA to 1200 ng DNA (e)? Drop in efficiency for 60-100 kb deletion but back up from 160-180, and then the huge drop at 200 (f)? None of these make sense empirically without seeing the editing efficiencies on the graphs, and all the data could be strengthened by comparing with native recombination machinery as a control

Figure 2g-h, this is really just a 2-step tandem transformation method, not technically duplexed. Also in (h) it would be useful to see CFU and targeting other sites with different types of mutations as ~27% may not be either a “standard” percent depending on mutation and loci and may thus not be sufficient to use this method for all combinations to get double transformants, saving no time over just doing subsequent transformations. It is also perplexing again at the jump from 2-4 h when that wasn’t seen for the single mutants in (c)

For the marker recycle using Cre-loxP, the recombineering machinery isn’t necessary for this, this really isn’t novel at all and couldn’t be used for any edits where the cre-loxP scar would impede, e.g. base editing, need to better justify novelty versus native system) a comparison could help)

Figure S5 and S6, the design of the experiment should be more clearly stated to understand the results in the text. This isn’t at all in the figure and isn’t helpful in knowing what the expected size of the WT versus integrated construct would be

Figure 3a isn’t necessarily correct in the lower half of the figure and description in the text, the edit introduced by the repair template is removing the target sequence for the TnpB/reRNA complex to bind and cut, protecting the edited sequence from cutting if repaired

For Figure S7, it is stated that the reRNA is also on the construct, is it a non-targeting sequence as it is not causing cell growth defects? This needs to be better explained

For Figure 3b, where is the reRNA targeting? Important to include

For Figure 3c, the authors need to more clearly explain the mutation you are introducing and that this is WITHOUT selection, understanding may be improved by a schematic

Figure 3d, the authors need to show editing efficiency alongside CFUs and this is not indicative of no effect of HA length

(and their conclusion that the DSB is driving repair is not consistent with results from 1e that aren't noticeably statistically different), the authors need to more clearly address experimentally

Figure 3e needs statistical significance applied and editing % included, this does not clearly show that DNA ratio is a significant driver as stated in the text

Figure 4a could be much clearer by delineating the construct and deletion sizes more clearly across the diagram

Figure 4b-e, the authors now only show the editing efficiency, what was the CFU/ml? These numbers should really consistently be shown across all figures, as well as more tick marks should be added on x-axis to more clearly see representative data, and there should be data on colonies screened included in each legend as well as the actual target region as this can make a difference as to where it is located on the chromosome (e.g. https://doi.org/10.1021/acssynbio.5b00187)

Figure S8, you have roman numeral numbers delineating steps in the legend but don't show them in the figure, should be included

Figure 4d, what were the sizes and locations of the 2 targeted sites? The authors discuss an attempt at expressing 2 ISDra2 nucleases (why do you need 2? Do you mean the reRNAs for targeting? Why not include both reRNAs on the same ISDRa2 vector?) from a single plasmid and introduction of 2 HR templates. What were the sites, and which one was preferentially edited? If this is included, there should minimally be data included in supplemental.

For Figure 4e, there should be a more thorough discussion on the tested classes of SNE mutations as well as chromosomal location attempted

The authors say 2 weeks to mutant in abstract, but 3 weeks in the text, should resolve

The authors need to clarify whether the Hu04 strain that the MxPKS was derived from was also the strain that the genetic tools were evaluated in, and the authors should denote in table 1 what mutations were in that starting strain versus the new mutations introduced. If KO7 was the strain you started with to knock out other competing BGCs, where KO5-6 made before or after? Need to clarify
The authors claim that the HPLC profile of KO12 was "cleaner" than WT, but this should minimally be shown in supplemental if it is included (I see MxPKS in Figure S17, but it is not called out in the manuscript text)

If φC31-mediated site-specific integration and the pMiniHimar mariner-transposon containing plasmid were utilized for integration of BGC components in the MxPKS chassis, why was cre-loxP such a focus in the tools aspect? Clarification of exactly why and how these specific strategies were employed in MxPKS BGC expressing strains as well as whether they were sequenced, etc. is needed. Also, there is very little discussion of the promoter replacements (strength?) in the text, which is also needed to clarify the experimental design.

Figure 5a could be more clearly labeled with PKS types and linked to 5b and c, maybe color coding or some other clear delineation, to ensure broader reader understanding. The color coding and abbreviations (KS, AT, KR, etc.) are used without a legend in the figure, this should be explicitly defined, even if standard. It would also help to explain the meaning of the white and gray arrows—presumably non-PKS genes or hypothetical ORFs—but this is not specified in the caption.

Figure 5c, While the caption states which are newly identified compounds (4–9 and 12–15), but it would be clearer if these were visually differentiated (e.g., by shading or an asterisk).

Missing callouts to S17, S18 and Supplemental Tables 3-15 and supplemental supporting spectra where they could be useful for understanding and improving clarity in the manuscript

The first half of the Discussion presents a comprehensive genomic overview but feels disconnected from the subsequent experimental results. The manuscript would be stronger if the authors explicitly linked specific insights from the pan-genome analysis (e.g. prevalence of PKS/NRPS classes) to the design and validation of MxPKS. As written, the transition from computational discovery to molecular tool development lacks a unifying conceptual thread that ties the data-driven rationale to experimental innovation.

Along the same line, the discussion currently reads as three loosely connected mini-reviews: Pan-genomic landscape and potential of myxobacteria, development and mechanistic optimization of genome editing tools (MxRedET, TnpB, MxDIRECT), and engineering of the MxPKS chassis and prospects. While each section is individually strong, the transitions between them are abrupt. This disconnect weakens the overall narrative flow of the discussion.

In the discussion, the authors state "We did observe interference from MMEJ during the practical implement of MxDIRECT, which competed with the desired HDR." No evidence is shown for this in the manuscript and thus this is purely speculative here without any data to confirm. This entire paragraph is unnecessary without data.

The exclusive reliance on M. xanthus DK1622 as the engineering host may limit the immediate translatability of this platform to another myxobacterial genera; this limitation should be acknowledged and discussed further in the context of cross-species applicability.

Again in Methods, there is no specific information on where the recombinases were integrated in the genome

Reviewer #2

(Remarks to the Author)
In this work, Zhang, Hu, and colleagues report a genetic toolkit and new heterologous expression host for myxobacteria. Myxobacteria are prevalent producers of secondary metabolites but can be considered as underutilized due to several reasons including lack of robust genetic tools and systems for heterologous expression. Here, a pair of Myxococcus-derived recombinases MxRedET, galK counterselection, Cre/loxP, and transposase TnpB are combined to create the MxDIRECT tool. They used MxDIRECT to generate a new MxPKS chassis from Myxococcus xanthous DK1622 and showed successful heterologous expression of PKS genes/pathways. This is a substantial amount of work, in fact, I find that this manuscript is way too long. It feels like two different papers smashed into one: development of MxRedET and creation of MxPKS chassis with test heterologous expression of PKSs that are homologous to other known ones. >100 references for one research paper is a lot! Also, I don't think I've ever seen a discussion section after the conclusion. Overall, the advancement of this system is important for the field of myxobacteria, but this study feels too specialized for Nature Communications.

1. While a comprehensive BGC atlas for myxobacteria may not be available yet, other groups have analyzed and curated BGCs from myxobacterial genomes. These should be included and cited. https://doi.org/10.1093/database/baae056; https://doi.org/10.1101/2025.06.19.660557 (this is cited in the post-conclusion discussion, rather than in the section on BGCs in results); 10.3390/microorganisms7060181

2. Fig. 2e: the data point at 100 bp looks like an outlier?

3. Fig. 5: Please label the chromatograms in 5b with compound numbers, and provide the wavelength and y-axis scale used to detect the compounds. Several stereocenters are missing in the structures in 5c. If these stereocenters are not known for new compounds, they should be determined.

4. Please report yields of all compounds. Only the coralinones (2 mg/L) were given, as well as isolation yield of 14 (1.5 mg from 30 L) and 15 (0.8 mg from 30 L).

5. Confirmatory NMR and/or MS should be provided for identification of compounds 1–3, 10, and 11.

6. SI de novo structural elucidation: Similar TLC staining does not indicate structurally similar compounds.

7. Calculated masses for expected molecular formulas should be provided for all compounds for simple comparison with HRESIMS data. For some, mass errors are higher than expected (compound 12 = 16 ppm; compound 14 = 69 ppm)

8. The purity of several compounds is not high enough for new natural products: particularly compounds 6 and 9

9. Raw and/or processed spectroscopy data for new, pure, compounds should be uploaded to a publicly available repository.

10. Some typos:
-Extra "and" on page 2: Bacillus, and Escherichia, and Aspergillus
-"sparely" and "worthy" on page 3
-"dervied", "constrain", and "jointed" on page 5

Reviewer #3

(Remarks to the Author)
I co-reviewed this manuscript with one of the reviewers who provided the listed reports. This is part of the Nature Communications initiative to facilitate training in peer review and to provide appropriate recognition for Early Career Researchers who co-review manuscripts.

Reviewer #4

(Remarks to the Author)
The Myxococcocota represent an ecologically important phylum of soil-dwelling bacterial predators with long-recognized potential for secondary-metabolite production. However, this potential has not been explored to its fullest, largely because only a very small number of model species can be cultivated in the laboratory and genetically manipulated. In practice, most functional and genetic studies have focused exclusively on Myxococcus xanthus, for which decades of research have produced an extensive biological toolkit. Culturing other members of the phylum and manipulating them genetically has been hindered by their complex social lifestyles—particularly their strong aggregative behaviour and the production of a dense extracellular polysaccharide (EPS) matrix. Moreover, these organisms grow slowly, which further complicates genetic analysis.

In this context, using a well-established model organism—Myxococcus xanthus strain DK1622—is a sound approach and

provides opportunities to reconstruct entire biosynthetic pathways heterologously. Such a strategy enables the exploration of the vast diversity of secondary metabolites encoded by wild strains, as well as the investigation of other biological processes. To date, however, these efforts have been limited by the availability of genetic tools for constructing robust "chassis" strains.
This study presents a highly sophisticated and comprehensive approach to overcoming these limitations. The authors first demonstrate that overexpression of an endogenous Mx-RedET recombination system enables rapid, targeted gene deletions through the introduction of linear DNA fragments. They further combine this system with Cre–Lox to excise resistance markers, and they show that TnpB-mediated cleavage can subsequently remove all remaining chromosomal scars. This final technology, which they refer to as MsDIRECT, supports the generation of multiple, very large deletions as well as the insertion of large foreign gene clusters.

The authors then show that this platform enables the production of new metabolites via heterologous expression of both characterized biosynthetic gene clusters (BGCs) and predicted BGCs with previously unknown products. Their extended bioinformatic analyses provide an updated overview of the diversity and potential of these systems. Overall, this work represents a major advance in synthetic biology and significantly expands our capacity to explore the large—and still largely mysterious—repertoire of secondary metabolites and other biological processes within this important bacterial phylum.

Suggested revisions to improve clarity and precision:

The text is written in highly technical terms, which is appropriate given the sophistication of the genetic systems involved, but it is occasionally dense. Some terms could be better defined for accessibility—e.g., explicitly introducing ISDra2 as a transposase, and explaining in clearer terms how TnpB-based systems function mechanistically.

The MxPKS chassis strain is genetically very sophisticated: it combines deletions of key genes (motility, development, cell lysis), removal of endogenous BGCs, expression of efflux pumps, and introduction of a malonyl-CoA precursor pathway to enhance heterologous production. However, the manuscript does not convincingly demonstrate how these modifications improve performance relative to the parental strain. in particular:
– Does the introduction of the malonyl-CoA precursor pathway measurably increase the intracellular malonyl-CoA pool, and does this demonstrably enhance metabolite production?
– Are the introduced efflux pumps effective at preventing the accumulation of toxic compounds, and are there associated fitness costs?
These questions are important, as the chassis strain is presented as a foundational resource for future studies.

Although the development of the MxPKS chassis is a major advance for secondary-metabolite studies, the genetic tools described also have broad potential for more general functional analyses. It would be valuable to discuss the limitations of these systems for such applications.
– Does overexpression of Mx-RedET have physiological side effects?
– Can the introduced mutations or modifications be readily transferred into an otherwise wild-type background?
– The curing of the ISDra2 expression plasmid seems straightforward, but are there circumstances where this may be problematic?

A minor point: while the MxPKS chassis is clearly novel, it is not the first chassis proposed for myxobacteria. Sourice et al. recently published a strain combining high expression capacity with deletion of endogenous BGCs as a chassis, and this should be acknowledged for completeness.

Version 1:

Reviewer comments:

Reviewer #1

(Remarks to the Author)
While changes were made in content that did improve the data presentation and description for this study, the manuscript remains difficult to read in areas, with words used out of context and often changing the desired meaning. The manuscript would greatly benefit from thorough revision by a technical writer or a careful language edit to improve clarity, flow, and precision—particularly in the Introduction section, which still requires substantial refinement. There also continues to be some concerns on data presented for editing efficiencies that could be improved by additional testing in at least one more locus (which could be available, but the manuscript lacks the clarity to determine if this is the case). These concerns should be addressed before the manuscript is in a state suitable for publication in Nature Communications. Specific points to address follow.
- The whole manuscript, but especially the introduction, contain several examples of unnecessarily embellished language and unconventional word choices that reduce clarity and detract from the scientific tone. Overall, the text would benefit from being simplified and made more direct. For example, phrases such as "superlative capacity," "exquisite mode of action," and "voluminous untapped biosynthetic gene clusters" could be replaced with more neutral wording. Similarly, some expressions are unusual or awkward in a technical context, such as "clique of life," "stubborn cultivability," "bacteria insubordinate in genetic manipulation;" standard terminology would improve readability. For example, "bacteria insubordinate in genetic manipulation" should be replaced with a conventional term such as "genetically intractable bacteria" and "gifted strains" should be replaced with "promising strains".

- In reference to the sentence “Given that native recombinase pairs have been found in E. coli (17–19) Burkholderia (35) and Bacillus (36) which have demonstrated high efficiency in their respective hosts,”—the studies for E. coli cited (17-19) are Lambda Red phage machinery studies using the prophage-derived recombineering systems, not E. coli, it would be more appropriate here to cite https://doi.org/10.1073/pnas.91.8.3205 that describes the E. coli RecT homolog if they used that sequence to search (it is not clear from the manuscript); either way the authors need to correct and clarify this sentence to elaborate that although the common systems utilized are from phage, there are systems present in bacterial genomes that have been harnessed for recombineering, and specify which they used to determine the homologs chosen in Myxobacteria.

- In testing these systems, the authors also seem to use the canonical phage-derived exo beta and recET systems in Figure 2b (as described above, they are not from E. coli, this should also be corrected in the Figure 2 legend), but there is no mention of this in the text. If the authors aim to emphasize rationale of the experiments looking for RecET homologs versus using the phage -derived systems often used, this could easily be stated before going into that data.

- In reference to the sentence “We proceeded to refine the experimental optimal parameters for MxRecET, by setting the length of HR to 100 bp, using 1200 ng of exogenous donor DNA, and extending the recovery time of post-electroporation to10 hours (Figure 2c–e2e).” This is awkwardly stated and before the findings that led to these choices. It would be more appropriate to state “We evaluated parameters including homology arm length (2e), amount of DNA (2d), and recovery time post transformation (2c) to determine optimal conditions” and lead into the data that led to the choices of 100 bp homology arms, 1200 ng DNA, and 10 hour recovery. That said, there are still issues that remain with the authors just stating that in all conditions that % edited is 100% here and only showing CFUs, as this would be highly unusual, especially with the very short homology arms. For example, homology arm limitations in many bacteria, even with recombineering systems, is known in the literature. Therefore, the authors really should be showing both CFU and editing here to justify the optimized protocol choices, and further editing of this section is needed to more clearly outline experimental design. In addition, there are continued concerns for the lower CFUs/mL with the larger arm length, and the drop in between 120-200 and from 100-80 bp in Figure 2e. This is not consistent with typical trends in bacterial editing efficiencies, and as the authors stated in the rebuttal and edited text, could be contextual on a number of reasons, but many of the conditions (e.g. electroporation, DNA amount) should not fluctuate to that large of an extent if you are performing experiments properly. While any specific mechanisms are beyond the scope, the authors are presenting this work as a general toolkit, and variation needs to be at least in part accountable, especially when so outside of expected trends, and in this case is potentially chromosomal context, which is often required in these types of studies to test the system for at least one other locus, especially with the data presented that is very skewed to only 100 bp arms (see https://doi.org/10.1021/acssynbio.5b00187 as an example). The same is also a concern for Figure 2F, another locus should really be shown as well to explore any contextual considerations due to locus.

- The paragraph on Cre-Lox marker removal can be shortened with less detail (covered in Supplemental Figures) and just emphasized as needed to recycle the marker for subsequent rounds of editing.

- “Followingly” should be replaced by “ Subsequently”, “In Brief” with “Briefly”.

- In the sentence “A continuous fragment (~0.5 kb) of the myxovirescin BGC was targeted for scarless deletion (Figure S9).”, what is the rationale for selecting a ~0.5 kb fragment of the myxovirescin BGC for deletion? This is not explained, was this region chosen because it is non-essential, representative of typical editing targets, or part of a functional domain?

- The abbreviation “HR length” may cause confusion because HR often refers to homologous recombination itself rather than homology arm length. It may be clearer to write “homology arm length” or abbreviate “HA.”

- In the text removed, the abbreviation Repair template (RT) was also removed, so make sure you re-introduce that at first appearance.

- When pointing out the homology arm size discrepancy between MxRecET alone and with ISDra2, this is also a point where locus context is important, and the authors should be clear on locus targeted and adding additional loci to show consistency of the method as discussed previously (Figure 3d). Also, it is not directly clear if the same loci was targeted with both systems? The locus targeted for every experiment should be consistent and clearly stated in the text if you are comparing outputs.

- In this same paragraph, the statement that the ISDra2–MxRecET system “exhibited behavior distinct from MxRecET alone” may be somewhat overstated. The observed differences in colony formation across homology arm lengths could potentially arise from the additional selection pressure imposed by ISDra2-induced double-strand breaks rather than from intrinsic mechanistic differences in the recombination process itself. Therefore, the authors may wish to moderate this interpretation and clarify that the observed differences likely reflect the requirements for DSB repair under counterselection conditions rather than fundamentally distinct recombination behavior of the MxRecET system.

- While Figure 4d shows improvement for a single, 2 step experiment, this could vary for other experiments and should at least be emphasized that further troubleshooting will be necessary to optimize this for more generalized use.

- What supplemental data do you mean, clarify: “De novo structural elucidation (see supplementary data) revealed that these compounds arise from double-bond rearrangements or oxidation/reduction of the parental conjugated allene, thereby

significantly expanding the chemical diversity of this structural family."

- The first 2 paragraphs of the edited Discussion are still disjointed from the larger findings and lack clear justification and impact, with presentation of the tools developed without the broader context. There should be a clear overview on the broader impact of the study, the impact of the developed tools over state of the art, and an overview of limitations to address further in future studies.

- In the discussion, the statement that the biosynthetic potential of myxobacteria is "critically stymied by the absence of robust and universal genetic manipulation tools" may be somewhat overstated. While genetic tools are indeed limited for many myxobacteria, several strategies (e.g., homologous recombination, transposon mutagenesis, and heterologous expression platforms) have been successfully applied. The authors may consider moderating the statement or acknowledging existing approaches while emphasizing their limitations.

- The section discussing chassis engineering priorities (morphology, autolysis, restriction-modification systems) is insightful but reads somewhat disconnected from the experimental work presented. The authors may wish to more explicitly link these strategies to the capabilities enabled by the MxDIRECT system.

Reviewer #2

(Remarks to the Author)
The authors significantly revised the manuscript, and most concerns have been addressed. A few that still need answering:

1. Fig. 5: Compounds 4–8 are not labeled in these chromatograms. The authors have standards that can be now run to determine which peaks are those compounds.
2. Cpd 2 is still missing a stereocenter.
3. There is not clarity for how all the stereocenters and alkene configurations were determined for compounds 4–6. The authors state that they compared chemical shifts and coupling constants with that of cpd 3, but the authors do not provide coupling constants for all carbons with stereocenters. And in the macrocycle, given its presumed flexibility, are coupling constants adequate for determination at C16 and C17? The 10,11 and 12,13 alkenes in cpd 4 and the 11,12 alkene in cpd 5 does not have spectroscopic support (NOE, coupling constants) or text description for their E/Z designations.
4. SI page 34 has a section titled "SUPPORTING SPECTRA OF NEW COMPOUNDS" but many of these are known compounds.

Reviewer #3

(Remarks to the Author)
I co-reviewed this manuscript with one of the reviewers who provided the listed reports. This is part of the Nature Communications initiative to facilitate training in peer review and to provide appropriate recognition for Early Career Researchers who co-review manuscripts.

Version 2:

Reviewer comments:

Reviewer #1

(Remarks to the Author)
Overall, the reviewer comments were well addressed and the manuscript is much improved overall, both in writing and in data presentation and justification. One very minor comment remains that affects the broader readership for the journal. While the clarity has improved, word choices remain in the introduction that could be simplified, including "fraught with challenges", "thwarted", "attractive vehicle", "ineluctably teeming with obstacles"... Similarly, a few terms in the Discussion feel somewhat more formal or stylistically elevated compared to the straightforward tone of the rest of the manuscript, such as "protracted timelines," "circumventing," "obviates," "enticing opportunity" . These are not incorrect but simplifying them could further improve readability and maintain a more consistent tone.

Reviewer #2

(Remarks to the Author)
The authors addressed all my concerns.

Open Access This Peer Review File is licensed under a Creative Commons Attribution 4.0 International License, which permits use, sharing, adaptation, distribution and reproduction in any medium or format, as long as you give appropriate credit to the original author(s) and the source, provide a link to the Creative Commons license, and indicate if changes were made.
In cases where reviewers are anonymous, credit should be given to 'Anonymous Referee' and the source.
The images or other third party material in this Peer Review File are included in the article's Creative Commons license, unless indicated otherwise in a credit line to the material. If material is not included in the article's Creative Commons license and your intended use is not permitted by statutory regulation or exceeds the permitted use, you will need to obtain permission directly from the copyright holder.
To view a copy of this license, visit https://creativecommons.org/licenses/by/4.0/

Reviewer's Comments:

Reviewer #1 (Remarks to the Author)

This manuscript reports the development of a genetic toolkit in Myxococcus xanthus, to unlock the large potential of myxobacterial secondary metabolites. Myxobacteria are prolific producers of bioactive natural products with complex structures and potent biological activities. However, the discovery and production of new compounds are hindered by difficulties in culturing and genetically manipulating non-model myxobacteria. This work builds on previous genetic tool development (e.g. HR, galK counterselection, φC31-mediated site-specific integration, cre-loxP, pMiniHimar mariner-transposon) by this group, employing a RecET recombineering system identified from other myxobacteria (MxRedET), and the TnpB system (specifically ISDra2) to enable markerless deletions up to 200 kb, duplex editing, and SNP generation. They use these new and existing tools to engineer a chassis strain of Myxococcus xanthus DK1622, MxPKS (derived from a previously engineered Hu04 strain) to evaluate heterologous expression of BGCs resulting in discovery of novel polyketides. While there are some promising novel findings here, it is often hard to determine the impact due to missing data, teasing out relevant new versus previous genetic tools for the ultimate effort of designing the chassis to evaluate known BGCs for function and elucidate novel BGCs or lack of clear rationale for experimental design. To this end, the manuscript reads almost like 2 separate papers—a comprehensive search of BGCs across myxobacteria and tool development—the tool development beyond the recombineering machinery and the TnpB editing not directly relevant to the construction of MxPKD (e.g. the Cre-loxP work) should be greatly reduced in scope or removed. In addition, there are several instances of awkward phrasing and grammatical errors throughout the text, and the manuscript would benefit from careful editorial revision to improve clarity and readability. There are also many opportunities to edit the text to better describe and confer rationale, experimental design, and interpretation of results to a more general audience. Therefore, I would only support publication after a major revision.

>>> We sincerely thank the reviewer for the careful, constructive, and insightful comments on our work. We apologize that the logical connection between the systematic bioinformatic analysis and the downstream development of genetic tools and chassis cell construction was not sufficiently articulated in the original manuscript, which may have given the impression that the study consists of two loosely connected parts.

In fact, our intention was to present an integrated blueprint for the genome mining of myxobacteria (as the title states). Through comprehensive bioinformatic analyses, we demonstrate the vast, largely unexplored biosynthetic potential ("dark matter") of myxobacterial natural products, particularly highlighting the prevalence of PKS-type biosynthetic gene clusters. This systematic insight directly underscores the necessity and urgency of establishing an efficient heterologous expression chassis, especially tailored for PKS pathways. Importantly, the intensive construction of such chassis cells inevitably relies on the development of robust and efficient genetic engineering tools. From this perspective, the different components of this work form an organic and logically connected whole,

collectively advancing the exploitation of myxobacteria as an exceptionally important yet still underdeveloped resource for natural product discovery. To further clarify this integrated research logic, we have revised the ABSTRACT and the final paragraph of the INTRODUCTION to explicitly outline the systematic rationale and overall framework of the study.

Although recombineering combined with Cre/loxP site-specific recombination has been reported in other microorganisms, its application in myxobacteria has not previously been demonstrated. We believe that reporting this strategy in myxobacteria is therefore valuable, as it provides the community with an alternative genetic tool.

In response to the reviewer's suggestion, we have substantially condensed both the bioinformatic analysis and the Cre/loxP-related sections, and we have strengthened cross-references between sections throughout the revised manuscript. These changes are intended to improve coherence and emphasize the integrated encouraging of the study.

Some specific points that need to be addressed are here:
Red/ET should have the "/" as they are distinct recombineering systems (Lambda Red, RecET) if using that terminology; Red/ET is not generally common in the broader field, its usually just referred to as recombineering. Also ensure that Redα is also referred as "exo" and Redβ as "beta" as these are the more commonly used gene names in the broader field. Also, for consistency, ensure that "RedET" consistently refers to the same system; currently, it alternates between the E. coli and myxobacterial systems which makes the context confusing.

>>> We sincerely thank the reviewer for this important clarification. We were unaware of the distinction and have now standardized the terminology throughout the manuscript. "Red/ET recombineering " is used when referring to the general approach, and "RecET" is consistently used to denote the specific recombinase system, following standard nomenclature. According to the more commonly use, Red α is now referred to as "exo" and Red β as "beta". These revisions have been applied uniformly across the text, figures, and supplementary materials to eliminate any ambiguity.

When stating "In addition, Red/ET recombineering excels over RecA-dependent HR by requiring only short homology arms (40–100 bp), simply crafted through PCR" it should be stated that this is known for E. coli, as longer arms are often still needed in other bacteria

>>> This sentence has been deleted from the revised manuscript.

The citation #26 is not correct for this statement: "For instance, its combination with Cre/loxP facilitates complex, markerless manipulations"

>>>This sentence has been deleted from the revised manuscript.

The statement "Its pairing with CRISPR/Cas9-mediated double-strand breaks dramatically enhances the efficiency of targeted gene replacement." Is missing seminal references for

E. coli (e.g. https://doi-org.ornl.idm.oclc.org/10.1038/srep15096, https://doi.org/10.1021/acssynbio.5b00187)
>>>The two papers recommended by the reviewer 1 have been cited.

The authors should include description of the broader classification of the TnpB as well as introduction of the system they chose to deploy (ISDra2) and why instead of CRISPR-Cas9 in the introduction and not just the discussion as rationale for the study. In the Introduction, the justification for adopting TnpB instead of the well-established CRISPR/Cas platforms is not so clear. The authors mention its small size and specificity but could clarify why these features are particularly advantageous in M. xanthus.
>>> We appreciate the reviewer's suggestion and have substantially expanded the Introduction to provide a comprehensive classification of TnpB. We now include a detailed overview of TnpB as a compact RNA-guided nuclease within the IS200/IS605 transposon superfamily, distinct from CRISPR-Cas systems. The reason why TnpB was chosen over CRISPR-Cas9: (1) our previous attempts with Cas9 in *M. xanthus* DK1622 revealed significant off-target effects and cellular toxicity; (2) The small size of TnpB (~400 aa vs. ~1,600 aa for Cas9) is particularly advantageous for genetic tool development, facilitating delivery via plasmids or phage vectors—essential for notoriously difficult-to-manipulate non-model myxobacteria; (3) Furthermore, the synergistic combination of Red/ET recombineering with TnpB cleavage represents a new paradigm for precision genome editing that extends beyond more common CRISPR-based approaches.

In the introduction, the authors imply that morphological improvements of DK1622 (such as dispersed growth and single-colony formation) and the removal of specific BGCs inherently translate to better heterologous expression outcomes. This assumption lacks further explanation. The authors should provide a logical explanation connecting these phenotypic traits to enhanced biosynthetic performance.
>>> We have re-organized the INTRODUCTION section, and moved the content about morphological improvements into the section of "Construction of the chassis cell MxPKS".

For brief explanation: dispersed growth and single-colony formation are beneficial for the fermentation and genetic manipulation of chassis cell for heterologous expression. Knockout of specific BGCs that compete with the target pathway for key precursors, such as acetyl-CoA and malonyl-CoA, is useful to simplify the complex metabolic network and ensure sufficient resource availability for efficient synthesis of the target heterologous product. We have clarified these points in the section "Construction of the chassis cell MxPKS" of revised manuscript.

“It was advisable to exclude these two families as priority for future genome mining endeavors” sounds like a subjective statement. Can the authors provide clarification?
>>> This subjective statement has been deleted.

The authors state “The resulting constructs were separately integrated at the Mx8 site of

the M. xanthus DK1622 genome." but it appears from the text and Figure S3 that there is only 1 construct? It is also missing in the text how and where this construct is integrated. It appears from Figure S3 its using the φC31-mediated site-specific integration referenced later based on the attP-attB sites shown? This all needs to be described here.
>>> The *recET* was inserted into the *attB1* site of the tandem $tRNA^{Asp}$ gene *trnD1* in DK1622 genome through the Mx8 site-specific integration system. The integration method and location are detailed in the updated Figure S3. We now clearly specify it in the main text and method of revised manuscript.

It is unclear why a medium-strength constitutive promoter was selected. Given that recombineering genes are often toxic when constitutively expressed, many bacterial systems instead employ transient or inducible expression strategies. The authors should therefore assess and report any effects on cell fitness and potential changes in mutation frequency to provide a more comprehensive evaluation.
>>> Currently, the inducible promoters available in myxobacteria, such as the vanillate promoter (P_{Van}), mostly exhibit significant leaky expression, which often leads to persistent DNA toxicity. Our preliminary experiment indicated that driving MxRecET with a strong promoter is lethal. Therefore, we chose a medium-strength constitutive promoter rrnD5, which balances the recombination efficiency and metabolic burden.

Following the reviewer's suggestions, we further evaluated the effects of the constitutive expression of MxRecET on the cell fitness of the strains, and found that there were no significant differences in growth rate, transformation efficiency or mutation frequency between the engineered strain and the wild-type strain (updated Figure S3). This indicates that the selection of the medium-strength constitutive promoter is relatively appropriate.

Figure 2 includes multiple statistical significance annotations (e.g., P values and asterisks), but the caption does not specify which statistical tests were used to generate these values. The authors should clearly state the statistical test applied. This clarification should also be extended to all other figures where statistical comparisons are presented.
>>> We have updated the figure legends for Figure 2 and all other figures involving statistical comparisons to explicitly specify the statistical tests employed, such as the two-tailed Student's t-test for two groups, Šidák's test for no less than three groups and so on.

Figure 2a, "yqaJ" is the label for what I assume is the recE homolog, the authors should explain this in the text or change to recE. The caption refers to "RecE," whereas the figure itself labels it as "yqaJ." Please standardize the nomenclature throughout for clarity. Additionally, it is unclear what the white arrows in panel (a) represent are these hypothetical or uncharacterized neighboring genes?
>>> We have standardized the gene or protein of *recE* homolog nomenclature to *recE* or RecE throughout the main text, figures and supplementary materials, and have removed the confusing yqaJ label. In addition, we clearly stated in the legend of Figure 2a that the

white arrows represent genes encoding hypothetical proteins.

The authors state that they use “flanking homology arms as short as 60 bp” but do not specify what the length of the HA sequence is for Figure 2a

>>> We used 500 bp homology arm for Figure 2a, and specify it in the main text now.

Figure 2b, the red bar should be labeled fraction edited, and the y-axes needs to be labeled accordingly

>>>We have revised the red bar label and y-axis of Figure 2b.

Figures 2 c-f, the authors state they are showing recombination efficiency but are only showing CFU/ml, which does not necessarily equate with editing and must be verified by PCR with a statistically relevant number of colonies. More discussion is needed before you discuss the experimental parameters for better understanding of what was gleaned from these data as well. For example, why is there a substantial drop off after 200 bp, but a jump at 100 bp (c)? Why a substantial jump at 10 h from 8 h (d)? 1000ng DNA to 1200 ng DNA (e)? Drop in efficiency for 60-100 kb deletion but back up from 160-180, and then the huge drop at 200 (f)? None of these make sense empirically without seeing the editing efficiencies on the graphs, and all the data could be strengthened by comparing with native recombination machinery as a control

>>> Figures 2c-f actually show the colony-forming units per milliliter (CFU/mL) in different edited conditions. We randomly selected 24 colonies each condition with PCR and sequencing verification, and found that the editing accuracy of all selected colonies is 100%. Therefore, CFU/mL serves as a reliable indicator of recombination efficiency.

Regarding the observed fluctuations in CFU/mL, we performed strict parallel experiments and report the data as obtained to get the optimal parameters for MxRecET. While we share the reviewer's curiosity about the underlying mechanisms—for instance, the drop after 200 bp homology arms, the peak at 100 bp, or the variations in DNA mass and time course—these phenomena likely reflect complex interplay between transformation efficiency, DNA repair dynamics, and genomic context. Future mechanistic dissection is beyond the scope of this study.

We respect the reviewer's suggestion, and have added a brief discussion, as following: *“Notably, 24 colonies were randomly selected for PCR and sequencing verification across all experimental conditions, with all validated colonies demonstrating 100% editing accuracy. These findings indicate that MxRecET-mediated homologous recombination in DK1622 is efficient once linear DNA enters the cell, suggesting that transformation efficiency—rather than recombination itself—represents the primary bottleneck. The observed variations in CFU/mL likely reflect the complex, interdependent influence on DNA uptake and cellular viability, including homology arm sequence composition and length, DNA mass, electroporation conditions, and the specific genomic context of the target locus. For instance, shorter homology arms (e.g., 100 bp) may balance between transformation efficiency and recombination substrate availability. Similarly, fluctuations in DNA mass or*

recovery time may affect cellular stress responses or DNA stability, and target site accessibility may vary with local chromatin structure or transcriptional activity."

Figure 2g-h, this is really just a 2-step tandem transformation method, not technically duplexed. Also in (h) it would be useful to see CFU and targeting other sites with different types of mutations as ~27% may not be either a "standard" percent depending on mutation and loci and may thus not be sufficient to use this method for all combinations to get double transformants, saving no time over just doing subsequent transformations. It is also perplexing again at the jump from 2-4 h when that wasn't seen for the single mutants in (c)

>>> Thanks for your precise correction on the terminology. We have accordingly revised the manuscript by replacing "duplexed editing" with "tandem editing" throughout the text. We also supplemented the editing efficiency data on different types of mutation and more dual-locus editing combinations (updated Figure 2h and 2i). Compared with the conventional iterative approach, our tandem editing strategy shortens the total experimental timeline for two clean deletions by approximately 50%, greatly improving efficiency.

Single-locus editing (Figure 2c) involves only one post-electroporation recovery step, whereas tandem editing (Figure 2h) includes an additional antibiotic-enrichment recovery step following the initial short recovery (2-6 h). The distinct workflows account for the different rules between these two approaches.

For the marker recycle using Cre-loxP, the recombineering machinery isn't necessary for this, this really isn't novel at all and couldn't be used for any edits where the cre-loxP scar would impede, e.g. base editing, need to better justify novelty versus native system) a comparison could help)

>>> We fully agree with the reviewer that recombineering itself is independent of Cre/loxP. However, we employed the Cre/loxP system specifically to efficiently excise resistance markers following MxRecET-mediated recombination, streamlining the generation of scarless mutants. While this combination is well established in model organisms such as *E. coli*, it had not been previously demonstrated in myxobacteria. We also very respect the reviewer's suggestion and have condensed the Cre/loxP section, de-emphasizing its novelty and integrating it into the second part of the RESULTS as a complementary tool rather than a standalone advance.

Figure S5 and S6, the design of the experiment should be more clearly stated to understand the results in the text. This isn't at all in the figure and isn't helpful in knowing what the expected size of the WT versus integrated construct would be

>>> We supplemented the schematic diagrams in Figures S5 and S6 to illustrate the expected size of successful mutation.

Figure 3a isn't necessarily correct in the lower half of the figure and description in the text, the edit introduced by the repair template is removing the target sequence for the

TnpB/reRNA complex to bind and cut, protecting the edited sequence from cutting if repaired

>>> We corrected the lower half of the Figure 3a and updated the description in the manuscript as following: "*the MxRecET system utilizes a donor DNA fragment as a template for homology-directed repair and eliminates the original target cut site recognized by the TnpB/reRNA complex.*"

For Figure S7, it is stated that the reRNA is also on the construct, is it a non-targeting sequence as it is not causing cell growth defects? This needs to be better explained

>>> The construct contains the reRNA element, whose 20 bp guide sequence specifically targets the exogenous apramycin resistance gene (designed for subsequent plasmid interference assay). We added a schematic diagram to explicitly illustrate plasmid interference assay (updated Figure S7).

For Figure 3b, where is the reRNA targeting? Important to include

>>> The target of ISDra2-reRNA is on the apramycin resistance gene of the second plasmid, as illustrated in Figure S7 of the revised supplementary data file. We have also clearly clarified this in the revised manuscript.

For Figure 3c, the authors need to more clearly explain the mutation you are introducing and that this is WITHOUT selection, understanding may be improved by a schematic

>>> A continuous fragment (~0.5 kb) of the myxovirescin BGC was targeted for scarless deletion without the usage of selection marker. We have added a new schematic as Figure S9 to explain this mutation.

Figure 3d, the authors need to show editing efficiency alongside CFUs and this is not indicative of no effect of HA length (and their conclusion that the DSB is driving repair is not consistent with results from 1e that aren't noticeably statistically different), the authors need to more clearly address experimentally

Figure 3e needs statistical significance applied and editing % included, this does not clearly show that DNA ratio is a significant driver as stated in the text

>>> We provided the editing efficiency of Figure 3d and Figure 3e in Figure S10. As the editing efficiency approached 100% under all conditions tested, we used CFU/mL as a direct metric to distinguish the impact of HA length and DNA ratio. The statistical significance has been also added in the revised Figure 3e.

The optimal HA length differs between the marker-dependent editing system (MxRecET alone) and the marker-free editing system (ISDra2-MxRecET). MxRecET alone enriches positive colonies only through the selectable marker *apra*^{R}, without relying on double-strand break (DSB), whereas the ISDra2-MxRecET combined system achieves marker-free editing via ISDra2-mediated DSB. Owing to the distinct mechanisms and experimental designs of the two systems, their optimal HA lengths differ, and we have clearly stated this in the revised manuscript.

In Figure 3c, we clearly demonstrated that the concurrent presence of ISDra2, MxRecET, and RT is essential for selectable marker-free editing, indicating that the ISDra2-induced DSB is required for this process. In contrast, the experiments in Figure 1e were designed to optimize transformation parameters: varying the DNA mass ratio primarily affected CFU/mL rather than the repair outcome itself. We have revised the main text to avoid overemphasizing the role of DNA ratio.

Figure 4a could be much clearer by delineating the construct and deletion sizes more clearly across the diagram

>>> We have supplemented the size differences of the knockout fragments in Figure 4a and the target positions in figure legend.

Figure 4b-e, the authors now only show the editing efficiency, what was the CFU/ml? These numbers should really consistently be shown across all figures, as well as more tick marks should be added on x-axis to more clearly see representative data, and there should be data on colonies screened included in each legend as well as the actual target region as this can make a difference as to where it is located on the chromosome (e.g. https://doi.org/10.1021/acssynbio.5b00187)

>>> We apologize for any confusion caused by the inconsistent data presentation in the manuscript. To clarify: for condition optimization experiments where the editing efficiency (closely) approached 100%, we presented CFU/mL data to reflect transformation and growth performance (Figures 2c-f and 3d-e); for functional characterization experiments, we focused on editing efficiency, which is the key index for evaluating the function of the system (https://doi.org/10.1038/s41467-024-54191-4). We have carefully unified the data presentation across the entire manuscript according to this principle to ensure consistency.

During the experiments for Figures 4b-e, we screened 24 colonies for each condition to ensure the reliability of the editing efficiency results, and we have supplemented the relevant details in the main text and figure legends. In addition, we have improved the display and layout of both the x-axis and y-axis for better readability, and also supplemented the number of colonies screened under each condition and the chromosomal position of the target site in the figure legends.

Figure S8, you have roman numeral numbers delineating steps in the legend but don't show them in the figure, should be included

>>> We added the roman numeral numbers in the updated Figure S12 and modified the figure legend.

Figure 4d, what were the sizes and locations of the 2 targeted sites? The authors discuss an attempt at expressing 2 ISDra2 nucleases (why do you need 2? Do you mean the reRNAs for targeting? Why not include both reRNAs on the same ISDRa2 vector?) from a single plasmid and introduction of 2 HR templates. What were the sites, and which one

was preferentially edited? If this is included, there should minimally be data included in supplemental.

>>> We have supplemented the detailed information of the two target loci into the main text and figure legend of Figure 4. To achieve simultaneous editing at two distinct sites, two separate ISDra2-reRNA systems are required, each targeting one specific locus. We attempted to co-express both ISDra2-reRNA from a single plasmid with two repair templates, but failed to obtain dual-knockout mutants; screening of 96 colonies revealed editing at only one locus in all cases (data now included as Figure S11). We attribute this to the low probability of delivering three DNA components into one cell, rather than differential targeting efficiency.

For Figure 4e, there should be a more thorough discussion on the tested classes of SNE mutations as well as chromosomal location attempted

>>> We expanded our SNE analysis to include an additional chromosomal locus (MXAN_RS17630) besides the original site (MXAN_RS19070), and the new data has been included as Figure S13. At both loci, we tested mutations at guide positions 1 and 8, revealing significant site-dependent variation in editing efficiency. These findings indicate that SNE efficiency is governed by the combined effects of mutation position, mutation class, and chromosomal microenvironment, and we have expanded our discussion accordingly. And also, we have extensively discussed SNE in the DISCUSSION section.

The authors say 2 weeks to mutant in abstract, but 3 weeks in the text, should resolve

>>> We are sorry for the carelessness and corrected the editing timeframe to two weeks throughout the manuscript.

The authors need to clarify whether the Hu04 strain that the MxPKS was derived from was also the strain that the genetic tools were evaluated in, and the authors should denote in table 1 what mutations were in that starting strain versus the new mutations introduced. If KO7 was the strain you started with to knock out other competing BGCs, where KO5-6 made before or after? Need to clarify

>>> We have clarified in the METHODS section that all genetic tools were evaluated in the wild-type strain DK1622. The order for the genetic manipulation of chassis has been clearly illustrated in the annotation below table, as following: "*KO and IS mean knockout and insertion, respectively. Numbers indicate the sequential order of genetic manipulations from wild-type M. xanthus DK1622. KO1–KO6 represent knockouts completed in M. xanthus Hu04; KO7–KO12 were generated using MxDIRECT in this study; IS13 and IS14 indicate functional element insertions into the chassis strain*."

The authors claim that the HPLC profile of KO12 was "cleaner" than WT, but this should minimally be shown in supplemental if it is included (I see MxPKS in Figure S17, but it is not called out in the manuscript text)

>>> We have now included a direct HPLC-DAD comparison of the DK1622-WT and KO12

strains in Figure S18 of the revised Supplementary data file. As well, the Figure S17 (now Figure S18) is called out in the section of "Construction of the chassis cell MxPKS".

If φC31-mediated site-specific integration and the pMiniHimar mariner-transposon containing plasmid were utilized for integration of BGC components in the MxPKS chassis, why was cre-loxP such a focus in the tools aspect? Clarification of exactly why and how these specific strategies were employed in MxPKS BGC expressing strains as well as whether they were sequenced, etc. is needed. Also, there is very little discussion of the promoter replacements (strength?) in the text, which is also needed to clarify the experimental design.

>>> The cre/loxP system was employed specifically to excise antibiotic resistance markers introduced by MxRecET recombineering, rather than for BGC integration. As stated above, a major objective of this work is to provide the myxobacterial research community with a comprehensive genome editing toolkit. We therefore demonstrate a feasible workflow: MxRecET recombineering followed by marker removal using cre/loxP. While this paradigm is well established in model microbes such as *E. coli*, it has not been experimentally demonstrated in myxobacteria yet.

The φC31 and pMiniHimar were used for the integration of BGCs. We have now clearly delineated these applications in the revised manuscript and apologize for the previous lack of clarity. All constructs were verified by Sanger sequencing, and the resulting sequences have been deposited in NCBI GenBank via BankIt.

We have now specified the promoters used for each construct throughout the manuscript and supplementary data. The relative strengths and characteristics of these promoters (e.g. J23104, J23117, rrnD5, etc.) have been systematically characterized in our previous work (see *ACS Synth. Biol.* 2021, 10, 2904–2909). The promoter replacement of BGCs has been added into Figure 5.

Figure 5a could be more clearly labeled with PKS types and linked to 5b and c, maybe color coding or some other clear delineation, to ensure broader reader understanding. The color coding and abbreviations (KS, AT, KR, etc.) are used without a legend in the figure, this should be explicitly defined, even if standard. It would also help to explain the meaning of the white and gray arrows—presumably non-PKS genes or hypothetical ORFs—but this is not specified in the caption.

>>> We have revised Figure 5a to improve clarity and accessibility for broader readership. PKS types are now directly labeled with text annotations; a comprehensive legend has been added to explicitly define all domain abbreviations, and gene functions are now color-classified.

Figure 5c, While the caption states which are newly identified compounds (4–9 and 12–15), but it would be clearer if these were visually differentiated (e.g., by shading or an asterisk).

>>> In the revised Figure 5c, all the new compounds are depicted in blue and known

compounds are in black.

Missing callouts to S17, S18 and Supplemental Tables 3-15 and supplemental supporting spectra where they could be useful for understanding and improving clarity in the manuscript

>>> All supplementary figures and tables are now called out in the main manuscript or figure legends.

The first half of the Discussion presents a comprehensive genomic overview but feels disconnected from the subsequent experimental results. The manuscript would be stronger if the authors explicitly linked specific insights from the pan-genome analysis (e.g. prevalence of PKS/NRPS classes) to the design and validation of MxPKS. As written, the transition from computational discovery to molecular tool development lacks a unifying conceptual thread that ties the data-driven rationale to experimental innovation.

>>> We appreciate the reviewer's insightful comment. In the revised DISCUSSION, we have integrated the pan-genome bioinformatics analysis with the experimental development of MxPKS to create a unified narrative.

Along the same line, the discussion currently reads as three loosely connected mini-reviews: Pan-genomic landscape and potential of myxobacteria, development and mechanistic optimization of genome editing tools (MxRedET, TnpB, MxDIRECT), and engineering of the MxPKS chassis and prospects. While each section is individually strong, the transitions between them are abrupt. This disconnect weakens the overall narrative flow of the discussion.

>>> We have substantially rewritten the DISCUSSION section.

In the discussion, the authors state "We did observe interference from MMEJ during the practical implement of MxDIRECT, which competed with the desired HDR." No evidence is shown for this in the manuscript and thus this is purely speculative here without any data to confirm. This entire paragraph is unnecessary without data.

>>> We agree with the comment. Since no relevant experimental data is available to support this statement, we have removed the entire paragraph from the DISCUSSION section in the revised manuscript.

The exclusive reliance on M. xanthus DK1622 as the engineering host may limit the immediate translatability of this platform to another myxobacterial genera; this limitation should be acknowledged and discussed further in the context of cross-species applicability.

>>> We fully agree with the reviewer's point. In the revised DISCUSSION, we have added a section addressing the challenges of genetic manipulation in non-model myxobacteria and the importance of developing chassis strains in other genera. We also discuss strategies for adapting MxDIRECT to diverse myxobacterial species, including the identification of endogenous recombineering systems and optimization of transformation

protocols.

Again in Methods, there is no specific information on where the recombinases were integrated in the genome

>>> The RecET recombinases were integrated into *attB1*_Mx8 site within the tandem $tRNA^{Asp}$ gene *trnD1* of *M. xanthus* DK1622 genome via Mx8 site-specific recombination. We supplemented it in the METHODS section.

Reviewer #2 (Remarks to the Author)

In this work, Zhang, Hu, and colleagues report a genetic toolkit and new heterologous expression host for myxobacteria. Myxobacteria are prevalent producers of secondary metabolites but can be considered as underutilized due to several reasons including lack of robust genetic tools and systems for heterologous expression. Here, a pair of Myxococcus-derived recombinases MxRedET, galK counterselection, Cre/loxP, and transposase TnpB are combined to create the MxDIRECT tool. They used MxDIRECT to generate a new MxPKS chassis from Myxococcus xanthous DK1622 and showed successful heterologous expression of PKS genes/pathways. This is a substantial amount of work, in fact, I find that this manuscript is way too long. It feels like two different papers smashed into one: development of MxRedET and creation of MxPKS chassis with test heterologous expression of PKSs that are homologous to other known ones. >100 references for one research paper is a lot! Also, I don't think I've ever seen a discussion section after the conclusion. Overall, the advancement of this system is important for the field of myxobacteria, but this study feels too specialized for Nature Communications.

>>> Many thanks for the Reviewer 2's constructive comments and for recognizing the substantial amount of work embodied in our study.

We would like to clarify that the overarching logic of our work is intended to be a systematic pipeline. Our study begins with a comprehensive bioinformatic analysis to reveal the immense yet untapped biosynthetic potential of myxobacteria. This analysis highlights a critical bottleneck: the lack of efficient chassis cells to unlock this potential. Consequently, developing high-efficiency genetic editing tools (MxDIRECT) was a prerequisite to constructing the MxPKS chassis. Finally, we validated the entire system by heterologously expressing several BGCs. We believe that presenting the "potential," the "tools," and the "application" together provides a complete map for the community. Meanwhile, we fully respect the reviewer's suggestion. In the revised manuscript, we have: (1) Streamlined the text: We have tightened the prose and condensed redundant descriptions to ensure a more concise narrative; (2) Reduced references: We have pruned the reference list to include only the most essential works; (3) Enhanced connectivity: We have rewritten transition sections to better bridge the bioinformatics, tool development, and chassis validation, ensuring the manuscript reads as a cohesive and tightly-knit story.

Regarding suitability for the journal *Nature Communications*, we respectfully highlight that the Myxobacteria constitute an entire bacterial phylum like Actinobacteria as a source of structurally diverse natural products, yet remains severely under-exploited because of

the very genetic intractability that we address here. By providing a universal, portable toolkit (MxDIRECT) and a streamlined chassis (MxPKS), our work lowers the technical barrier for the broader microbiology and natural-product communities and should stimulate research well beyond the current, limited cohort of myxobacterial specialists. We therefore feel that the study matches the journal's mission to publish advances that open new avenues across disciplines.

1. While a comprehensive BGC atlas for myxobacteria may not be available yet, other groups have analyzed and curated BGCs from myxobacterial genomes. These should be included and cited. https://doi.org/10.1093/database/baae056; https://doi.org/10.1101/2025.06.19.660557 (this is cited in the post-conclusion discussion, rather than in the section on BGCs in results); 10.3390/microorganisms7060181

>>> The references recommended by the reviewer have been cited in the introduction and bioinformatics section of the revised manuscript.

2. Fig. 2e: the data point at 100 bp looks like an outlier?

>>> The elevated efficiency at 100 bp was consistently observed in parallel experiments, confirming its robustness rather than representing an experimental outlier. Shorter homology arms (e.g., 100 bp) may balance between transformation efficiency and recombination substrate availability. This phenomenon likely reflects the complex interplay between transformation efficiency, DNA repair dynamics, and genomic context. The mechanistic dissection will be undertaken in future work.

3. Fig. 5: Please label the chromatograms in 5b with compound numbers, and provide the wavelength and y-axis scale used to detect the compounds. Several stereocenters are missing in the structures in 5c. If these stereocenters are not known for new compounds, they should be determined.

>>> We highly appreciate the Reviewer 2's expert comments from the perspective of natural products chemistry. For figure 5b, the chromatograms are now labeled with their corresponding compound numbers, and the detection wavelength and y-axis scale have been clearly indicated. Regarding figure 5c, we have deleted five minor compounds that were obtained in insufficient amounts or have degraded, so that we were unable to clarify the stereochemistry. Actually, the deletion does not lower down the quality of the whole manuscript, since the core objective of this study is the development of the MxDIRECT toolkit and the MxPKS chassis. The remaining major compounds as revealed in Figure 5b provide (even better) proofs-of-concept that the MxPKS chassis can harness four different gene clusters. Throughout the revised manuscript and supplementary data file, the numbering of compounds has been adjusted following the removal of five structures from the series.

All the remaining new compounds have been assigned for stereochemistry. Specifically, the configurations for the new compounds **4**, **5**, and **6** were assigned by

comparing their NMR chemical shifts and coupling constants with those of known compound **3**, whose configuration is known from its published crystal structure (*Org. Lett. 2021, 23, 2114−2119*). The absolute configurations of the new compounds **8** and **9** were determined by comparing their experimental ECD spectra with quantum chemical calculation results, as found in the revised supplementary data file.

4. Please report yields of all compounds. Only the coralinones (2 mg/L) were given, as well as isolation yield of 14 (1.5 mg from 30 L) and 15 (0.8 mg from 30 L).
>>> We have now incorporated the isolation yields for described compounds **1**–**10** within the corresponding sections of the main text.

5. Confirmatory NMR and/or MS should be provided for identification of compounds 1–3, 10, and 11.
>>>We have added the requested confirmatory NMR data for all the known compounds in the section "SUPPORTING SPECTRA OF ALL COMPOUNDS" of Supplementary data file.

6. SI de novo structural elucidation: Similar TLC staining does not indicate structurally similar compounds.
>>> We deleted the sentences about TLC staining for the determination of structural analogue.

7. Calculated masses for expected molecular formulas should be provided for all compounds for simple comparison with HRESIMS data. For some, mass errors are higher than expected (compound 12 = 16 ppm; compound 14 = 69 ppm)
>>> Thanks for the valuable suggestions. In the revised supplementary data file, the calculated mass for expected molecular formulas of each new compound has been added in the de novo structural elucidation.

The carboxylic acid of compound **14** (now compound **10**) has been corrected into an amide group, bringing the proposed structure into line with the molecular formula calculated from the HRESIMS data. Compound **15** was obtained only in trace quantities and prone to decomposition, so that it could not be subjected to further rigorous planar or stereochemical analysis. Therefore, we have deleted it from the revised manuscript.

8. The purity of several compounds is not high enough for new natural products: particularly compounds 6 and 9
>>> We sincerely appreciate the reviewer's professional rigor regarding the structural elucidation of new natural products. During the revision of manuscript, we attempted to re-purify compounds **6** and **9** via semi-preparative HPLC; however, as shown in the HPLC profiling of Figure 5b, compound **3** is the predominant product, and the trace amounts of **6** and **9** resulted in their loss during the purification process. Therefore, we have deleted

them from the manuscript. For the remaining new compounds, their structures have been rigorously confirmed through a combination of HRESIMS and comprehensive 2D NMR analysis, ensuring the scientific integrity of our findings.

9. Raw and/or processed spectroscopy data for new, pure, compounds should be uploaded to a publicly available repository.
>>>We have uploaded the processed spectroscopy data for the new compounds to the figshare repository. This data will be made publicly accessible upon publication of the manuscript.

10. Some typos:
-Extra “and” on page 2: Bacillus, and Escherichia, and Aspergillus
-“sparely” and “worthy” on page 3
-“dervied”, “constrain”, and “jointed” on page 5
>>> The typos have been corrected.

Reviewer #3 (Remarks to the Author)
I co-reviewed this manuscript with one of the reviewers who provided the listed reports. This is part of the Nature Communications initiative to facilitate training in peer review and to provide appropriate recognition for Early Career Researchers who co-review manuscripts.
>>> The authors thank for the Reviewer 3's work to improve our manuscript.

Reviewer #4 (Remarks to the Author):
The Myxococcocota represent an ecologically important phylum of soil-dwelling bacterial predators with long-recognized potential for secondary-metabolite production. However, this potential has not been explored to its fullest, largely because only a very small number of model species can be cultivated in the laboratory and genetically manipulated. In practice, most functional and genetic studies have focused exclusively on Myxococcus xanthus, for which decades of research have produced an extensive biological toolkit. Culturing other members of the phylum and manipulating them genetically has been hindered by their complex social lifestyles—particularly their strong aggregative behaviour and the production of a dense extracellular polysaccharide (EPS) matrix. Moreover, these organisms grow slowly, which further complicates genetic analysis. In this context, using a well-established model organism—Myxococcus xanthus strain DK1622—is a sound approach and provides opportunities to reconstruct entire biosynthetic pathways heterologously. Such a strategy enables the exploration of the vast diversity of secondary metabolites encoded by wild strains, as well as the investigation of other biological processes. To date, however, these efforts have been limited by the availability of genetic tools for constructing robust “chassis” strains. This study presents a highly sophisticated and comprehensive approach to overcoming these limitations. The authors first demonstrate that overexpression of an endogenous Mx-RedET recombination system

enables rapid, targeted gene deletions through the introduction of linear DNA fragments. They further combine this system with Cre–Lox to excise resistance markers, and they show that TnpB-mediated cleavage can subsequently remove all remaining chromosomal scars. This final technology, which they refer to as MsDIRECT, supports the generation of multiple, very large deletions as well as the insertion of large foreign gene clusters. The authors then show that this platform enables the production of new metabolites via heterologous expression of both characterized biosynthetic gene clusters (BGCs) and predicted BGCs with previously unknown products. Their extended bioinformatic analyses provide an updated overview of the diversity and potential of these systems. Overall, this work represents a major advance in synthetic biology and significantly expands our capacity to explore the large—and still largely mysterious—repertoire of secondary metabolites and other biological processes within this important bacterial phylum.
>>> Thank you very much for your positive and insightful comments. We are grateful that you recognize the significance of our work and appreciate your encouragement.

The text is written in highly technical terms, which is appropriate given the sophistication of the genetic systems involved, but it is occasionally dense. Some terms could be better defined for accessibility—e.g., explicitly introducing ISDra2 as a transposase, and explaining in clearer terms how TnpB-based systems function mechanistically.
>>> We have modified the INTRODUCTION section, giving more explanation on the TnpB-based genetic tool.

The MxPKS chassis strain is genetically very sophisticated: it combines deletions of key genes (motility, development, cell lysis), removal of endogenous BGCs, expression of efflux pumps, and introduction of a malonyl-CoA precursor pathway to enhance heterologous production. However, the manuscript does not convincingly demonstrate how these modifications improve performance relative to the parental strain. in particular: Does the introduction of the malonyl-CoA precursor pathway measurably increase the intracellular malonyl-CoA pool, and does this demonstrably enhance metabolite production? Are the introduced efflux pumps effective at preventing the accumulation of toxic compounds, and are there associated fitness costs? These questions are important, as the chassis strain is presented as a foundational resource for future studies.
>>> We greatly appreciate your valuable comments, and have performed additional experiments accordingly. For the NCM pathway, the malonyl-CoA level of the NCM engineered strain (IS14) was 52% higher than that of the wild-type DK1622 (updated Figure S21). The introduce efflux pumps into the chassis cell increased minimum inhibitory concentrations (MICs) against antibiotics representative of different chemical skeletons, indicating the potential to expel toxic compounds from the cell, but without affecting the growth curve (updated Figure S20).

Although the development of the MxPKS chassis is a major advance for secondary-metabolite studies, the genetic tools described also have broad potential for more general

functional analyses. It would be valuable to discuss the limitations of these systems for such applications.

– Does overexpression of Mx-RedET have physiological side effects?

>>> Regarding potential physiological side effects of MxRecET overexpression, we employed the moderate-strength rrnD5 promoter to balance editing efficiency with cellular fitness. As shown in Figure S3 of revised Supplementary data file, MxRecET expression did not cause significant growth defects or elevate spontaneous mutation frequencies under standard laboratory conditions. However, we acknowledge that prolonged or high-level expression may impose metabolic burden, and we have discussed strategies for inducible or transient expression to mitigate such effects in future applications.

– Can the introduced mutations or modifications be readily transferred into an otherwise wild-type background?

>>> As already mentioned in the DISCUSSION section, all genetic modifications were performed in the model strain DK1622. Transfer of these tools and engineered traits to non-model myxobacteria has not yet been attempted and represents an important direction for our future work.

– The curing of the ISDra2 expression plasmid seems straightforward, but are there circumstances where this may be problematic?

>>> After relaxed cultivation in antibiotic-free medium, the plasmid curing efficiency was approximately 75%, with most colonies achieving complete loss of plasmid.

A minor point: while the MxPKS chassis is clearly novel, it is not the first chassis proposed for myxobacteria. Sourice et al. recently published a strain combining high expression capacity with deletion of endogenous BGCs as a chassis, and this should be acknowledged for completeness.

>>> The work by Sourice et al. has been cited (ref. 47) and acknowledged in the section of chassis construction.

Reviewer #1 (Remarks to the Author):

While changes were made in content that did improve the data presentation and description for this study, the manuscript remains difficult to read in areas, with words used out of context and often changing the desired meaning. The manuscript would greatly benefit from thorough revision by a technical writer or a careful language edit to improve clarity, flow, and precision—particularly in the Introduction section, which still requires substantial refinement. There also continues to be some concerns on data presented for editing efficiencies that could be improved by additional testing in at least one more locus (which could be available, but the manuscript lacks the clarity to determine if this is the case). These concerns should be addressed before the manuscript is in a state suitable for publication in Nature Communications. Specific points to address follow.

>>> Many thanks for your valuable comments. We have now professionally edited the entire manuscript to improve clarity and precision via Springer Nature Author Services, especially the Introduction section. All key editing efficiency experiments have been validated at other two independent genomic loci to strengthen the generalizability of our findings.

- The whole manuscript, but especially the introduction, contain several examples of unnecessarily embellished language and unconventional word choices that reduce clarity and detract from the scientific tone. Overall, the text would benefit from being simplified and made more direct. For example, phrases such as "superlative capacity," "exquisite mode of action," and "voluminous untapped biosynthetic gene clusters" could be replaced with more neutral wording. Similarly, some expressions are unusual or awkward in a technical context, such as "clique of life," "stubborn cultivability," "bacteria insubordinate in genetic manipulation;" standard terminology would improve readability. For example, "bacteria insubordinate in genetic manipulation" should be replaced with a conventional term such as "genetically intractable bacteria" and "gifted strains" should be replaced with "promising strains".

>>> We have now thoroughly revised the entire manuscript with special attention to the Introduction section. All unconventional word choices have been replaced with standard, neutral scientific terminology.

- In reference to the sentence "Given that native recombinase pairs have been found in E. coli (17–19) Burkholderia (35) and Bacillus (36) which have demonstrated high efficiency in their respective hosts,"—the studies for E. coli cited (17-19) are Lambda Red phage machinery studies using the prophage-derived recombineering systems, not E. coli, it would be more appropriate here to cite https://doi.org/10.1073/pnas.91.8.3205 that describes the E. coli RecT homolog if they used that sequence to search (it is not clear from the manuscript); either way the authors need to correct and clarify this sentence to

elaborate that although the common systems utilized are from phage, there are systems present in bacterial genomes that have been harnessed for recombineering, and specify which they used to determine the homologs chosen in Myxobacteria.

>>> The *E. coli* RecT protein as independent query probes to search the endogenous recombineering systems in myxobacterial genomes. We have updated the correct reference as recommended by the reviewer.

- In testing these systems, the authors also seem to use the canonical phage-derived exo beta and recET systems in Figure 2b (as described above, they are not from E. coli, this should also be corrected in the Figure 2 legend), but there is no mention of this in the text. If the authors aim to emphasize rationale of the experiments looking for RecET homologs versus using the phage -derived systems often used, this could easily be stated before going into that data.

>>> We have revised the legend of Figure 2b, and added a brief description before introducing the MxRecET system, as follows: "*The E. coli Rac prophage-encoded RecET and the phage-derived λ Red (exo-beta) systems were employed as comparative controls.*"

- In reference to the sentence "We proceeded to refine the experimental optimal parameters for MxRecET, by setting the length of HR to 100 bp, using 1200 ng of exogenous donor DNA, and extending the recovery time of post-electroporation to10 hours (Figure 2c–e2e)." This is awkwardly stated and before the findings that led to these choices. It would be more appropriate to state "We evaluated parameters including homology arm length (2e), amount of DNA (2d), and recovery time post transformation (2c) to determine optimal conditions" and lead into the data that led to the choices of 100 bp homology arms, 1200 ng DNA, and 10 hour recovery. That said, there are still issues that remain with the authors just stating that in all conditions that % edited is 100% here and only showing CFUs, as this would be highly unusual, especially with the very short homology arms. For example, homology arm limitations in many bacteria, even with recombineering systems, is known in the literature. Therefore, the authors really should be showing both CFU and editing here to justify the optimized protocol choices, and further editing of this section is needed to more clearly outline experimental design. In addition, there are continued concerns for the lower CFUs/mL with the larger arm length, and the drop in between 120-200 and from 100-80 bp in Figure 2e. This is not consistent with typical trends in bacterial editing efficiencies, and as the authors stated in the rebuttal and edited text, could be contextual on a number of reasons, but many of the conditions (e.g. electroporation, DNA amount) should not fluctuate to that large of an extent if you are performing experiments properly. While any specific mechanisms are beyond the scope, the authors are presenting this work as a general toolkit, and variation needs to be at least in part accountable, especially when so outside of expected trends, and in this case is potentially chromosomal

context, which is often required in these types of studies to test the system for at least one other locus, especially with the data presented that is very skewed to only 100 bp arms (see https://doi.org/10.1021/acssynbio.5b00187 as an example). The same is also a concern for Figure 2F, another locus should really be shown as well to explore any contextual considerations due to locus.

>>> We greatly appreciate the reviewer's constructive comments, and have substantially revised this section. We have added the editing efficiency data into Figure 2, and supplemented additional two independent loci (Figure S4) to enhance the credibility of the MxRecET optimal parameters. The editing efficiency of MxRecET recombinase is influenced by both operational parameters and genomic context of the target locus.

- The paragraph on Cre-Lox marker removal can be shortened with less detail (covered in Supplemental Figures) and just emphasized as needed to recycle the marker for subsequent rounds of editing.

>>> We have significantly streamlined the paragraph on Cre/loxP.

- "Followingly" should be replaced by " Subsequently", "In Brief" with "Briefly".

>>> Corrected.

- In the sentence "A continuous fragment (~0.5 kb) of the myxovirescin BGC was targeted for scarless deletion (Figure S9).", what is the rationale for selecting a ~0.5 kb fragment of the myxovirescin BGC for deletion? This is not explained, was this region chosen because it is non-essential, representative of typical editing targets, or part of a functional domain?

>>> The ~0.5 kb fragment within the myxovirescin BGC was selected as a representative editing target because this region is non-essential for growth and contains suitable TAM sites for scarless deletion.

- The abbreviation "HR length" may cause confusion because HR often refers to homologous recombination itself rather than homology arm length. It may be clearer to write "homology arm length" or abbreviate "HA."

>>> We have replaced "HR length" with "homology arm length" for clarity throughout the manuscript.

- In the text removed, the abbreviation Repair template (RT) was also removed, so make sure you re-introduce that at first appearance.

>>> The abbreviation "RT(s)" has already been annotated when it first appeared in the manuscript.

- When pointing out the homology arm size discrepancy between MxRecET alone and with ISDra2, this is also a point where locus context is important, and the authors should be clear on locus targeted and adding additional loci to show consistency of the method as discussed previously (Figure 3d). Also, it is not directly clear if the same loci was targeted with both systems? The locus targeted for every experiment should be consistent and clearly stated in the text if you are comparing outputs.

>>> We supplemented the ISDra2–MxRecET system validation at another two independent genomic loci to ensure the consistency and comparability with the results obtained by using MxRecET alone (*mch* locus for Figure 2c－2f and Figure 3d－3e; *ta* and *bgc18* independent loci for Figure S4 and Figure S11). When testing the same locus, the combined system and the individual MxRecET demonstrated similar optimal parameters, with 100－200 bp homology arm lengths. The previously reported differences were more likely attributable to locus-specific variations rather than intrinsic system differences. We apologize for this oversight and have revised the text accordingly.

- In this same paragraph, the statement that the ISDra2–MxRecET system "exhibited behavior distinct from MxRecET alone" may be somewhat overstated. The observed differences in colony formation across homology arm lengths could potentially arise from the additional selection pressure imposed by ISDra2-induced double-strand breaks rather than from intrinsic mechanistic differences in the recombination process itself. Therefore, the authors may wish to moderate this interpretation and clarify that the observed differences likely reflect the requirements for DSB repair under counterselection conditions rather than fundamentally distinct recombination behavior of the MxRecET system.

>>> We fully agree with the reviewer's comments and have removed the exaggerated description in the revised manuscript.

- While Figure 4d shows improvement for a single, 2 step experiment, this could vary for other experiments and should at least be emphasized that further troubleshooting will be necessary to optimize this for more generalized use.

>>> We have supplemented a note as follows: "*Nevertheless, further optimization would be necessary to generalize this two-step electroporation protocol to broader experimental settings.*"

- What supplemental data do you mean, clarify: "De novo structural elucidation (see supplementary data) revealed that these compounds arise from double-bond rearrangements or oxidation/reduction of the parental conjugated allene, thereby significantly expanding the chemical diversity of this structural family."

>>> We have cited Tables S3-S5 for the *de novo* structural elucidation of archangiumide analogs.

- The first 2 paragraphs of the edited Discussion are still disjointed from the larger findings and lack clear justification and impact, with presentation of the tools developed without the broader context. There should be a clear overview on the broader impact of the study, the impact of the developed tools over state of the art, and an overview of limitations to address further in future studies.

>>> We have substantially revised the first two paragraphs of the Discussion to provide a cohesive narrative with clear justification, broader impact, and forward-looking limitations. Specifically, we now open by establishing the longstanding bottleneck in myxobacterial genetics and explicitly contrast the limitations of existing approaches (RecA-dependent recombination, transposon mutagenesis, heterologous expression, and CRISPR/Cas9) with the advantages of our MxRecET platform. We have expanded the impact statement beyond natural product chassis development to include enabling mechanistic dissection of complex myxobacterial social behaviors and facilitating synthetic biology applications such as biocontrol agents and bioproduction chassis, while also articulating that the MxDIRECT architecture offers a generalizable blueprint for genome editing in other genetically recalcitrant microorganisms. We strengthened comparisons to existing methodologies, positioning MxRecET as overcoming the efficiency bottleneck in the field and highlighting MxDIRECT as the first integration of recombineering with TnpB that circumvents Cas9 toxicity, with three specific advantages over standalone recombineering. Finally, we consolidated discussion of limitations for both tools and explicitly linked them to future solutions, including inducible regulation for MxRecET, leveraging additional RecET-like systems for cross-species portability, and sequential strategies for synchronous multiplex editing with MxDIRECT.

- In the discussion, the statement that the biosynthetic potential of myxobacteria is “critically stymied by the absence of robust and universal genetic manipulation tools” may be somewhat overstated. While genetic tools are indeed limited for many myxobacteria, several strategies (e.g., homologous recombination, transposon mutagenesis, and heterologous expression platforms) have been successfully applied. The authors may consider moderating the statement or acknowledging existing approaches while emphasizing their limitations.

>>> We fully agree that the original wording overstated the situation, as several genetic approaches have indeed been successfully applied in myxobacteria. To address this, we have revised the statement to acknowledge existing strategies while emphasizing their limitations. The revised text now reads: "*Previously established genetic manipulation approaches, such as RecA-dependent homologous recombination, transposon mutagenesis, and heterologous expression in surrogate hosts, are constrained by low efficiency, poor adaptability, or protracted timelines, while CRISPR/Cas9 systems exhibit pronounced toxicity and off-target effects in M. xanthus DK1622.*" We believe this revision

provides a more balanced and accurate portrayal of the field.

- The section discussing chassis engineering priorities (morphology, autolysis, restriction-modification systems) is insightful but reads somewhat disconnected from the experimental work presented. The authors may wish to more explicitly link these strategies to the capabilities enabled by the MxDIRECT system.
>>> We have revised the chassis engineering discussion to more explicitly link these strategies to the capabilities enabled by MxDIRECT. Specifically, we now frame this section as highlighting that MxDIRECT makes feasible a range of ongoing chassis optimization efforts. These include eliminating autolysis-related genes to sustain high-density cultivation, disrupting restriction-modification systems to enhance exogenous DNA uptake without accumulating selection markers, engineering morphology for high-throughput handling, and further simplifying metabolic background by deleting BGCs responsible for persistent metabolite accumulation. We also emphasize that NRPS, RiPPs, and terpene BGCs represent comparably abundant yet underexplored reservoirs, presenting necessity for further simplification of metabolic background and/or dedicated chassis development. Collectively, these revisions clarify that while MxPKS addresses critical initial limitations, the construction of a truly superior "plug-and-play" chassis is an ongoing endeavor enabled by the MxDIRECT technology.

Reviewer #2 (Remarks to the Author):

The authors significantly revised the manuscript, and most concerns have been addressed. A few that still need answering:

>>> We highly appreciate this reviewer's valuable comments to our work.

1. Fig. 5: Compounds 4–8 are not labeled in these chromatograms. The authors have standards that can be now run to determine which peaks are those compounds.
>>> Thank you for the comment. Compounds **4**–**6** were obtained only after large-scale fermentation (100 L) and subsequent isolation, yielding very small amounts: **4** (0.5 mg), **5** (0.9 mg), and **6** (0.7 mg). We indeed analyzed the purified standards of compounds **3**–**6** alongside the crude extract of MxPKS-*arc* by HPLC (see attached figure). The retention times of **4** and **6** were very close to that of archangiumide (**3**), and their peak intensities in the crude extract were extremely low due to their low abundance. For compound **5**, no corresponding peak could be detected at the expected retention time, again due to its very low titer. As natural products chemists, we recognize that trace-level compounds are often difficult to detect in crude extracts. Therefore, we did not label **4**–**6** directly on the chromatogram in Figure 5b, as their peaks either co-elute with compound **3** or fall below

the reliable detection limit.

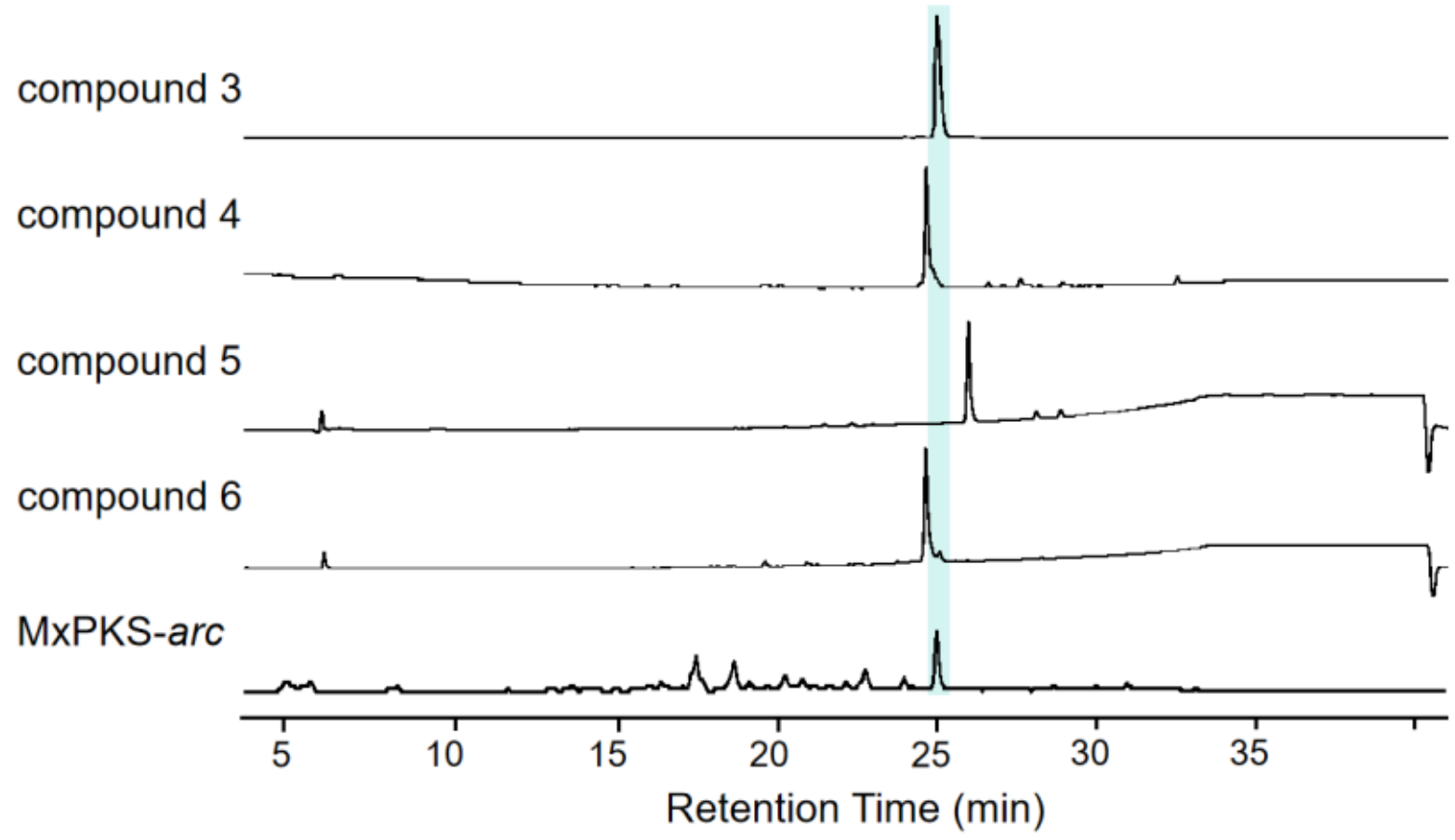

The position of compound **7** is clearly indicated in the figure. For compounds **8** and **9**, their retention times are almost identical under the applied HPLC conditions; therefore, they are jointly labeled as **8**/**9** in the chromatogram.

2. Cpd 2 is still missing a stereocenter.

>>> We previously determined the stereochemistry of **2**, as found in reference *https://doi.org/10.1021/acscentsci.3c01363*.

3. There is not clarity for how all the stereocenters and alkene configurations were determined for compounds 4–6. The authors state that they compared chemical shifts and coupling constants with that of cpd 3, but the authors do not provide coupling constants for all carbons with stereocenters. And in the macrocycle, given its presumed flexibility, are coupling constants adequate for determination at C16 and C17? The 10,11 and 12,13 alkenes in cpd 4 and the 11,12 alkene in cpd 5 does not have spectroscopic support (NOE, coupling constants) or text description for their E/Z designations.

>>> We thank the reviewer for the careful assessment of the structural elucidation of compounds **4**–**6**. To address the lack of clarity regarding stereocenters and alkene configurations, we have now provided all relevant coupling constants for stereocenters in Tables S3–S5 of the revised Supporting Information. Furthermore, we have additionally included key NOESY correlations for compounds **5** and **6** in Figure S26. For compound **4**, however, we observed significant degradation, and the remaining amount was too low to acquire a reliable NOESY spectrum. Despite this limitation, compounds **3**–**6** actually share the same biosynthetic origin, and the absolute configuration of compound **3** has been unambiguously established by single-crystal X-ray diffraction (see reference https://dx.doi.org/10.1021/acs.orglett.1c00265). Therefore, the stereochemistry (*16S, 17R)* and geometry of *trans* double bond $\Delta^{6,7}$ of compounds **3**–**6** were determined by the co-linearity of *arc* modular PKS assembly line. Furthermore, the close similarity in their ^{1}H NMR chemical shifts and coupling constants also allowed a confident assignment by

analogy. For the alkene geometries, coupling constants for the relevant double bonds ($\Delta^{10,11}$ and $\Delta^{12,13}$ in compound **4,** $\Delta^{11,12}$ in compound **5**) are now reported in Tables S3–S5. To resolve signal overlap, we changed the NMR solvent to pyridine-d_5 with two drops of D_2O, which fully separated the alkene proton signals and enabled accurate determination of coupling constants, observable NOE correlations supporting the *E*/*Z* assignments have also been added. The combined evidence from coupling constants, NOE data, and biosynthetic analogy to compound **3,** firmly supported our stereo assignments of compounds **4**–**6**. The corresponding text descriptions have been incorporated into the "*De novo* structural elucidation" section.

4. SI page 34 has a section titled "SUPPORTING SPECTRA OF NEW COMPOUNDS" but many of these are known compounds.

>>> The title "SUPPORTING SPECTRA OF NEW COMPOUNDS" was retained from the initial submission. During the previous revision, we added spectral data for several known compounds but forgot to update the section title accordingly. We have now corrected it to "SUPPORTING SPECTRA OF THE ISOLATED COMPOUNDS" in the revised supplementary information.

Reviewer #3 (Remarks to the Author):

I co-reviewed this manuscript with one of the reviewers who provided the listed reports. This is part of the Nature Communications initiative to facilitate training in peer review and to provide appropriate recognition for Early Career Researchers who co-review manuscripts.

>>> Thanks for co-reviewing and your valuable comments.

Reviewer #1 (Remarks to the Author):

Overall, the reviewer comments were well addressed and the manuscript is much improved overall, both in writing and in data presentation and justification. One very minor comment remains that affects the broader readership for the journal. While the clarity has improved, word choices remain in the introduction that could be simplified, including “fraught with challenges”, “thwarted", "attractive vehicle", "ineluctably teeming with obstacles"... Similarly, a few terms in the Discussion feel somewhat more formal or stylistically elevated compared to the straightforward tone of the rest of the manuscript, such as “protracted timelines,” “circumventing,” “obviates,” “enticing opportunity” . These are not incorrect but simplifying them could further improve readability and maintain a more consistent tone.

>>> Thanks for your valuable suggestions. We simplify the overly formal or ornate phrases in the Introduction and Discussion sections to improve readability and maintain a consistent, straightforward tone throughout the manuscript.

Reviewer #2 (Remarks to the Author):

The authors addressed all my concerns.

>>> We sincerely appreciate your affirmation.
